# Supplementary material for: Bats as ecosystem engineers in iron ore caves in the Carajás National Forest, Brazilian Amazonia
Source: PLoS One. 2023 May 11;18(5):e0267870. doi: 10.1371/journal.pone.0267870 (PMC10174506; doi:10.1371/journal.pone.0267870)
Supplement: S1 Fig — Scatterplot graph considering the length (in meters) and volume (in cubic meters) of 1,309 caves in the Carajás National Forest, Pará State, Brazilian Amazonia. Solid diamonds represent active and inactive bat caves, which tend to be longer and bulkier than the majority of the regional caves. Cave size alone does not explain bat choice for these specific bat caves, and the deep corrosion signs and the higher richness and variety of speleothems were observed only in bat caves. (DOCX) [file pone.0267870.s001.docx]

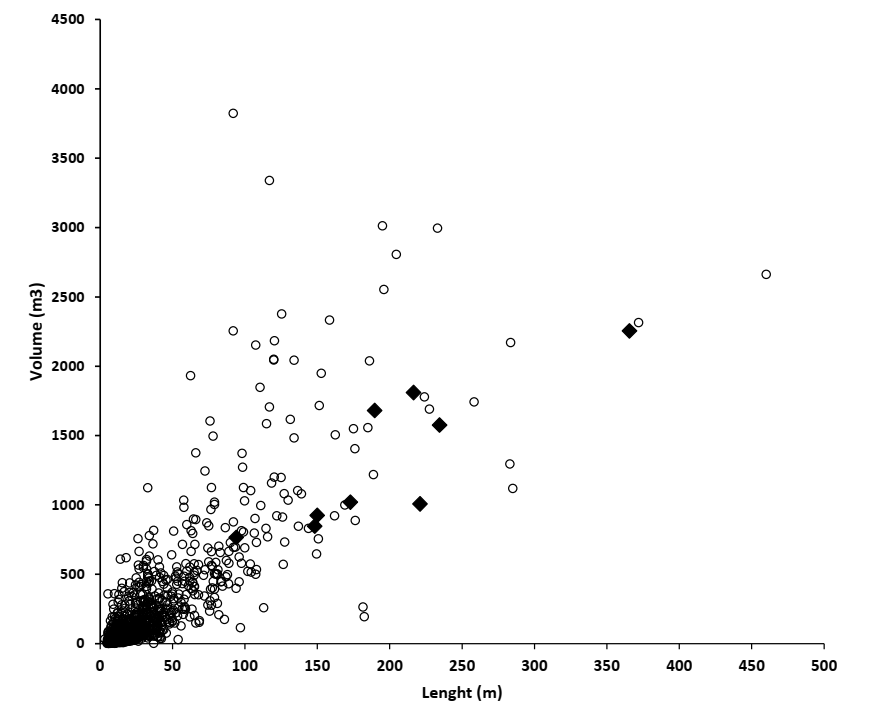


Figure S1: Scatterplot graph considering the length (in meters) and volume (in cubic meters) of 1,309 caves in the Carajás National Forest, Pará State, Brazilian Amazonia. Solid diamonds represent active and inactive bat caves, which tend to be longer and bulkier than the majority of the regional caves. Cave size alone does not explain bat choice for these specific bat caves, and the deep corrosion signs and the higher richness and variety of speleothems were observed only in bat caves.
