# Supplementary material for: Bats as ecosystem engineers in iron ore caves in the Carajás National Forest, Brazilian Amazonia
Source: PLoS One. 2023 May 11;18(5):e0267870. doi: 10.1371/journal.pone.0267870 (PMC10174506; doi:10.1371/journal.pone.0267870)
Supplement: S1 Table — Coordinates, dimensions (length, area and volume), and information on their biogenic morphology and speleothems for 10 caves observed. (DOCX) [file pone.0267870.s002.docx]

**Table S1**: Active and inactive bat caves studied in the Carajás National Forest, Pará State, Brazilian Amazonia, with cave dimensions and information on their biogenic morphology and speleothems observed.

| **Cave** | **Cave coordinates**  (UTM E, UTM N) SAD69/Z22M | | **Cave status** | **Guano deposit** | **Lenght**  **(m)** | **Area**  **(m^2^)** | **Volume (m^3^)** | **Biogenic morphology** | **Speleothems** |
| --- | --- | --- | --- | --- | --- | --- | --- | --- | --- |
| N5SM2-0099 | 596307 | 9321757 | Active bat cave | large | 189 | 582 | 1680 | Very developed biogenic morphogenesis. Several compartments, gullying, pinnacles, dripping holes, coralloids, cannelure | Crusts, stalactites, stalagmites, coralloids |
| N5SM2-0019 | 596323 | 9321708 | Active bat cave | large | ~ 150 | 351 | 925 | Very developed biogenic morphogenesis. |  |
| S11A-0036 | 560311 | 9301740 | Active bat cave | medium | 190 | - | - | Very developed biogenic morphogenesis. Several compartments, more than one level, gullying, pinnacles, dripping holes | Crusts, columns, stalactites, stalagmites, coralloids |
| N3-0023 | 586379 | 9331983 | Inactive bat cave. Currently with small colony | medium | 365 | 990 | 2254 | Very developed biogenic morphogenesis. Several compartments, more than one level, gullying, pinnacles, dripping holes, cannelure |  |
| N4WS-0067 | 589452 | 9328690 | Inactive bat cave. Currently with small colony. | small | 234 | 684 | 1575 | Very developed biogenic morphogenesis. Several compartments, more than one level, gullying, pinnacles, dripping holes, cannelure |  |
| S11B-0094 | 567130 | 9298209 | Inactive bat cave. Currently with small colony. | small | 216 | 1116 | 1809 | Very developed biogenic morphogenesis | Crusts, column, stalactites, stalagmites, coralloids |
| N4WS-0072 | 589675 | 9328540 | Inactive bat cave. Currently with small colony. | small | 148 | 398 | 847 | Moderate biogenic morphogenesis |  |
| S11C-0041 | 568528 | 9294526 | Inactive bat cave. Currently with small colony. | small | 173 | 647 | 1020 | Moderate biogenic morphogenesis |  |
| S11D-0083 | 574802 | 9292886 | Inactive bat cave. Currently small colony | small | 94 | 418 | 765 | Moderate biogenic morphogenesis |  |
| N5S-0063 | 595672 | 9325284 | Inactive bat cave. Currently with small colony. | medium | 221 | 666 | 1007 | Less developed biogenic morphogenesis | Dripping holes |
