# Supplementary material for: Bats as ecosystem engineers in iron ore caves in the Carajás National Forest, Brazilian Amazonia
Source: PLoS One. 2023 May 11;18(5):e0267870. doi: 10.1371/journal.pone.0267870 (PMC10174506; doi:10.1371/journal.pone.0267870)
Supplement: S2 Table — Cave coordinates and description of samples collected and analyses performed. * Denotes active and inactive bat caves, as described in S1 Table. (DOCX) [file pone.0267870.s003.docx]

**Table S2**: Caves studied in the Carajás National Forest, Pará State, Brazilian Amazonia, with coordinates and description of samples collected and analyses performed. * Denotes active and inactive bat caves, as described in Table S1.

| **Cave** | **Coordinates (SAD69/22M)** | |  |
| --- | --- | --- | --- |
|  | UTM E | UTM N | **Samples collected and analyses** |
| N5SM2-0099* | 596307 | 9321757 | Stalactite, speleothem mineralogy and chemistry, excavated trench, radiocarbon data, guano chemistry |
| N5SM2-0019* | 596323 | 9321708 | Guano chemistry, superficial and circulating water pH |
| N3-0023* | 586379 | 9331983 | Superficial and circulating water pH, water chemistry, excavated trench, guano chemistry, radiocarbon data, stalactite, speleothem mineralogy and chemistry |
| N4WS-0067* | 589452 | 9328690 | Superficial and dripping water pH, sampling hole, radiocarbon data, stalactite, speleothem mineralogy and chemistry |
| N4WS-0072* | 589672 | 9328580 | Superficial and dripping water pH, water chemistry, guano chemistry, stalactite, speleothem mineralogy and chemistry |
| S11A-0036* | 560311 | 9301740 | Superficial and dripping water pH, guano chemistry, stalactite, speleothem mineralogy and chemistry |
| S11B-0094* | 567130 | 9298208 | Superficial and dripping water pH, excavated trench, radiocarbon data, stalagmite, speleothem mineralogy and chemistry |
| S11C-0041* | 568524 | 9294526 | Guano chemistry, excavated trench, radiocarbon data, stalactite, speleothem mineralogy and chemistry |
| S11D-0083* | 574802 | 9292886 | Guano chemistry, speleothem mineralogy and chemistry |
| N5S-0063* | 595682 | 9325264 | Excavated trench, radiocarbon data |
| S11B-0168 | 560261 | 9301699 | Superficial and dripping water pH, guano chemistry |
| CAV-0041 | 570340 | 9290825 | Speleothem mineralogy and chemistry |
| CRIS-0020 | 645071 | 9289468 | Speleothem |
| N3-0074 | 585168 | 9331968 | Stalactite, speleothem mineralogy and chemistry |
| N4E-0014 | 592917 | 9332512 | speleothem mineralogy and chemistry |
| N4E-0023 | 592031 | 9333013 | speleothem mineralogy and chemistry |
| N4E-0033 | 592972 | 9332288 | speleothem mineralogy and chemistry |
| N4E-0072 | 593664 | 9333140 | speleothem mineralogy and chemistry |
| N4E-0092 | 593106 | 9332366 | speleothem mineralogy and chemistry |
| N4WS-0015 | 589697 | 9329388 | Stalactite, speleothem mineralogy and chemistry |
| N5E-0002 | 597594 | 9328450 | speleothem mineralogy and chemistry |
| N5E-0003 | 597245 | 9327356 | speleothem mineralogy and chemistry |
| N5E-0005 | 596329 | 9327242 | speleothem mineralogy and chemistry |
| N5S-0004 | 595814 | 9325049 | speleothem mineralogy and chemistry |
| N5S-0011 | 596301 | 9325116 | Superficial and dripping water pH, water chemistry |
| N5S-0012 | 596768 | 9325320 | Superficial and dripping water pH, water chemistry |
| N5S-0014 | 595871 | 9325070 | speleothem mineralogy and chemistry |
| N5S-0015 | 595886 | 9325086 | speleothem mineralogy and chemistry |
| N5S-0017 | 597412 | 9327012 | speleothem mineralogy and chemistry |
| N5S-0021 | 596716 | 9327033 | speleothem mineralogy and chemistry |
| NV-0011 | 621068 | 9285015 | speleothem mineralogy and chemistry |
| S11-0007 | 583854 | 9286363 | speleothem mineralogy and chemistry |
| S11D-0001 | 571128 | 9292715 | speleothem mineralogy and chemistry |
| S11D-0012 | 570832 | 9292964 | speleothem mineralogy and chemistry |
| S11D-0047 | 575721 | 9291345 | speleothem mineralogy and chemistry |
| S11D-0055 | 575188 | 9291828 | speleothem mineralogy and chemistry |
| S11D-0078 | 575634 | 9293373 | speleothem mineralogy and chemistry |
| S11D-0094 | 575039 | 9293131 | speleothem mineralogy and chemistry |
| S11D-0096 | 574738 | 9293228 | speleothem mineralogy and chemistry |
| S11D-0101 | 570402 | 9293694 | speleothem mineralogy and chemistry |
| S11D-0121 |  |  | Guano chemistry |
