# Supplementary material for: Bats as ecosystem engineers in iron ore caves in the Carajás National Forest, Brazilian Amazonia
Source: PLoS One. 2023 May 11;18(5):e0267870. doi: 10.1371/journal.pone.0267870 (PMC10174506; doi:10.1371/journal.pone.0267870)
Supplement: S3 Table — * Denotes samples from active and inactive bat caves, as described in S1 Table; ** denotes samples from other caves; and SP denotes samples from superficial waters, including artificial lakes and drainages. (DOCX) [file pone.0267870.s004.docx]

**Table S3**: pH of superficial and circulating water samples collected in caves in the Carajás National Forest, Pará State, Brazilian Amazonia. * Denotes samples from active and inactive bat caves, as described in Tables S1; ** denotes samples from other non-bat caves; and ^SP^ denotes samples from superficial waters, including artificial lakes and drainages.

| **Sample** | **pH** |
| --- | --- |
| M2-0019-W03* | 6.3 |
| M2-0019-W04* | 7.58 |
| M2-0099-W01* | 5.07 |
| M2-0099-W02* | 6.79 |
| M2-0099-W03* | 1.46 |
| M2-0099-W04* | 1.48 |
| M2-0099-W05* | 3.21 |
| M2-0099-W06* | 6.68 |
| M2-0099-W07* | 7.12 |
| M2-0099-W08* | 7.38 |
| S11A-0036-W01* | 6.91 |
| S11A-0036-W02* | 4.93 |
| S11A-0036-W03* | 2.88 |
| S11A-0036-W04* | 2.89 |
| S11A-0036-W05* | 2.5 |
| S11A-0036-W06* | 4.87 |
| S11A-0036-W07* | 7.2 |
| S11A-0036-W08* | 2.77 |
| S11A-0036-W09* | 2.7 |
| S11A-0036-W10* | 2.86 |
| S11A-0036-W11* | 2.9 |
| N3-0023-W01* | 3.79 |
| N3-0023-W02* | 3.36 |
| N3-0023-W03* | 2.56 |
| N3-0023-W04* | 2.7 |
| N3-0023-W05* | 4.02 |
| N3-0023-W06* | 3.06 |
| N3-0023-W07* | 2.76 |
| N3-0023-W08* | 2.98 |
| N3-0023-W09* | 3.53 |
| N3-0023-W10* | 2.67 |
| N3-0023-W11* | 2.24 |
| N3-0023-W12* | 2.45 |
| N3-0023-W13* | 2.8 |
| N3-0023-W14* | 2.53 |
| N3-0023-W15* | 2.41 |
| N3-0023-W16* | 2.8 |
| N3-0023-W17* | 3.23 |
| S11B-0094-W01* | 3.55 |
| S11B-0094-W02* | 3.26 |
| S11B-0094-W03* | 3.96 |
| S11B-0094-W04* | 2.4 |
| S11B-0094-W05* | 2.73 |
| S11B-0094-W06* | 3.16 |
| S11B-0094-W07* | 5.78 |
| S11B-0094-W08* | 3.13 |
| N4WS-0067-W01* | 5.99 |
| N4WS-0067-W02* | 2.2 |
| N4WS-0067-W03* | 2.37 |
| S11B-0168-W01** | 7.59 |
| N5S11-W01** | 4.09 |
| N5S11-W02** | 3.81 |
| N5S11-W03** | 3.93 |
| N5S12-W01** | 3.91 |
| N5S12-W02** | 4.08 |
| N5S12-W03** | 4.7 |
| LA-01^SP^ | 5.05 |
| LA-02^SP^ | 5.02 |
| LC-01^SP^ | 5.11 |
| S11C-1^SP^ | 4.49 |
| S11C-1^SP^ | 4.61 |
| S11C-2^SP^ | 5.47 |
| S11C-3^SP^ | 4.69 |
| S11B-1^SP^ | 5.01 |
| LRT-1^SP^ | 5.52 |
| BC-1^SP^ | 4.82 |
