# Supplementary material for: Bats as ecosystem engineers in iron ore caves in the Carajás National Forest, Brazilian Amazonia
Source: PLoS One. 2023 May 11;18(5):e0267870. doi: 10.1371/journal.pone.0267870 (PMC10174506; doi:10.1371/journal.pone.0267870)
Supplement: S1 File — Reports issued by the Laboratório de Fertilizantes, Corretivos e Resíduos Orgânicos of the Escola Superior de Agricultura Luiz de Queiroz (ESALQ/USP) indicating organic matter and chemical composition of guano samples analyzed. (PDF) [file pone.0267870.s005.pdf]

Luís Piló
Rua Conselheiro Barbosa , 35 Apto. 301
Santa Tereza
31.010-510 - Belo Horizonte - MG

Número: 0016546.1-N - O.S.: 9819
Data de Finalização: 18/12/2019

**Proprietário:**	**Propriedade:**	**Material:**
IABS - Instituto Brasileiro de Desenvolvim	UFPE / Carajás	Mat. Org. sólido

IDENTIFICAÇÃO DA AMOSTRA

Nº Amostra: LFC-002179/2019
Identificação: N323-P1-C1 (C=camada)

DETERMINAÇÕES	RESULTADOS	
	BASE SECA (65 °C)	BASE ÚMIDA
pH (CaCl2 0,01 M)	-	4,5
Densidade (Residuo Organico)	-	0,68 g/cm3
Umidade (Residuo Orgânico) 60 - 65º C	-	30,44 %
Umidade (Residuo Orgânico) 110°C	-	5,04 %
Umidade Total	-	35,48 %
Materia Orgânica Total (Combustão)	67,24 %	46,77 %
Carbono Orgânico	35,75 %	24,87 %
Resíduo Mineral Total (R.M.T.)	25,52 %	17,75 %
Resíduo Mineral (R.M.)	25,01 %	17,40 %
Resíduo Mineral Insolúvel (R.M.I.)	0,50 %	0,35 %
Nitrogênio Total	5,58 %	3,88 %
Fosforo (P2O5) Total	9,09 %	6,32 %
Potassio (K2O) Total	1,67 %	1,16 %
Calcio (Ca) Total	7,49 %	5,21 %
Magnésio (Mg) Total	0,43 %	0,30 %
Enxofre (S) Total	1,67 %	1,16 %
Relação C/N	-	6
Cobre (Cu) Total	167 mg/kg	116 mg/kg
Mangânes (Mn) Total	482 mg/kg	335 mg/kg
Zinco (Zn) Total	758 mg/kg	527 mg/kg
Ferro (Fe) Total	18363 mg/kg	12773 mg/kg
Boro (B) Total	13 mg/kg	9 mg/kg
Sódio (Na) Total	2394 mg/kg	1665 mg/kg

Métodos: pH em CaCl2 0,01 M determinação potenciometria; Densidade (m/v); Umidade 60-65º, Umidade 110º e Umidade total determinação por umidade; Carbono Orgânico (CO) oxidação dicromato seguido de titulação;Nitrogênio total digestão sulfúrica (Kjeldahl); Fósforo (P2O5) determinação por espectrofotômetro pelo método com a solução de vanadomolibdica; Potássio (K2O) e Sódio (Na) fotometria de chama; Enxofre (S) gravimétrico de sulfato de bário; Cálcio (Ca), Magnésio (Mg), Cobre (Cu), Manganês (Mn), Zinco (ZN), Ferro (Fe) extração com HCl por espectrofotômetro de absorção atômica; Boro (B) espectrofotometria da azometina-H; Relação C/N cálculo (Ref.: BRASIL, 2017. Manual de Métodos Oficiais para Fertilizantes Minerais, Orgânicos e Corretivos. MAPA). Matéria Orgânica Total, Resíduo Mineral Insolúvel (RMI), Resíduo Mineral (RM) e Resíduo Mineral Total (RMT) por combustão em Mufla (Ref.: ALCARDE, José Carlos. Manual de Análise de Fertilizantes - Piracicaba: FEALQ, 2009.)

SIGNATÁRIO AUTORIZADO

Luís Piló
Rua Conselheiro Barbosa , 35 Apto. 301
Santa Tereza
31.010-510 - Belo Horizonte - MG

Número: 0016546.1-N - O.S.: 9819
Data de Finalização: 18/12/2019

Proprietário:	Propriedade:	Material:
IABS - Instituto Brasileiro de Desenvolvim	UFPE / Carajás	Mat. Org. sólido

IDENTIFICAÇÃO DA AMOSTRA

Nº Amostra: LFC-002180/2019
Identificação: N323-P1-C2

DETERMINAÇÕES	RESULTADOS	
	BASE SECA (65 °C)	BASE ÚMIDA
pH (CaCl2 0,01 M)	-	4,4
Densidade (Residuo Organico)	-	0,60 g/cm3
Umidade (Residuo Orgânico) 60 - 65º C	-	34,59 %
Umidade (Residuo Orgânico) 110°C	-	5,15 %
Umidade Total	-	39,74 %
Materia Orgânica Total (Combustão)	63,23 %	41,36 %
Carbono Orgânico	33,22 %	21,73 %
Resíduo Mineral Total (R.M.T.)	28,89 %	18,90 %
Resíduo Mineral (R.M.)	28,37 %	18,56 %
Resíduo Mineral Insolúvel (R.M.I.)	0,52 %	0,34 %
Nitrogênio Total	5,11 %	3,34 %
Fosforo (P2O5) Total	9,17 %	6,00 %
Potassio (K2O) Total	2,14 %	1,40 %
Calcio (Ca) Total	7,66 %	5,01 %
Magnésio (Mg) Total	0,26 %	0,17 %
Enxofre (S) Total	2,42 %	1,58 %
Relação C/N	-	7
Cobre (Cu) Total	136 mg/kg	89 mg/kg
Mangânes (Mn) Total	503 mg/kg	329 mg/kg
Zinco (Zn) Total	689 mg/kg	451 mg/kg
Ferro (Fe) Total	10634 mg/kg	6956 mg/kg
Boro (B) Total	15 mg/kg	10 mg/kg
Sódio (Na) Total	2331 mg/kg	1525 mg/kg

Métodos: pH em CaCl2 0,01 M determinação potenciometria; Densidade (m/v); Umidade 60-65º, Umidade 110º e Umidade total determinação por umidade; Carbono Orgânico (CO) oxidação dicromato seguido de titulação;Nitrogênio total digestão sulfúrica (Kjeldahl); Fósforo (P2O5) determinação por espectrofotômetro pelo método com a solução de vanadomolibdica; Potássio (K2O) e Sódio (Na) fotometria de chama; Enxofre (S) gravimétrico de sulfato de bário; Cálcio (Ca), Magnésio (Mg), Cobre (Cu), Manganês (Mn), Zinco (ZN), Ferro (Fe) extração com HCl por espectrofotômetro de absorção atômica; Boro (B) espectrofotometria da azometina-H; Relação C/N cálculo (Ref.: BRASIL, 2017. Manual de Métodos Oficiais para Fertilizantes Minerais, Orgânicos e Corretivos. MAPA). Matéria Orgânica Total, Resíduo Mineral Insolúvel (RMI), Resíduo Mineral (RM) e Resíduo Mineral Total (RMT) por combustão em Mufla (Ref.: ALCARDE, José Carlos. Manual de Análise de Fertilizantes - Piracicaba: FEALQ, 2009.)

SIGNATÁRIO AUTORIZADO

Luís Piló
Rua Conselheiro Barbosa , 35 Apto. 301
Santa Tereza
31.010-510 - Belo Horizonte - MG

Número: 0016546.1-N - O.S.: 9819
Data de Finalização: 18/12/2019

Proprietário:	Propriedade:	Material:
IABS - Instituto Brasileiro de Desenvolvim	UFPE / Carajás	Mat. Org. sólido

IDENTIFICAÇÃO DA AMOSTRA

Nº Amostra: LFC-002181/2019
Identificação: N323-P1-C3

DETERMINAÇÕES	RESULTADOS	
	BASE SECA (65 °C)	BASE ÚMIDA
pH (CaCl2 0,01 M)	-	4,5
Densidade (Residuo Organico)	-	0,57 g/cm3
Umidade (Residuo Orgânico) 60 - 65º C	-	31,83 %
Umidade (Residuo Orgânico) 110°C	-	4,93 %
Umidade Total	-	36,76 %
Materia Orgânica Total (Combustão)	70,34 %	47,95 %
Carbono Orgânico	37,17 %	25,34 %
Resíduo Mineral Total (R.M.T.)	22,43 %	15,29 %
Resíduo Mineral (R.M.)	21,78 %	14,85 %
Resíduo Mineral Insolúvel (R.M.I.)	0,65 %	0,44 %
Nitrogênio Total	5,59 %	3,81 %
Fosforo (P2O5) Total	5,46 %	3,72 %
Potassio (K2O) Total	2,14 %	1,46 %
Calcio (Ca) Total	7,63 %	5,20 %
Magnésio (Mg) Total	0,34 %	0,23 %
Enxofre (S) Total	2,02 %	1,38 %
Relação C/N	-	7
Cobre (Cu) Total	191 mg/kg	130 mg/kg
Mangânes (Mn) Total	666 mg/kg	454 mg/kg
Zinco (Zn) Total	1042 mg/kg	710 mg/kg
Ferro (Fe) Total	8978 mg/kg	6120 mg/kg
Boro (B) Total	32 mg/kg	22 mg/kg
Sódio (Na) Total	1976 mg/kg	1347 mg/kg

Métodos: pH em CaCl2 0,01 M determinação potenciometria; Densidade (m/v); Umidade 60-65º, Umidade 110º e Umidade total determinação por umidade; Carbono Orgânico (CO) oxidação dicromato seguido de titulação;Nitrogênio total digestão sulfúrica (Kjeldahl); Fósforo (P2O5) determinação por espectrofotômetro pelo método com a solução de vanadomolibdica; Potássio (K2O) e Sódio (Na) fotometria de chama; Enxofre (S) gravimétrico de sulfato de bário; Cálcio (Ca), Magnésio (Mg), Cobre (Cu), Manganês (Mn), Zinco (ZN), Ferro (Fe) extração com HCl por espectrofotômetro de absorção atômica; Boro (B) espectrofotometria da azometina-H; Relação C/N cálculo (Ref.: BRASIL, 2017. Manual de Métodos Oficiais para Fertilizantes Minerais, Orgânicos e Corretivos. MAPA). Matéria Orgânica Total, Resíduo Mineral Insolúvel (RMI), Resíduo Mineral (RM) e Resíduo Mineral Total (RMT) por combustão em Mufla (Ref.: ALCARDE, José Carlos. Manual de Análise de Fertilizantes - Piracicaba: FEALQ, 2009.)

SIGNATÁRIO AUTORIZADO

Av. Pádua Dias, 11 - Piracicaba-SP - CEP 13418-900 - Tel.: (19) 3417-2117 / 3417-2159 - lso.lab@usp.br - www.esalqsolos.com.br

Luís Piló
Rua Conselheiro Barbosa , 35 Apto. 301
Santa Tereza
31.010-510 - Belo Horizonte - MG

Número: 0016546.1-N - O.S.: 9819
Data de Finalização: 18/12/2019

Proprietário:	Propriedade:	Material:
IABS - Instituto Brasileiro de Desenvolvim	UFPE / Carajás	Mat. Org. sólido

IDENTIFICAÇÃO DA AMOSTRA

Nº Amostra: LFC-002182/2019
Identificação: N323-P1-C4

DETERMINAÇÕES	RESULTADOS	
	BASE SECA (65 °C)	BASE ÚMIDA
pH (CaCl2 0,01 M)	-	4,6
Densidade (Residuo Organico)	-	0,67 g/cm3
Umidade (Residuo Orgânico) 60 - 65º C	-	29,15 %
Umidade (Residuo Orgânico) 110°C	-	5,58 %
Umidade Total	-	34,73 %
Materia Orgânica Total (Combustão)	53,47 %	37,88 %
Carbono Orgânico	28,10 %	19,91 %
Resíduo Mineral Total (R.M.T.)	38,66 %	27,39 %
Resíduo Mineral (R.M.)	37,80 %	26,78 %
Resíduo Mineral Insolúvel (R.M.I.)	0,86 %	0,61 %
Nitrogênio Total	4,73 %	3,35 %
Fosforo (P2O5) Total	14,24 %	10,09 %
Potassio (K2O) Total	1,61 %	1,14 %
Calcio (Ca) Total	10,56 %	7,48 %
Magnésio (Mg) Total	0,27 %	0,19 %
Enxofre (S) Total	2,09 %	1,48 %
Relação C/N	-	6
Cobre (Cu) Total	247 mg/kg	175 mg/kg
Mangânes (Mn) Total	868 mg/kg	615 mg/kg
Zinco (Zn) Total	1098 mg/kg	778 mg/kg
Ferro (Fe) Total	20782 mg/kg	14724 mg/kg
Boro (B) Total	13 mg/kg	9 mg/kg
Sódio (Na) Total	1732 mg/kg	1227 mg/kg

Métodos: pH em CaCl2 0,01 M determinação potenciometria; Densidade (m/v); Umidade 60-65º, Umidade 110º e Umidade total determinação por umidade; Carbono Orgânico (CO) oxidação dicromato seguido de titulação;Nitrogênio total digestão sulfúrica (Kjeldahl); Fósforo (P2O5) determinação por espectrofotômetro pelo método com a solução de vanadomolibdica; Potássio (K2O) e Sódio (Na) fotometria de chama; Enxofre (S) gravimétrico de sulfato de bário; Cálcio (Ca), Magnésio (Mg), Cobre (Cu), Manganês (Mn), Zinco (ZN), Ferro (Fe) extração com HCl por espectrofotômetro de absorção atômica; Boro (B) espectrofotometria da azometina-H; Relação C/N cálculo (Ref.: BRASIL, 2017. Manual de Métodos Oficiais para Fertilizantes Minerais, Orgânicos e Corretivos. MAPA). Matéria Orgânica Total, Resíduo Mineral Insolúvel (RMI), Resíduo Mineral (RM) e Resíduo Mineral Total (RMT) por combustão em Mufla (Ref.: ALCARDE, José Carlos. Manual de Análise de Fertilizantes - Piracicaba: FEALQ, 2009.)

SIGNATÁRIO AUTORIZADO

Luís Piló
Rua Conselheiro Barbosa , 35 Apto. 301
Santa Tereza
31.010-510 - Belo Horizonte - MG

Número: 0016546.1-N - O.S.: 9819
Data de Finalização: 18/12/2019

Proprietário:	Propriedade:	Material:
IABS - Instituto Brasileiro de Desenvolvim	UFPE / Carajás	Mat. Org. sólido

IDENTIFICAÇÃO DA AMOSTRA

Nº Amostra: LFC-002183/2019
Identificação: N323-P1-C5

DETERMINAÇÕES	RESULTADOS	
	BASE SECA (65 °C)	BASE ÚMIDA
pH (CaCl2 0,01 M)	-	4,5
Densidade (Residuo Organico)	-	0,57 g/cm3
Umidade (Residuo Orgânico) 60 - 65º C	-	32,04 %
Umidade (Residuo Orgânico) 110°C	-	4,18 %
Umidade Total	-	36,22 %
Materia Orgânica Total (Combustão)	75,74 %	51,47 %
Carbono Orgânico	40,26 %	27,36 %
Resíduo Mineral Total (R.M.T.)	18,11 %	12,31 %
Resíduo Mineral (R.M.)	17,33 %	11,78 %
Resíduo Mineral Insolúvel (R.M.I.)	0,78 %	0,53 %
Nitrogênio Total	6,30 %	4,28 %
Fosforo (P2O5) Total	4,19 %	2,85 %
Potassio (K2O) Total	1,77 %	1,20 %
Calcio (Ca) Total	4,75 %	3,23 %
Magnésio (Mg) Total	0,29 %	0,20 %
Enxofre (S) Total	1,49 %	1,01 %
Relação C/N	-	6
Cobre (Cu) Total	269 mg/kg	183 mg/kg
Mangânes (Mn) Total	867 mg/kg	589 mg/kg
Zinco (Zn) Total	1283 mg/kg	872 mg/kg
Ferro (Fe) Total	10284 mg/kg	6989 mg/kg
Boro (B) Total	35 mg/kg	24 mg/kg
Sódio (Na) Total	2057 mg/kg	1398 mg/kg

Métodos: pH em CaCl2 0,01 M determinação potenciometria; Densidade (m/v); Umidade 60-65º, Umidade 110º e Umidade total determinação por umidade; Carbono Orgânico (CO) oxidação dicromato seguido de titulação;Nitrogênio total digestão sulfúrica (Kjeldahl); Fósforo (P2O5) determinação por espectrofotômetro pelo método com a solução de vanadomolibdica; Potássio (K2O) e Sódio (Na) fotometria de chama; Enxofre (S) gravimétrico de sulfato de bário; Cálcio (Ca), Magnésio (Mg), Cobre (Cu), Manganês (Mn), Zinco (ZN), Ferro (Fe) extração com HCl por espectrofotômetro de absorção atômica; Boro (B) espectrofotometria da azometina-H; Relação C/N cálculo (Ref.: BRASIL, 2017. Manual de Métodos Oficiais para Fertilizantes Minerais, Orgânicos e Corretivos. MAPA). Matéria Orgânica Total, Resíduo Mineral Insolúvel (RMI), Resíduo Mineral (RM) e Resíduo Mineral Total (RMT) por combustão em Mufla (Ref.: ALCARDE, José Carlos. Manual de Análise de Fertilizantes - Piracicaba: FEALQ, 2009.)

SIGNATÁRIO AUTORIZADO

Luís Piló
Rua Conselheiro Barbosa , 35 Apto. 301
Santa Tereza
31.010-510 - Belo Horizonte - MG

Número: 0016546.1-N - O.S.: 9819
Data de Finalização: 18/12/2019

Proprietário:	Propriedade:	Material:
IABS - Instituto Brasileiro de Desenvolvim	UFPE / Carajás	Mat. Org. sólido

IDENTIFICAÇÃO DA AMOSTRA

Nº Amostra: LFC-002184/2019
Identificação: N323-P1-C6

DETERMINAÇÕES	RESULTADOS	
	BASE SECA (65 °C)	BASE ÚMIDA
pH (CaCl2 0,01 M)	-	4,6
Densidade (Residuo Organico)	-	0,54 g/cm3
Umidade (Residuo Orgânico) 60 - 65º C	-	23,69 %
Umidade (Residuo Orgânico) 110°C	-	6,12 %
Umidade Total	-	29,81 %
Materia Orgânica Total (Combustão)	51,55 %	39,34 %
Carbono Orgânico	27,05 %	20,64 %
Resíduo Mineral Total (R.M.T.)	40,44 %	30,86 %
Resíduo Mineral (R.M.)	39,10 %	29,84 %
Resíduo Mineral Insolúvel (R.M.I.)	1,34 %	1,02 %
Nitrogênio Total	3,72 %	2,84 %
Fosforo (P2O5) Total	11,43 %	8,72 %
Potassio (K2O) Total	1,47 %	1,12 %
Calcio (Ca) Total	14,99 %	11,44 %
Magnésio (Mg) Total	0,26 %	0,20 %
Enxofre (S) Total	3,01 %	2,30 %
Relação C/N	-	7
Cobre (Cu) Total	279 mg/kg	213 mg/kg
Mangânes (Mn) Total	885 mg/kg	675 mg/kg
Zinco (Zn) Total	1136 mg/kg	867 mg/kg
Ferro (Fe) Total	18323 mg/kg	13982 mg/kg
Boro (B) Total	9 mg/kg	7 mg/kg
Sódio (Na) Total	1519 mg/kg	1159 mg/kg

Métodos: pH em CaCl2 0,01 M determinação potenciometria; Densidade (m/v); Umidade 60-65º, Umidade 110º e Umidade total determinação por umidade; Carbono Orgânico (CO) oxidação dicromato seguido de titulação;Nitrogênio total digestão sulfúrica (Kjeldahl); Fósforo (P2O5) determinação por espectrofotômetro pelo método com a solução de vanadomolibdica; Potássio (K2O) e Sódio (Na) fotometria de chama; Enxofre (S) gravimétrico de sulfato de bário; Cálcio (Ca), Magnésio (Mg), Cobre (Cu), Manganês (Mn), Zinco (ZN), Ferro (Fe) extração com HCl por espectrofotômetro de absorção atômica; Boro (B) espectrofotometria da azometina-H; Relação C/N cálculo (Ref.: BRASIL, 2017. Manual de Métodos Oficiais para Fertilizantes Minerais, Orgânicos e Corretivos. MAPA). Matéria Orgânica Total, Resíduo Mineral Insolúvel (RMI), Resíduo Mineral (RM) e Resíduo Mineral Total (RMT) por combustão em Mufla (Ref.: ALCARDE, José Carlos. Manual de Análise de Fertilizantes - Piracicaba: FEALQ, 2009.)

SIGNATÁRIO AUTORIZADO

Luís Piló
Rua Conselheiro Barbosa , 35 Apto. 301
Santa Tereza
31.010-510 - Belo Horizonte - MG

Número: 0016546.1-N - O.S.: 9819
Data de Finalização: 18/12/2019

Proprietário:	Propriedade:	Material:
IABS - Instituto Brasileiro de Desenvolvim	UFPE / Carajás	Mat. Org. sólido

IDENTIFICAÇÃO DA AMOSTRA

Nº Amostra: LFC-002185/2019
Identificação: N323-P1-C7

DETERMINAÇÕES	RESULTADOS	
	BASE SECA (65 °C)	BASE ÚMIDA
pH (CaCl2 0,01 M)	-	4,5
Densidade (Residuo Organico)	-	0,72 g/cm3
Umidade (Residuo Orgânico) 60 - 65º C	-	19,32 %
Umidade (Residuo Orgânico) 110°C	-	2,28 %
Umidade Total	-	21,60 %
Materia Orgânica Total (Combustão)	57,02 %	46,00 %
Carbono Orgânico	30,13 %	24,31 %
Resíduo Mineral Total (R.M.T.)	40,16 %	32,40 %
Resíduo Mineral (R.M.)	35,71 %	28,81 %
Resíduo Mineral Insolúvel (R.M.I.)	4,45 %	3,59 %
Nitrogênio Total	5,68 %	4,58 %
Fosforo (P2O5) Total	14,82 %	11,96 %
Potassio (K2O) Total	1,34 %	1,08 %
Calcio (Ca) Total	2,37 %	1,91 %
Magnésio (Mg) Total	0,26 %	0,21 %
Enxofre (S) Total	0,29 %	0,23 %
Relação C/N	-	5
Cobre (Cu) Total	1144 mg/kg	923 mg/kg
Mangânes (Mn) Total	1119 mg/kg	903 mg/kg
Zinco (Zn) Total	1673 mg/kg	1350 mg/kg
Ferro (Fe) Total	71151 mg/kg	57405 mg/kg
Boro (B) Total	11 mg/kg	9 mg/kg
Sódio (Na) Total	1542 mg/kg	1244 mg/kg

Métodos: pH em CaCl2 0,01 M determinação potenciometria; Densidade (m/v); Umidade 60-65º, Umidade 110º e Umidade total determinação por umidade; Carbono Orgânico (CO) oxidação dicromato seguido de titulação;Nitrogênio total digestão sulfúrica (Kjeldahl); Fósforo (P2O5) determinação por espectrofotômetro pelo método com a solução de vanadomolibdica; Potássio (K2O) e Sódio (Na) fotometria de chama; Enxofre (S) gravimétrico de sulfato de bário; Cálcio (Ca), Magnésio (Mg), Cobre (Cu), Manganês (Mn), Zinco (ZN), Ferro (Fe) extração com HCl por espectrofotômetro de absorção atômica; Boro (B) espectrofotometria da azometina-H; Relação C/N cálculo (Ref.: BRASIL, 2017. Manual de Métodos Oficiais para Fertilizantes Minerais, Orgânicos e Corretivos. MAPA). Matéria Orgânica Total, Resíduo Mineral Insolúvel (RMI), Resíduo Mineral (RM) e Resíduo Mineral Total (RMT) por combustão em Mufla (Ref.: ALCARDE, José Carlos. Manual de Análise de Fertilizantes - Piracicaba: FEALQ, 2009.)

SIGNATÁRIO AUTORIZADO

Luís Piló
Rua Conselheiro Barbosa , 35 Apto. 301
Santa Tereza
31.010-510 - Belo Horizonte - MG

Número: 0016546.1-N - O.S.: 9819
Data de Finalização: 18/12/2019

**Proprietário:**	**Propriedade:**	**Material:**
IABS - Instituto Brasileiro de Desenvolvim	UFPE / Carajás	Mat. Org. sólido

IDENTIFICAÇÃO DA AMOSTRA

Nº Amostra: LFC-002186/2019
Identificação: M2-99-C1

DETERMINAÇÕES	RESULTADOS	
	BASE SECA (65 °C)	BASE ÚMIDA
pH (CaCl2 0,01 M)	-	5,4
Densidade (Residuo Organico)	-	0,66 g/cm3
Umidade (Residuo Orgânico) 60 - 65º C	-	40,04 %
Umidade (Residuo Orgânico) 110°C	-	4,70 %
Umidade Total	-	44,74 %
Materia Orgânica Total (Combustão)	77,37 %	46,39 %
Carbono Orgânico	40,91 %	24,53 %
Resíduo Mineral Total (R.M.T.)	14,79 %	8,87 %
Resíduo Mineral (R.M.)	14,48 %	8,68 %
Resíduo Mineral Insolúvel (R.M.I.)	0,32 %	0,19 %
Nitrogênio Total	9,26 %	5,55 %
Fosforo (P2O5) Total	7,94 %	4,76 %
Potassio (K2O) Total	2,08 %	1,25 %
Calcio (Ca) Total	1,12 %	0,67 %
Magnésio (Mg) Total	0,97 %	0,58 %
Enxofre (S) Total	0,05 %	0,03 %
Relação C/N	-	4
Cobre (Cu) Total	339 mg/kg	203 mg/kg
Mangânes (Mn) Total	447 mg/kg	268 mg/kg
Zinco (Zn) Total	1119 mg/kg	671 mg/kg
Ferro (Fe) Total	5222 mg/kg	3131 mg/kg
Boro (B) Total	8 mg/kg	5 mg/kg
Sódio (Na) Total	6770 mg/kg	4059 mg/kg

Métodos: pH em CaCl2 0,01 M determinação potenciometria; Densidade (m/v); Umidade 60-65º, Umidade 110º e Umidade total determinação por umidade; Carbono Orgânico (CO) oxidação dicromato seguido de titulação;Nitrogênio total digestão sulfúrica (Kjeldahl); Fósforo (P2O5) determinação por espectrofotômetro pelo método com a solução de vanadomolibdica; Potássio (K2O) e Sódio (Na) fotometria de chama; Enxofre (S) gravimétrico de sulfato de bário; Cálcio (Ca), Magnésio (Mg), Cobre (Cu), Manganês (Mn), Zinco (ZN), Ferro (Fe) extração com HCl por espectrofotômetro de absorção atômica; Boro (B) espectrofotometria da azometina-H; Relação C/N cálculo (Ref.: BRASIL, 2017. Manual de Métodos Oficiais para Fertilizantes Minerais, Orgânicos e Corretivos. MAPA). Matéria Orgânica Total, Resíduo Mineral Insolúvel (RMI), Resíduo Mineral (RM) e Resíduo Mineral Total (RMT) por combustão em Mufla (Ref.: ALCARDE, José Carlos. Manual de Análise de Fertilizantes - Piracicaba: FEALQ, 2009.)

SIGNATÁRIO AUTORIZADO

Luís Piló
Rua Conselheiro Barbosa , 35 Apto. 301
Santa Tereza
31.010-510 - Belo Horizonte - MG

Número: 0016546.1-N - O.S.: 9819
Data de Finalização: 18/12/2019

Proprietário:	Propriedade:	Material:
IABS - Instituto Brasileiro de Desenvolvim	UFPE / Carajás	Mat. Org. sólido

IDENTIFICAÇÃO DA AMOSTRA

Nº Amostra: LFC-002187/2019
Identificação: M2-99-C2

DETERMINAÇÕES	RESULTADOS	
	BASE SECA (65 °C)	BASE ÚMIDA
pH (CaCl2 0,01 M)	-	4,9
Densidade (Residuo Organico)	-	0,67 g/cm3
Umidade (Residuo Orgânico) 60 - 65º C	-	38,08 %
Umidade (Residuo Orgânico) 110°C	-	4,62 %
Umidade Total	-	42,70 %
Materia Orgânica Total (Combustão)	68,52 %	42,43 %
Carbono Orgânico	36,09 %	22,35 %
Resíduo Mineral Total (R.M.T.)	24,01 %	14,87 %
Resíduo Mineral (R.M.)	22,87 %	14,16 %
Resíduo Mineral Insolúvel (R.M.I.)	1,15 %	0,71 %
Nitrogênio Total	8,19 %	5,07 %
Fosforo (P2O5) Total	9,82 %	6,08 %
Potassio (K2O) Total	2,37 %	1,47 %
Calcio (Ca) Total	1,94 %	1,20 %
Magnésio (Mg) Total	1,57 %	0,97 %
Enxofre (S) Total	0,87 %	0,54 %
Relação C/N	-	4
Cobre (Cu) Total	2038 mg/kg	1262 mg/kg
Mangânes (Mn) Total	786 mg/kg	487 mg/kg
Zinco (Zn) Total	8587 mg/kg	5317 mg/kg
Ferro (Fe) Total	20229 mg/kg	12526 mg/kg
Boro (B) Total	5 mg/kg	3 mg/kg
Sódio (Na) Total	5336 mg/kg	3304 mg/kg

Métodos: pH em CaCl2 0,01 M determinação potenciometria; Densidade (m/v); Umidade 60-65º, Umidade 110º e Umidade total determinação por umidade; Carbono Orgânico (CO) oxidação dicromato seguido de titulação;Nitrogênio total digestão sulfúrica (Kjeldahl); Fósforo (P2O5) determinação por espectrofotômetro pelo método com a solução de vanadomolibdica; Potássio (K2O) e Sódio (Na) fotometria de chama; Enxofre (S) gravimétrico de sulfato de bário; Cálcio (Ca), Magnésio (Mg), Cobre (Cu), Manganês (Mn), Zinco (ZN), Ferro (Fe) extração com HCl por espectrofotômetro de absorção atômica; Boro (B) espectrofotometria da azometina-H; Relação C/N cálculo (Ref.: BRASIL, 2017. Manual de Métodos Oficiais para Fertilizantes Minerais, Orgânicos e Corretivos. MAPA). Matéria Orgânica Total, Resíduo Mineral Insolúvel (RMI), Resíduo Mineral (RM) e Resíduo Mineral Total (RMT) por combustão em Mufla (Ref.: ALCARDE, José Carlos. Manual de Análise de Fertilizantes - Piracicaba: FEALQ, 2009.)

SIGNATÁRIO AUTORIZADO

Av. Pádua Dias, 11 - Piracicaba-SP - CEP 13418-900 - Tel.: (19) 3417-2117 / 3417-2159 - lso.lab@usp.br - www.esalqsolos.com.br

Luís Piló
Rua Conselheiro Barbosa , 35 Apto. 301
Santa Tereza
31.010-510 - Belo Horizonte - MG

Número: 0016546.1-N - O.S.: 9819
Data de Finalização: 18/12/2019

Proprietário:	Propriedade:	Material:
IABS - Instituto Brasileiro de Desenvolvim	UFPE / Carajás	Mat. Org. sólido

IDENTIFICAÇÃO DA AMOSTRA

Nº Amostra: LFC-002188/2019
Identificação: M2-99-C3

DETERMINAÇÕES	RESULTADOS	
	BASE SECA (65 °C)	BASE ÚMIDA
pH (CaCl2 0,01 M)	-	3,9
Densidade (Residuo Organico)	-	0,70 g/cm3
Umidade (Residuo Orgânico) 60 - 65º C	-	41,44 %
Umidade (Residuo Orgânico) 110°C	-	4,16 %
Umidade Total	-	45,60 %
Materia Orgânica Total (Combustão)	64,99 %	38,06 %
Carbono Orgânico	34,02 %	19,92 %
Resíduo Mineral Total (R.M.T.)	27,92 %	16,35 %
Resíduo Mineral (R.M.)	26,76 %	15,67 %
Resíduo Mineral Insolúvel (R.M.I.)	1,16 %	0,68 %
Nitrogênio Total	6,88 %	4,03 %
Fosforo (P2O5) Total	5,87 %	3,44 %
Potassio (K2O) Total	2,19 %	1,28 %
Calcio (Ca) Total	5,43 %	3,18 %
Magnésio (Mg) Total	0,24 %	0,14 %
Enxofre (S) Total	3,21 %	1,88 %
Relação C/N	-	5
Cobre (Cu) Total	1988 mg/kg	1164 mg/kg
Mangânes (Mn) Total	567 mg/kg	332 mg/kg
Zinco (Zn) Total	9063 mg/kg	5307 mg/kg
Ferro (Fe) Total	16817 mg/kg	9848 mg/kg
Boro (B) Total	3 mg/kg	2 mg/kg
Sódio (Na) Total	6784 mg/kg	3973 mg/kg

Métodos: pH em CaCl2 0,01 M determinação potenciometria; Densidade (m/v); Umidade 60-65º, Umidade 110º e Umidade total determinação por umidade; Carbono Orgânico (CO) oxidação dicromato seguido de titulação;Nitrogênio total digestão sulfúrica (Kjeldahl); Fósforo (P2O5) determinação por espectrofotômetro pelo método com a solução de vanadomolibdica; Potássio (K2O) e Sódio (Na) fotometria de chama; Enxofre (S) gravimétrico de sulfato de bário; Cálcio (Ca), Magnésio (Mg), Cobre (Cu), Manganês (Mn), Zinco (ZN), Ferro (Fe) extração com HCl por espectrofotômetro de absorção atômica; Boro (B) espectrofotometria da azometina-H; Relação C/N cálculo (Ref.: BRASIL, 2017. Manual de Métodos Oficiais para Fertilizantes Minerais, Orgânicos e Corretivos. MAPA). Matéria Orgânica Total, Resíduo Mineral Insolúvel (RMI), Resíduo Mineral (RM) e Resíduo Mineral Total (RMT) por combustão em Mufla (Ref.: ALCARDE, José Carlos. Manual de Análise de Fertilizantes - Piracicaba: FEALQ, 2009.)

SIGNATÁRIO AUTORIZADO

Luís Piló
Rua Conselheiro Barbosa , 35 Apto. 301
Santa Tereza
31.010-510 - Belo Horizonte - MG

Número: 0016546.1-N - O.S.: 9819
Data de Finalização: 18/12/2019

Proprietário:	Propriedade:	Material:
IABS - Instituto Brasileiro de Desenvolvim	UFPE / Carajás	Mat. Org. sólido

IDENTIFICAÇÃO DA AMOSTRA

Nº Amostra: LFC-002189/2019
Identificação: M2-99-C4

DETERMINAÇÕES	RESULTADOS	
	BASE SECA (65 °C)	BASE ÚMIDA
pH (CaCl2 0,01 M)	-	3,5
Densidade (Residuo Organico)	-	0,70 g/cm3
Umidade (Residuo Orgânico) 60 - 65º C	-	37,97 %
Umidade (Residuo Orgânico) 110°C	-	2,05 %
Umidade Total	-	40,02 %
Materia Orgânica Total (Combustão)	59,39 %	36,84 %
Carbono Orgânico	31,10 %	19,29 %
Resíduo Mineral Total (R.M.T.)	37,30 %	23,14 %
Resíduo Mineral (R.M.)	35,39 %	21,95 %
Resíduo Mineral Insolúvel (R.M.I.)	1,92 %	1,19 %
Nitrogênio Total	6,50 %	4,03 %
Fosforo (P2O5) Total	14,73 %	9,14 %
Potassio (K2O) Total	2,51 %	1,56 %
Calcio (Ca) Total	1,13 %	0,70 %
Magnésio (Mg) Total	0,19 %	0,12 %
Enxofre (S) Total	0,48 %	0,30 %
Relação C/N	-	5
Cobre (Cu) Total	2616 mg/kg	1623 mg/kg
Mangânes (Mn) Total	750 mg/kg	465 mg/kg
Zinco (Zn) Total	8589 mg/kg	5328 mg/kg
Ferro (Fe) Total	88012 mg/kg	54594 mg/kg
Boro (B) Total	5 mg/kg	3 mg/kg
Sódio (Na) Total	5430 mg/kg	3368 mg/kg

Métodos: pH em CaCl2 0,01 M determinação potenciometria; Densidade (m/v); Umidade 60-65º, Umidade 110º e Umidade total determinação por umidade; Carbono Orgânico (CO) oxidação dicromato seguido de titulação;Nitrogênio total digestão sulfúrica (Kjeldahl); Fósforo (P2O5) determinação por espectrofotômetro pelo método com a solução de vanadomolibdica; Potássio (K2O) e Sódio (Na) fotometria de chama; Enxofre (S) gravimétrico de sulfato de bário; Cálcio (Ca), Magnésio (Mg), Cobre (Cu), Manganês (Mn), Zinco (ZN), Ferro (Fe) extração com HCl por espectrofotômetro de absorção atômica; Boro (B) espectrofotometria da azometina-H; Relação C/N cálculo (Ref.: BRASIL, 2017. Manual de Métodos Oficiais para Fertilizantes Minerais, Orgânicos e Corretivos. MAPA). Matéria Orgânica Total, Resíduo Mineral Insolúvel (RMI), Resíduo Mineral (RM) e Resíduo Mineral Total (RMT) por combustão em Mufla (Ref.: ALCARDE, José Carlos. Manual de Análise de Fertilizantes - Piracicaba: FEALQ, 2009.)

SIGNATÁRIO AUTORIZADO

Luís Piló
Rua Conselheiro Barbosa , 35 Apto. 301
Santa Tereza
31.010-510 - Belo Horizonte - MG

Número: 0016546.1-N - O.S.: 9819
Data de Finalização: 18/12/2019

Proprietário:	Propriedade:	Material:
IABS - Instituto Brasileiro de Desenvolvim	UFPE / Carajás	Mat. Org. sólido

IDENTIFICAÇÃO DA AMOSTRA

Nº Amostra: LFC-002190/2019
Identificação: N323-P2-C1

DETERMINAÇÕES	RESULTADOS	
	BASE SECA (65 °C)	BASE ÚMIDA
pH (CaCl2 0,01 M)	-	2,2
Densidade (Residuo Organico)	-	0,70 g/cm3
Umidade (Residuo Orgânico) 60 - 65º C	-	43,65 %
Umidade (Residuo Orgânico) 110°C	-	2,54 %
Umidade Total	-	46,19 %
Materia Orgânica Total (Combustão)	70,61 %	39,79 %
Carbono Orgânico	37,04 %	20,87 %
Resíduo Mineral Total (R.M.T.)	24,88 %	14,02 %
Resíduo Mineral (R.M.)	18,35 %	10,34 %
Resíduo Mineral Insolúvel (R.M.I.)	6,53 %	3,68 %
Nitrogênio Total	4,54 %	2,56 %
Fosforo (P2O5) Total	6,00 %	3,38 %
Potassio (K2O) Total	0,28 %	0,16 %
Calcio (Ca) Total	0,91 %	0,51 %
Magnésio (Mg) Total	0,07 %	0,04 %
Enxofre (S) Total	0,09 %	0,05 %
Relação C/N	-	8
Cobre (Cu) Total	4018 mg/kg	2264 mg/kg
Mangânes (Mn) Total	323 mg/kg	182 mg/kg
Zinco (Zn) Total	1232 mg/kg	694 mg/kg
Ferro (Fe) Total	40713 mg/kg	22942 mg/kg
Boro (B) Total	4 mg/kg	2 mg/kg
Sódio (Na) Total	417 mg/kg	235 mg/kg

Métodos: pH em CaCl2 0,01 M determinação potenciometria; Densidade (m/v); Umidade 60-65º, Umidade 110º e Umidade total determinação por umidade; Carbono Orgânico (CO) oxidação dicromato seguido de titulação;Nitrogênio total digestão sulfúrica (Kjeldahl); Fósforo (P2O5) determinação por espectrofotômetro pelo método com a solução de vanadomolibdica; Potássio (K2O) e Sódio (Na) fotometria de chama; Enxofre (S) gravimétrico de sulfato de bário; Cálcio (Ca), Magnésio (Mg), Cobre (Cu), Manganês (Mn), Zinco (ZN), Ferro (Fe) extração com HCl por espectrofotômetro de absorção atômica; Boro (B) espectrofotometria da azometina-H; Relação C/N cálculo (Ref.: BRASIL, 2017. Manual de Métodos Oficiais para Fertilizantes Minerais, Orgânicos e Corretivos. MAPA). Matéria Orgânica Total, Resíduo Mineral Insolúvel (RMI), Resíduo Mineral (RM) e Resíduo Mineral Total (RMT) por combustão em Mufla (Ref.: ALCARDE, José Carlos. Manual de Análise de Fertilizantes - Piracicaba: FEALQ, 2009.)

SIGNATÁRIO AUTORIZADO

Luís Piló
Rua Conselheiro Barbosa , 35 Apto. 301
Santa Tereza
31.010-510 - Belo Horizonte - MG

Número: 0016546.1-N - O.S.: 9819
Data de Finalização: 18/12/2019

Proprietário:	Propriedade:	Material:
IABS - Instituto Brasileiro de Desenvolvim	UFPE / Carajás	Mat. Org. sólido

IDENTIFICAÇÃO DA AMOSTRA

Nº Amostra: LFC-002191/2019
Identificação: N323-P2-C2

DETERMINAÇÕES	RESULTADOS	
	BASE SECA (65 °C)	BASE ÚMIDA
pH (CaCl2 0,01 M)	-	2,2
Densidade (Residuo Organico)	-	0,68 g/cm3
Umidade (Residuo Orgânico) 60 - 65º C	-	38,78 %
Umidade (Residuo Orgânico) 110°C	-	2,74 %
Umidade Total	-	41,52 %
Materia Orgânica Total (Combustão)	68,36 %	41,85 %
Carbono Orgânico	35,95 %	22,01 %
Resíduo Mineral Total (R.M.T.)	27,16 %	16,63 %
Resíduo Mineral (R.M.)	23,16 %	14,18 %
Resíduo Mineral Insolúvel (R.M.I.)	4,00 %	2,45 %
Nitrogênio Total	7,43 %	4,55 %
Fosforo (P2O5) Total	7,95 %	4,87 %
Potassio (K2O) Total	0,34 %	0,21 %
Calcio (Ca) Total	0,91 %	0,56 %
Magnésio (Mg) Total	0,07 %	0,04 %
Enxofre (S) Total	0,03 %	0,02 %
Relação C/N	-	5
Cobre (Cu) Total	5893 mg/kg	3608 mg/kg
Mangânes (Mn) Total	474 mg/kg	290 mg/kg
Zinco (Zn) Total	1209 mg/kg	740 mg/kg
Ferro (Fe) Total	55100 mg/kg	33732 mg/kg
Boro (B) Total	5 mg/kg	3 mg/kg
Sódio (Na) Total	688 mg/kg	421 mg/kg

Métodos: pH em CaCl2 0,01 M determinação potenciometria; Densidade (m/v); Umidade 60-65º, Umidade 110º e Umidade total determinação por umidade; Carbono Orgânico (CO) oxidação dicromato seguido de titulação;Nitrogênio total digestão sulfúrica (Kjeldahl); Fósforo (P2O5) determinação por espectrofotômetro pelo método com a solução de vanadomolibdica; Potássio (K2O) e Sódio (Na) fotometria de chama; Enxofre (S) gravimétrico de sulfato de bário; Cálcio (Ca), Magnésio (Mg), Cobre (Cu), Manganês (Mn), Zinco (ZN), Ferro (Fe) extração com HCl por espectrofotômetro de absorção atômica; Boro (B) espectrofotometria da azometina-H; Relação C/N cálculo (Ref.: BRASIL, 2017. Manual de Métodos Oficiais para Fertilizantes Minerais, Orgânicos e Corretivos. MAPA). Matéria Orgânica Total, Resíduo Mineral Insolúvel (RMI), Resíduo Mineral (RM) e Resíduo Mineral Total (RMT) por combustão em Mufla (Ref.: ALCARDE, José Carlos. Manual de Análise de Fertilizantes - Piracicaba: FEALQ, 2009.)

SIGNATÁRIO AUTORIZADO

Luís Piló
Rua Conselheiro Barbosa , 35 Apto. 301
Santa Tereza
31.010-510 - Belo Horizonte - MG

Número: 0016546.1-N - O.S.: 9819
Data de Finalização: 18/12/2019

Proprietário:	Propriedade:	Material:
IABS - Instituto Brasileiro de Desenvolvim	UFPE / Carajás	Mat. Org. sólido

IDENTIFICAÇÃO DA AMOSTRA

Nº Amostra: LFC-002192/2019
Identificação: N323-P2-C3

DETERMINAÇÕES	RESULTADOS	
	BASE SECA (65 °C)	BASE ÚMIDA
pH (CaCl2 0,01 M)	-	2,1
Densidade (Residuo Organico)	-	0,68 g/cm3
Umidade (Residuo Orgânico) 60 - 65º C	-	49,30 %
Umidade (Residuo Orgânico) 110°C	-	3,31 %
Umidade Total	-	52,61 %
Materia Orgânica Total (Combustão)	69,64 %	35,31 %
Carbono Orgânico	36,25 %	18,38 %
Resíduo Mineral Total (R.M.T.)	23,83 %	12,08 %
Resíduo Mineral (R.M.)	20,39 %	10,34 %
Resíduo Mineral Insolúvel (R.M.I.)	3,43 %	1,74 %
Nitrogênio Total	5,86 %	2,97 %
Fosforo (P2O5) Total	7,02 %	3,56 %
Potassio (K2O) Total	1,10 %	0,56 %
Calcio (Ca) Total	0,65 %	0,33 %
Magnésio (Mg) Total	0,04 %	0,02 %
Enxofre (S) Total	0,08 %	0,04 %
Relação C/N	-	6
Cobre (Cu) Total	4688 mg/kg	2377 mg/kg
Mangânes (Mn) Total	225 mg/kg	114 mg/kg
Zinco (Zn) Total	795 mg/kg	403 mg/kg
Ferro (Fe) Total	51963 mg/kg	26345 mg/kg
Boro (B) Total	4 mg/kg	2 mg/kg
Sódio (Na) Total	387 mg/kg	196 mg/kg

Métodos: pH em CaCl2 0,01 M determinação potenciometria; Densidade (m/v); Umidade 60-65º, Umidade 110º e Umidade total determinação por umidade; Carbono Orgânico (CO) oxidação dicromato seguido de titulação;Nitrogênio total digestão sulfúrica (Kjeldahl); Fósforo (P2O5) determinação por espectrofotômetro pelo método com a solução de vanadomolibdica; Potássio (K2O) e Sódio (Na) fotometria de chama; Enxofre (S) gravimétrico de sulfato de bário; Cálcio (Ca), Magnésio (Mg), Cobre (Cu), Manganês (Mn), Zinco (ZN), Ferro (Fe) extração com HCl por espectrofotômetro de absorção atômica; Boro (B) espectrofotometria da azometina-H; Relação C/N cálculo (Ref.: BRASIL, 2017. Manual de Métodos Oficiais para Fertilizantes Minerais, Orgânicos e Corretivos. MAPA). Matéria Orgânica Total, Resíduo Mineral Insolúvel (RMI), Resíduo Mineral (RM) e Resíduo Mineral Total (RMT) por combustão em Mufla (Ref.: ALCARDE, José Carlos. Manual de Análise de Fertilizantes - Piracicaba: FEALQ, 2009.)

SIGNATÁRIO AUTORIZADO

Luís Piló
Rua Conselheiro Barbosa , 35 Apto. 301
Santa Tereza
31.010-510 - Belo Horizonte - MG

Número: 0016546.1-N - O.S.: 9819
Data de Finalização: 18/12/2019

Proprietário:	Propriedade:	Material:
IABS - Instituto Brasileiro de Desenvolvim	UFPE / Carajás	Mat. Org. sólido

IDENTIFICAÇÃO DA AMOSTRA

Nº Amostra: LFC-002193/2019
Identificação: S11D121-SUP

DETERMINAÇÕES	RESULTADOS	
	BASE SECA (65 °C)	BASE ÚMIDA
pH (CaCl2 0,01 M)	-	5,3
Densidade (Residuo Organico)	-	0,67 g/cm3
Umidade (Residuo Orgânico) 60 - 65º C	-	17,02 %
Umidade (Residuo Orgânico) 110°C	-	6,29 %
Umidade Total	-	23,31 %
Materia Orgânica Total (Combustão)	70,32 %	58,35 %
Carbono Orgânico	37,58 %	31,18 %
Resíduo Mineral Total (R.M.T.)	22,09 %	18,33 %
Resíduo Mineral (R.M.)	21,57 %	17,90 %
Resíduo Mineral Insolúvel (R.M.I.)	0,52 %	0,43 %
Nitrogênio Total	4,21 %	3,49 %
Fosforo (P2O5) Total	4,93 %	4,09 %
Potassio (K2O) Total	2,52 %	2,09 %
Calcio (Ca) Total	2,06 %	1,71 %
Magnésio (Mg) Total	0,99 %	0,82 %
Enxofre (S) Total	1,37 %	1,14 %
Relação C/N	-	9
Cobre (Cu) Total	158 mg/kg	131 mg/kg
Mangânes (Mn) Total	592 mg/kg	491 mg/kg
Zinco (Zn) Total	481 mg/kg	399 mg/kg
Ferro (Fe) Total	28254 mg/kg	23445 mg/kg
Boro (B) Total	55 mg/kg	46 mg/kg
Sódio (Na) Total	986 mg/kg	818 mg/kg

Métodos: pH em CaCl2 0,01 M determinação potenciometria; Densidade (m/v); Umidade 60-65º, Umidade 110º e Umidade total determinação por umidade; Carbono Orgânico (CO) oxidação dicromato seguido de titulação;Nitrogênio total digestão sulfúrica (Kjeldahl); Fósforo (P2O5) determinação por espectrofotômetro pelo método com a solução de vanadomolibdica; Potássio (K2O) e Sódio (Na) fotometria de chama; Enxofre (S) gravimétrico de sulfato de bário; Cálcio (Ca), Magnésio (Mg), Cobre (Cu), Manganês (Mn), Zinco (ZN), Ferro (Fe) extração com HCl por espectrofotômetro de absorção atômica; Boro (B) espectrofotometria da azometina-H; Relação C/N cálculo (Ref.: BRASIL, 2017. Manual de Métodos Oficiais para Fertilizantes Minerais, Orgânicos e Corretivos. MAPA). Matéria Orgânica Total, Resíduo Mineral Insolúvel (RMI), Resíduo Mineral (RM) e Resíduo Mineral Total (RMT) por combustão em Mufla (Ref.: ALCARDE, José Carlos. Manual de Análise de Fertilizantes - Piracicaba: FEALQ, 2009.)

SIGNATÁRIO AUTORIZADO

Luís Piló
Rua Conselheiro Barbosa , 35 Apto. 301
Santa Tereza
31.010-510 - Belo Horizonte - MG

Número: 0016546.1-N - O.S.: 9819
Data de Finalização: 18/12/2019

**Proprietário:**	**Propriedade:**	**Material:**
IABS - Instituto Brasileiro de Desenvolvim	UFPE / Carajás	Mat. Org. sólido

IDENTIFICAÇÃO DA AMOSTRA

Nº Amostra: LFC-002194/2019
Identificação: S11B168-SUP

DETERMINAÇÕES	RESULTADOS	
	BASE SECA (65 °C)	BASE ÚMIDA
pH (CaCl2 0,01 M)	-	3,1
Densidade (Residuo Organico)	-	0,76 g/cm3
Umidade (Residuo Orgânico) 60 - 65º C	-	52,35 %
Umidade (Residuo Orgânico) 110°C	-	0,61 %
Umidade Total	-	52,96 %
Materia Orgânica Total (Combustão)	50,85 %	24,23 %
Carbono Orgânico	25,62 %	12,21 %
Resíduo Mineral Total (R.M.T.)	47,87 %	22,81 %
Resíduo Mineral (R.M.)	46,32 %	22,07 %
Resíduo Mineral Insolúvel (R.M.I.)	1,55 %	0,74 %
Nitrogênio Total	5,52 %	2,63 %
Fosforo (P2O5) Total	19,06 %	9,08 %
Potassio (K2O) Total	2,29 %	1,09 %
Calcio (Ca) Total	0,84 %	0,40 %
Magnésio (Mg) Total	0,19 %	0,09 %
Enxofre (S) Total	0,08 %	0,04 %
Relação C/N	-	5
Cobre (Cu) Total	3079 mg/kg	1467 mg/kg
Mangânes (Mn) Total	258 mg/kg	123 mg/kg
Zinco (Zn) Total	1414 mg/kg	674 mg/kg
Ferro (Fe) Total	124407 mg/kg	59280 mg/kg
Boro (B) Total	4 mg/kg	2 mg/kg
Sódio (Na) Total	1391 mg/kg	663 mg/kg

Métodos: pH em CaCl2 0,01 M determinação potenciometria; Densidade (m/v); Umidade 60-65º, Umidade 110º e Umidade total determinação por umidade; Carbono Orgânico (CO) oxidação dicromato seguido de titulação;Nitrogênio total digestão sulfúrica (Kjeldahl); Fósforo (P2O5) determinação por espectrofotômetro pelo método com a solução de vanadomolibdica; Potássio (K2O) e Sódio (Na) fotometria de chama; Enxofre (S) gravimétrico de sulfato de bário; Cálcio (Ca), Magnésio (Mg), Cobre (Cu), Manganês (Mn), Zinco (ZN), Ferro (Fe) extração com HCl por espectrofotômetro de absorção atômica; Boro (B) espectrofotometria da azometina-H; Relação C/N cálculo (Ref.: BRASIL, 2017. Manual de Métodos Oficiais para Fertilizantes Minerais, Orgânicos e Corretivos. MAPA). Matéria Orgânica Total, Resíduo Mineral Insolúvel (RMI), Resíduo Mineral (RM) e Resíduo Mineral Total (RMT) por combustão em Mufla (Ref.: ALCARDE, José Carlos. Manual de Análise de Fertilizantes - Piracicaba: FEALQ, 2009.)

SIGNATÁRIO AUTORIZADO

Luís Piló
Rua Conselheiro Barbosa , 35 Apto. 301
Santa Tereza
31.010-510 - Belo Horizonte - MG

Número: 0016546.1-N - O.S.: 9819
Data de Finalização: 18/12/2019

Proprietário:	Propriedade:	Material:
IABS - Instituto Brasileiro de Desenvolvim	UFPE / Carajás	Mat. Org. sólido

IDENTIFICAÇÃO DA AMOSTRA

Nº Amostra: LFC-002195/2019
Identificação: N4WS72-SUP

DETERMINAÇÕES	RESULTADOS	
	BASE SECA (65 °C)	BASE ÚMIDA
pH (CaCl2 0,01 M)	-	4,0
Densidade (Residuo Organico)	-	0,63 g/cm3
Umidade (Residuo Orgânico) 60 - 65º C	-	44,96 %
Umidade (Residuo Orgânico) 110°C	-	3,56 %
Umidade Total	-	48,52 %
Materia Orgânica Total (Combustão)	75,55 %	41,58 %
Carbono Orgânico	39,86 %	21,94 %
Resíduo Mineral Total (R.M.T.)	17,99 %	9,90 %
Resíduo Mineral (R.M.)	17,31 %	9,53 %
Resíduo Mineral Insolúvel (R.M.I.)	0,67 %	0,37 %
Nitrogênio Total	6,69 %	3,68 %
Fosforo (P2O5) Total	7,21 %	3,97 %
Potassio (K2O) Total	0,35 %	0,19 %
Calcio (Ca) Total	1,40 %	0,77 %
Magnésio (Mg) Total	2,03 %	1,12 %
Enxofre (S) Total	0,05 %	0,03 %
Relação C/N	-	6
Cobre (Cu) Total	521 mg/kg	287 mg/kg
Mangânes (Mn) Total	798 mg/kg	439 mg/kg
Zinco (Zn) Total	6790 mg/kg	3737 mg/kg
Ferro (Fe) Total	18574 mg/kg	10223 mg/kg
Boro (B) Total	20 mg/kg	11 mg/kg
Sódio (Na) Total	776 mg/kg	427 mg/kg

Métodos: pH em CaCl2 0,01 M determinação potenciometria; Densidade (m/v); Umidade 60-65º, Umidade 110º e Umidade total determinação por umidade; Carbono Orgânico (CO) oxidação dicromato seguido de titulação;Nitrogênio total digestão sulfúrica (Kjeldahl); Fósforo (P2O5) determinação por espectrofotômetro pelo método com a solução de vanadomolibdica; Potássio (K2O) e Sódio (Na) fotometria de chama; Enxofre (S) gravimétrico de sulfato de bário; Cálcio (Ca), Magnésio (Mg), Cobre (Cu), Manganês (Mn), Zinco (ZN), Ferro (Fe) extração com HCl por espectrofotômetro de absorção atômica; Boro (B) espectrofotometria da azometina-H; Relação C/N cálculo (Ref.: BRASIL, 2017. Manual de Métodos Oficiais para Fertilizantes Minerais, Orgânicos e Corretivos. MAPA). Matéria Orgânica Total, Resíduo Mineral Insolúvel (RMI), Resíduo Mineral (RM) e Resíduo Mineral Total (RMT) por combustão em Mufla (Ref.: ALCARDE, José Carlos. Manual de Análise de Fertilizantes - Piracicaba: FEALQ, 2009.)

SIGNATÁRIO AUTORIZADO

Luís Piló
Rua Conselheiro Barbosa , 35 Apto. 301
Santa Tereza
31.010-510 - Belo Horizonte - MG

Número: 0016546.1-N - O.S.: 9819
Data de Finalização: 18/12/2019

**Proprietário:**	**Propriedade:**	**Material:**
IABS - Instituto Brasileiro de Desenvolvim	UFPE / Carajás	Mat. Org. sólido

IDENTIFICAÇÃO DA AMOSTRA

Nº Amostra: LFC-002196/2019
Identificação: M219-SUP

DETERMINAÇÕES	RESULTADOS	
	BASE SECA (65 °C)	BASE ÚMIDA
pH (CaCl2 0,01 M)	-	2,4
Densidade (Residuo Organico)	-	0,71 g/cm3
Umidade (Residuo Orgânico) 60 - 65º C	-	62,88 %
Umidade (Residuo Orgânico) 110°C	-	1,62 %
Umidade Total	-	64,50 %
Materia Orgânica Total (Combustão)	74,60 %	27,69 %
Carbono Orgânico	38,20 %	14,18 %
Resíduo Mineral Total (R.M.T.)	21,04 %	7,81 %
Resíduo Mineral (R.M.)	16,70 %	6,20 %
Resíduo Mineral Insolúvel (R.M.I.)	4,34 %	1,61 %
Nitrogênio Total	7,79 %	2,89 %
Fosforo (P2O5) Total	4,53 %	1,68 %
Potassio (K2O) Total	0,16 %	0,06 %
Calcio (Ca) Total	0,62 %	0,23 %
Magnésio (Mg) Total	0,11 %	0,04 %
Enxofre (S) Total	0,05 %	0,02 %
Relação C/N	-	5
Cobre (Cu) Total	1215 mg/kg	451 mg/kg
Mangânes (Mn) Total	113 mg/kg	42 mg/kg
Zinco (Zn) Total	364 mg/kg	135 mg/kg
Ferro (Fe) Total	78596 mg/kg	29175 mg/kg
Boro (B) Total	3 mg/kg	1 mg/kg
Sódio (Na) Total	364 mg/kg	135 mg/kg

Métodos: pH em CaCl2 0,01 M determinação potenciometria; Densidade (m/v); Umidade 60-65º, Umidade 110º e Umidade total determinação por umidade; Carbono Orgânico (CO) oxidação dicromato seguido de titulação;Nitrogênio total digestão sulfúrica (Kjeldahl); Fósforo (P2O5) determinação por espectrofotômetro pelo método com a solução de vanadomolibdica; Potássio (K2O) e Sódio (Na) fotometria de chama; Enxofre (S) gravimétrico de sulfato de bário; Cálcio (Ca), Magnésio (Mg), Cobre (Cu), Manganês (Mn), Zinco (ZN), Ferro (Fe) extração com HCl por espectrofotômetro de absorção atômica; Boro (B) espectrofotometria da azometina-H; Relação C/N cálculo (Ref.: BRASIL, 2017. Manual de Métodos Oficiais para Fertilizantes Minerais, Orgânicos e Corretivos. MAPA). Matéria Orgânica Total, Resíduo Mineral Insolúvel (RMI), Resíduo Mineral (RM) e Resíduo Mineral Total (RMT) por combustão em Mufla (Ref.: ALCARDE, José Carlos. Manual de Análise de Fertilizantes - Piracicaba: FEALQ, 2009.)

SIGNATÁRIO AUTORIZADO

ESALQ

Luís Piló
Rua Conselheiro Barbosa , 35 Apto. 301
Santa Tereza
31.010-510 - Belo Horizonte - MG

Número: 0016546.1-N - O.S.: 9819
Data de Finalização: 18/12/2019

Proprietário:	Propriedade:	Material:
IABS - Instituto Brasileiro de Desenvolvim	UFPE / Carajás	Mat. Org. sólido

IDENTIFICAÇÃO DA AMOSTRA

Nº Amostra: LFC-002197/2019
Identificação: S11A36-MVF

DETERMINAÇÕES	RESULTADOS	
	BASE SECA (65 °C)	BASE ÚMIDA
pH (CaCl2 0,01 M)	-	3,2
Densidade (Residuo Organico)	-	0,93 g/cm3
Umidade (Residuo Orgânico) 60 - 65º C	-	6,12 %
Umidade (Residuo Orgânico) 110°C	-	0,72 %
Umidade Total	-	6,84 %
Materia Orgânica Total (Combustão)	31,16 %	29,25 %
Carbono Orgânico	15,89 %	14,92 %
Resíduo Mineral Total (R.M.T.)	68,07 %	63,90 %
Resíduo Mineral (R.M.)	63,26 %	59,39 %
Resíduo Mineral Insolúvel (R.M.I.)	4,80 %	4,51 %
Nitrogênio Total	1,20 %	1,13 %
Fosforo (P2O5) Total	6,77 %	6,36 %
Potassio (K2O) Total	0,02 %	0,02 %
Calcio (Ca) Total	0,60 %	0,56 %
Magnésio (Mg) Total	0,04 %	0,04 %
Enxofre (S) Total	0,05 %	0,05 %
Relação C/N	-	13
Cobre (Cu) Total	72 mg/kg	68 mg/kg
Mangânes (Mn) Total	125 mg/kg	117 mg/kg
Zinco (Zn) Total	55 mg/kg	52 mg/kg
Ferro (Fe) Total	38531 mg/kg	36173 mg/kg
Boro (B) Total	3 mg/kg	3 mg/kg
Sódio (Na) Total	575 mg/kg	540 mg/kg

Métodos: pH em CaCl2 0,01 M determinação potenciometria; Densidade (m/v); Umidade 60-65º, Umidade 110º e Umidade total determinação por umidade; Carbono Orgânico (CO) oxidação dicromato seguido de titulação;Nitrogênio total digestão sulfúrica (Kjeldahl); Fósforo (P2O5) determinação por espectrofotômetro pelo método com a solução de vanadomolibdica; Potássio (K2O) e Sódio (Na) fotometria de chama; Enxofre (S) gravimétrico de sulfato de bário; Cálcio (Ca), Magnésio (Mg), Cobre (Cu), Manganês (Mn), Zinco (ZN), Ferro (Fe) extração com HCl por espectrofotômetro de absorção atômica; Boro (B) espectrofotometria da azometina-H; Relação C/N cálculo (Ref.: BRASIL, 2017. Manual de Métodos Oficiais para Fertilizantes Minerais, Orgânicos e Corretivos. MAPA). Matéria Orgânica Total, Resíduo Mineral Insolúvel (RMI), Resíduo Mineral (RM) e Resíduo Mineral Total (RMT) por combustão em Mufla (Ref.: ALCARDE, José Carlos. Manual de Análise de Fertilizantes - Piracicaba: FEALQ, 2009.)

SIGNATÁRIO AUTORIZADO

Luís Piló
Rua Conselheiro Barbosa , 35 Apto. 301
Santa Terezinha
31010-510 - Belo Horizonte - MG

Número: 0014532.1-N - O.S.: 8565
Data de Finalização: 09/04/2019

Proprietário:	Propriedade:	Material:
IABS - Instituto Brasileiro de Desenvolvim	(não informado)	Mat. Org. sólido

IDENTIFICAÇÃO DA AMOSTRA

Nº Amostra: LFC-000390/2019
Identificação: S11D-83-SUP

DETERMINAÇÕES	RESULTADOS	
	BASE SECA (65 °C)	BASE ÚMIDA
pH (CaCl2 0,01 M)	-	5,6
Densidade (Residuo Organico)	-	0,56 g/cm3
Umidade (Residuo Orgânico) 60 - 65º C	-	43,96 %
Umidade (Residuo Orgânico) 110°C	-	3,72 %
Umidade Total	-	47,68 %
Materia Orgânica Total (Combustão)	80,50 %	45,11 %
Carbono Orgânico	42,18 %	23,64 %
Resíduo Mineral Total (R.M.T.)	12,87 %	7,21 %
Resíduo Mineral (R.M.)	12,24 %	6,86 %
Resíduo Mineral Insolúvel (R.M.I.)	0,62 %	0,35 %
Nitrogênio Total	13,22 %	7,41 %
Fosforo (P2O5) Total	5,60 %	3,14 %
Potassio (K2O) Total	2,82 %	1,58 %
Calcio (Ca) Total	1,20 %	0,67 %
Magnésio (Mg) Total	0,68 %	0,38 %
Enxofre (S) Total	0,25 %	0,14 %
Relação C/N	-	3
Cobre (Cu) Total	202 mg/kg	113 mg/kg
Mangânes (Mn) Total	202 mg/kg	113 mg/kg
Zinco (Zn) Total	616 mg/kg	345 mg/kg
Ferro (Fe) Total	4363 mg/kg	2445 mg/kg
Boro (B) Total	5 mg/kg	3 mg/kg
Sódio (Na) Total	8242 mg/kg	4619 mg/kg

Métodos: pH em CaCl2 0,01 M determinação potenciometria; Densidade (m/v); Umidade 60-65º, Umidade 110º e Umidade total determinação por umidade; Carbono Orgânico (CO) oxidação dicromato seguido de titulação;Nitrogênio total digestão sulfúrica (Kjeldahl); Fósforo (P2O5) determinação por espectrofotômetro pelo método com a solução de vanadomolibdica; Potássio (K2O) e Sódio (Na) fotometria de chama; Enxofre (S) gravimétrico de sulfato de bário; Cálcio (Ca), Magnésio (Mg), Cobre (Cu), Manganês (Mn), Zinco (ZN), Ferro (Fe) extração com HCl por espectrofotômetro de absorção atômica; Boro (B) espectrofotometria da azometina-H; Relação C/N cálculo (Ref.: BRASIL, 2017. Manual de Métodos Oficiais para Fertilizantes Minerais, Orgânicos e Corretivos. MAPA). Matéria Orgânica Total, Resíduo Mineral Insolúvel (RMI), Resíduo Mineral (RM) e Resíduo Mineral Total (RMT) por combustão em Mufla (Ref.: ALCARDE, José Carlos. Manual de Análise de Fertilizantes - Piracicaba: FEALQ, 2009.)

SIGNATÁRIO AUTORIZADO

Luís Piló
Rua Conselheiro Barbosa , 35 Apto. 301
Santa Terezinha
31010-510 - Belo Horizonte - MG

Número: 0014532.1-N - O.S.: 8565
Data de Finalização: 09/04/2019

Proprietário:	Propriedade:	Material:
IABS - Instituto Brasileiro de Desenvolvim	(não informado)	Mat. Org. sólido

IDENTIFICAÇÃO DA AMOSTRA

Nº Amostra: LFC-000391/2019
Identificação: S11C-41-SUP

DETERMINAÇÕES	RESULTADOS	
	BASE SECA (65 °C)	BASE ÚMIDA
pH (CaCl2 0,01 M)	-	4,5
Densidade (Residuo Organico)	-	0,70 g/cm3
Umidade (Residuo Orgânico) 60 - 65º C	-	56,89 %
Umidade (Residuo Orgânico) 110°C	-	2,98 %
Umidade Total	-	59,87 %
Materia Orgânica Total (Combustão)	80,96 %	34,90 %
Carbono Orgânico	40,29 %	17,37 %
Resíduo Mineral Total (R.M.T.)	12,13 %	5,23 %
Resíduo Mineral (R.M.)	11,41 %	4,92 %
Resíduo Mineral Insolúvel (R.M.I.)	0,72 %	0,31 %
Nitrogênio Total	11,69 %	5,04 %
Fosforo (P2O5) Total	4,38 %	1,89 %
Potassio (K2O) Total	2,34 %	1,01 %
Calcio (Ca) Total	1,30 %	0,56 %
Magnésio (Mg) Total	0,53 %	0,23 %
Enxofre (S) Total	0,26 %	0,11 %
Relação C/N	-	3
Cobre (Cu) Total	1294 mg/kg	558 mg/kg
Mangânes (Mn) Total	292 mg/kg	126 mg/kg
Zinco (Zn) Total	3023 mg/kg	1303 mg/kg
Ferro (Fe) Total	4414 mg/kg	1903 mg/kg
Boro (B) Total	7 mg/kg	3 mg/kg
Sódio (Na) Total	1751 mg/kg	755 mg/kg

Métodos: pH em CaCl2 0,01 M determinação potenciometria; Densidade (m/v); Umidade 60-65º, Umidade 110º e Umidade total determinação por umidade; Carbono Orgânico (CO) oxidação dicromato seguido de titulação;Nitrogênio total digestão sulfúrica (Kjeldahl); Fósforo (P2O5) determinação por espectrofotômetro pelo método com a solução de vanadomolibdica; Potássio (K2O) e Sódio (Na) fotometria de chama; Enxofre (S) gravimétrico de sulfato de bário; Cálcio (Ca), Magnésio (Mg), Cobre (Cu), Manganês (Mn), Zinco (ZN), Ferro (Fe) extração com HCl por espectrofotômetro de absorção atômica; Boro (B) espectrofotometria da azometina-H; Relação C/N cálculo (Ref.: BRASIL, 2017. Manual de Métodos Oficiais para Fertilizantes Minerais, Orgânicos e Corretivos. MAPA). Matéria Orgânica Total, Resíduo Mineral Insolúvel (RMI), Resíduo Mineral (RM) e Resíduo Mineral Total (RMT) por combustão em Mufla (Ref.: ALCARDE, José Carlos. Manual de Análise de Fertilizantes - Piracicaba: FEALQ, 2009.)

SIGNATÁRIO AUTORIZADO

Luís Piló
Rua Conselheiro Barbosa , 35 Apto. 301
Santa Terezinha
31010-510 - Belo Horizonte - MG

Número: 0014532.1-N - O.S.: 8565
Data de Finalização: 09/04/2019

Proprietário:	Propriedade:	Material:
IABS - Instituto Brasileiro de Desenvolvim	(não informado)	Mat. Org. sólido

IDENTIFICAÇÃO DA AMOSTRA

Nº Amostra: LFC-000392/2019
Identificação: S11C-41-30

DETERMINAÇÕES	RESULTADOS	
	BASE SECA (65 °C)	BASE ÚMIDA
pH (CaCl2 0,01 M)	-	4,5
Densidade (Residuo Organico)	-	0,69 g/cm3
Umidade (Residuo Orgânico) 60 - 65º C	-	48,60 %
Umidade (Residuo Orgânico) 110°C	-	3,26 %
Umidade Total	-	51,86 %
Materia Orgânica Total (Combustão)	73,31 %	37,68 %
Carbono Orgânico	37,47 %	19,26 %
Resíduo Mineral Total (R.M.T.)	20,35 %	10,46 %
Resíduo Mineral (R.M.)	18,02 %	9,26 %
Resíduo Mineral Insolúvel (R.M.I.)	2,33 %	1,20 %
Nitrogênio Total	10,93 %	5,62 %
Fosforo (P2O5) Total	5,04 %	2,59 %
Potassio (K2O) Total	2,04 %	1,05 %
Calcio (Ca) Total	1,44 %	0,74 %
Magnésio (Mg) Total	0,47 %	0,24 %
Enxofre (S) Total	0,33 %	0,17 %
Relação C/N	-	3
Cobre (Cu) Total	1405 mg/kg	722 mg/kg
Mangânes (Mn) Total	586 mg/kg	301 mg/kg
Zinco (Zn) Total	2807 mg/kg	1443 mg/kg
Ferro (Fe) Total	45844 mg/kg	23564 mg/kg
Boro (B) Total	6 mg/kg	3 mg/kg
Sódio (Na) Total	1405 mg/kg	722 mg/kg

Métodos: pH em CaCl2 0,01 M determinação potenciometria; Densidade (m/v); Umidade 60-65º, Umidade 110º e Umidade total determinação por umidade; Carbono Orgânico (CO) oxidação dicromato seguido de titulação;Nitrogênio total digestão sulfúrica (Kjeldahl); Fósforo (P2O5) determinação por espectrofotômetro pelo método com a solução de vanadomolibdica; Potássio (K2O) e Sódio (Na) fotometria de chama; Enxofre (S) gravimétrico de sulfato de bário; Cálcio (Ca), Magnésio (Mg), Cobre (Cu), Manganês (Mn), Zinco (ZN), Ferro (Fe) extração com HCl por espectrofotômetro de absorção atômica; Boro (B) espectrofotometria da azometina-H; Relação C/N cálculo (Ref.: BRASIL, 2017. Manual de Métodos Oficiais para Fertilizantes Minerais, Orgânicos e Corretivos. MAPA). Matéria Orgânica Total, Resíduo Mineral Insolúvel (RMI), Resíduo Mineral (RM) e Resíduo Mineral Total (RMT) por combustão em Mufla (Ref.: ALCARDE, José Carlos. Manual de Análise de Fertilizantes - Piracicaba: FEALQ, 2009.)

SIGNATÁRIO AUTORIZADO

Luís Piló
Rua Conselheiro Barbosa , 35 Apto. 301
Santa Terezinha
31010-510 - Belo Horizonte - MG

Número: 0014532.1-N - O.S.: 8565
Data de Finalização: 09/04/2019

Proprietário:	Propriedade:	Material:
IABS - Instituto Brasileiro de Desenvolvim	(não informado)	Mat. Org. sólido

IDENTIFICAÇÃO DA AMOSTRA

Nº Amostra: LFC-000393/2019
Identificação: S11A-36-BG

DETERMINAÇÕES	RESULTADOS	
	BASE SECA (65 °C)	BASE ÚMIDA
pH (CaCl2 0,01 M)	-	5,2
Densidade (Residuo Organico)	-	0,67 g/cm3
Umidade (Residuo Orgânico) 60 - 65º C	-	41,48 %
Umidade (Residuo Orgânico) 110°C	-	3,53 %
Umidade Total	-	45,01 %
Materia Orgânica Total (Combustão)	75,36 %	44,10 %
Carbono Orgânico	38,57 %	22,57 %
Resíduo Mineral Total (R.M.T.)	18,59 %	10,88 %
Resíduo Mineral (R.M.)	17,58 %	10,29 %
Resíduo Mineral Insolúvel (R.M.I.)	1,01 %	0,59 %
Nitrogênio Total	8,99 %	5,26 %
Fosforo (P2O5) Total	7,11 %	4,16 %
Potassio (K2O) Total	4,34 %	2,54 %
Calcio (Ca) Total	0,77 %	0,45 %
Magnésio (Mg) Total	0,39 %	0,23 %
Enxofre (S) Total	0,41 %	0,24 %
Relação C/N	-	4
Cobre (Cu) Total	924 mg/kg	541 mg/kg
Mangânes (Mn) Total	222 mg/kg	130 mg/kg
Zinco (Zn) Total	1558 mg/kg	912 mg/kg
Ferro (Fe) Total	18360 mg/kg	10744 mg/kg
Boro (B) Total	10 mg/kg	6 mg/kg
Sódio (Na) Total	8766 mg/kg	5130 mg/kg

Métodos: pH em CaCl2 0,01 M determinação potenciometria; Densidade (m/v); Umidade 60-65º, Umidade 110º e Umidade total determinação por umidade; Carbono Orgânico (CO) oxidação dicromato seguido de titulação;Nitrogênio total digestão sulfúrica (Kjeldahl); Fósforo (P2O5) determinação por espectrofotômetro pelo método com a solução de vanadomolibdica; Potássio (K2O) e Sódio (Na) fotometria de chama; Enxofre (S) gravimétrico de sulfato de bário; Cálcio (Ca), Magnésio (Mg), Cobre (Cu), Manganês (Mn), Zinco (ZN), Ferro (Fe) extração com HCl por espectrofotômetro de absorção atômica; Boro (B) espectrofotometria da azometina-H; Relação C/N cálculo (Ref.: BRASIL, 2017. Manual de Métodos Oficiais para Fertilizantes Minerais, Orgânicos e Corretivos. MAPA). Matéria Orgânica Total, Resíduo Mineral Insolúvel (RMI), Resíduo Mineral (RM) e Resíduo Mineral Total (RMT) por combustão em Mufla (Ref.: ALCARDE, José Carlos. Manual de Análise de Fertilizantes - Piracicaba: FEALQ, 2009.)

SIGNATÁRIO AUTORIZADO

Luís Piló
Rua Conselheiro Barbosa , 35 Apto. 301
Santa Terezinha
31010-510 - Belo Horizonte - MG

Número: 0014532.1-N - O.S.: 8565
Data de Finalização: 09/04/2019

Proprietário:	Propriedade:	Material:
IABS - Instituto Brasileiro de Desenvolvim	(não informado)	Mat. Org. sólido

IDENTIFICAÇÃO DA AMOSTRA

Nº Amostra: LFC-000394/2019
Identificação: S11A-36-VERT

DETERMINAÇÕES	RESULTADOS	
	BASE SECA (65 °C)	BASE ÚMIDA
pH (CaCl2 0,01 M)	-	2,7
Densidade (Residuo Organico)	-	0,39 g/cm3
Umidade (Residuo Orgânico) 60 - 65º C	-	46,55 %
Umidade (Residuo Orgânico) 110°C	-	2,87 %
Umidade Total	-	49,42 %
Materia Orgânica Total (Combustão)	75,77 %	40,50 %
Carbono Orgânico	39,46 %	21,09 %
Resíduo Mineral Total (R.M.T.)	18,86 %	10,08 %
Resíduo Mineral (R.M.)	17,72 %	9,47 %
Resíduo Mineral Insolúvel (R.M.I.)	1,14 %	0,61 %
Nitrogênio Total	5,86 %	3,13 %
Fosforo (P2O5) Total	2,17 %	1,16 %
Potassio (K2O) Total	0,13 %	0,07 %
Calcio (Ca) Total	0,13 %	0,07 %
Magnésio (Mg) Total	0,04 %	0,02 %
Enxofre (S) Total	0,13 %	0,07 %
Relação C/N	-	7
Cobre (Cu) Total	385 mg/kg	206 mg/kg
Mangânes (Mn) Total	314 mg/kg	168 mg/kg
Zinco (Zn) Total	30 mg/kg	16 mg/kg
Ferro (Fe) Total	78913 mg/kg	42179 mg/kg
Boro (B) Total	2 mg/kg	1 mg/kg
Sódio (Na) Total	413 mg/kg	221 mg/kg

Métodos: pH em CaCl2 0,01 M determinação potenciometria; Densidade (m/v); Umidade 60-65º, Umidade 110º e Umidade total determinação por umidade; Carbono Orgânico (CO) oxidação dicromato seguido de titulação;Nitrogênio total digestão sulfúrica (Kjeldahl); Fósforo (P2O5) determinação por espectrofotômetro pelo método com a solução de vanadomolibdica; Potássio (K2O) e Sódio (Na) fotometria de chama; Enxofre (S) gravimétrico de sulfato de bário; Cálcio (Ca), Magnésio (Mg), Cobre (Cu), Manganês (Mn), Zinco (ZN), Ferro (Fe) extração com HCl por espectrofotômetro de absorção atômica; Boro (B) espectrofotometria da azometina-H; Relação C/N cálculo (Ref.: BRASIL, 2017. Manual de Métodos Oficiais para Fertilizantes Minerais, Orgânicos e Corretivos. MAPA). Matéria Orgânica Total, Resíduo Mineral Insolúvel (RMI), Resíduo Mineral (RM) e Resíduo Mineral Total (RMT) por combustão em Mufla (Ref.: ALCARDE, José Carlos. Manual de Análise de Fertilizantes - Piracicaba: FEALQ, 2009.)

SIGNATÁRIO AUTORIZADO

ESALQ
