# Supplementary material for: Bats as ecosystem engineers in iron ore caves in the Carajás National Forest, Brazilian Amazonia
Source: PLoS One. 2023 May 11;18(5):e0267870. doi: 10.1371/journal.pone.0267870 (PMC10174506; doi:10.1371/journal.pone.0267870)

Escola Politécnica da Universidade de São Paulo
Departamento de Engenharia de Minas e de Petróleo
Laboratório de Caracterização Tecnológica
Av. Prof. Mello Moraes, 2373 CEP 05508-030 São Paulo - SP www.lct.poli.usp.br
Tel: 11 3091-5151 e-mail: lct@lct.poli.usp.br

RESULTADO DE IDENTIFICAÇÃO DE FASES POR DIFRATOMETRIA DE RAIOS X

RELATÓRIO: DRX 610/19 **DATA:** 12/04/19

CLIENTE: Luís Piló

AMOSTRA: S11D-83-SUP **IDENT. LCT:** 130-2097.HPF

1. MÉTODO

O estudo foi efetuado através do método do pó, mediante o emprego de difratômetro de raios X com detector sensível a posição.

A identificação das fases cristalinas, abaixo discriminadas, foi obtida por comparação do difratograma da amostra com os bancos de dados PDF2 do ICDD - International Centre for Diffraction Data e ICSD – Inorganic Crystal Structure Database.

2. RESULTADOS

Os resultados obtidos estão listados na tabela abaixo:

ICDD	Mineral/Composto	Fórmula Química	Obs
01-074-2051	Nitrato de amônio e potássio	$(NH_4)_{0,88}K_{0,12}NO_3$	
98-007-9199	Biphosammita	$(NH_4)(H_2PO_4)$	
98-000-6104	Hannayita	$Mg_3(NH_4)_2(HPO_4)_4(H_2O)_8$	
00-011-0117	Syngenita	$K_2Ca(SO_4)_2\ H_2O$	
	Fase amorfa		
01-080-0759	Fosfato de ferro hidratado	$Fe_4(PO_4)_3(OH)_3$	pp

Nota: pp = possível presença

O difratograma obtido (cor vermelha), onde são assinaladas as linhas de difração correspondente(s) à(s) fase(s) identificada(s) (cada fase em uma cor distinta) é apresentado anexo.

Profa. Dra. Carina Ulsen
Coordenadora do LCT

Dra. Maria Manuela Tassinari
Pesquisadora sênior

Dra. Juliana Lívi Antoniassi
Pesquisadora

Verifique a autenticidade deste documento em www.lct.poli.usp.br utilizando o código **BKQA-ZKJA-INTA-OCXB**

Escola Politécnica da Universidade de São Paulo
Departamento de Engenharia de Minas e de Petróleo
Laboratório de Caracterização Tecnológica
Av. Prof. Mello Moraes, 2373 CEP 05508-030 São Paulo - SP www.lct.poli.usp.br
Tel: 11 3091-5151 e-mail: lct@lct.poli.usp.br

DIFRATOGRAMA DE RAIOS X

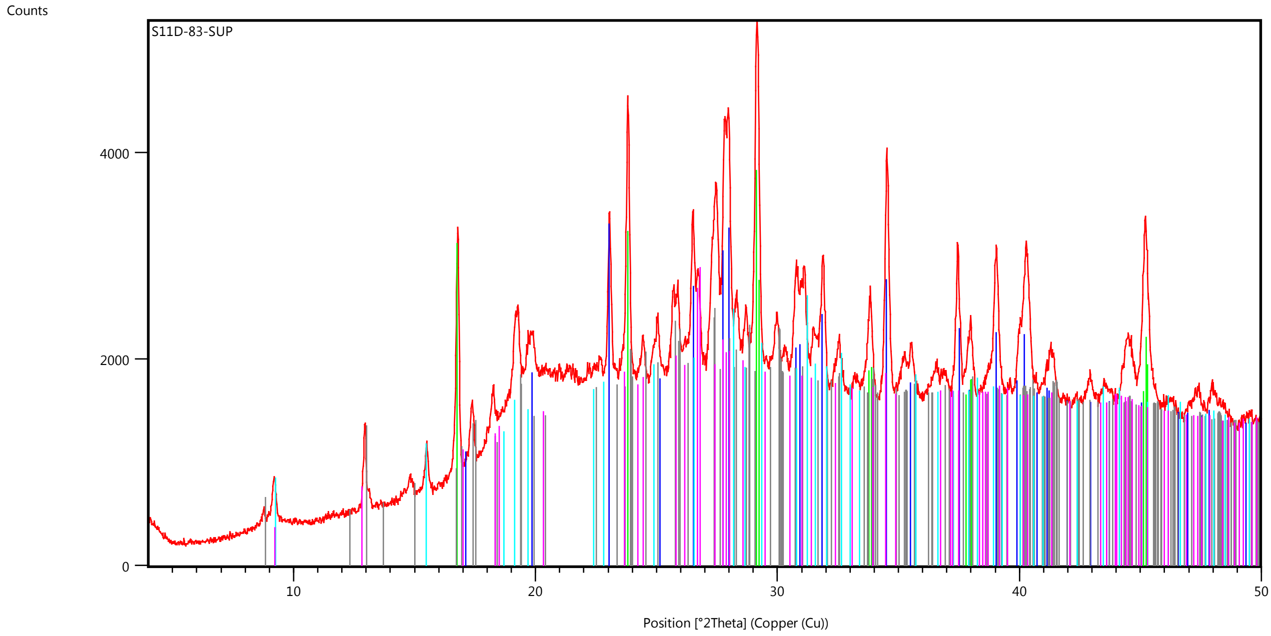

FASES IDENTIFICADAS

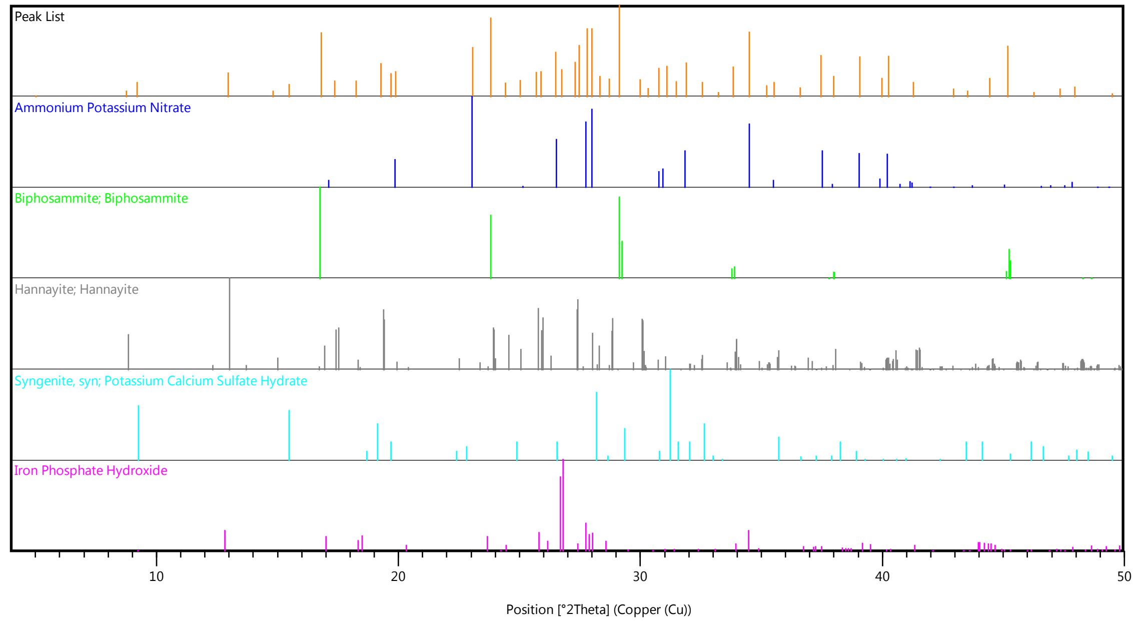

Escola Politécnica da Universidade de São Paulo
Departamento de Engenharia de Minas e de Petróleo
Laboratório de Caracterização Tecnológica
Av. Prof. Mello Moraes, 2373 CEP 05508-030 São Paulo - SP www.lct.poli.usp.br
Tel: 11 3091-5151 e-mail: lct@lct.poli.usp.br

RESULTADO DE IDENTIFICAÇÃO DE FASES POR DIFRATOMETRIA DE RAIOS X

RELATÓRIO: DRX 611/19 **DATA:** 12/04/19

CLIENTE: Luís Piló

AMOSTRA: S11A-36-BG **IDENT. LCT:** 130-2098.HPF

1. MÉTODO

O estudo foi efetuado através do método do pó, mediante o emprego de difratômetro de raios X com detector sensível a posição.

A identificação das fases cristalinas, abaixo discriminadas, foi obtida por comparação do difratograma da amostra com os bancos de dados PDF2 do ICDD - International Centre for Diffraction Data e ICSD – Inorganic Crystal Structure Database.

2. RESULTADOS

Os resultados obtidos estão listados na tabela abaixo:

ICDD	Mineral/Composto	Fórmula Química	Obs
98-007-9199	Biphosammita	$(NH_4)(H_2PO_4)$	
00-020-0928	Aftitalita	$K_3Na(SO_4)_2$	
00-037-0466	Leucofosfita	$KFe_2^{+3}(PO_4)_2(OH)\ 2H_2O$	e/ou
00-041-0593	Spheniscidita	$(NH_4)Fe_2(PO_4)_2(OH)\ 2H_2O$	
01-074-1118	Syngenita	$K_2Ca(SO_4)_2(H_2O)$	
	Fase amorfa		
01-087-2096	Quartzo	SiO_2	pp

Nota: pp = possível presença

O difratograma obtido (cor vermelha), onde são assinaladas as linhas de difração correspondente(s) à(s) fase(s) identificada(s) (cada fase em uma cor distinta) é apresentado anexo.

Profa. Dra. Carina Ulsen
Coordenadora do LCT

Dra. Maria Manuela Tassinari
Pesquisadora sênior

Dra. Juliana Lívi Antoniassi
Pesquisadora

Verifique a autenticidade deste documento em www.lct.poli.usp.br utilizando o código **QKQA-KKJA-ORTA-WJXB**

DIFRATOGRAMA DE RAIOS X

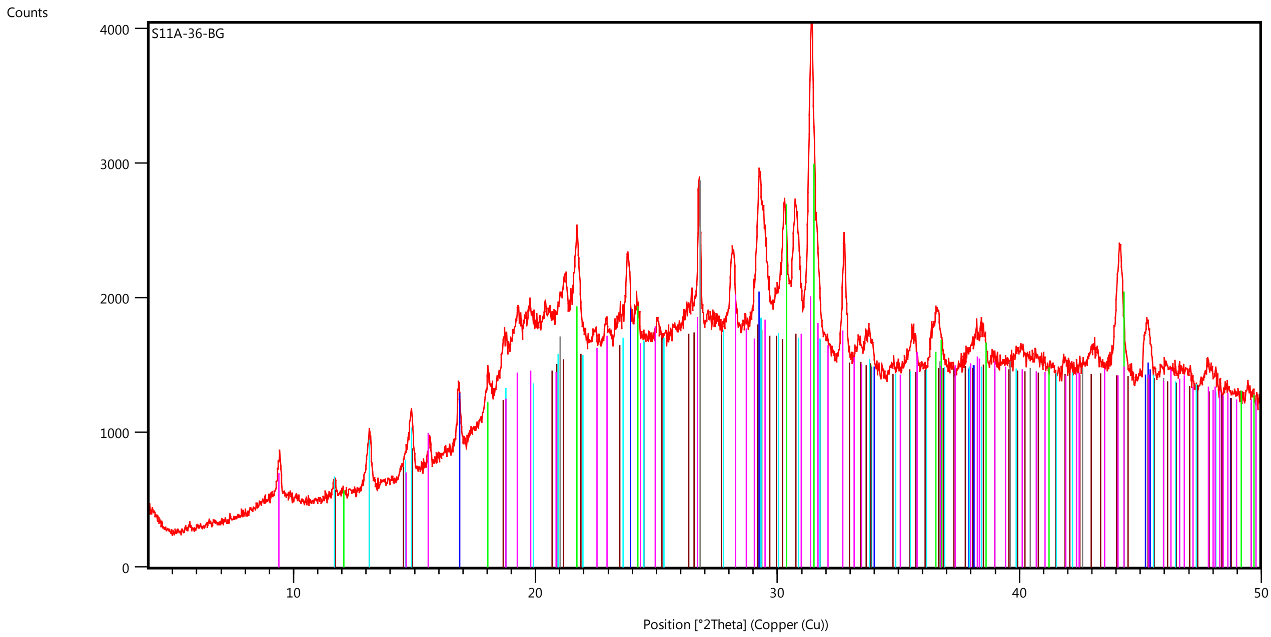

FASES IDENTIFICADAS

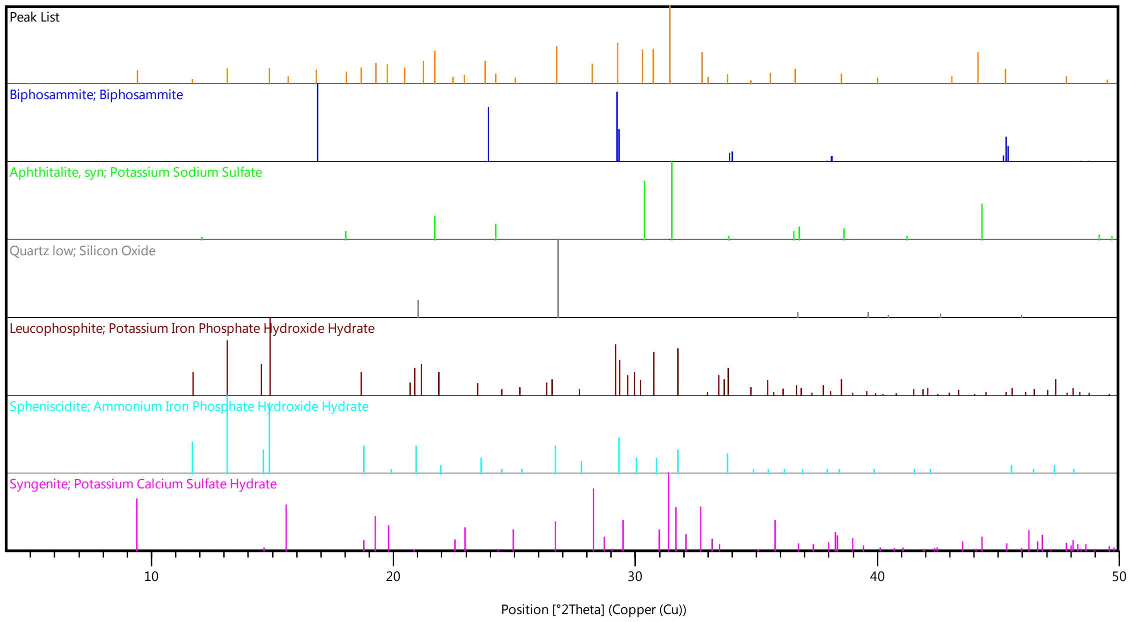

Escola Politécnica da Universidade de São Paulo
Departamento de Engenharia de Minas e de Petróleo
Laboratório de Caracterização Tecnológica
Av. Prof. Mello Moraes, 2373 CEP 05508-030 São Paulo - SP www.lct.poli.usp.br
Tel: 11 3091-5151 e-mail: lct@lct.poli.usp.br

RESULTADO DE IDENTIFICAÇÃO DE FASES POR DIFRATOMETRIA DE RAIOS X

RELATÓRIO: DRX 612/19 **DATA:** 12/04/19

CLIENTE: Luís Piló

AMOSTRA: N3-23-BG **IDENT. LCT:** 130-2099.HPF

1. MÉTODO

O estudo foi efetuado através do método do pó, mediante o emprego de difratômetro de raios X com detector sensível a posição.

A identificação das fases cristalinas, abaixo discriminadas, foi obtida por comparação do difratograma da amostra com os bancos de dados PDF2 do ICDD - International Centre for Diffraction Data e ICSD – Inorganic Crystal Structure Database.

2. RESULTADOS

Os resultados obtidos estão listados na tabela abaixo:

ICDD	Mineral/Composto	Fórmula Química	Obs
98-001-7872	Spheniscidita	$(NH_4)Fe_2(PO_4)_2(OH)\ 2H_2O$	e/ou
01-088-0651	Leucofosfita	$K(Fe_2(PO_4)_2(OH)(H_2O))(H_2O)$	
	Fase amorfa		
00-022-1037	Sulfato de amônio e cálcio	$(NH_4)_2Ca_2(SO_4)_3$	pp
00-029-0981	Taranakita	$H_6K_3Al_5(PO_4)_8\ 18H_2O$	pp

Nota: pp = possível presença

O difratograma obtido (cor vermelha), onde são assinaladas as linhas de difração correspondente(s) à(s) fase(s) identificada(s) (cada fase em uma cor distinta) é apresentado anexo.

Profa. Dra. Carina Ulsen
Coordenadora do LCT

Dra. Maria Manuela Tassinari
Pesquisadora sênior

Dra. Juliana Lívi Antoniassi
Pesquisadora

Verifique a autenticidade deste documento em www.lct.poli.usp.br utilizando o código HKQA-AKJA-QVTA-WWXB

DIFRATOGRAMA DE RAIOS X

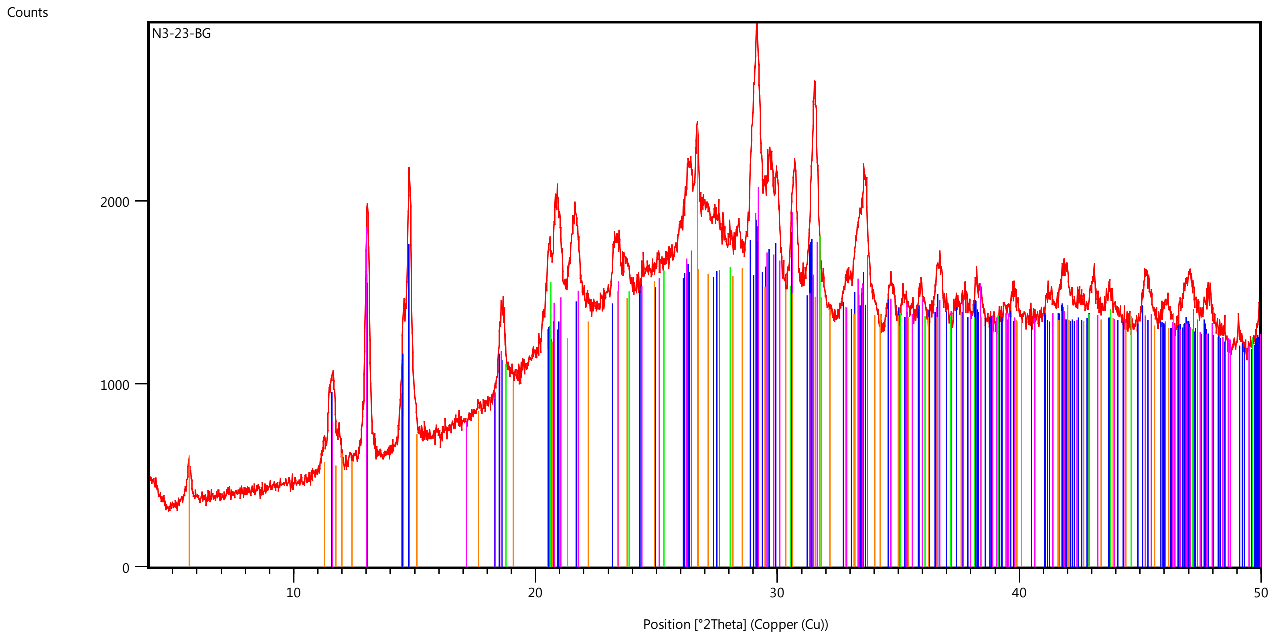

FASES IDENTIFICADAS

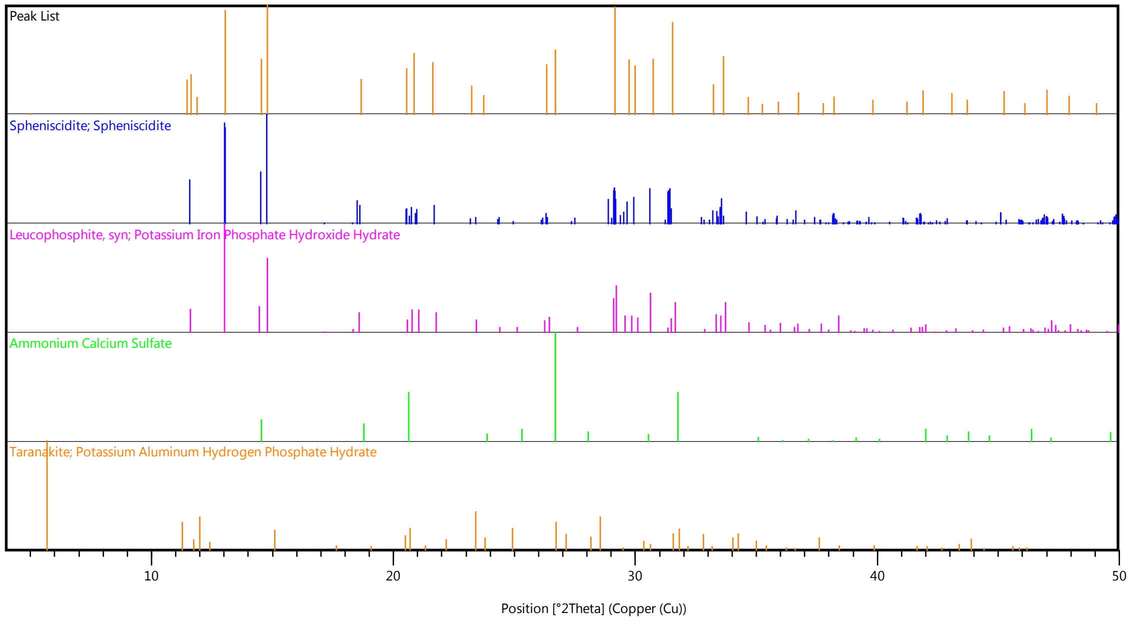

Escola Politécnica da Universidade de São Paulo
Departamento de Engenharia de Minas e de Petróleo
Laboratório de Caracterização Tecnológica
Av. Prof. Mello Moraes, 2373 CEP 05508-030 São Paulo - SP www.lct.poli.usp.br
Tel: 11 3091-5151 e-mail: lct@lct.poli.usp.br

RESULTADO DE IDENTIFICAÇÃO DE FASES POR DIFRATOMETRIA DE RAIOS X

RELATÓRIO: DRX 613/19 **DATA:** 12/04/19

CLIENTE: Luís Piló

AMOSTRA: N5S-63-SUP **IDENT. LCT:** 130-2100.HPF

1. MÉTODO

O estudo foi efetuado através do método do pó, mediante o emprego de difratômetro de raios X com detector sensível a posição.

A identificação das fases cristalinas, abaixo discriminadas, foi obtida por comparação do difratograma da amostra com os bancos de dados PDF2 do ICDD - International Centre for Diffraction Data e ICSD – Inorganic Crystal Structure Database.

2. RESULTADOS

Os resultados obtidos estão listados na tabela abaixo:

ICDD	Mineral/Composto	Fórmula Química	Obs
01-074-2052	Nitrato de amônio e potássio	$K_{0,250}(NH_4)_{0,750}NO_3$	
98-007-9199	Biphosammita	$(NH_4)(H_2PO_4)$	
98-003-4677	Bassanita	$Ca(SO_4)(H_2O)_{0,5}$	
00-011-0117	Syngenita	$K_2Ca(SO_4)_2\ H_2O$	
	Fase amorfa		
01-089-0950	Magnetita	Fe_3O_4	pp
00-014-0281	Sjogrenita	$Mg_6Fe_2CO_3(OH)_{16}\ 4H_2O$	pp

Nota: pp = possível presença

O difratograma obtido (cor vermelha), onde são assinaladas as linhas de difração correspondente(s) à(s) fase(s) identificada(s) (cada fase em uma cor distinta) é apresentado anexo.

Profa. Dra. Carina Ulsen
Coordenadora do LCT

Dra. Maria Manuela Tassinari
Pesquisadora sênior

Dra. Juliana Lívi Antoniassi
Pesquisadora

Verifique a autenticidade deste documento em www.lct.poli.usp.br utilizando o código LKQA-ALJA-EATA-SEXB

DIFRATOGRAMA DE RAIOS X

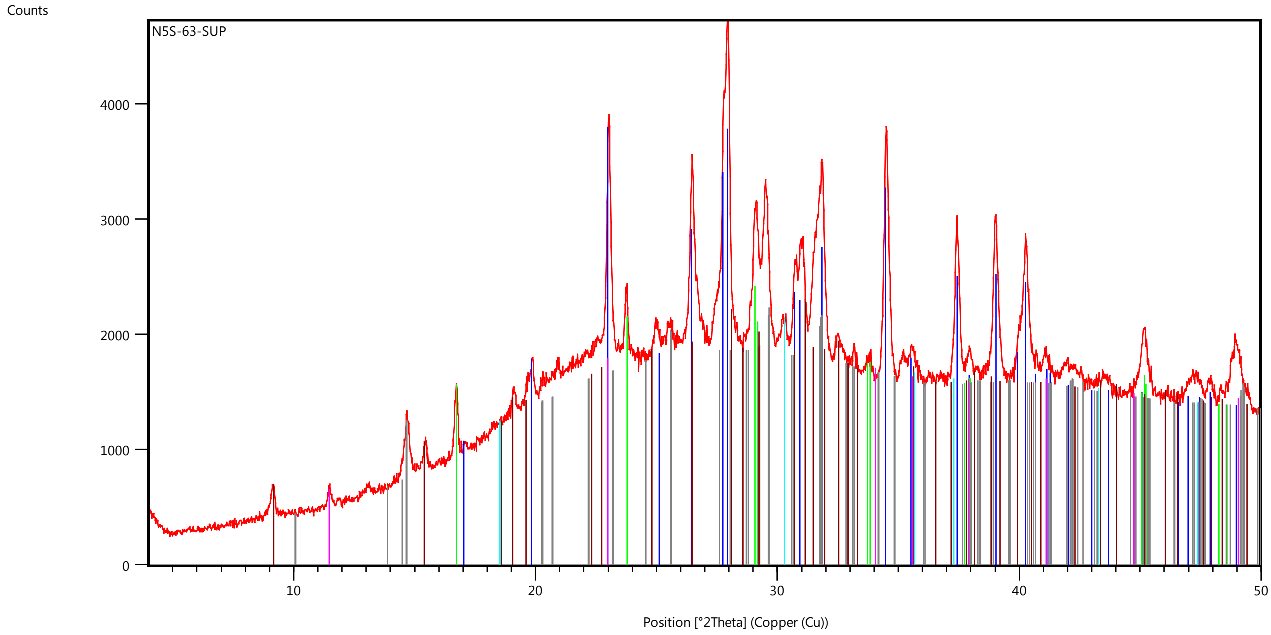

FASES IDENTIFICADAS

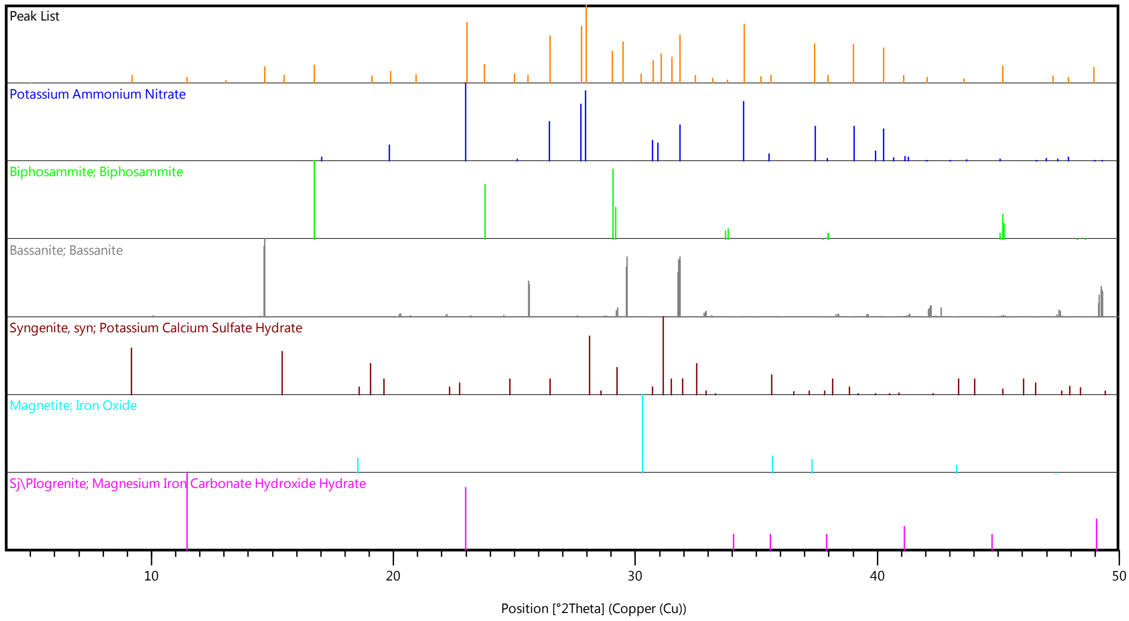

Escola Politécnica da Universidade de São Paulo
Departamento de Engenharia de Minas e de Petróleo
Laboratório de Caracterização Tecnológica
Av. Prof. Mello Moraes, 2373 CEP 05508-030 São Paulo - SP www.lct.poli.usp.br
Tel: 11 3091-5151 e-mail: lct@lct.poli.usp.br

RESULTADO DE IDENTIFICAÇÃO DE FASES POR DIFRATOMETRIA DE RAIOS X

RELATÓRIO: DRX 614/19 **DATA:** 12/04/19

CLIENTE: Luís Piló

AMOSTRA: N5S-63-60 **IDENT. LCT:** 130-2101.HPF

1. MÉTODO

O estudo foi efetuado através do método do pó, mediante o emprego de difratômetro de raios X com detector sensível a posição.

A identificação das fases cristalinas, abaixo discriminadas, foi obtida por comparação do difratograma da amostra com os bancos de dados PDF2 do ICDD - International Centre for Diffraction Data e ICSD – Inorganic Crystal Structure Database.

2. RESULTADOS

Os resultados obtidos estão listados na tabela abaixo:

ICDD	Mineral/Composto	Fórmula Química	Obs
01-074-2052	Nitrato de amônio e potássio	$K_{0,250}(NH_4)_{0,750}NO_3$	
01-070-0259	Hannayita	$Mg_3(NH_4)_2(HPO_4)_4(H_2O)_8$	
00-041-0593	Spheniscidita	$(NH_4)Fe_2(PO_4)_2(OH)\ 2H_2O$	
	Fase amorfa		

O difratograma obtido (cor vermelha), onde são assinaladas as linhas de difração correspondente(s) à(s) fase(s) identificada(s) (cada fase em uma cor distinta) é apresentado anexo.

Profa. Dra. Carina Ulsen
Coordenadora do LCT

Dra. Maria Manuela Tassinari
Pesquisadora sênior

Dra. Juliana Lívi Antoniassi
Pesquisadora

Verifique a autenticidade deste documento em www.lct.poli.usp.br utilizando o código **FKQA-YLJA-WETA-SOXB**

DIFRATOGRAMA DE RAIOS X

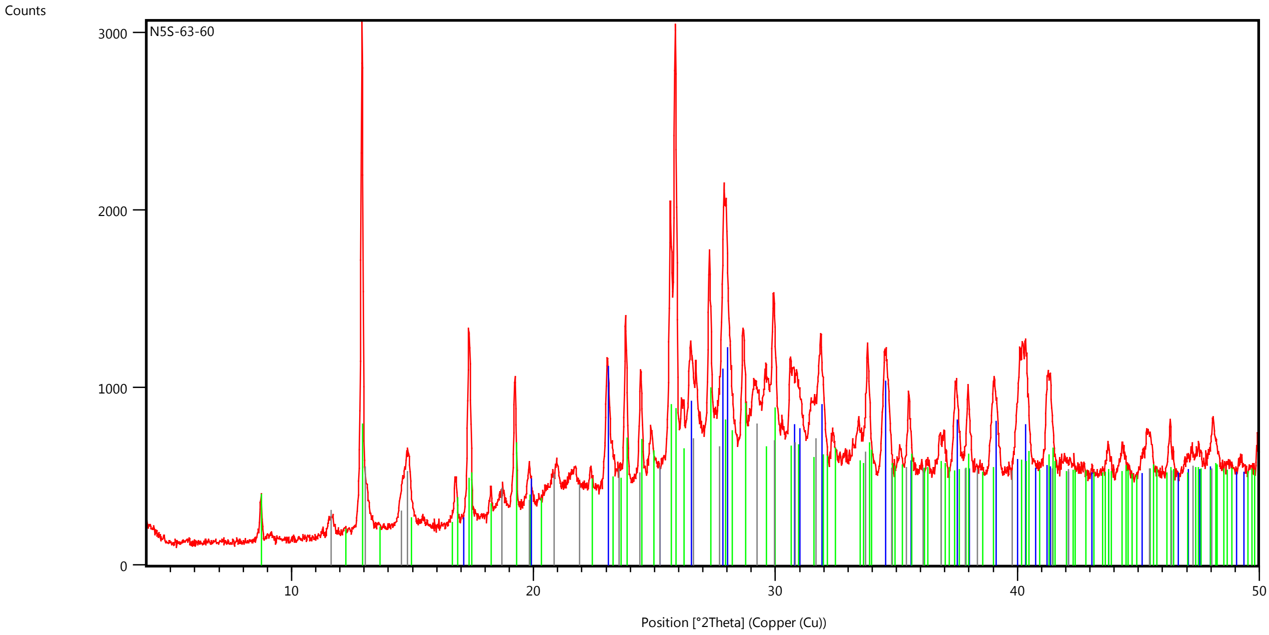

FASES IDENTIFICADAS

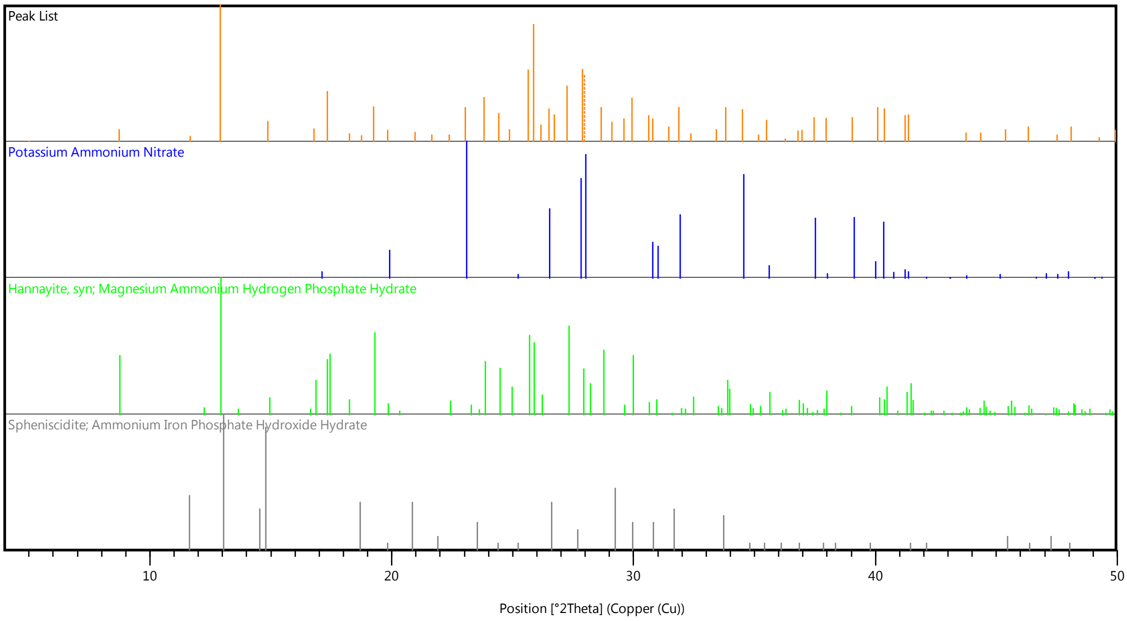

Escola Politécnica da Universidade de São Paulo
Departamento de Engenharia de Minas e de Petróleo
Laboratório de Caracterização Tecnológica
Av. Prof. Mello Moraes, 2373 CEP 05508-030 São Paulo - SP www.lct.poli.usp.br
Tel: 11 3091-5151 e-mail: lct@lct.poli.usp.br

RESULTADO DE IDENTIFICAÇÃO DE FASES POR DIFRATOMETRIA DE RAIOS X

RELATÓRIO: DRX 615/19 **DATA:** 12/04/19

CLIENTE: Luís Piló

AMOSTRA: S11C-41-SUP **IDENT. LCT:** 130-2102.HPF

1. MÉTODO

O estudo foi efetuado através do método do pó, mediante o emprego de difratômetro de raios X com detector sensível a posição.

A identificação das fases cristalinas, abaixo discriminadas, foi obtida por comparação do difratograma da amostra com os bancos de dados PDF2 do ICDD - International Centre for Diffraction Data e ICSD – Inorganic Crystal Structure Database.

2. RESULTADOS

Os resultados obtidos estão listados na tabela abaixo:

ICDD	Mineral/Composto	Fórmula Química	Obs
01-074-2051	Nitrato de amônio e potássio	$(NH_4)_{0,88}K_{0,12}NO_3$	
98-007-9199	Biphosammita	$(NH_4)(H_2PO_4)$	
	Fase amorfa		
01-075-0443	Quartzo	SiO_2	pp
01-076-0933	Cristobalita	SiO_2	pp
01-082-1164	Spheniscidita	$Fe_2(NH_4)(OH)(PO_4)_2(H_2O)2$	pp
01-074-1118	Syngenita	$K_2Ca(SO_4)_2(H_2O)$	pp

Nota: pp = possível presença

O difratograma obtido (cor vermelha), onde são assinaladas as linhas de difração correspondente(s) à(s) fase(s) identificada(s) (cada fase em uma cor distinta) é apresentado anexo.

Profa. Dra. Carina Ulsen
Coordenadora do LCT

Dra. Maria Manuela Tassinari
Pesquisadora sênior

Dra. Juliana Lívi Antoniassi
Pesquisadora

Verifique a autenticidade deste documento em www.lct.poli.usp.br utilizando o código **XKQA-ELJA-XITA-GWXB**

DIFRATOGRAMA DE RAIOS X

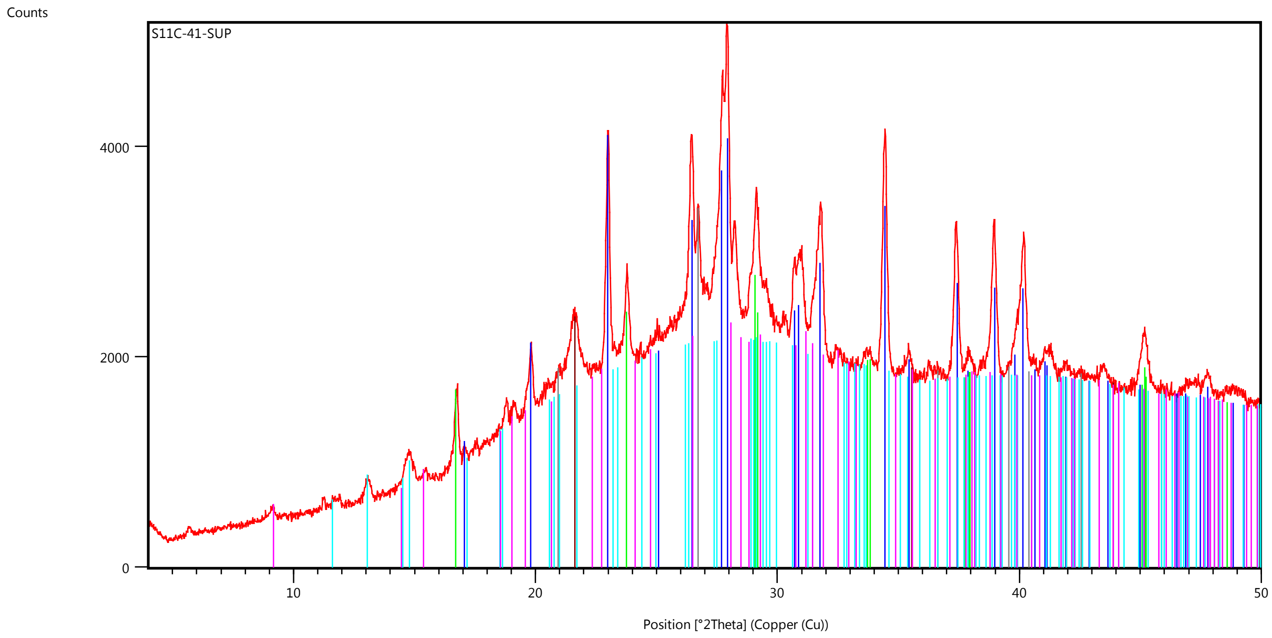

FASES IDENTIFICADAS

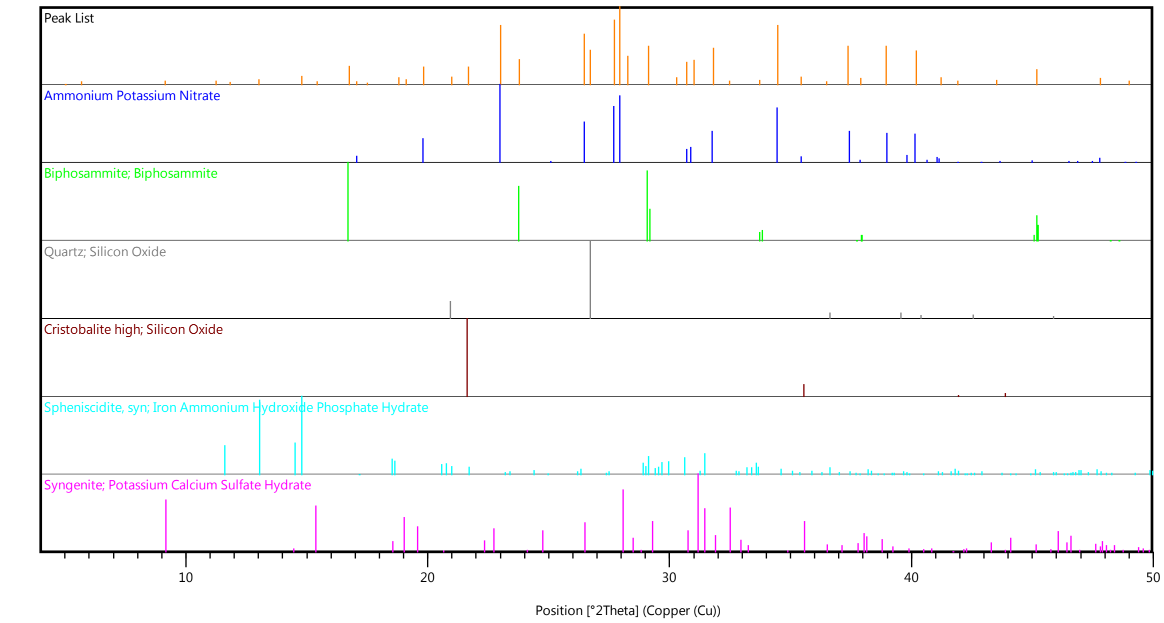

Escola Politécnica da Universidade de São Paulo
Departamento de Engenharia de Minas e de Petróleo
Laboratório de Caracterização Tecnológica
Av. Prof. Mello Moraes, 2373 CEP 05508-030 São Paulo - SP www.lct.poli.usp.br
Tel: 11 3091-5151 e-mail: lct@lct.poli.usp.br

RESULTADO DE IDENTIFICAÇÃO DE FASES POR DIFRATOMETRIA DE RAIOS X

RELATÓRIO: DRX 1226/19 **DATA:** 11/12/2019

CLIENTE: Luís Piló

AMOSTRA: N3-23-TM-A **IDENT. LCT:** 511-10065.HPF

1. MÉTODO

O estudo foi efetuado através do método do pó, mediante o emprego de difratômetro de raios X com detector sensível a posição.

A identificação das fases cristalinas, abaixo discriminadas, foi obtida por comparação do difratograma da amostra com os bancos de dados PDF2 do ICDD - International Centre for Diffraction Data e ICSD – Inorganic Crystal Structure Database.

2. RESULTADOS

Os resultados obtidos estão listados na tabela abaixo:

ICDD	Mineral	Fórmula Química	Obs
01-074-1904	Gipsita	$Ca(SO_4)(H_2O)_2$	

O difratograma obtido (cor vermelha), onde são assinaladas as linhas de difração correspondente(s) à(s) fase(s) identificada(s) (cada fase em uma cor distinta) é apresentado anexo.

Executado por: M.Sc. Gaspar Darin Filho (16/12/2019 18:04 BRT)
Revisado por: Dra. Maria Manuela Tassinari (16/12/2019 18:24 BRT)

Profa. Dra. Carina Ulsen
Coordenadora do LCT - Poli/USP

NOTA: Os resultados expostos acima referem-se apenas à(s) amostra(s) enviada(s) ao LCT; a representatividade da(s) mesma(s) é de inteira responsabilidade do cliente.

Verifique a autenticidade deste documento em www.lct.poli.usp.br utilizando o código **UFQV-QYYU-SAUT-IPIB**

DIFRATOGRAMA DE RAIOS X

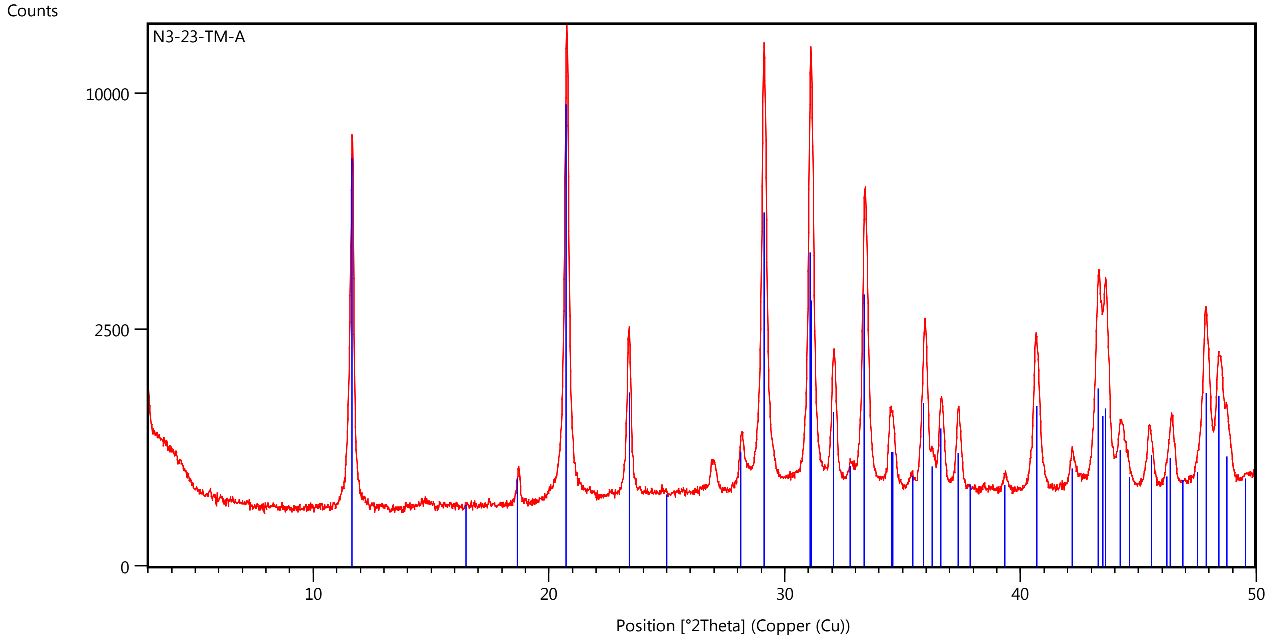

FASES IDENTIFICADAS

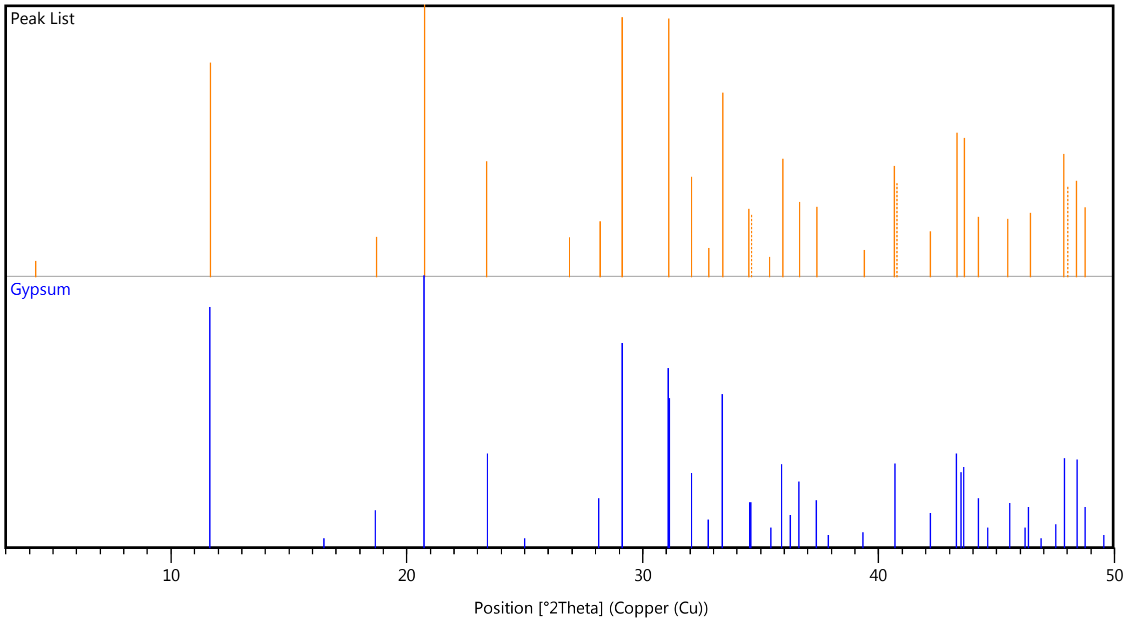

Escola Politécnica da Universidade de São Paulo
Departamento de Engenharia de Minas e de Petróleo
Laboratório de Caracterização Tecnológica
Av. Prof. Mello Moraes, 2373 CEP 05508-030 São Paulo - SP www.lct.poli.usp.br
Tel: 11 3091-5151 e-mail: lct@lct.poli.usp.br

RESULTADO DE IDENTIFICAÇÃO DE FASES POR DIFRATOMETRIA DE RAIOS X

RELATÓRIO: DRX 1227/19 **DATA:** 11/12/2019

CLIENTE: Luís Piló

AMOSTRA: N3-23-TM-B **IDENT. LCT:** 511-10066.HPF

1. MÉTODO

O estudo foi efetuado através do método do pó, mediante o emprego de difratômetro de raios X com detector sensível a posição.

A identificação das fases cristalinas, abaixo discriminadas, foi obtida por comparação do difratograma da amostra com os bancos de dados PDF2 do ICDD - International Centre for Diffraction Data e ICSD – Inorganic Crystal Structure Database.

2. RESULTADOS

Os resultados obtidos estão listados na tabela abaixo:

ICDD	Mineral	Fórmula Química	Obs
01-082-1164	Spheniscidita	$Fe_2(NH_4)(OH)(PO_4)_2(H_2O)_2$	
00-029-0981	Taranakita	$H_6K_3Al_5(PO_4)_8$ $18H_2O$	

O difratograma obtido (cor vermelha), onde são assinaladas as linhas de difração correspondente(s) à(s) fase(s) identificada(s) (cada fase em uma cor distinta) é apresentado anexo.

Executado por: M.Sc. Gaspar Darin Filho (16/12/2019 18:04 BRT)
Revisado por: Dra. Maria Manuela Tassinari (16/12/2019 18:24 BRT)

Profa. Dra. Carina Ulsen
Coordenadora do LCT - Poli/USP

NOTA: Os resultados expostos acima referem-se apenas à(s) amostra(s) enviada(s) ao LCT; a representatividade da(s) mesma(s) é de inteira responsabilidade do cliente.

Verifique a autenticidade deste documento em www.lct.poli.usp.br utilizando o código **SFQW-OYYU-OHUT-MMIB**

DIFRATOGRAMA DE RAIOS X

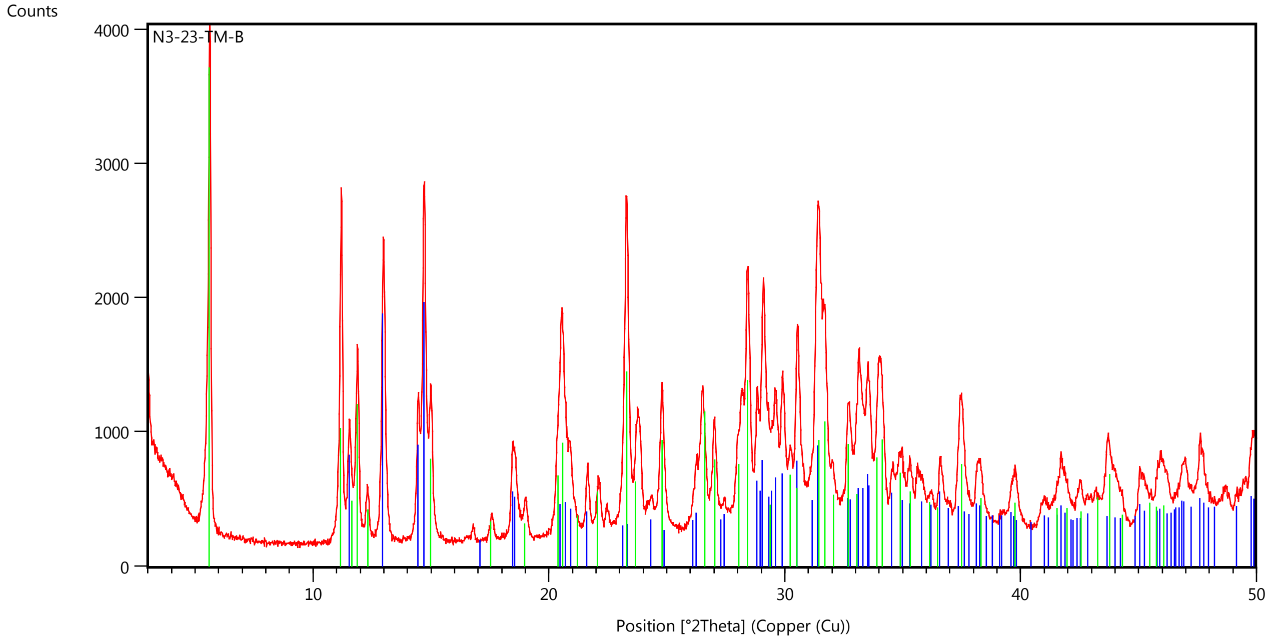

FASES IDENTIFICADAS

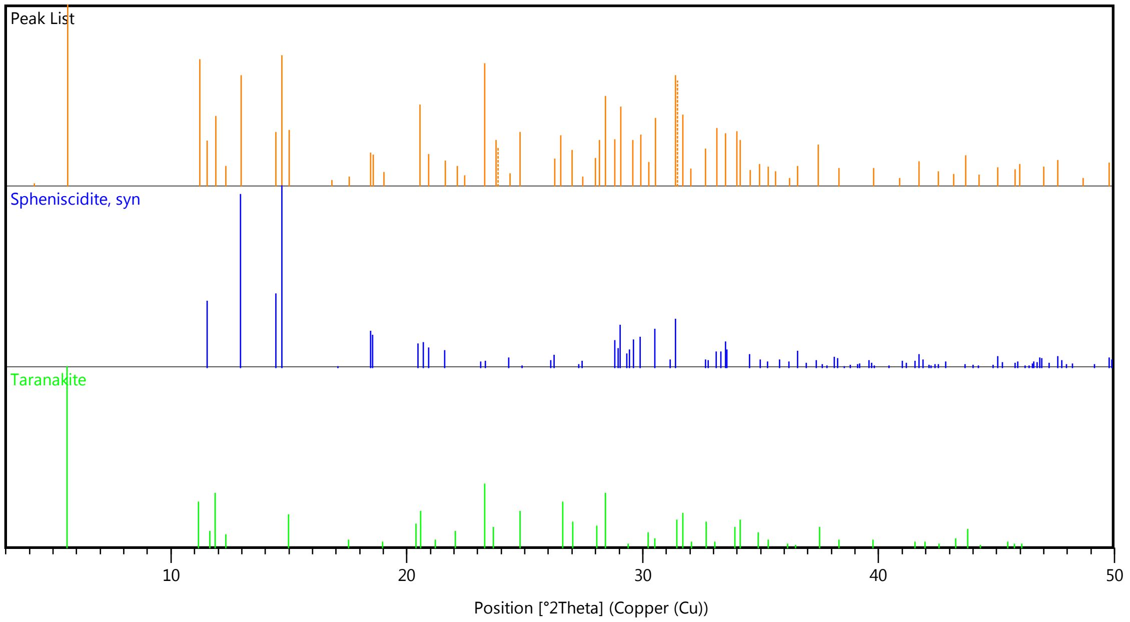

Escola Politécnica da Universidade de São Paulo
Departamento de Engenharia de Minas e de Petróleo
Laboratório de Caracterização Tecnológica
Av. Prof. Mello Moraes, 2373 CEP 05508-030 São Paulo - SP www.lct.poli.usp.br
Tel: 11 3091-5151 e-mail: lct@lct.poli.usp.br

RESULTADO DE IDENTIFICAÇÃO DE FASES POR DIFRATOMETRIA DE RAIOS X

RELATÓRIO: DRX 1218/19 **DATA:** 11/12/2019

CLIENTE: Luís Piló

AMOSTRA: M2-99-C1 **IDENT. LCT:** 511-10053.HPF

1. MÉTODO

O estudo foi efetuado através do método do pó, mediante o emprego de difratômetro de raios X com detector sensível a posição.

A identificação das fases cristalinas, abaixo discriminadas, foi obtida por comparação do difratograma da amostra com os bancos de dados PDF2 do ICDD - International Centre for Diffraction Data e ICSD – Inorganic Crystal Structure Database.

2. RESULTADOS

Os resultados obtidos estão listados na tabela abaixo:

ICDD	Mineral	Fórmula Química	Obs
	Fase amorfa		
00-037-1479	Bifosfammita	$NH_4H_2PO_4$	
00-050-1566	Gwihabaita	$(NH_4,K)NO_3$	
01-088-0651	Leucofosfita	$K(Fe_2(PO_4)_2(OH)(H_2O))(H_2O)$	
01-070-0259	Hannayita	$Mg_3(NH_4)_2(HPO_4)_4(H_2O)_8$	pp
00-011-0117	Syngenita	$K_2Ca(SO_4)_2\,H_2O$	pp

Nota: pp = possível presença

O difratograma obtido (cor vermelha), onde são assinaladas as linhas de difração correspondente(s) à(s) fase(s) identificada(s) (cada fase em uma cor distinta) é apresentado anexo.

Executado por: M.Sc. Gaspar Darin Filho (16/12/2019 18:04 BRT)
Revisado por: Dra. Maria Manuela Tassinari (16/12/2019 18:24 BRT)

Profa. Dra. Carina Ulsen
Coordenadora do LCT - Poli/USP

NOTA: Os resultados expostos acima referem-se apenas à(s) amostra(s) enviada(s) ao LCT; a representatividade da(s) mesma(s) é de inteira responsabilidade do cliente.

Verifique a autenticidade deste documento em www.lct.poli.usp.br utilizando o código **YFQN-QVYU-WNUT-QOIB**

DIFRATOGRAMA DE RAIOS X

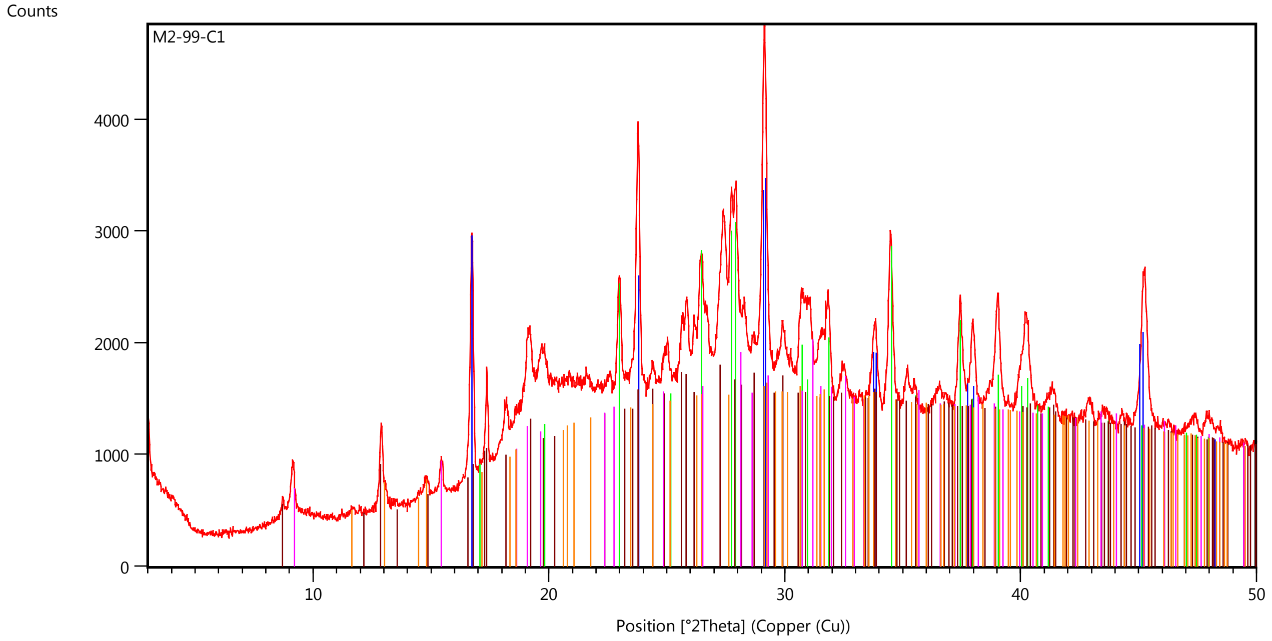

FASES IDENTIFICADAS

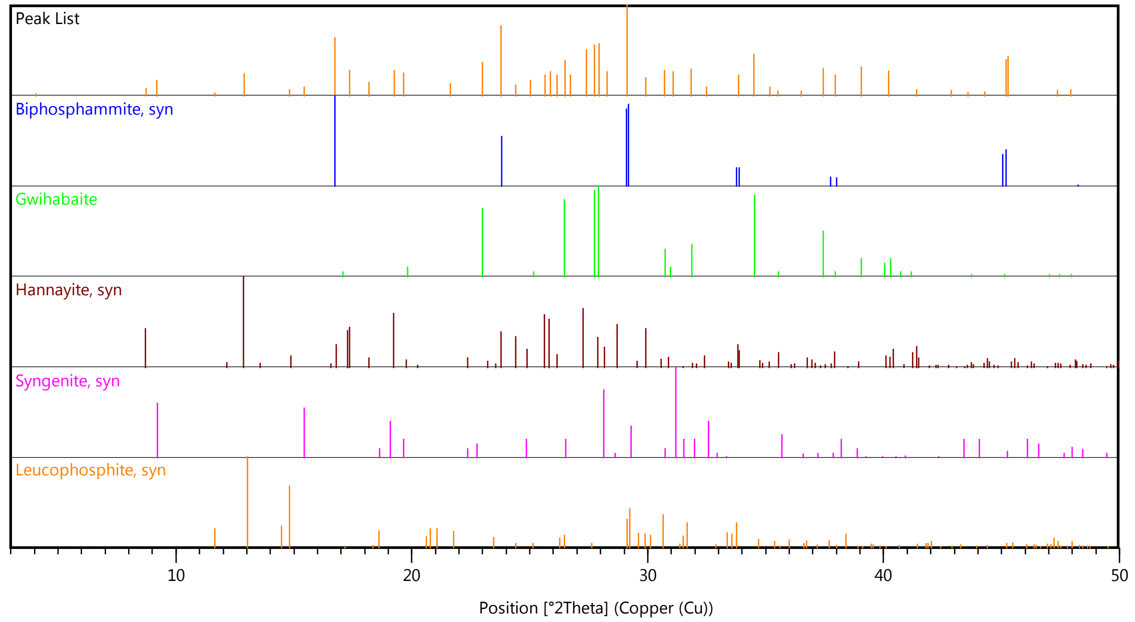

Escola Politécnica da Universidade de São Paulo
Departamento de Engenharia de Minas e de Petróleo
Laboratório de Caracterização Tecnológica
Av. Prof. Mello Moraes, 2373 CEP 05508-030 São Paulo - SP www.lct.poli.usp.br
Tel: 11 3091-5151 e-mail: lct@lct.poli.usp.br

RESULTADO DE IDENTIFICAÇÃO DE FASES POR DIFRATOMETRIA DE RAIOS X

RELATÓRIO: DRX 1219/19 **DATA:** 11/12/2019

CLIENTE: Luís Piló

AMOSTRA: M2-99-C2 **IDENT. LCT:** 511-10054.HPF

1. MÉTODO

O estudo foi efetuado através do método do pó, mediante o emprego de difratômetro de raios X com detector sensível a posição.

A identificação das fases cristalinas, abaixo discriminadas, foi obtida por comparação do difratograma da amostra com os bancos de dados PDF2 do ICDD - International Centre for Diffraction Data e ICSD – Inorganic Crystal Structure Database.

2. RESULTADOS

Os resultados obtidos estão listados na tabela abaixo:

ICDD	Mineral	Fórmula Química	Obs
01-070-0259	Hannayita	$Mg_3(NH_4)_2(HPO_4)_4(H_2O)_8$	
01-074-1904	Gipsita	$Ca(SO_4)(H_2O)_2$	
01-082-1164	Spheniscidita	$Fe_2(NH_4)(OH)(PO_4)_2(H_2O)_2$	
01-085-0815	Bifosammita	$NH_4H_2PO_4$	
	Fase amorfa		
00-029-0981	Taranakita	$H_6K_3Al_5(PO_4)_8$ $18H_2O$	pp
00-011-0117	Syngenita	$K_2Ca(SO_4)_2$ H_2O	pp

Nota: pp = possível presença

O difratograma obtido (cor vermelha), onde são assinaladas as linhas de difração correspondente(s) à(s) fase(s) identificada(s) (cada fase em uma cor distinta) é apresentado anexo.

Executado por: M.Sc. Gaspar Darin Filho (16/12/2019 18:04 BRT)
Revisado por: Dra. Maria Manuela Tassinari (16/12/2019 18:24 BRT)

Profa. Dra. Carina Ulsen
Coordenadora do LCT - Poli/USP

NOTA: Os resultados expostos acima referem-se apenas à(s) amostra(s) enviada(s) ao LCT; a representatividade da(s) mesma(s) é de inteira responsabilidade do cliente.

Verifique a autenticidade deste documento em www.lct.poli.usp.br utilizando o código **WFQO-NVYU-VVUT-EAIB**

DIFRATOGRAMA DE RAIOS X

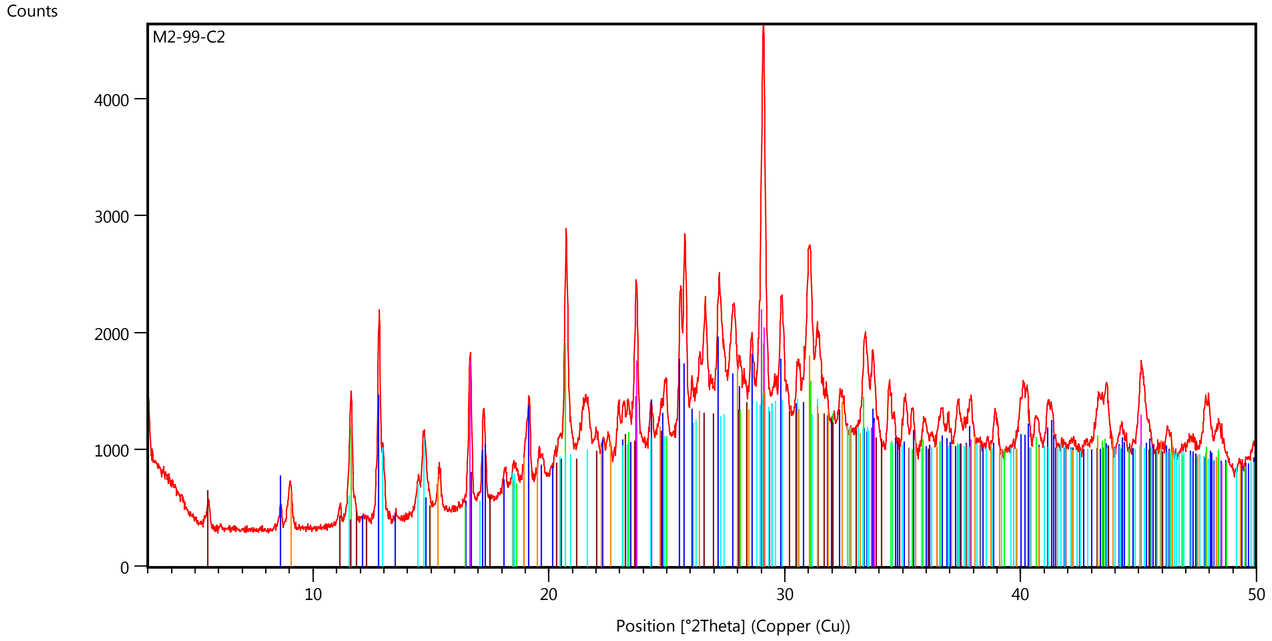

FASES IDENTIFICADAS

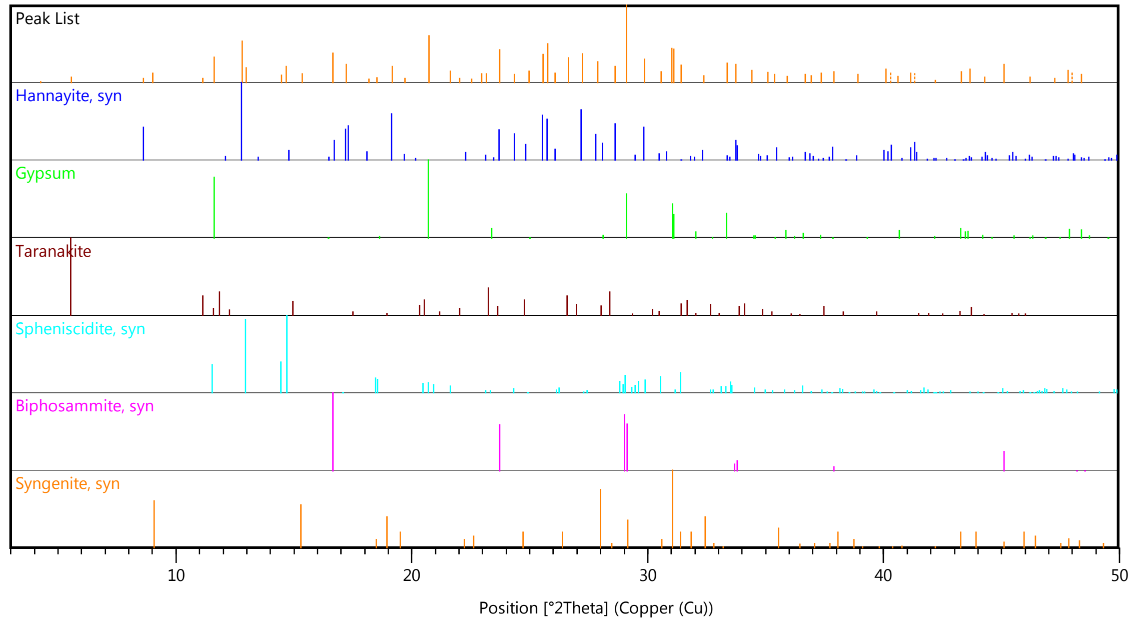

Escola Politécnica da Universidade de São Paulo
Departamento de Engenharia de Minas e de Petróleo
Laboratório de Caracterização Tecnológica
Av. Prof. Mello Moraes, 2373 CEP 05508-030 São Paulo - SP www.lct.poli.usp.br
Tel: 11 3091-5151 e-mail: lct@lct.poli.usp.br

RESULTADO DE IDENTIFICAÇÃO DE FASES POR DIFRATOMETRIA DE RAIOS X

RELATÓRIO: DRX 1220/19 **DATA:** 11/12/2019

CLIENTE: Luís Piló

AMOSTRA: M2-99-C3 **IDENT. LCT:** 511-10055.HPF

1. MÉTODO

O estudo foi efetuado através do método do pó, mediante o emprego de difratômetro de raios X com detector sensível a posição.

A identificação das fases cristalinas, abaixo discriminadas, foi obtida por comparação do difratograma da amostra com os bancos de dados PDF2 do ICDD - International Centre for Diffraction Data e ICSD – Inorganic Crystal Structure Database.

2. RESULTADOS

Os resultados obtidos estão listados na tabela abaixo:

ICDD	Mineral	Fórmula Química	Obs
01-074-1904	Gipsita	$Ca(SO_4)(H_2O)_2$	
00-037-1479	Bifosfammita	$NH_4H_2PO_4$	
	Fase amorfa		
01-082-1164	Spheniscidita	$Fe_2(NH_4)(OH)(PO_4)_2(H_2O)_2$	
00-011-0117	Syngenita	$K_2Ca(SO_4)_2\ H_2O$	
00-029-0981	Taranakita	$H_6K_3Al_5(PO_4)_8\ 18H_2O$	pp

Nota: pp = possível presença

O difratograma obtido (cor vermelha), onde são assinaladas as linhas de difração correspondente(s) à(s) fase(s) identificada(s) (cada fase em uma cor distinta) é apresentado anexo.

Executado por: M.Sc. Gaspar Darin Filho (16/12/2019 18:04 BRT)
Revisado por: Dra. Maria Manuela Tassinari (16/12/2019 18:24 BRT)

Profa. Dra. Carina Ulsen
Coordenadora do LCT - Poli/USP

NOTA: Os resultados expostos acima referem-se apenas à(s) amostra(s) enviada(s) ao LCT; a representatividade da(s) mesma(s) é de inteira responsabilidade do cliente.

Verifique a autenticidade deste documento em www.lct.poli.usp.br utilizando o código **GFQP-RWYU-WCUT-WLIB**

DIFRATOGRAMA DE RAIOS X

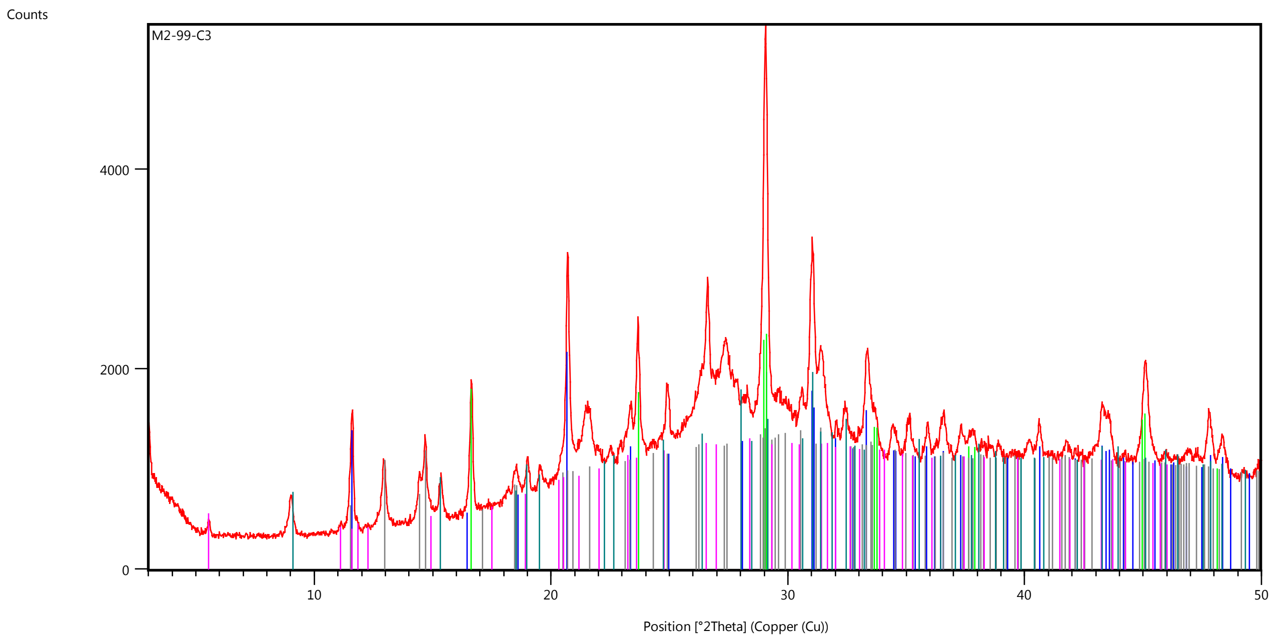

FASES IDENTIFICADAS

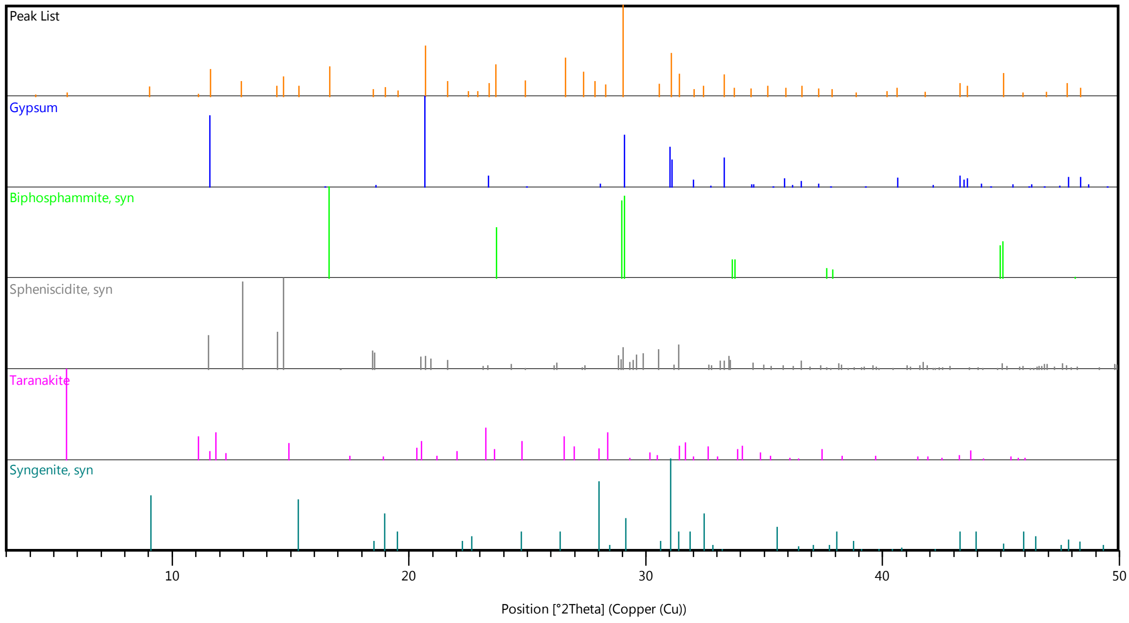

Escola Politécnica da Universidade de São Paulo
Departamento de Engenharia de Minas e de Petróleo
Laboratório de Caracterização Tecnológica
Av. Prof. Mello Moraes, 2373 CEP 05508-030 São Paulo - SP www.lct.poli.usp.br
Tel: 11 3091-5151 e-mail: lct@lct.poli.usp.br

RESULTADO DE IDENTIFICAÇÃO DE FASES POR DIFRATOMETRIA DE RAIOS X

RELATÓRIO: DRX 1221/19 **DATA:** 11/12/2019

CLIENTE: Luís Piló

AMOSTRA: M2-99-C4 **IDENT. LCT:** 511-10056.HPF

1. MÉTODO

O estudo foi efetuado através do método do pó, mediante o emprego de difratômetro de raios X com detector sensível a posição.

A identificação das fases cristalinas, abaixo discriminadas, foi obtida por comparação do difratograma da amostra com os bancos de dados PDF2 do ICDD - International Centre for Diffraction Data e ICSD – Inorganic Crystal Structure Database.

2. RESULTADOS

Os resultados obtidos estão listados na tabela abaixo:

ICDD	Mineral	Fórmula Química	Obs
01-088-0651	Leucofosfita	$K(Fe_2(PO_4)_2(OH)(H_2O))(H_2O)$	
00-037-1479	Bifosfammita	$NH_4H_2PO_4$	
01-075-0443	Quartzo	SiO_2	
00-011-0117	Syngenita	$K_2Ca(SO_4)_2\ H_2O$	pp
	Fase amorfa		pp

Nota: pp = possível presença

O difratograma obtido (cor vermelha), onde são assinaladas as linhas de difração correspondente(s) à(s) fase(s) identificada(s) (cada fase em uma cor distinta) é apresentado anexo.

Executado por: M.Sc. Gaspar Darin Filho (16/12/2019 18:04 BRT)
Revisado por: Dra. Maria Manuela Tassinari (16/12/2019 18:24 BRT)

Profa. Dra. Carina Ulsen
Coordenadora do LCT - Poli/USP

NOTA: Os resultados expostos acima referem-se apenas à(s) amostra(s) enviada(s) ao LCT; a representatividade da(s) mesma(s) é de inteira responsabilidade do cliente.

Verifique a autenticidade deste documento em www.lct.poli.usp.br utilizando o código **DFQQ-JWYU-MKUT-SDIB**

DIFRATOGRAMA DE RAIOS X

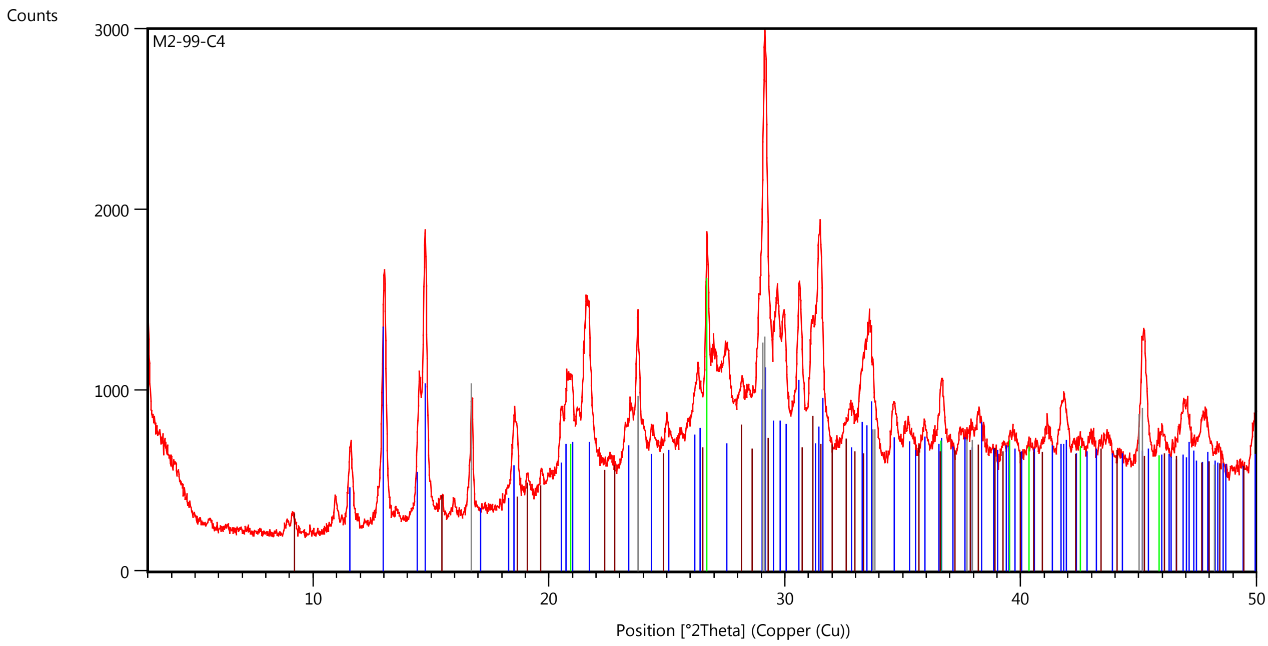

FASES IDENTIFICADAS

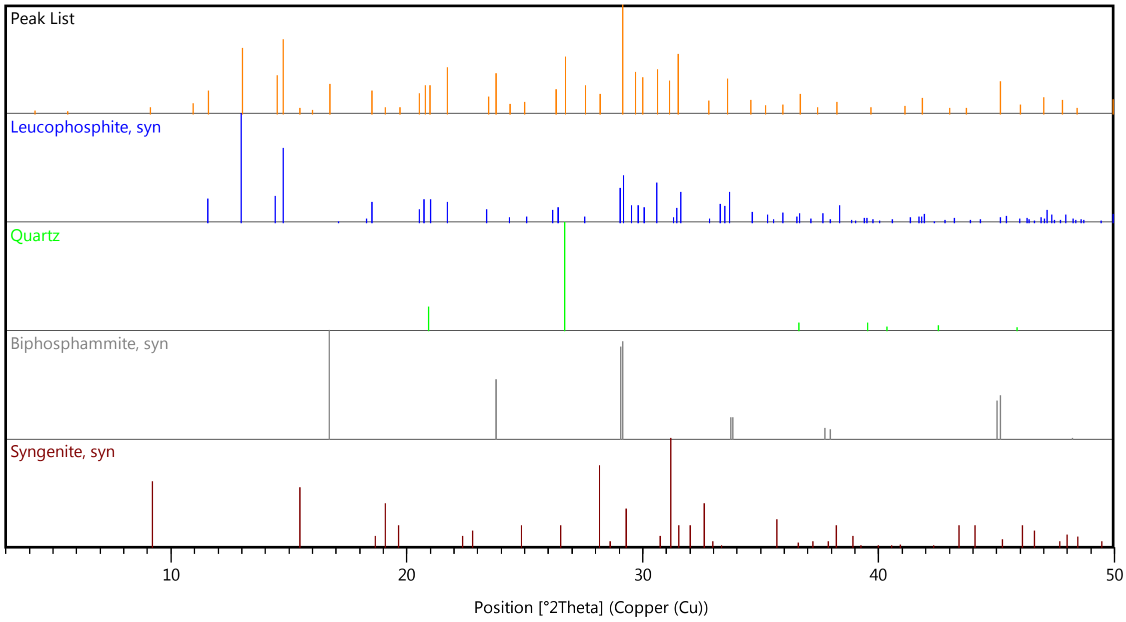

Escola Politécnica da Universidade de São Paulo
Departamento de Engenharia de Minas e de Petróleo
Laboratório de Caracterização Tecnológica
Av. Prof. Mello Moraes, 2373 CEP 05508-030 São Paulo - SP www.lct.poli.usp.br
Tel: 11 3091-5151 e-mail: lct@lct.poli.usp.br

RESULTADO DE IDENTIFICAÇÃO DE FASES POR DIFRATOMETRIA DE RAIOS X

RELATÓRIO: DRX 1208/19 **DATA:** 11/12/2019

CLIENTE: Luís Piló

AMOSTRA: N3-23-P1-C1 **IDENT. LCT:** 511-10043.HPF

1. MÉTODO

O estudo foi efetuado através do método do pó, mediante o emprego de difratômetro de raios X com detector sensível a posição.

A identificação das fases cristalinas, abaixo discriminadas, foi obtida por comparação do difratograma da amostra com os bancos de dados PDF2 do ICDD - International Centre for Diffraction Data e ICSD – Inorganic Crystal Structure Database.

2. RESULTADOS

Os resultados obtidos estão listados na tabela abaixo:

ICDD	Mineral	Fórmula Química	Obs
01-072-0713	Brushita	$CaHPO_4(H_2O)_2$	
01-074-1904	Gipsita	$Ca(SO_4)(H_2O)_2$	
01-082-1164	Spheniscidita	$Fe_2(NH_4)(OH)(PO_4)_2(H_2O)_2$	
	Fase amorfa		

O difratograma obtido (cor vermelha), onde são assinaladas as linhas de difração correspondente(s) à(s) fase(s) identificada(s) (cada fase em uma cor distinta) é apresentado anexo.

Executado por: M.Sc. Gaspar Darin Filho (16/12/2019 18:04 BRT)
Revisado por: Dra. Maria Manuela Tassinari (16/12/2019 18:24 BRT)

Profa. Dra. Carina Ulsen
Coordenadora do LCT - Poli/USP

NOTA: Os resultados expostos acima referem-se apenas à(s) amostra(s) enviada(s) ao LCT; a representatividade da(s) mesma(s) é de inteira responsabilidade do cliente.

Verifique a autenticidade deste documento em www.lct.poli.usp.br utilizando o código **ZFQD-OSYU-AXUT-KMIB**

Escola Politécnica da Universidade de São Paulo
Departamento de Engenharia de Minas e de Petróleo
Laboratório de Caracterização Tecnológica
Av. Prof. Mello Moraes, 2373 CEP 05508-030 São Paulo - SP www.lct.poli.usp.br
Tel: 11 3091-5151 e-mail: lct@lct.poli.usp.br

DIFRATOGRAMA DE RAIOS X

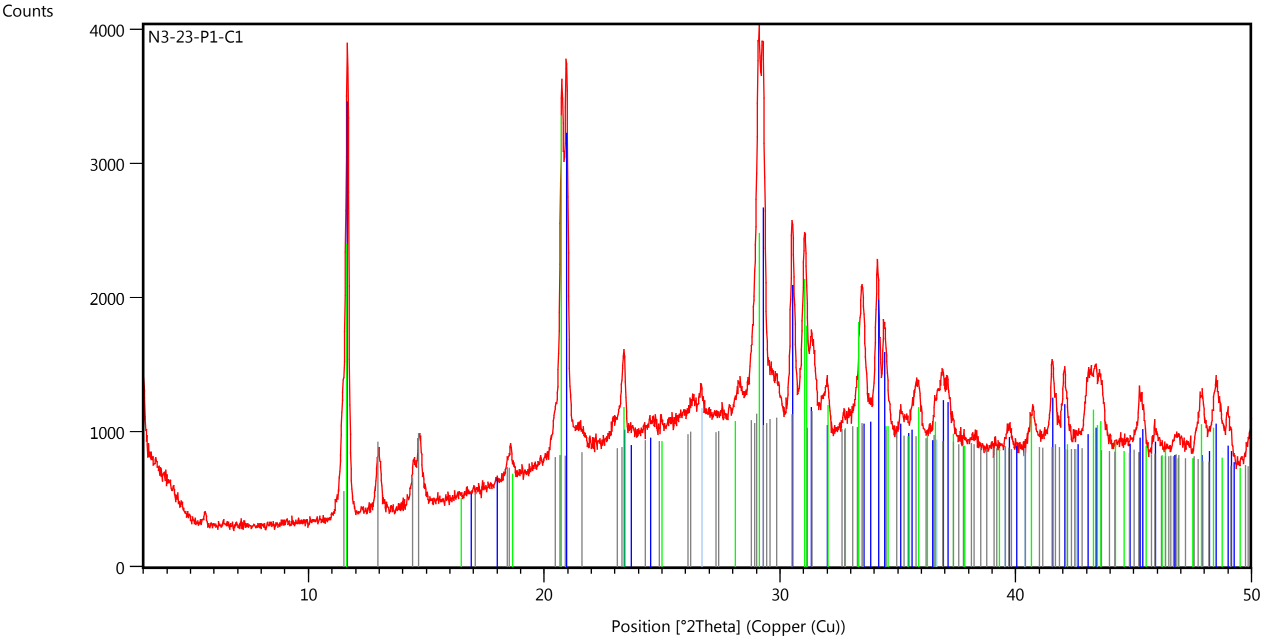

FASES IDENTIFICADAS

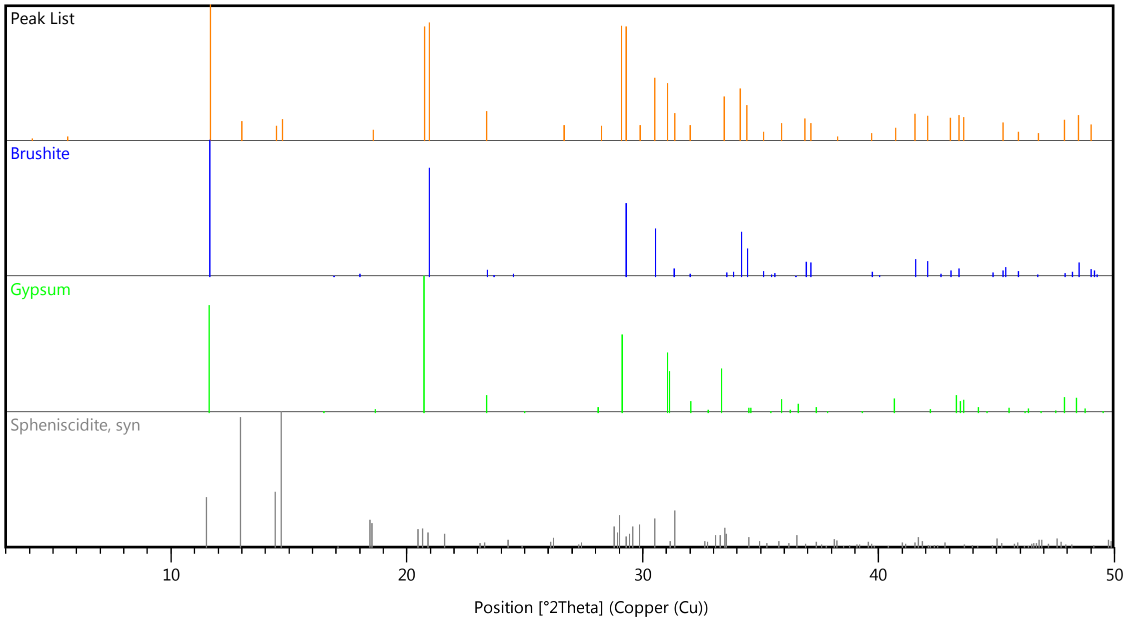

Escola Politécnica da Universidade de São Paulo
Departamento de Engenharia de Minas e de Petróleo
Laboratório de Caracterização Tecnológica
Av. Prof. Mello Moraes, 2373 CEP 05508-030 São Paulo - SP www.lct.poli.usp.br
Tel: 11 3091-5151 e-mail: lct@lct.poli.usp.br

RESULTADO DE IDENTIFICAÇÃO DE FASES POR DIFRATOMETRIA DE RAIOS X

RELATÓRIO: DRX 1209/19 **DATA:** 11/12/2019

CLIENTE: Luís Piló

AMOSTRA: N3-23-P1-C2 **IDENT. LCT:** 511-10044.HPF

1. MÉTODO

O estudo foi efetuado através do método do pó, mediante o emprego de difratômetro de raios X com detector sensível a posição.

A identificação das fases cristalinas, abaixo discriminadas, foi obtida por comparação do difratograma da amostra com os bancos de dados PDF2 do ICDD - International Centre for Diffraction Data e ICSD – Inorganic Crystal Structure Database.

2. RESULTADOS

Os resultados obtidos estão listados na tabela abaixo:

ICDD	Mineral	Fórmula Química	Obs
01-072-0713	Brushita	$CaHPO_4(H_2O)_2$	
01-074-1904	Gipsita	$Ca(SO_4)(H_2O)_2$	
01-082-1164	Spheniscidita	$Fe_2(NH_4)(OH)(PO_4)_2(H_2O)_2$	
	Fase amorfa		

O difratograma obtido (cor vermelha), onde são assinaladas as linhas de difração correspondente(s) à(s) fase(s) identificada(s) (cada fase em uma cor distinta) é apresentado anexo.

Executado por: M.Sc. Gaspar Darin Filho (16/12/2019 18:04 BRT)
Revisado por: Dra. Maria Manuela Tassinari (16/12/2019 18:24 BRT)

Profa. Dra. Carina Ulsen
Coordenadora do LCT - Poli/USP

NOTA: Os resultados expostos acima referem-se apenas à(s) amostra(s) enviada(s) ao LCT; a representatividade da(s) mesma(s) é de inteira responsabilidade do cliente.

Verifique a autenticidade deste documento em www.lct.poli.usp.br utilizando o código **NFQE-DSYU-JQUT-QCIB**

Escola Politécnica da Universidade de São Paulo
Departamento de Engenharia de Minas e de Petróleo
Laboratório de Caracterização Tecnológica
Av. Prof. Mello Moraes, 2373 CEP 05508-030 São Paulo - SP www.lct.poli.usp.br
Tel: 11 3091-5151 e-mail: lct@lct.poli.usp.br

DIFRATOGRAMA DE RAIOS X

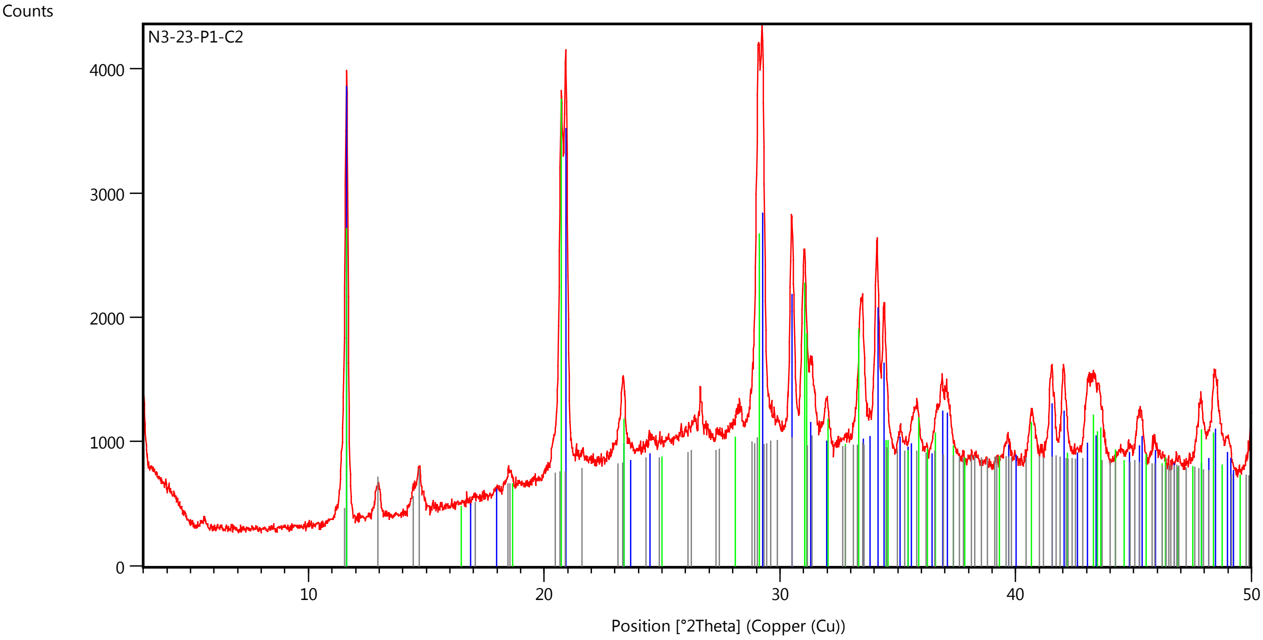

FASES IDENTIFICADAS

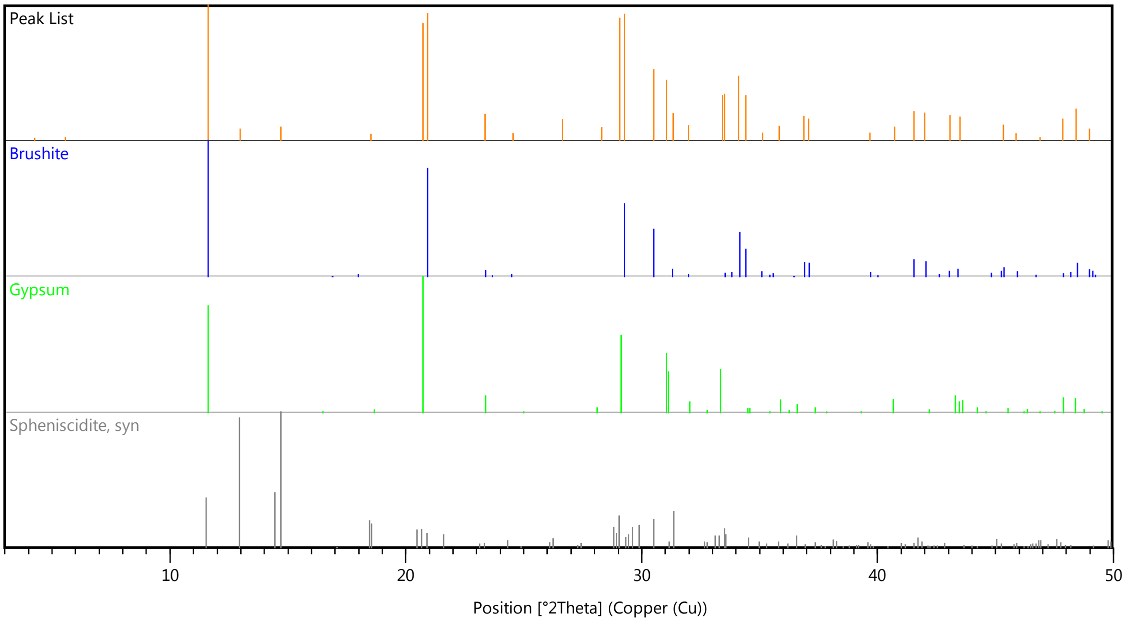

Escola Politécnica da Universidade de São Paulo
Departamento de Engenharia de Minas e de Petróleo
Laboratório de Caracterização Tecnológica
Av. Prof. Mello Moraes, 2373 CEP 05508-030 São Paulo - SP www.lct.poli.usp.br
Tel: 11 3091-5151 e-mail: lct@lct.poli.usp.br

RESULTADO DE IDENTIFICAÇÃO DE FASES POR DIFRATOMETRIA DE RAIOS X

RELATÓRIO: DRX 1210/19 **DATA:** 11/12/2019

CLIENTE: Luís Piló

AMOSTRA: N3-23-P1-C3 **IDENT. LCT:** 511-10045.HPF

1. MÉTODO

O estudo foi efetuado através do método do pó, mediante o emprego de difratômetro de raios X com detector sensível a posição.

A identificação das fases cristalinas, abaixo discriminadas, foi obtida por comparação do difratograma da amostra com os bancos de dados PDF2 do ICDD - International Centre for Diffraction Data e ICSD – Inorganic Crystal Structure Database.

2. RESULTADOS

Os resultados obtidos estão listados na tabela abaixo:

ICDD	Mineral	Fórmula Química	Obs
01-072-0713	Brushita	$CaHPO_4(H_2O)_2$	
01-074-1904	Gipsita	$Ca(SO_4)(H_2O)_2$	
01-082-1164	Spheniscidita	$Fe_2(NH_4)(OH)(PO_4)_2(H_2O)_2$	
	Fase amorfa		

O difratograma obtido (cor vermelha), onde são assinaladas as linhas de difração correspondente(s) à(s) fase(s) identificada(s) (cada fase em uma cor distinta) é apresentado anexo.

Executado por: M.Sc. Gaspar Darin Filho (16/12/2019 18:04 BRT)
Revisado por: Dra. Maria Manuela Tassinari (16/12/2019 18:24 BRT)

Profa. Dra. Carina Ulsen
Coordenadora do LCT - Poli/USP

NOTA: Os resultados expostos acima referem-se apenas à(s) amostra(s) enviada(s) ao LCT; a representatividade da(s) mesma(s) é de inteira responsabilidade do cliente.

Verifique a autenticidade deste documento em www.lct.poli.usp.br utilizando o código **DFQF-KTYU-ANUT-WKIB**

DIFRATOGRAMA DE RAIOS X

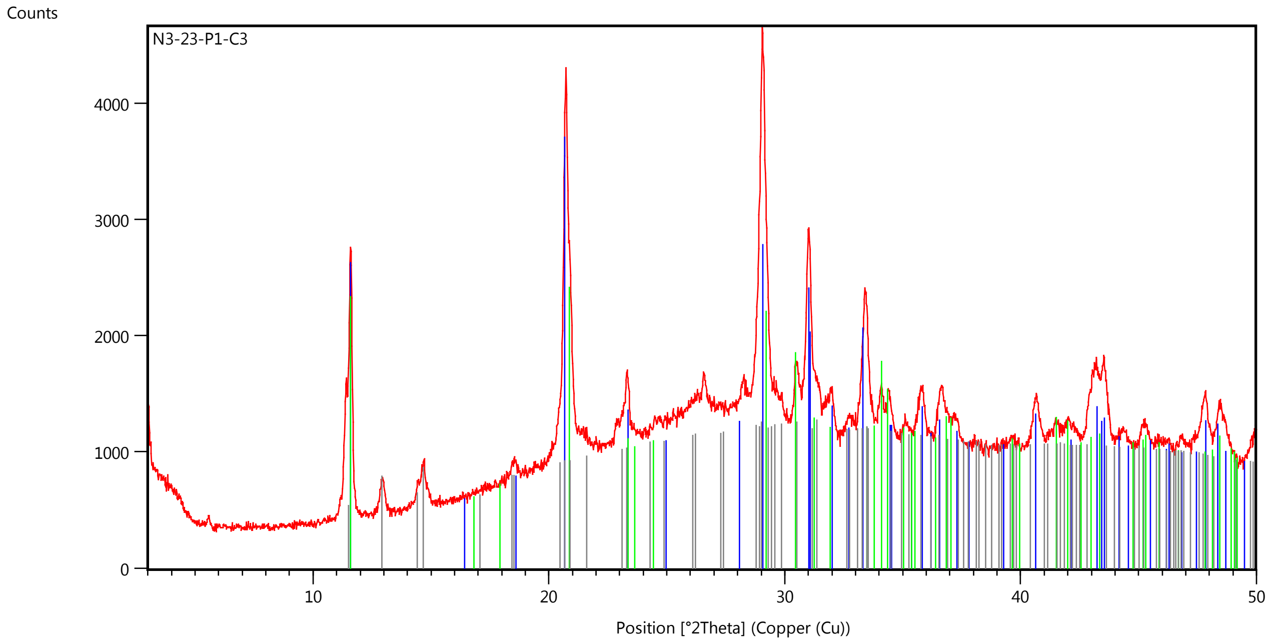

FASES IDENTIFICADAS

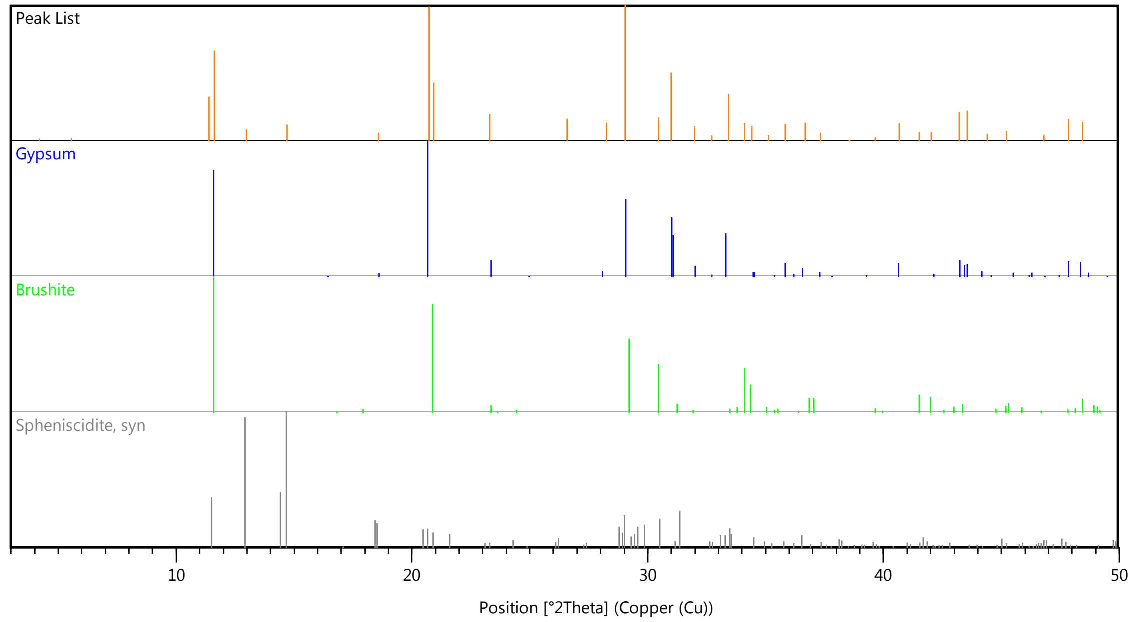

Escola Politécnica da Universidade de São Paulo
Departamento de Engenharia de Minas e de Petróleo
Laboratório de Caracterização Tecnológica
Av. Prof. Mello Moraes, 2373 CEP 05508-030 São Paulo - SP www.lct.poli.usp.br
Tel: 11 3091-5151 e-mail: lct@lct.poli.usp.br

RESULTADO DE IDENTIFICAÇÃO DE FASES POR DIFRATOMETRIA DE RAIOS X

RELATÓRIO: DRX 1211/19 **DATA:** 11/12/2019

CLIENTE: Luís Piló

AMOSTRA: N3-23-P1-C4 **IDENT. LCT:** 511-10046.HPF

1. MÉTODO

O estudo foi efetuado através do método do pó, mediante o emprego de difratômetro de raios X com detector sensível a posição.

A identificação das fases cristalinas, abaixo discriminadas, foi obtida por comparação do difratograma da amostra com os bancos de dados PDF2 do ICDD - International Centre for Diffraction Data e ICSD – Inorganic Crystal Structure Database.

2. RESULTADOS

Os resultados obtidos estão listados na tabela abaixo:

ICDD	Mineral	Fórmula Química	Obs
01-072-0713	Brushita	$CaHPO_4(H_2O)_2$	
01-074-1904	Gipsita	$Ca(SO_4)(H_2O)_2$	
01-082-1164	Spheniscidita	$Fe_2(NH_4)(OH)(PO_4)_2(H_2O)_2$	
	Fase amorfa		pp
01-087-2096	Quartzo	SiO_2	pp

Nota: pp = possível presença

O difratograma obtido (cor vermelha), onde são assinaladas as linhas de difração correspondente(s) à(s) fase(s) identificada(s) (cada fase em uma cor distinta) é apresentado anexo.

Executado por: M.Sc. Gaspar Darin Filho (16/12/2019 18:04 BRT)
Revisado por: Dra. Maria Manuela Tassinari (16/12/2019 18:24 BRT)

Profa. Dra. Carina Ulsen
Coordenadora do LCT - Poli/USP

NOTA: Os resultados expostos acima referem-se apenas à(s) amostra(s) enviada(s) ao LCT; a representatividade da(s) mesma(s) é de inteira responsabilidade do cliente.

Verifique a autenticidade deste documento em www.lct.poli.usp.br utilizando o código **EFQG-WTYU-OFUT-IEIB**

DIFRATOGRAMA DE RAIOS X

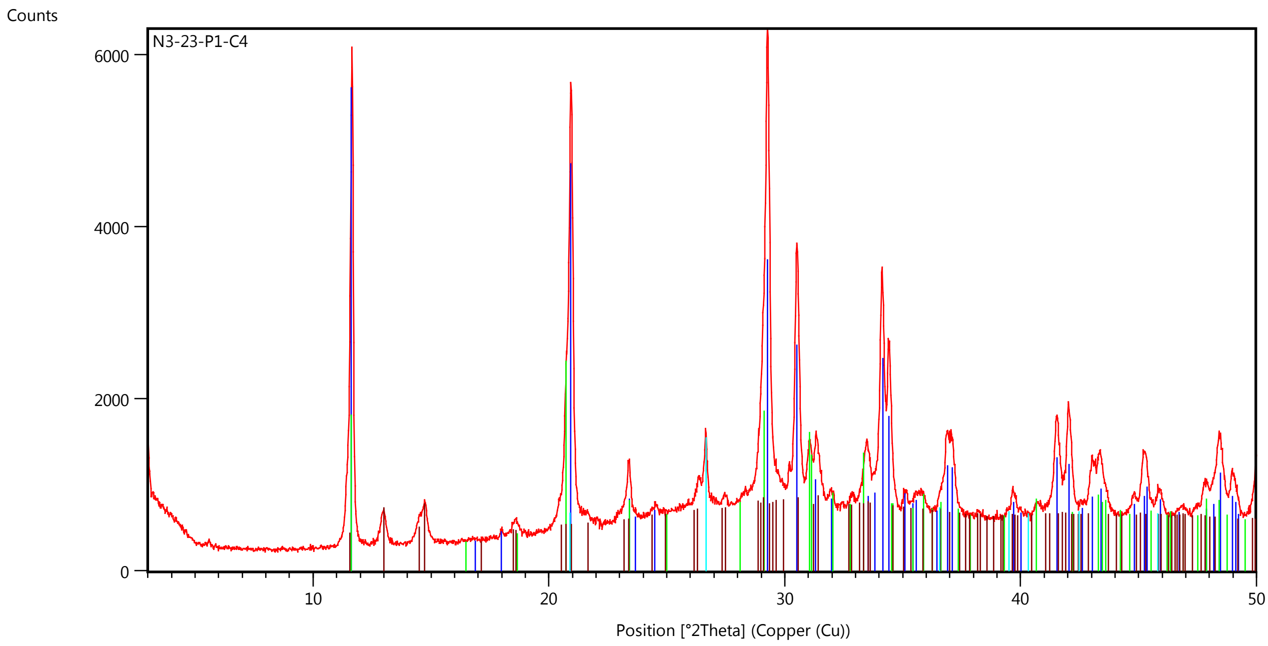

FASES IDENTIFICADAS

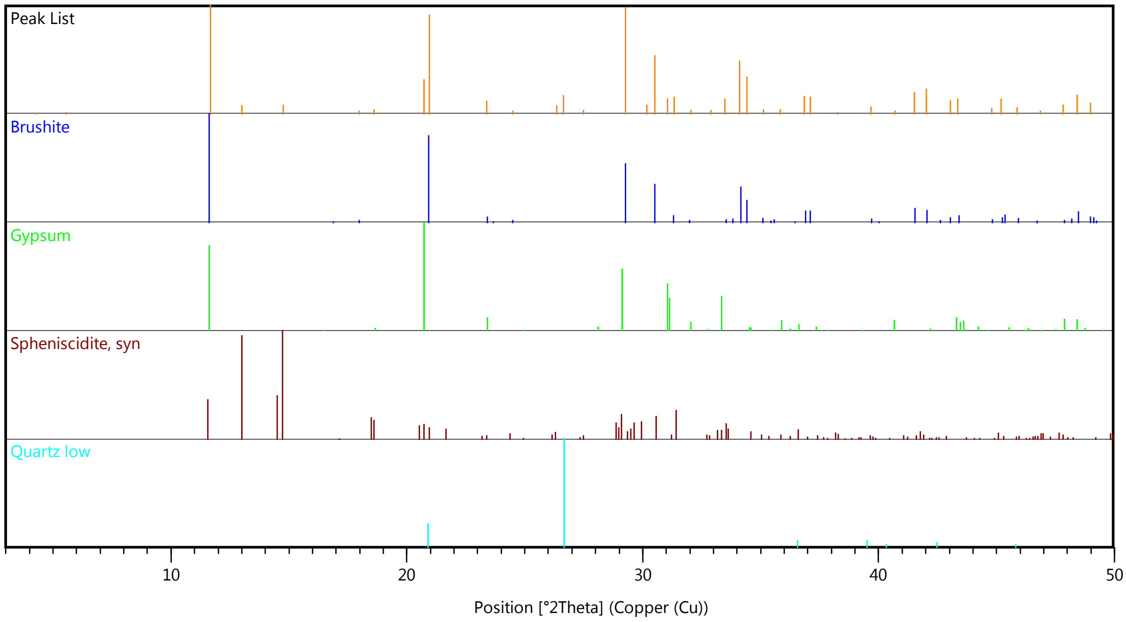

Escola Politécnica da Universidade de São Paulo
Departamento de Engenharia de Minas e de Petróleo
Laboratório de Caracterização Tecnológica
Av. Prof. Mello Moraes, 2373 CEP 05508-030 São Paulo - SP www.lct.poli.usp.br
Tel: 11 3091-5151 e-mail: lct@lct.poli.usp.br

RESULTADO DE IDENTIFICAÇÃO DE FASES POR DIFRATOMETRIA DE RAIOS X

RELATÓRIO: DRX 1212/19 **DATA:** 11/12/2019

CLIENTE: Luís Piló

AMOSTRA: N3-23-P1-C5 **IDENT. LCT:** 511-10047.HPF

1. MÉTODO

O estudo foi efetuado através do método do pó, mediante o emprego de difratômetro de raios X com detector sensível a posição.

A identificação das fases cristalinas, abaixo discriminadas, foi obtida por comparação do difratograma da amostra com os bancos de dados PDF2 do ICDD - International Centre for Diffraction Data e ICSD – Inorganic Crystal Structure Database.

2. RESULTADOS

Os resultados obtidos estão listados na tabela abaixo:

ICDD	Mineral	Fórmula Química	Obs
	Fase amorfa		
01-074-1904	Gipsita	$Ca(SO_4)(H_2O)_2$	
01-082-1164	Spheniscidita	$Fe_2(NH_4)(OH)(PO_4)_2(H_2O)_2$	

O difratograma obtido (cor vermelha), onde são assinaladas as linhas de difração correspondente(s) à(s) fase(s) identificada(s) (cada fase em uma cor distinta) é apresentado anexo.

Executado por: M.Sc. Gaspar Darin Filho (16/12/2019 18:04 BRT)
Revisado por: Dra. Maria Manuela Tassinari (16/12/2019 18:24 BRT)

Profa. Dra. Carina Ulsen
Coordenadora do LCT - Poli/USP

NOTA: Os resultados expostos acima referem-se apenas à(s) amostra(s) enviada(s) ao LCT; a representatividade da(s) mesma(s) é de inteira responsabilidade do cliente.

Verifique a autenticidade deste documento em www.lct.poli.usp.br utilizando o código **UFQH-DTYU-TVUT-WWIB**

DIFRATOGRAMA DE RAIOS X

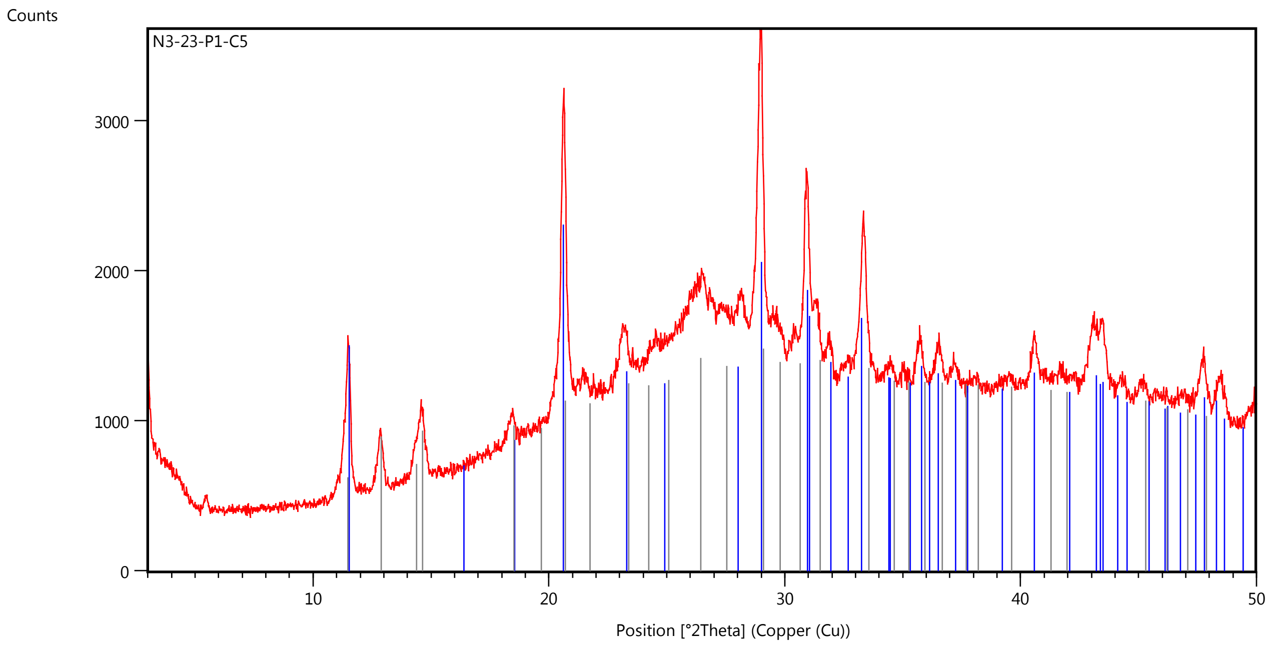

FASES IDENTIFICADAS

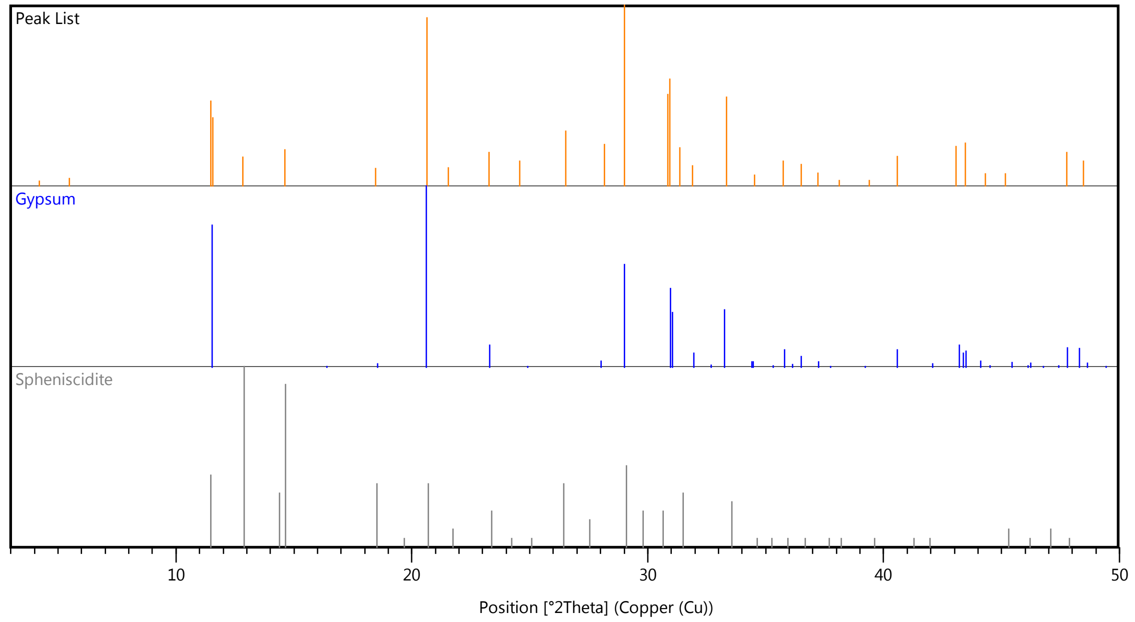

Escola Politécnica da Universidade de São Paulo
Departamento de Engenharia de Minas e de Petróleo
Laboratório de Caracterização Tecnológica
Av. Prof. Mello Moraes, 2373 CEP 05508-030 São Paulo - SP www.lct.poli.usp.br
Tel: 11 3091-5151 e-mail: lct@lct.poli.usp.br

RESULTADO DE IDENTIFICAÇÃO DE FASES POR DIFRATOMETRIA DE RAIOS X

RELATÓRIO: DRX 1213/19 **DATA:** 11/12/2019

CLIENTE: Luís Piló

AMOSTRA: N3-23-P1-C6 **IDENT. LCT:** 511-10048.HPF

1. MÉTODO

O estudo foi efetuado através do método do pó, mediante o emprego de difratômetro de raios X com detector sensível a posição.

A identificação das fases cristalinas, abaixo discriminadas, foi obtida por comparação do difratograma da amostra com os bancos de dados PDF2 do ICDD - International Centre for Diffraction Data e ICSD – Inorganic Crystal Structure Database.

2. RESULTADOS

Os resultados obtidos estão listados na tabela abaixo:

ICDD	Mineral	Fórmula Química	Obs
01-072-0713	Brushita	$CaHPO_4(H_2O)_2$	
01-074-1904	Gipsita	$Ca(SO_4)(H_2O)_2$	
01-082-1164	Spheniscidita	$Fe_2(NH_4)(OH)(PO_4)_2(H_2O)_2$	
	Fase amorfa		pp

Nota: pp = possível presença

O difratograma obtido (cor vermelha), onde são assinaladas as linhas de difração correspondente(s) à(s) fase(s) identificada(s) (cada fase em uma cor distinta) é apresentado anexo.

Executado por: M.Sc. Gaspar Darin Filho (16/12/2019 18:04 BRT)
Revisado por: Dra. Maria Manuela Tassinari (16/12/2019 18:24 BRT)

Profa. Dra. Carina Ulsen
Coordenadora do LCT - Poli/USP

NOTA: Os resultados expostos acima referem-se apenas à(s) amostra(s) enviada(s) ao LCT; a representatividade da(s) mesma(s) é de inteira responsabilidade do cliente.

Verifique a autenticidade deste documento em www.lct.poli.usp.br utilizando o código **NFQI-SUYU-QDUT-AGIB**

DIFRATOGRAMA DE RAIOS X

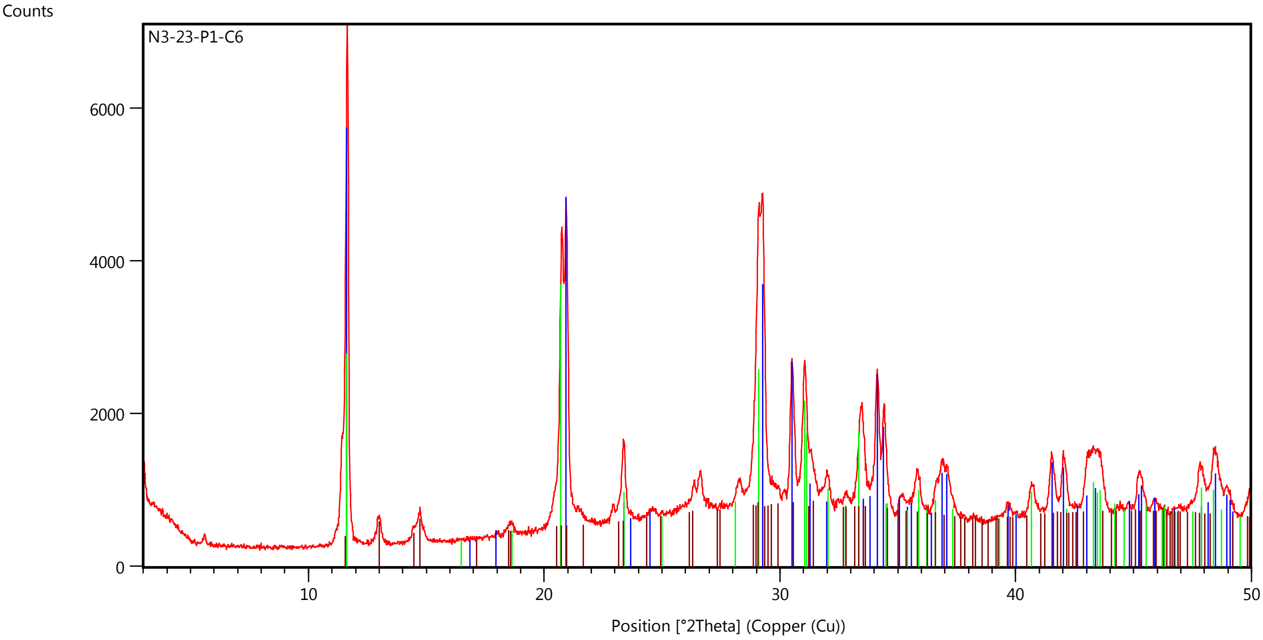

FASES IDENTIFICADAS

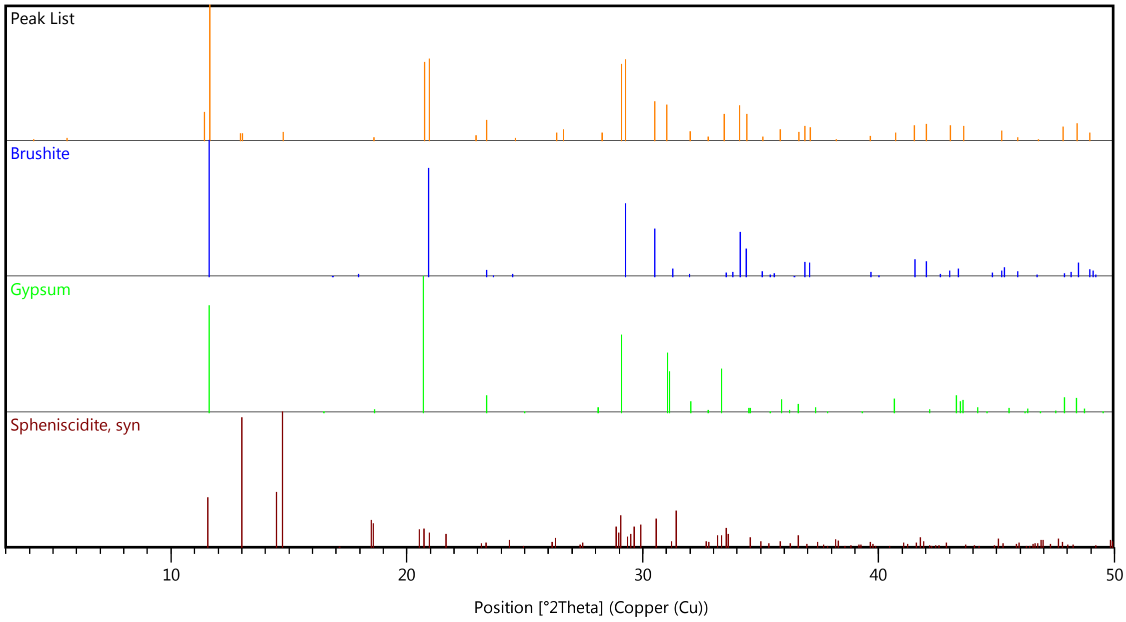

Escola Politécnica da Universidade de São Paulo
Departamento de Engenharia de Minas e de Petróleo
Laboratório de Caracterização Tecnológica
Av. Prof. Mello Moraes, 2373 CEP 05508-030 São Paulo - SP www.lct.poli.usp.br
Tel: 11 3091-5151 e-mail: lct@lct.poli.usp.br

RESULTADO DE IDENTIFICAÇÃO DE FASES POR DIFRATOMETRIA DE RAIOS X

RELATÓRIO: DRX 1214/19 **DATA:** 11/12/2019

CLIENTE: Luís Piló

AMOSTRA: N3-23-P1-C7 **IDENT. LCT:** 511-10049.HPF

1. MÉTODO

O estudo foi efetuado através do método do pó, mediante o emprego de difratômetro de raios X com detector sensível a posição.

A identificação das fases cristalinas, abaixo discriminadas, foi obtida por comparação do difratograma da amostra com os bancos de dados PDF2 do ICDD - International Centre for Diffraction Data e ICSD – Inorganic Crystal Structure Database.

2. RESULTADOS

Os resultados obtidos estão listados na tabela abaixo:

ICDD	Mineral	Fórmula Química	Obs
01-088-0651	Leucofosfita	$K(Fe_2(PO_4)_2(OH)(H_2O))(H_2O)$	
	Fase amorfa		
01-075-0443	Quartzo	SiO_2	

O difratograma obtido (cor vermelha), onde são assinaladas as linhas de difração correspondente(s) à(s) fase(s) identificada(s) (cada fase em uma cor distinta) é apresentado anexo.

Executado por: M.Sc. Gaspar Darin Filho (16/12/2019 18:04 BRT)
Revisado por: Dra. Maria Manuela Tassinari (16/12/2019 18:24 BRT)

Profa. Dra. Carina Ulsen
Coordenadora do LCT - Poli/USP

NOTA: Os resultados expostos acima referem-se apenas à(s) amostra(s) enviada(s) ao LCT; a representatividade da(s) mesma(s) é de inteira responsabilidade do cliente.

Verifique a autenticidade deste documento em www.lct.poli.usp.br utilizando o código **XFQJ-AUYU-VKUT-QMIB**

DIFRATOGRAMA DE RAIOS X

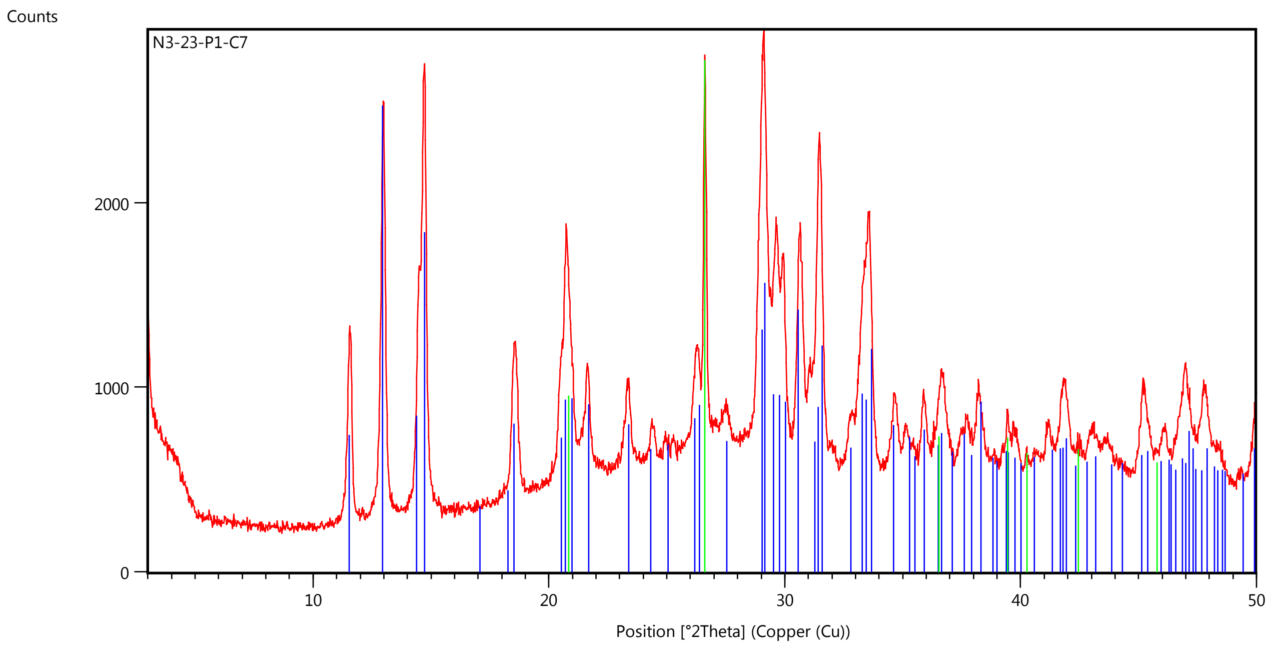

FASES IDENTIFICADAS

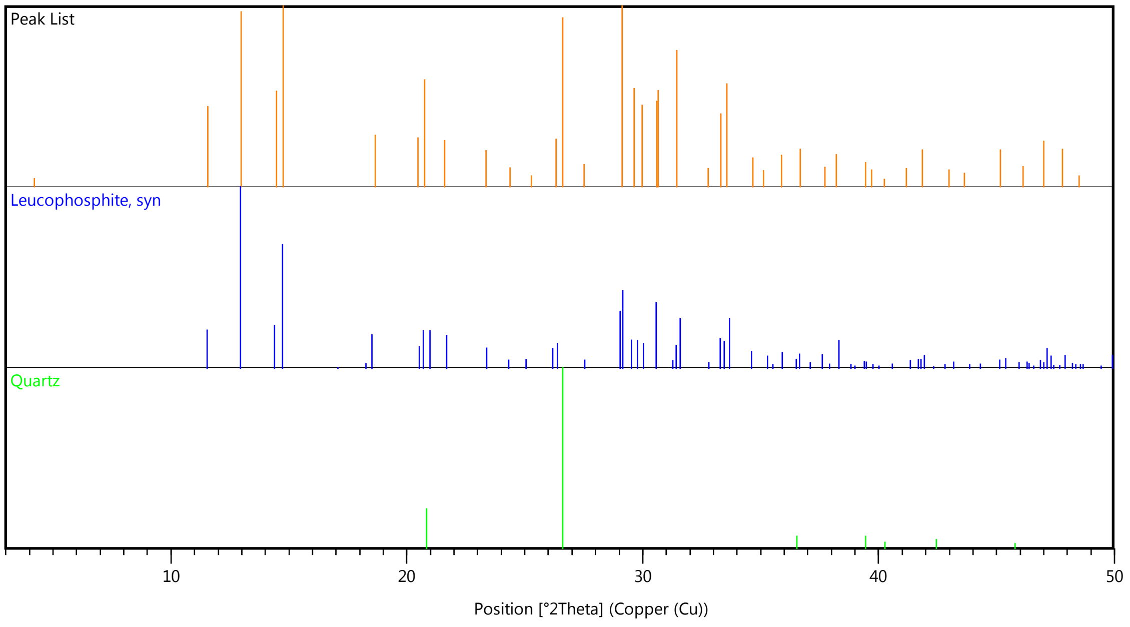

Escola Politécnica da Universidade de São Paulo
Departamento de Engenharia de Minas e de Petróleo
Laboratório de Caracterização Tecnológica
Av. Prof. Mello Moraes, 2373 CEP 05508-030 São Paulo - SP www.lct.poli.usp.br
Tel: 11 3091-5151 e-mail: lct@lct.poli.usp.br

RESULTADO DE IDENTIFICAÇÃO DE FASES POR DIFRATOMETRIA DE RAIOS X

RELATÓRIO: DRX 1215/19 **DATA:** 11/12/2019

CLIENTE: Luís Piló

AMOSTRA: N3-23-P2-C1 **IDENT. LCT:** 511-10050.HPF

1. MÉTODO

O estudo foi efetuado através do método do pó, mediante o emprego de difratômetro de raios X com detector sensível a posição.

A identificação das fases cristalinas, abaixo discriminadas, foi obtida por comparação do difratograma da amostra com os bancos de dados PDF2 do ICDD - International Centre for Diffraction Data e ICSD – Inorganic Crystal Structure Database.

2. RESULTADOS

Os resultados obtidos estão listados na tabela abaixo:

ICDD	Mineral	Fórmula Química	Obs
	Fase amorfa		
01-088-0651	Leucofosfita	$K(Fe_2(PO_4)_2(OH)(H_2O))(H_2O)$	
01-075-0443	Quartzo	SiO_2	

O difratograma obtido (cor vermelha), onde são assinaladas as linhas de difração correspondente(s) à(s) fase(s) identificada(s) (cada fase em uma cor distinta) é apresentado anexo.

Executado por: M.Sc. Gaspar Darin Filho (16/12/2019 18:04 BRT)
Revisado por: Dra. Maria Manuela Tassinari (16/12/2019 18:24 BRT)

Profa. Dra. Carina Ulsen
Coordenadora do LCT - Poli/USP

NOTA: Os resultados expostos acima referem-se apenas à(s) amostra(s) enviada(s) ao LCT; a representatividade da(s) mesma(s) é de inteira responsabilidade do cliente.

Verifique a autenticidade deste documento em www.lct.poli.usp.br utilizando o código **CFQK-PUYU-ORUT-SQIB**

DIFRATOGRAMA DE RAIOS X

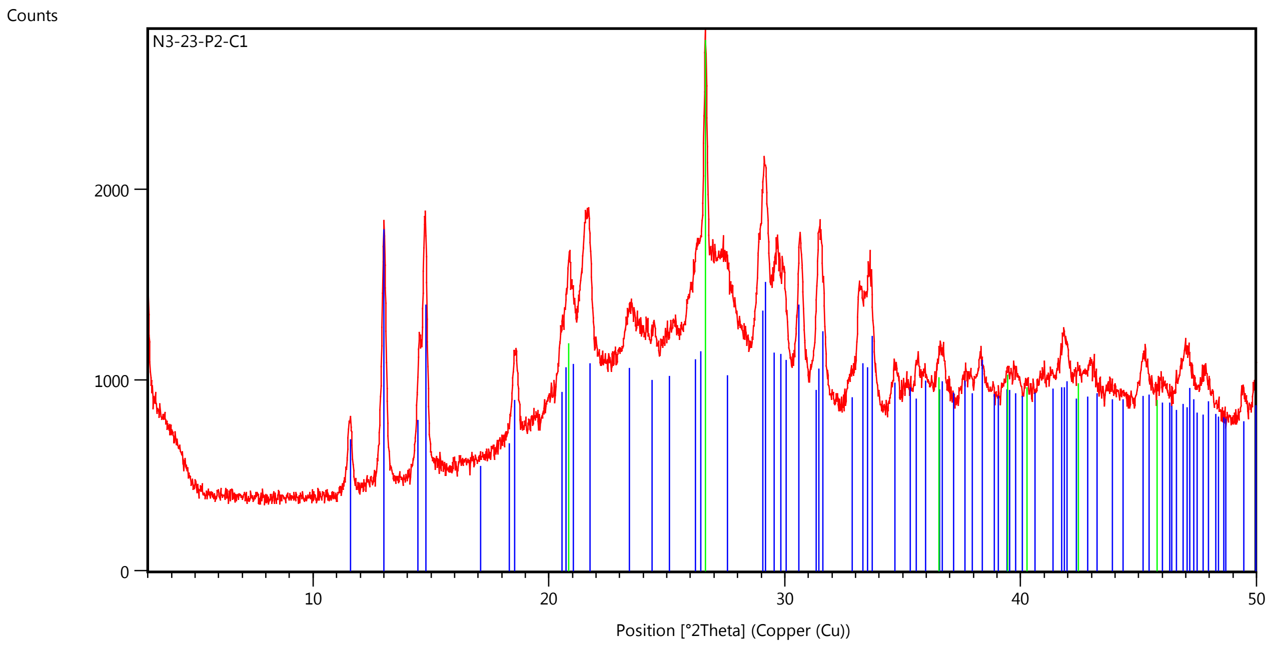

FASES IDENTIFICADAS

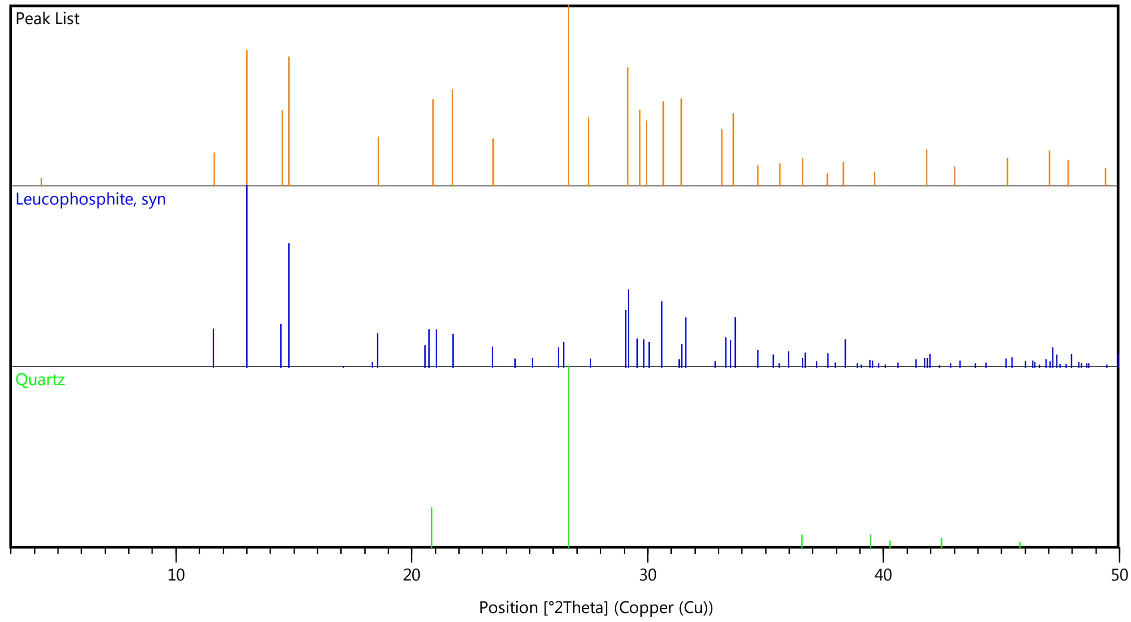

Escola Politécnica da Universidade de São Paulo
Departamento de Engenharia de Minas e de Petróleo
Laboratório de Caracterização Tecnológica
Av. Prof. Mello Moraes, 2373 CEP 05508-030 São Paulo - SP www.lct.poli.usp.br
Tel: 11 3091-5151 e-mail: lct@lct.poli.usp.br

RESULTADO DE IDENTIFICAÇÃO DE FASES POR DIFRATOMETRIA DE RAIOS X

RELATÓRIO: DRX 1216/19 **DATA:** 11/12/2019

CLIENTE: Luís Piló

AMOSTRA: N3-23-P2-C2 **IDENT. LCT:** 511-10051.HPF

1. MÉTODO

O estudo foi efetuado através do método do pó, mediante o emprego de difratômetro de raios X com detector sensível a posição.

A identificação das fases cristalinas, abaixo discriminadas, foi obtida por comparação do difratograma da amostra com os bancos de dados PDF2 do ICDD - International Centre for Diffraction Data e ICSD – Inorganic Crystal Structure Database.

2. RESULTADOS

Os resultados obtidos estão listados na tabela abaixo:

ICDD	Mineral	Fórmula Química	Obs
01-088-0651	Leucofosfita	$K(Fe_2(PO_4)_2(OH)(H_2O))(H_2O)$	
	Fase amorfa		
01-075-0443	Quartzo	SiO_2	

O difratograma obtido (cor vermelha), onde são assinaladas as linhas de difração correspondente(s) à(s) fase(s) identificada(s) (cada fase em uma cor distinta) é apresentado anexo.

Executado por: M.Sc. Gaspar Darin Filho (16/12/2019 18:04 BRT)
Revisado por: Dra. Maria Manuela Tassinari (16/12/2019 18:24 BRT)

Profa. Dra. Carina Ulsen
Coordenadora do LCT - Poli/USP

NOTA: Os resultados expostos acima referem-se apenas à(s) amostra(s) enviada(s) ao LCT; a representatividade da(s) mesma(s) é de inteira responsabilidade do cliente.

Verifique a autenticidade deste documento em www.lct.poli.usp.br utilizando o código **YFQL-ZUYU-QYUT-WVIB**

Escola Politécnica da Universidade de São Paulo
Departamento de Engenharia de Minas e de Petróleo
Laboratório de Caracterização Tecnológica
Av. Prof. Mello Moraes, 2373 CEP 05508-030 São Paulo - SP www.lct.poli.usp.br
Tel: 11 3091-5151 e-mail: lct@lct.poli.usp.br

DIFRATOGRAMA DE RAIOS X

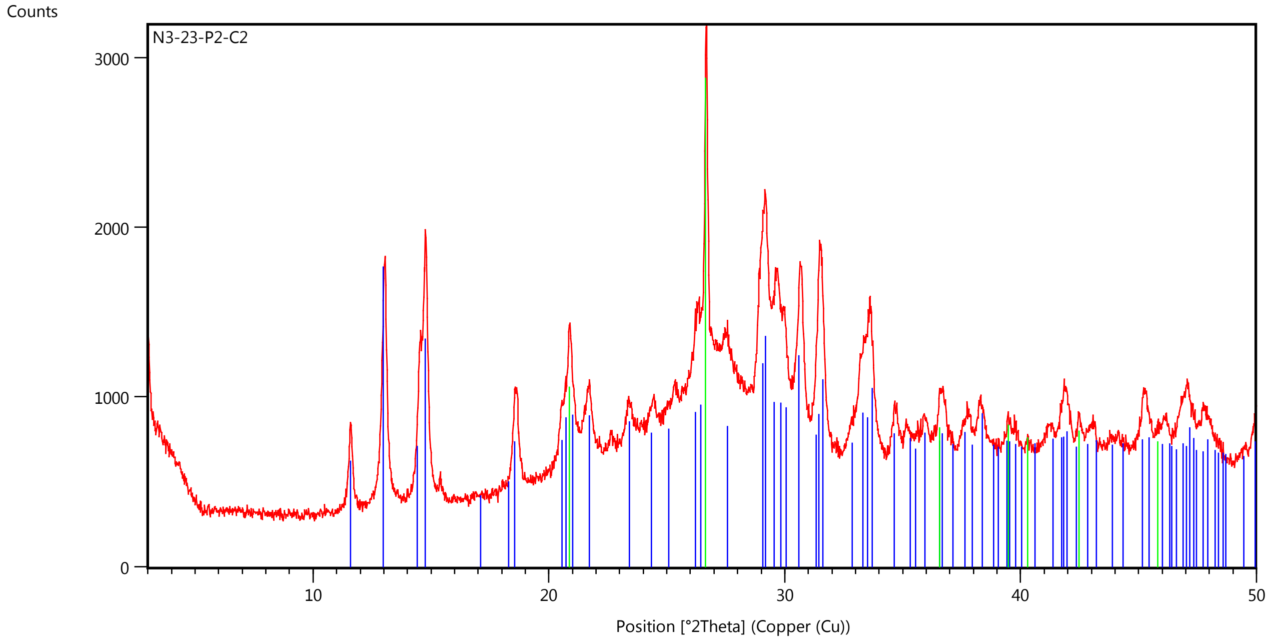

FASES IDENTIFICADAS

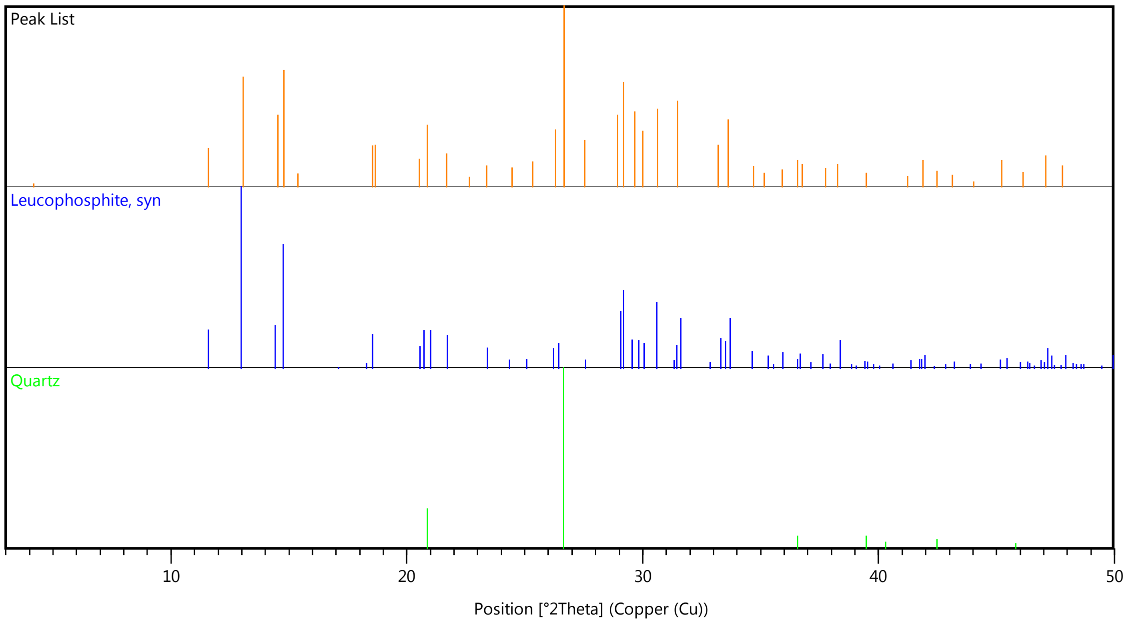

Escola Politécnica da Universidade de São Paulo
Departamento de Engenharia de Minas e de Petróleo
Laboratório de Caracterização Tecnológica
Av. Prof. Mello Moraes, 2373 CEP 05508-030 São Paulo - SP www.lct.poli.usp.br
Tel: 11 3091-5151 e-mail: lct@lct.poli.usp.br

RESULTADO DE IDENTIFICAÇÃO DE FASES POR DIFRATOMETRIA DE RAIOS X

RELATÓRIO: DRX 1217/19 **DATA:** 11/12/2019

CLIENTE: Luís Piló

AMOSTRA: N3-23-P2-C3 **IDENT. LCT:** 511-10052.HPF

1. MÉTODO

O estudo foi efetuado através do método do pó, mediante o emprego de difratômetro de raios X com detector sensível a posição.

A identificação das fases cristalinas, abaixo discriminadas, foi obtida por comparação do difratograma da amostra com os bancos de dados PDF2 do ICDD - International Centre for Diffraction Data e ICSD – Inorganic Crystal Structure Database.

2. RESULTADOS

Os resultados obtidos estão listados na tabela abaixo:

ICDD	Mineral	Fórmula Química	Obs
01-088-0651	Leucofosfita	$K(Fe_2(PO_4)_2(OH)(H_2O))(H_2O)$	
	Fase amorfa		
01-075-0443	Quartzo	SiO_2	

O difratograma obtido (cor vermelha), onde são assinaladas as linhas de difração correspondente(s) à(s) fase(s) identificada(s) (cada fase em uma cor distinta) é apresentado anexo.

Executado por: M.Sc. Gaspar Darin Filho (16/12/2019 18:04 BRT)
Revisado por: Dra. Maria Manuela Tassinari (16/12/2019 18:24 BRT)

Profa. Dra. Carina Ulsen
Coordenadora do LCT - Poli/USP

NOTA: Os resultados expostos acima referem-se apenas à(s) amostra(s) enviada(s) ao LCT; a representatividade da(s) mesma(s) é de inteira responsabilidade do cliente.

Verifique a autenticidade deste documento em www.lct.poli.usp.br utilizando o código **RFQM-FVYU-BGUT-YDIB**

DIFRATOGRAMA DE RAIOS X

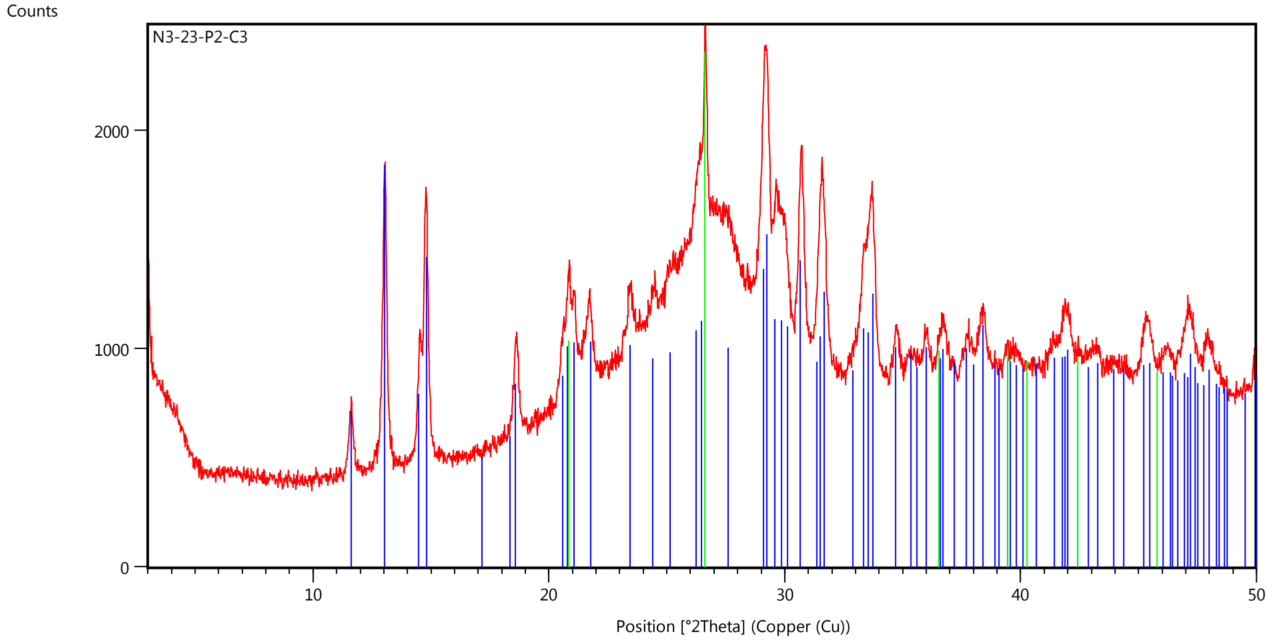

FASES IDENTIFICADAS

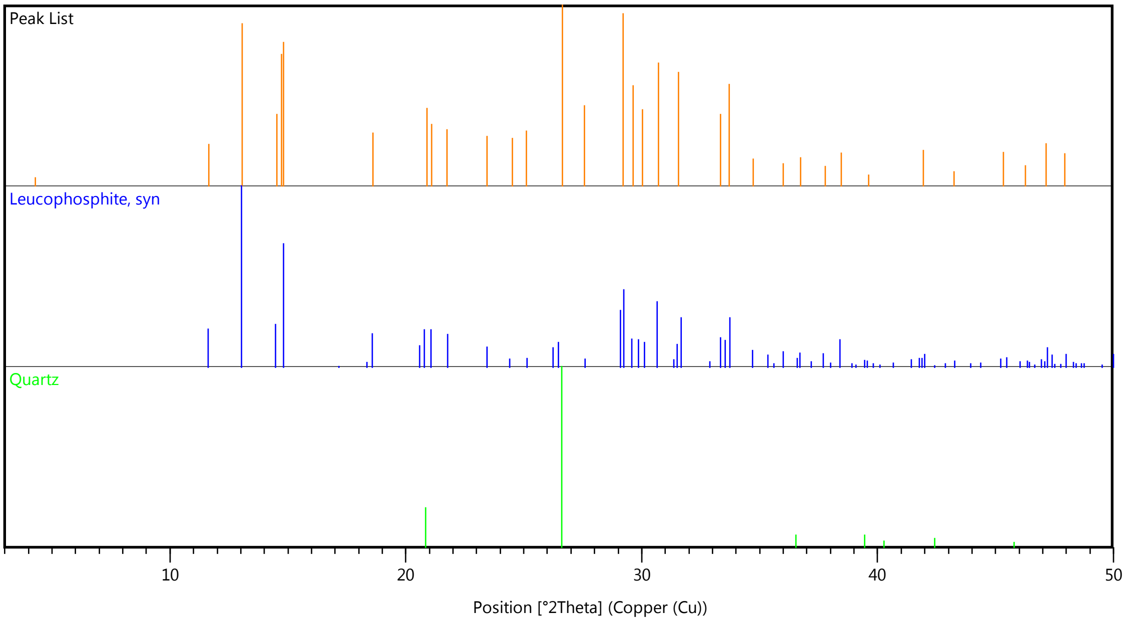

Escola Politécnica da Universidade de São Paulo
Departamento de Engenharia de Minas e de Petróleo
Laboratório de Caracterização Tecnológica
Av. Prof. Mello Moraes, 2373 CEP 05508-030 São Paulo - SP www.lct.poli.usp.br
Tel: 11 3091-5151 e-mail: lct@lct.poli.usp.br

RESULTADO DE IDENTIFICAÇÃO DE FASES POR DIFRATOMETRIA DE RAIOS X

RELATÓRIO: DRX 1222/19 **DATA:** 11/12/2019

CLIENTE: Luís Piló

AMOSTRA: S11B-94-C1 **IDENT. LCT:** 511-10057.HPF

1. MÉTODO

O estudo foi efetuado através do método do pó, mediante o emprego de difratômetro de raios X com detector sensível a posição.

A identificação das fases cristalinas, abaixo discriminadas, foi obtida por comparação do difratograma da amostra com os bancos de dados PDF2 do ICDD - International Centre for Diffraction Data e ICSD – Inorganic Crystal Structure Database.

2. RESULTADOS

Os resultados obtidos estão listados na tabela abaixo:

ICDD	Mineral	Fórmula Química	Obs
01-088-0651	Leucofosfita	$K(Fe_2(PO_4)_2(OH)(H_2O))(H_2O)$	
01-086-1628	Quartzo	SiO_2	
01-085-0882	Bifosammita	$NH_4H_2PO_4$	pp

Nota: pp = possível presença

O difratograma obtido (cor vermelha), onde são assinaladas as linhas de difração correspondente(s) à(s) fase(s) identificada(s) (cada fase em uma cor distinta) é apresentado anexo.

Executado por: M.Sc. Gaspar Darin Filho (16/12/2019 18:04 BRT)
Revisado por: Dra. Maria Manuela Tassinari (16/12/2019 18:24 BRT)

Profa. Dra. Carina Ulsen
Coordenadora do LCT - Poli/USP

NOTA: Os resultados expostos acima referem-se apenas à(s) amostra(s) enviada(s) ao LCT; a representatividade da(s) mesma(s) é de inteira responsabilidade do cliente.

Verifique a autenticidade deste documento em www.lct.poli.usp.br utilizando o código **GFQR-XWYU-TSUT-GHIB**

DIFRATOGRAMA DE RAIOS X

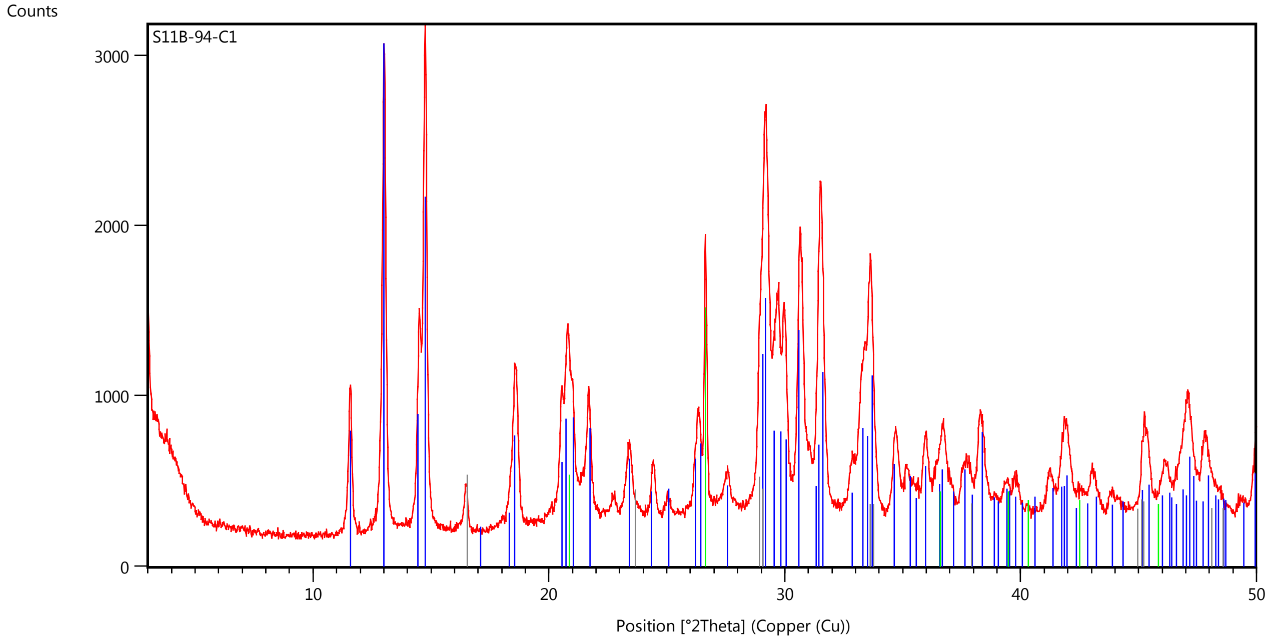

FASES IDENTIFICADAS

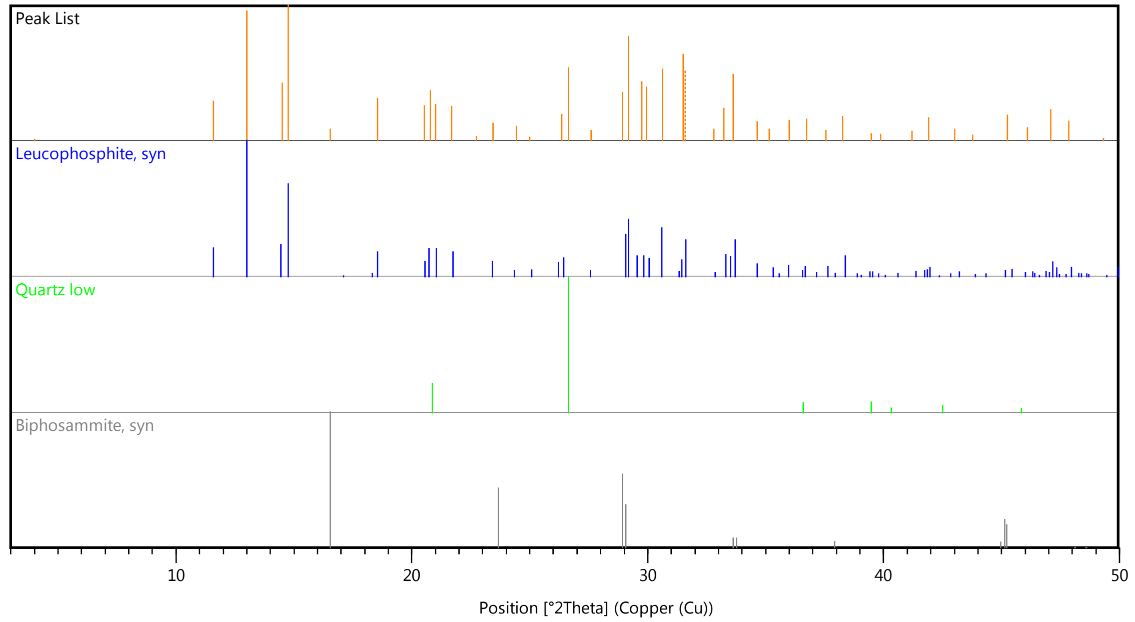

Escola Politécnica da Universidade de São Paulo
Departamento de Engenharia de Minas e de Petróleo
Laboratório de Caracterização Tecnológica
Av. Prof. Mello Moraes, 2373 CEP 05508-030 São Paulo - SP www.lct.poli.usp.br
Tel: 11 3091-5151 e-mail: lct@lct.poli.usp.br

RESULTADO DE IDENTIFICAÇÃO DE FASES POR DIFRATOMETRIA DE RAIOS X

RELATÓRIO: DRX 1223/19 **DATA:** 11/12/2019

CLIENTE: Luís Piló

AMOSTRA: S11B-94-C2 **IDENT. LCT:** 511-10058.HPF

1. MÉTODO

O estudo foi efetuado através do método do pó, mediante o emprego de difratômetro de raios X com detector sensível a posição.

A identificação das fases cristalinas, abaixo discriminadas, foi obtida por comparação do difratograma da amostra com os bancos de dados PDF2 do ICDD - International Centre for Diffraction Data e ICSD – Inorganic Crystal Structure Database.

2. RESULTADOS

Os resultados obtidos estão listados na tabela abaixo:

ICDD	Mineral	Fórmula Química	Obs
00-029-0981	Taranakita	$H_6K_3Al_5(PO_4)_8$ $18H_2O$	
00-033-0033	Variscita	$AlPO_4$ $2H_2O$	

O difratograma obtido (cor vermelha), onde são assinaladas as linhas de difração correspondente(s) à(s) fase(s) identificada(s) (cada fase em uma cor distinta) é apresentado anexo.

Executado por: M.Sc. Gaspar Darin Filho (16/12/2019 18:04 BRT)
Revisado por: Dra. Maria Manuela Tassinari (16/12/2019 18:24 BRT)

Profa. Dra. Carina Ulsen
Coordenadora do LCT - Poli/USP

NOTA: Os resultados expostos acima referem-se apenas à(s) amostra(s) enviada(s) ao LCT; a representatividade da(s) mesma(s) é de inteira responsabilidade do cliente.

Verifique a autenticidade deste documento em www.lct.poli.usp.br utilizando o código **TFQS-GXYU-UDUT-KVIB**

DIFRATOGRAMA DE RAIOS X

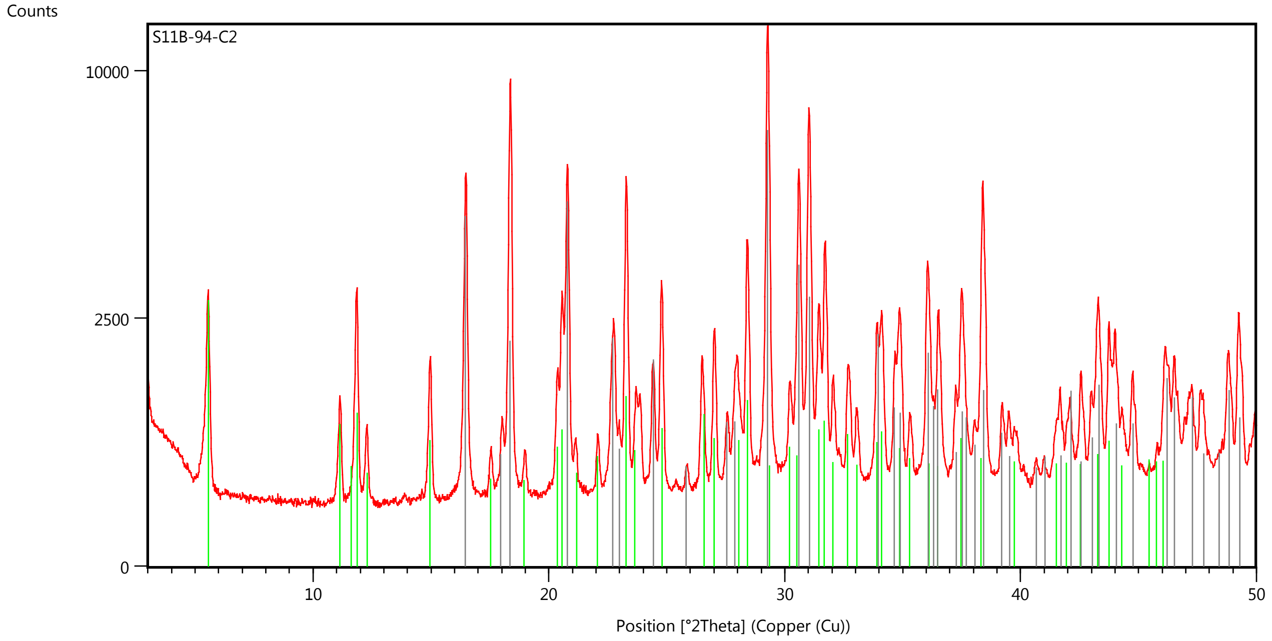

FASES IDENTIFICADAS

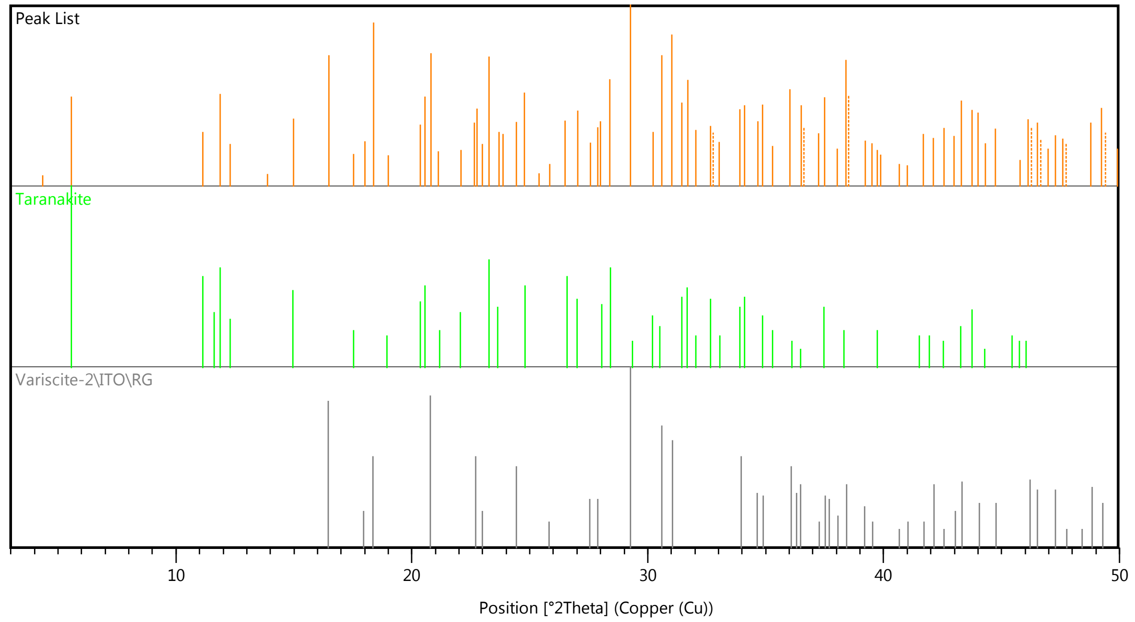

Escola Politécnica da Universidade de São Paulo
Departamento de Engenharia de Minas e de Petróleo
Laboratório de Caracterização Tecnológica
Av. Prof. Mello Moraes, 2373 CEP 05508-030 São Paulo - SP www.lct.poli.usp.br
Tel: 11 3091-5151 e-mail: lct@lct.poli.usp.br

RESULTADO DE IDENTIFICAÇÃO DE FASES POR DIFRATOMETRIA DE RAIOS X

RELATÓRIO: DRX 1224/19 **DATA:** 11/12/2019

CLIENTE: Luís Piló

AMOSTRA: S11B-94-C3 **IDENT. LCT:** 511-10059.HPF

1. MÉTODO

O estudo foi efetuado através do método do pó, mediante o emprego de difratômetro de raios X com detector sensível a posição.

A identificação das fases cristalinas, abaixo discriminadas, foi obtida por comparação do difratograma da amostra com os bancos de dados PDF2 do ICDD - International Centre for Diffraction Data e ICSD – Inorganic Crystal Structure Database.

2. RESULTADOS

Os resultados obtidos estão listados na tabela abaixo:

ICDD	Mineral	Fórmula Química	Obs
00-033-0033	Variscita	$AlPO_4$ $2H_2O$	

O difratograma obtido (cor vermelha), onde são assinaladas as linhas de difração correspondente(s) à(s) fase(s) identificada(s) (cada fase em uma cor distinta) é apresentado anexo.

Executado por: M.Sc. Gaspar Darin Filho (16/12/2019 18:04 BRT)
Revisado por: Dra. Maria Manuela Tassinari (16/12/2019 18:24 BRT)

Profa. Dra. Carina Ulsen
Coordenadora do LCT - Poli/USP

NOTA: Os resultados expostos acima referem-se apenas à(s) amostra(s) enviada(s) ao LCT; a representatividade da(s) mesma(s) é de inteira responsabilidade do cliente.

Verifique a autenticidade deste documento em www.lct.poli.usp.br utilizando o código **PFQT-IXYU-OMUT-CBIB**

DIFRATOGRAMA DE RAIOS X

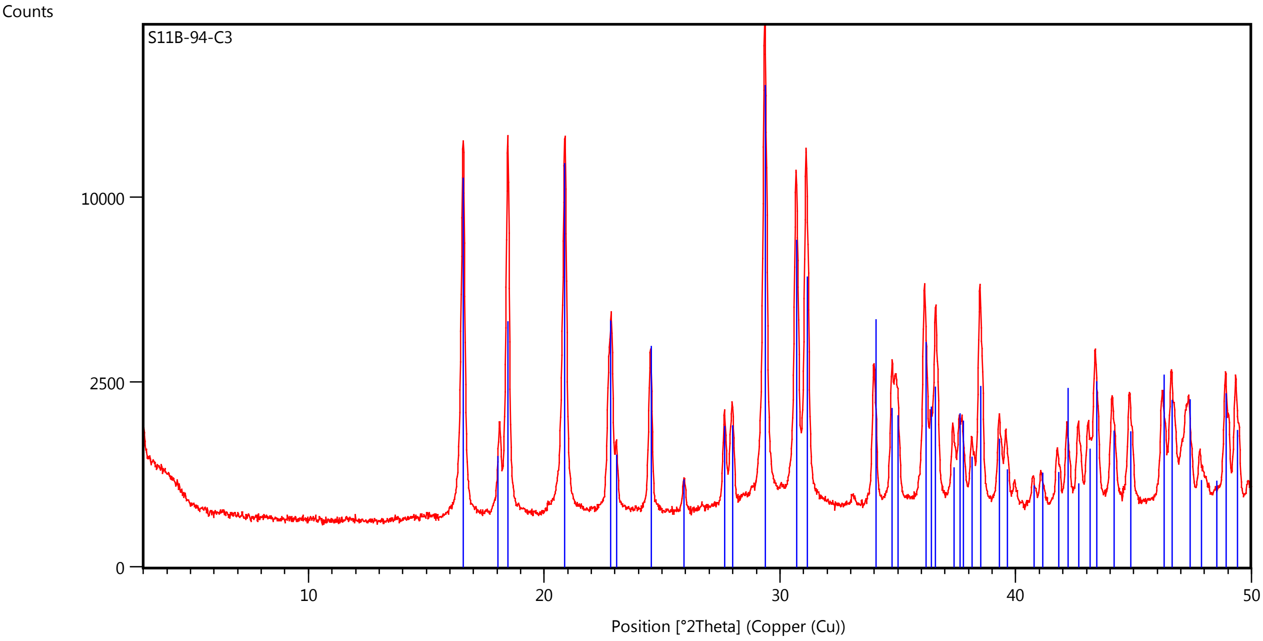

FASES IDENTIFICADAS

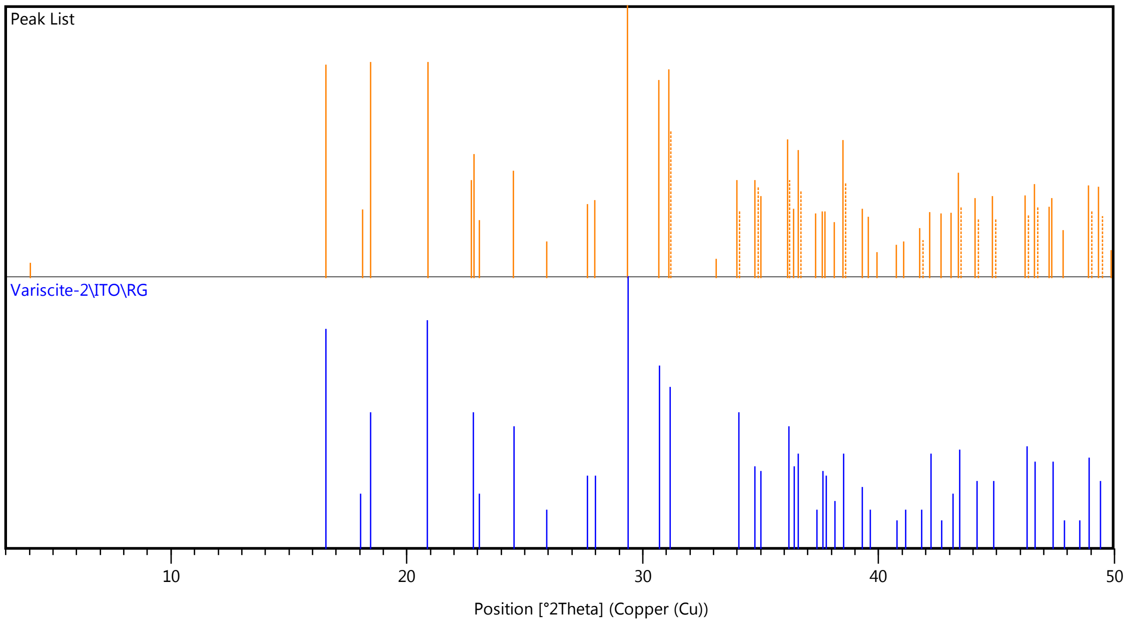

Escola Politécnica da Universidade de São Paulo
Departamento de Engenharia de Minas e de Petróleo
Laboratório de Caracterização Tecnológica
Av. Prof. Mello Moraes, 2373 CEP 05508-030 São Paulo - SP www.lct.poli.usp.br
Tel: 11 3091-5151 e-mail: lct@lct.poli.usp.br

RESULTADO DE IDENTIFICAÇÃO DE FASES POR DIFRATOMETRIA DE RAIOS X

RELATÓRIO: DRX 1225/19 **DATA:** 11/12/2019

CLIENTE: Luís Piló

AMOSTRA: S11B-94-C5 **IDENT. LCT:** 511-10060.HPF

1. MÉTODO

O estudo foi efetuado através do método do pó, mediante o emprego de difratômetro de raios X com detector sensível a posição.

A identificação das fases cristalinas, abaixo discriminadas, foi obtida por comparação do difratograma da amostra com os bancos de dados PDF2 do ICDD - International Centre for Diffraction Data e ICSD – Inorganic Crystal Structure Database.

2. RESULTADOS

Os resultados obtidos estão listados na tabela abaixo:

ICDD	Mineral	Fórmula Química	Obs
01-088-0651	Leucofosfita	$K(Fe_2(PO_4)_2(OH)(H_2O))(H_2O)$	
00-033-0033	Variscita	$AlPO_4$ $2H_2O$	pp

Nota: pp = possível presença

O difratograma obtido (cor vermelha), onde são assinaladas as linhas de difração correspondente(s) à(s) fase(s) identificada(s) (cada fase em uma cor distinta) é apresentado anexo.

Executado por: M.Sc. Gaspar Darin Filho (16/12/2019 18:04 BRT)
Revisado por: Dra. Maria Manuela Tassinari (16/12/2019 18:24 BRT)

Profa. Dra. Carina Ulsen
Coordenadora do LCT - Poli/USP

NOTA: Os resultados expostos acima referem-se apenas à(s) amostra(s) enviada(s) ao LCT; a representatividade da(s) mesma(s) é de inteira responsabilidade do cliente.

Verifique a autenticidade deste documento em www.lct.poli.usp.br utilizando o código **UFQU-QXYU-STUT-WJIB**

DIFRATOGRAMA DE RAIOS X

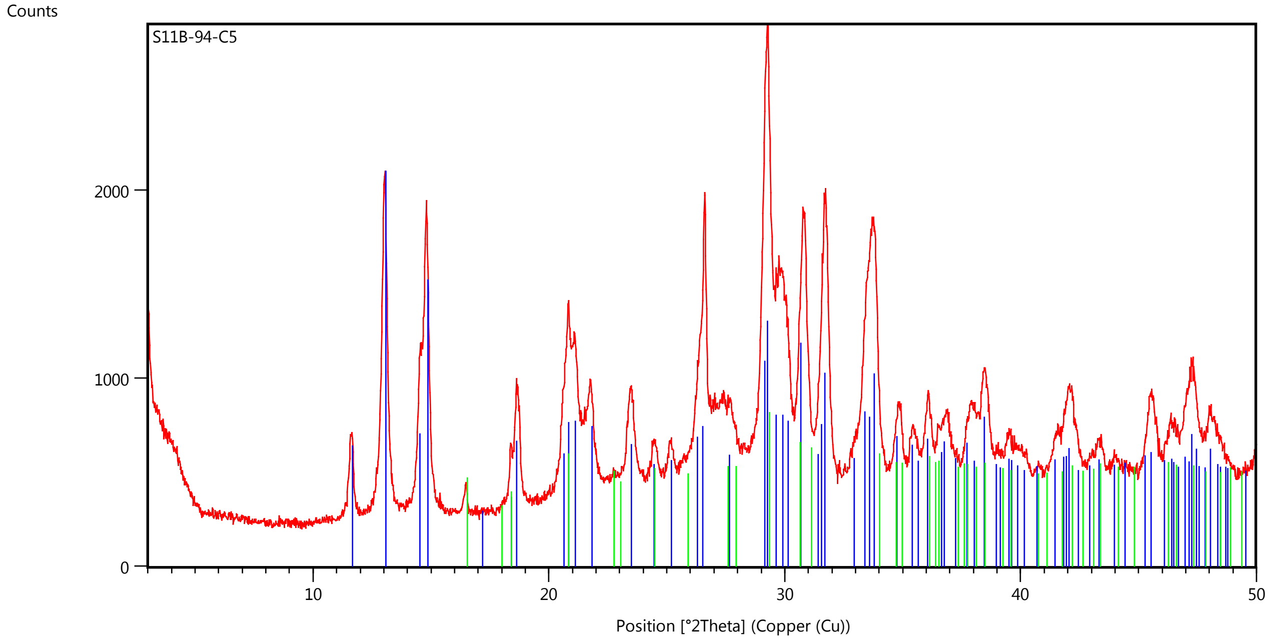

FASES IDENTIFICADAS

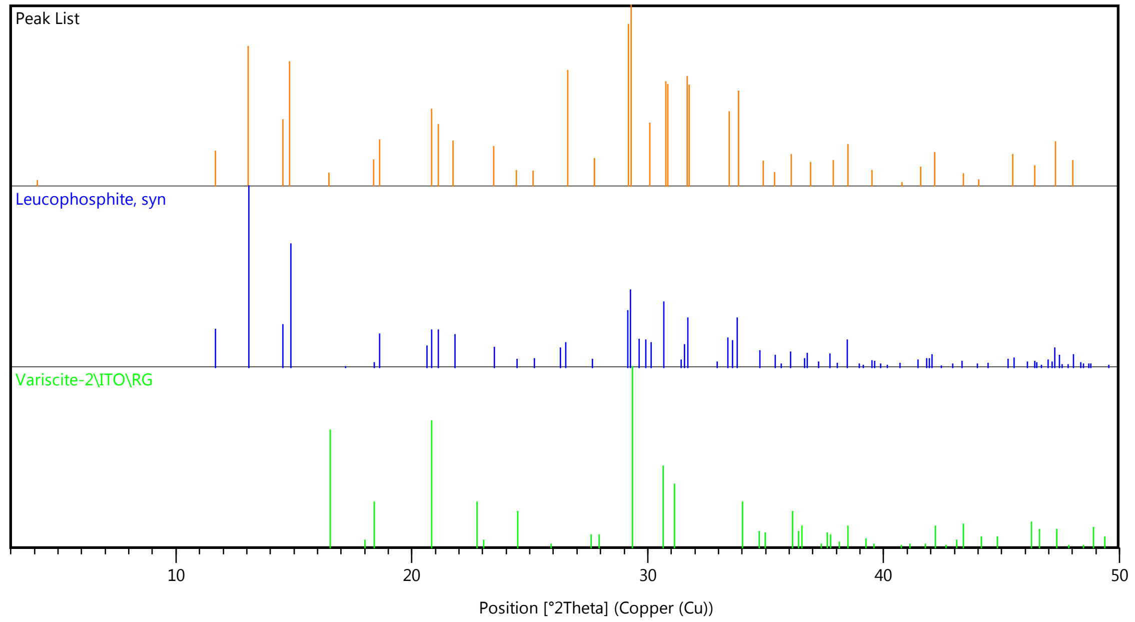

Supplement: S2 File — Reports issued by the Laboratório de Caracterização Tecnológica, Departamento de Engenharia de Minas e de Petróleo at the University of São Paulo´s Escola Politécnica, indicating mineral identification of guano samples using the powder method and a Panalytical X-ray diffractometer. (PDF) [file pone.0267870.s006.pdf]
