# Supplementary material for: Bats as ecosystem engineers in iron ore caves in the Carajás National Forest, Brazilian Amazonia
Source: PLoS One. 2023 May 11;18(5):e0267870. doi: 10.1371/journal.pone.0267870 (PMC10174506; doi:10.1371/journal.pone.0267870)

Escola Politécnica da Universidade de São Paulo
Departamento de Engenharia de Minas e de Petróleo
Laboratório de Caracterização Tecnológica
Av. Prof. Mello Moraes, 2373 CEP 05508-030 São Paulo - SP www.lct.poli.usp.br
Tel: 11 3091-5151 e-mail: lct@lct.poli.usp.br

RESULTADO DE IDENTIFICAÇÃO DE FASES POR DIFRATOMETRIA DE RAIOS X

RELATÓRIO: DRX 607/19 **DATA:** 12/04/19

CLIENTE: Luís Piló

AMOSTRA: N3-23-TITE **IDENT. LCT:** 130-2094.HPF

1. MÉTODO

O estudo foi efetuado através do método do pó, mediante o emprego de difratômetro de raios X com detector sensível a posição.

A identificação das fases cristalinas, abaixo discriminadas, foi obtida por comparação do difratograma da amostra com os bancos de dados PDF2 do ICDD - International Centre for Diffraction Data e ICSD – Inorganic Crystal Structure Database.

2. RESULTADOS

Os resultados obtidos estão listados na tabela abaixo:

ICDD	Mineral/Composto	Fórmula Química	Obs
98-001-7872	Spheniscidita	$(NH_4)(Fe_2(PO_4)_2(OH)(H_2O))(H_2O)$	e/ou
01-088-0651	Leucofosfita	$K(Fe_2(PO_4)_2(OH)(H_2O))(H_2O)$	

O difratograma obtido (cor vermelha), onde são assinaladas as linhas de difração correspondente(s) à(s) fase(s) identificada(s) (cada fase em uma cor distinta) é apresentado anexo.

Profa. Dra. Carina Ulsen — Coordenadora do LCT
Dra. Maria Manuela Tassinari — Pesquisadora sênior
Dra. Juliana Lívi Antoniassi — Pesquisadora

Verifique a autenticidade deste documento em www.lct.poli.usp.br utilizando o código **YKQA-VJJA-EXTA-KHXB**

Av. Prof. Mello Moraes, 2373 CEP 05508-030 São Paulo - SP www.lct.poli.usp.br
Tel: 11 3091-5151 e-mail: lct@lct.poli.usp.br

DIFRATOGRAMA DE RAIOS X

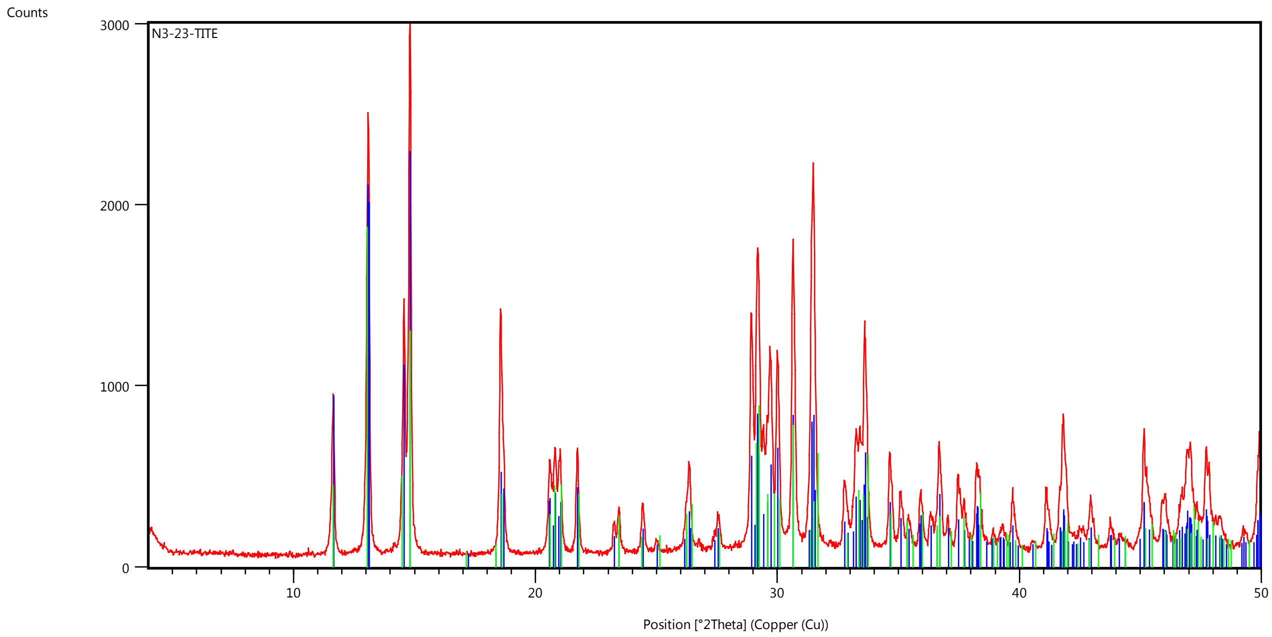

FASES IDENTIFICADAS

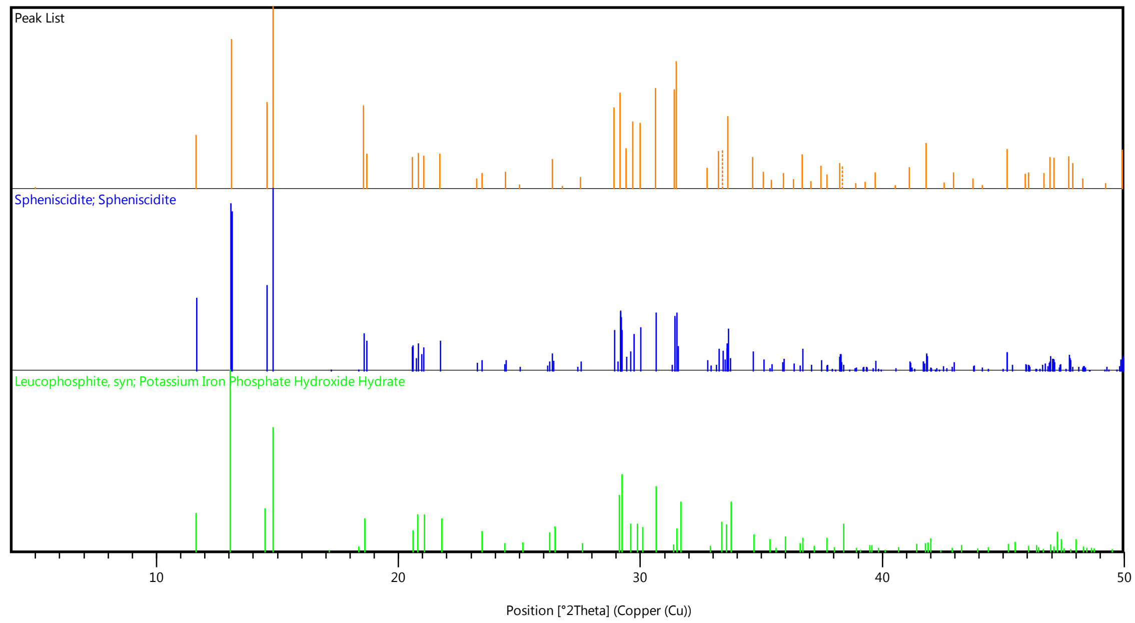

Escola Politécnica da Universidade de São Paulo
Departamento de Engenharia de Minas e de Petróleo
Laboratório de Caracterização Tecnológica
Av. Prof. Mello Moraes, 2373 CEP 05508-030 São Paulo - SP www.lct.poli.usp.br
Tel: 11 3091-5151 e-mail: lct@lct.poli.usp.br

RESULTADO DE IDENTIFICAÇÃO DE FASES POR DIFRATOMETRIA DE RAIOS X

RELATÓRIO: DRX 608/19 **DATA:** 12/04/19

CLIENTE: Luís Piló

AMOSTRA: N3-74-TITE **IDENT. LCT:** 130-2095.HPF

1. MÉTODO

O estudo foi efetuado através do método do pó, mediante o emprego de difratômetro de raios X com detector sensível a posição.

A identificação das fases cristalinas, abaixo discriminadas, foi obtida por comparação do difratograma da amostra com os bancos de dados PDF2 do ICDD - International Centre for Diffraction Data e ICSD – Inorganic Crystal Structure Database.

2. RESULTADOS

Os resultados obtidos estão listados na tabela abaixo:

ICDD	Mineral/Composto	Fórmula Química	Obs
00-033-0667	Strengita	$FePO_4$ $2H_2O$	
01-072-0471	Fosfosiderita	$FePO_4(H_2O)_2$	

O difratograma obtido (cor vermelha), onde são assinaladas as linhas de difração correspondente(s) à(s) fase(s) identificada(s) (cada fase em uma cor distinta) é apresentado anexo.

Profa. Dra. Carina Ulsen
Coordenadora do LCT

Dra. Maria Manuela Tassinari
Pesquisadora sênior

Dra. Juliana Lívi Antoniassi
Pesquisadora

Verifique a autenticidade deste documento em www.lct.poli.usp.br utilizando o código VKQA-CKJA-XETA-CKXB

Escola Politécnica da Universidade de São Paulo
Departamento de Engenharia de Minas e de Petróleo
Laboratório de Caracterização Tecnológica
Av. Prof. Mello Moraes, 2373 CEP 05508-030 São Paulo - SP www.lct.poli.usp.br
Tel: 11 3091-5151 e-mail: lct@lct.poli.usp.br

DIFRATOGRAMA DE RAIOS X

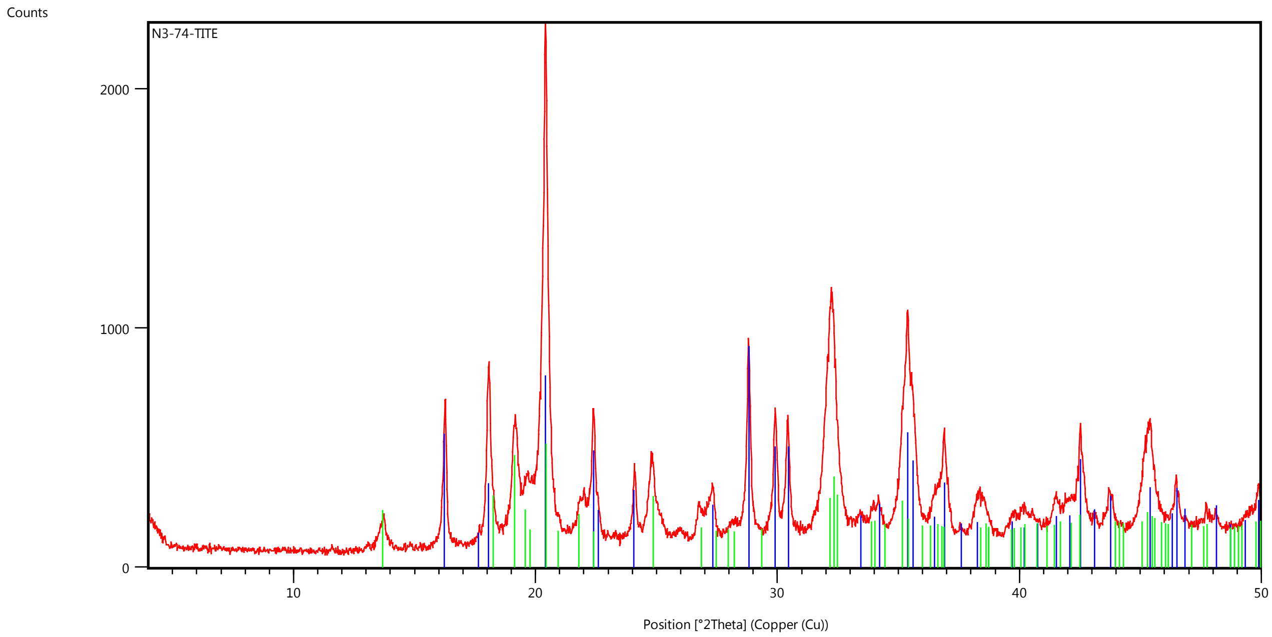

FASES IDENTIFICADAS

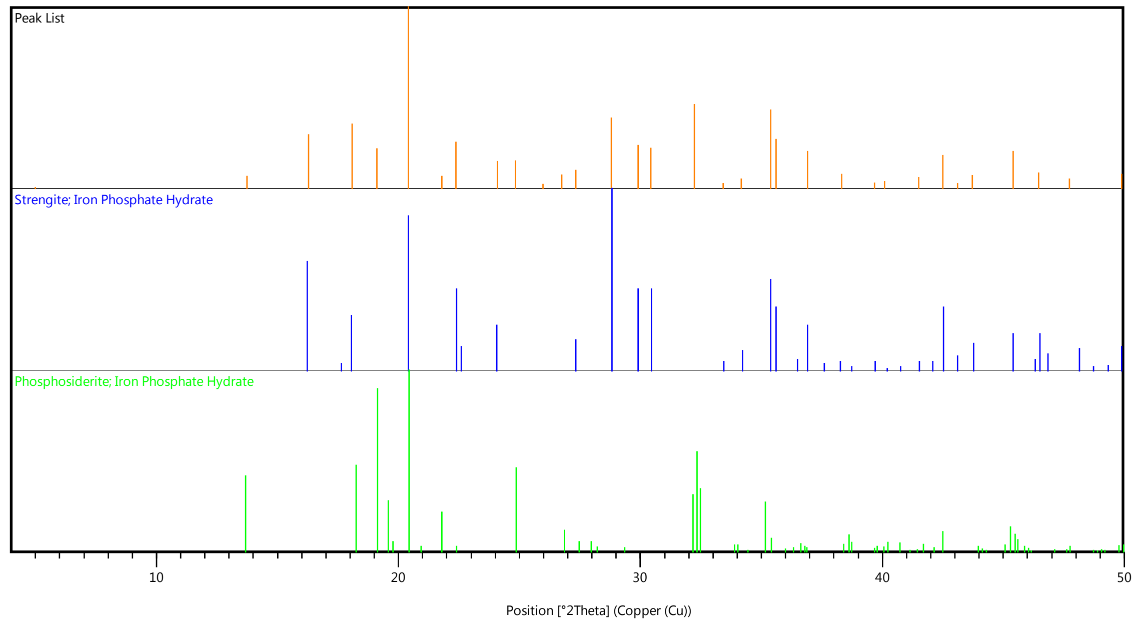

Escola Politécnica da Universidade de São Paulo
Departamento de Engenharia de Minas e de Petróleo
Laboratório de Caracterização Tecnológica
Av. Prof. Mello Moraes, 2373 CEP 05508-030 São Paulo - SP www.lct.poli.usp.br
Tel: 11 3091-5151 e-mail: lct@lct.poli.usp.br

RESULTADO DE IDENTIFICAÇÃO DE FASES POR DIFRATOMETRIA DE RAIOS X

RELATÓRIO: DRX 609/19 **DATA:** 12/04/19

CLIENTE: Luís Piló

AMOSTRA: S11A-36-TITE **IDENT. LCT:** 130-2096.HPF

1. MÉTODO

O estudo foi efetuado através do método do pó, mediante o emprego de difratômetro de raios X com detector sensível a posição.

A identificação das fases cristalinas, abaixo discriminadas, foi obtida por comparação do difratograma da amostra com os bancos de dados PDF2 do ICDD - International Centre for Diffraction Data e ICSD – Inorganic Crystal Structure Database.

2. RESULTADOS

Os resultados obtidos estão listados na tabela abaixo:

ICDD	Mineral/Composto	Fórmula Química	Obs
00-033-0667	Strengita	$FePO_4\ 2H_2O$	
01-076-0447	Fosfosiderita	$FePO_4\ (H_2O)_2$	
00-037-0466	Leucofosfita	$KFe_2^{+3}(PO_4)_2(OH)\ 2H_2O$	pp

Nota: pp = possível presença

O difratograma obtido (cor vermelha), onde são assinaladas as linhas de difração correspondente(s) à(s) fase(s) identificada(s) (cada fase em uma cor distinta) é apresentado anexo.

Profa. Dra. Carina Ulsen
Coordenadora do LCT

Dra. Maria Manuela Tassinari
Pesquisadora sênior

Dra. Juliana Lívi Antoniassi
Pesquisadora

Verifique a autenticidade deste documento em www.lct.poli.usp.br utilizando o código SKQA-BKJA-JITA-KQXB

DIFRATOGRAMA DE RAIOS X

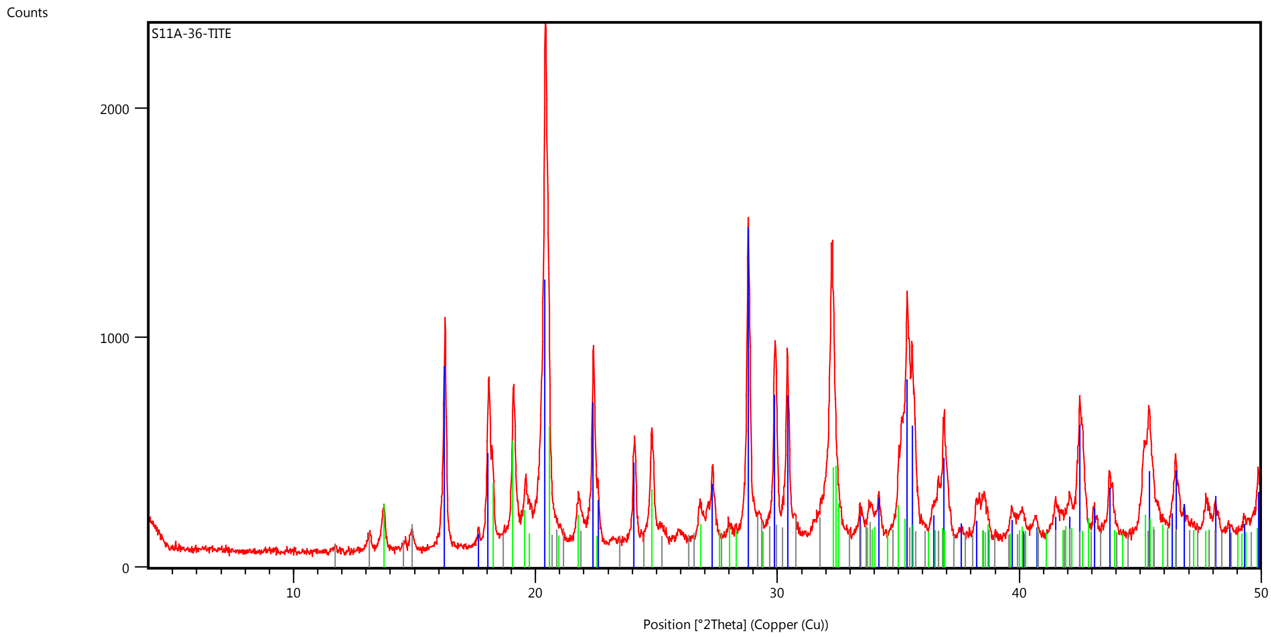

FASES IDENTIFICADAS

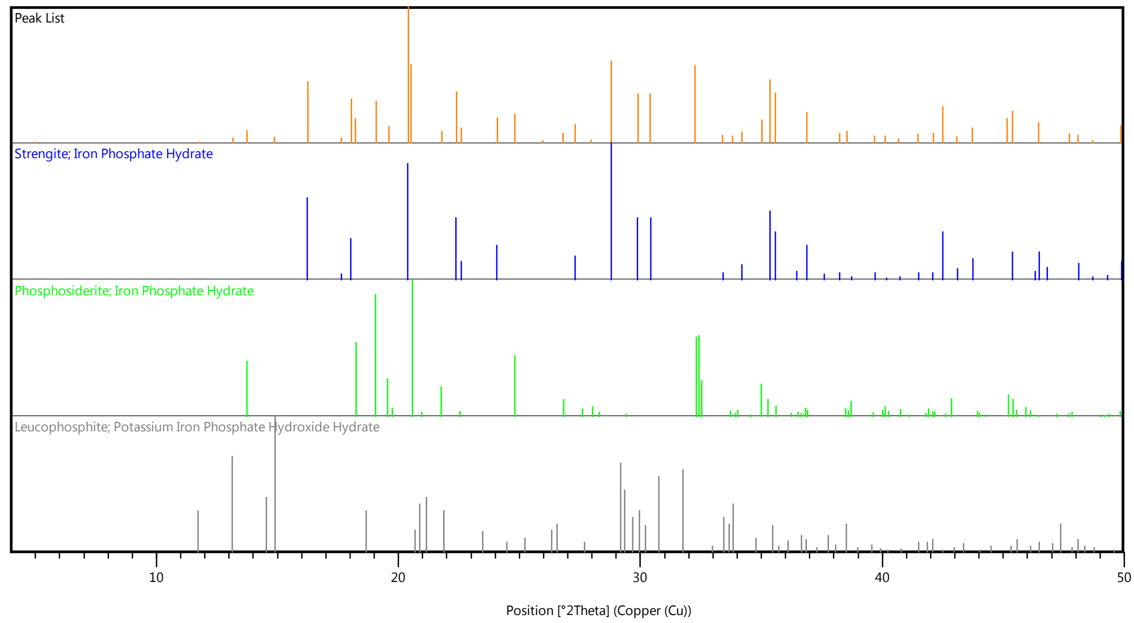

Escola Politécnica da Universidade de São Paulo
Departamento de Engenharia de Minas e de Petróleo
Laboratório de Caracterização Tecnológica
Av. Prof. Mello Moraes, 2373 CEP 05508-030 São Paulo - SP www.lct.poli.usp.br
Tel: 11 3091-5151 e-mail: lct@lct.poli.usp.br

RESULTADO DE IDENTIFICAÇÃO DE FASES POR DIFRATOMETRIA DE RAIOS X

RELATÓRIO: DRX 1019/19 **DATA:** 23/09/2019

CLIENTE: Luís Piló

AMOSTRA: N4WS-15 - TITE **IDENT. LCT:** 391-7696.HPF

1. MÉTODO

O estudo foi efetuado através do método do pó, mediante o emprego de difratômetro de raios X com detector sensível a posição.

A identificação das fases cristalinas, abaixo discriminadas, foi obtida por comparação do difratograma da amostra com os bancos de dados PDF2 do ICDD - International Centre for Diffraction Data e ICSD – Inorganic Crystal Structure Database.

2. RESULTADOS

Os resultados obtidos estão listados na tabela abaixo:

ICDD	Mineral/Composto	Fórmula Química	Obs
00-033-0667	Estrengita	$FePO_4\ 2H_2O$	
01-087-1165	Hematita	Fe_2O_3	
01-075-1346	Cacoxenita	$(Al_4Fe_{21}(PO_4)_{17}O_6(OH)_{12}(H_2O)_{24})$	pp

Nota: pp = possível presença

O difratograma obtido (cor vermelha), onde são assinaladas as linhas de difração correspondente(s) à(s) fase(s) identificada(s) (cada fase em uma cor distinta) é apresentado anexo.

Executado por: M.Sc. Gaspar Darin Filho (24/09/2019 09:55 BRT)
Revisado por: Dra. Maria Manuela Tassinari (24/09/2019 11:48 BRT)

Profa. Dra. Carina Ulsen
Coordenadora do LCT - Poli/USP

NOTA: Os resultados expostos acima referem-se apenas à(s) amostra(s) enviada(s) ao LCT; a representatividade da(s) mesma(s) é de inteira responsabilidade do cliente.

Verifique a autenticidade deste documento em www.lct.poli.usp.br utilizando o código **AOQV-XGTZ-IVRQ-WHWB**

Escola Politécnica da Universidade de São Paulo
Departamento de Engenharia de Minas e de Petróleo
Laboratório de Caracterização Tecnológica
Av. Prof. Mello Moraes, 2373 CEP 05508-030 São Paulo - SP www.lct.poli.usp.br
Tel: 11 3091-5151 e-mail: lct@lct.poli.usp.br

DIFRATOGRAMA DE RAIOS X

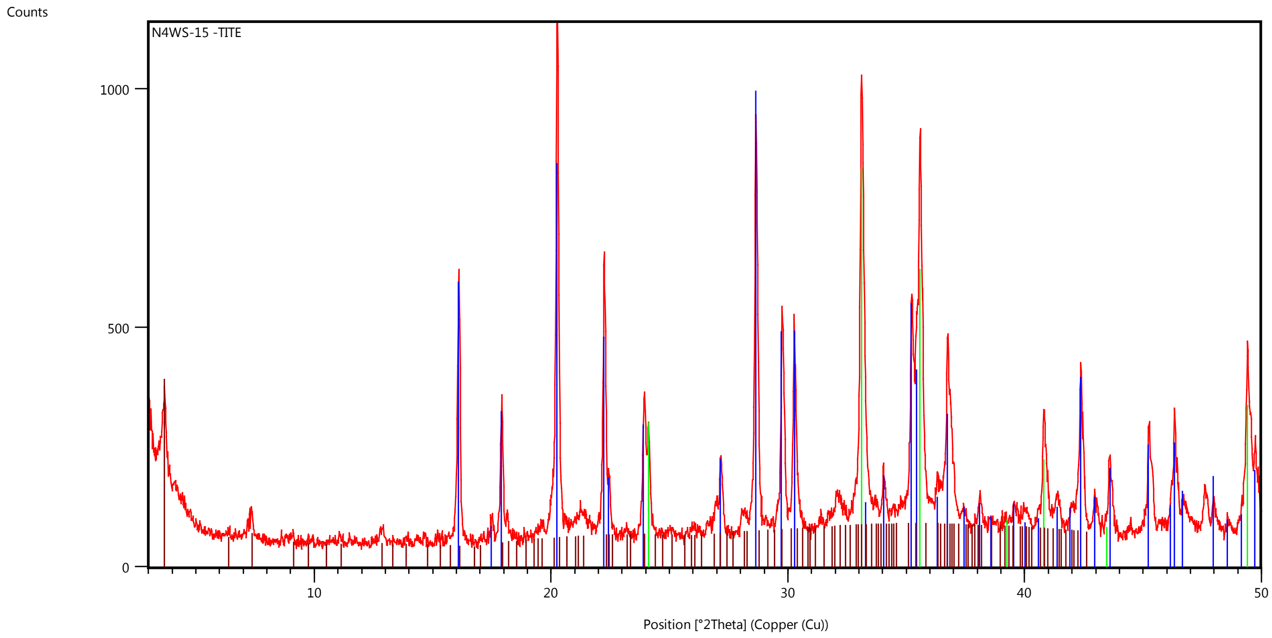

FASES IDENTIFICADAS

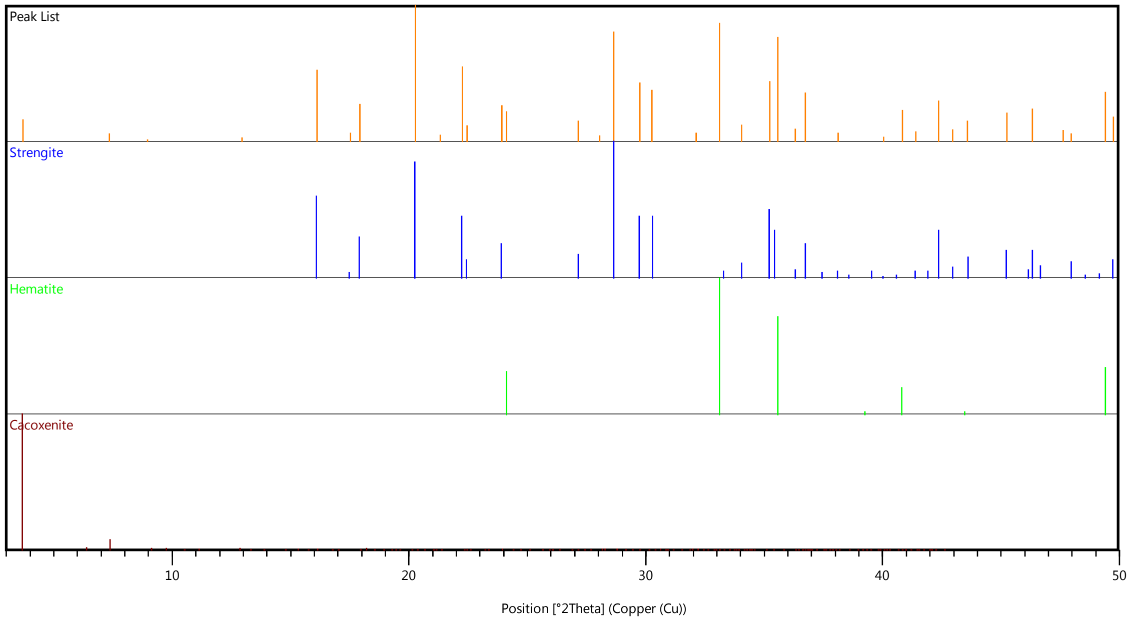

Escola Politécnica da Universidade de São Paulo
Departamento de Engenharia de Minas e de Petróleo
Laboratório de Caracterização Tecnológica
Av. Prof. Mello Moraes, 2373 CEP 05508-030 São Paulo - SP www.lct.poli.usp.br
Tel: 11 3091-5151 e-mail: lct@lct.poli.usp.br

RESULTADO DE IDENTIFICAÇÃO DE FASES POR DIFRATOMETRIA DE RAIOS X

RELATÓRIO: DRX 1020/19 **DATA:** 23/09/2019

CLIENTE: Luís Piló

AMOSTRA: N4WS-72 - TITE **IDENT. LCT:** 391-7697.HPF

1. MÉTODO

O estudo foi efetuado através do método do pó, mediante o emprego de difratômetro de raios X com detector sensível a posição.

A identificação das fases cristalinas, abaixo discriminadas, foi obtida por comparação do difratograma da amostra com os bancos de dados PDF2 do ICDD - International Centre for Diffraction Data e ICSD – Inorganic Crystal Structure Database.

2. RESULTADOS

Os resultados obtidos estão listados na tabela abaixo:

ICDD	Mineral/Composto	Fórmula Química	Obs
00-033-0667	Estrengita	$FePO_4\ 2H_2O$	
01-076-0451	Fosfosiderita	$FePO_4(H_2O)_2$	
01-080-0759	Hidroxi-fosfato de ferro	$Fe_4(PO_4)_3(OH)_3$	pp

Nota: pp = possível presença

O difratograma obtido (cor vermelha), onde são assinaladas as linhas de difração correspondente(s) à(s) fase(s) identificada(s) (cada fase em uma cor distinta) é apresentado anexo.

Executado por: M.Sc. Gaspar Darin Filho (24/09/2019 09:55 BRT)
Revisado por: Dra. Maria Manuela Tassinari (24/09/2019 11:48 BRT)

Profa. Dra. Carina Ulsen
Coordenadora do LCT - Poli/USP

NOTA: Os resultados expostos acima referem-se apenas à(s) amostra(s) enviada(s) ao LCT; a representatividade da(s) mesma(s) é de inteira responsabilidade do cliente.

Verifique a autenticidade deste documento em www.lct.poli.usp.br utilizando o código **EOQW-AGTZ-GGRQ-MCWB**

DIFRATOGRAMA DE RAIOS X

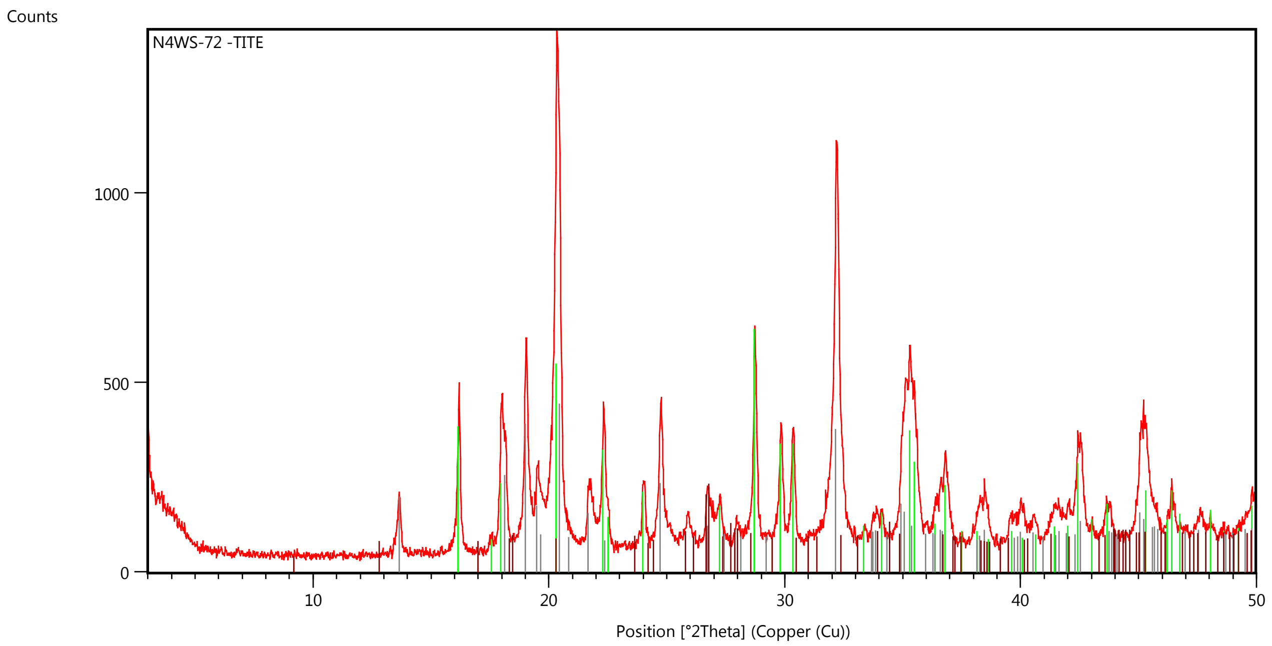

FASES IDENTIFICADAS

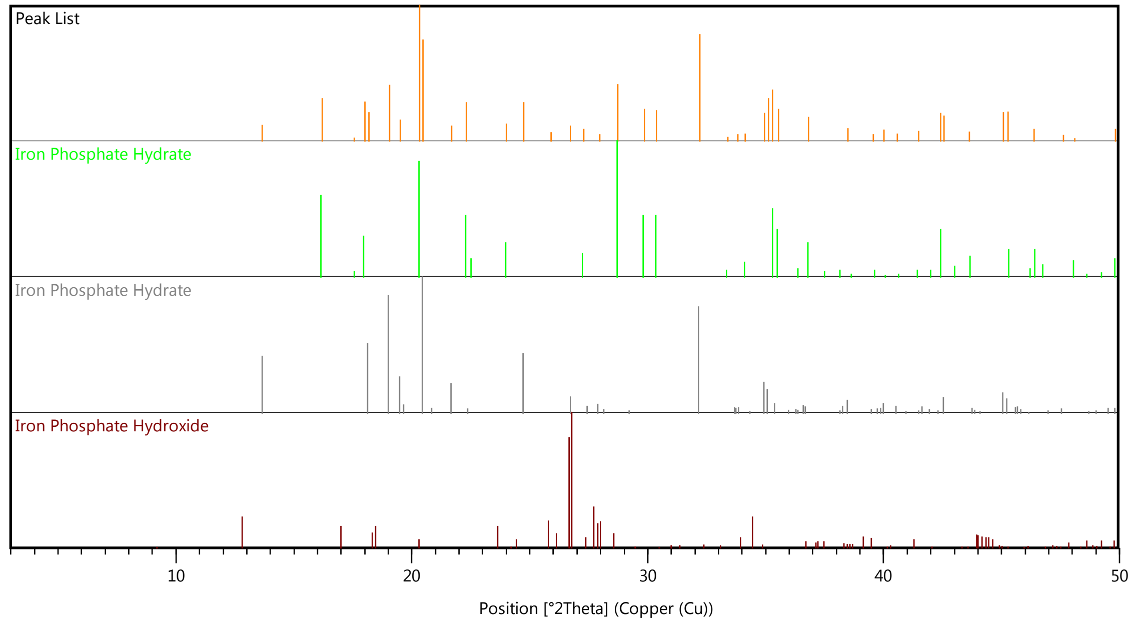

Escola Politécnica da Universidade de São Paulo
Departamento de Engenharia de Minas e de Petróleo
Laboratório de Caracterização Tecnológica
Av. Prof. Mello Moraes, 2373 CEP 05508-030 São Paulo - SP www.lct.poli.usp.br
Tel: 11 3091-5151 e-mail: lct@lct.poli.usp.br

RESULTADO DE IDENTIFICAÇÃO DE FASES POR DIFRATOMETRIA DE RAIOS X

RELATÓRIO: DRX 1021/19 **DATA:** 23/09/2019

CLIENTE: Luís Piló

AMOSTRA: N4WS-72A - TITE **IDENT. LCT:** 391-7698.HPF

1. MÉTODO

O estudo foi efetuado através do método do pó, mediante o emprego de difratômetro de raios X com detector sensível a posição.

A identificação das fases cristalinas, abaixo discriminadas, foi obtida por comparação do difratograma da amostra com os bancos de dados PDF2 do ICDD - International Centre for Diffraction Data e ICSD – Inorganic Crystal Structure Database.

2. RESULTADOS

Os resultados obtidos estão listados na tabela abaixo:

ICDD	Mineral/Composto	Fórmula Química	Obs
01-076-0451	Fosfosiderita	$FePO_4(H_2O)_2$	
00-033-0667	Estrengita	$FePO_4$ $2H_2O$	
01-080-0759	Hidroxi-fosfato de ferro	$Fe_4(PO_4)_3(OH)_3$	pp
00-041-0593	Spheniscidita	$(NH_4)Fe_2(PO_4)_2(OH)$ $2H_2O$	pp

Nota: pp = possível presença

O difratograma obtido (cor vermelha), onde são assinaladas as linhas de difração correspondente(s) à(s) fase(s) identificada(s) (cada fase em uma cor distinta) é apresentado anexo.

Executado por: M.Sc. Gaspar Darin Filho (24/09/2019 09:55 BRT)
Revisado por: Dra. Maria Manuela Tassinari (24/09/2019 11:48 BRT)

Profa. Dra. Carina Ulsen
Coordenadora do LCT - Poli/USP

NOTA: Os resultados expostos acima referem-se apenas à(s) amostra(s) enviada(s) ao LCT; a representatividade da(s) mesma(s) é de inteira responsabilidade do cliente.

Verifique a autenticidade deste documento em www.lct.poli.usp.br utilizando o código **KOQX-NHTZ-ECRQ-SIWB**

DIFRATOGRAMA DE RAIOS X

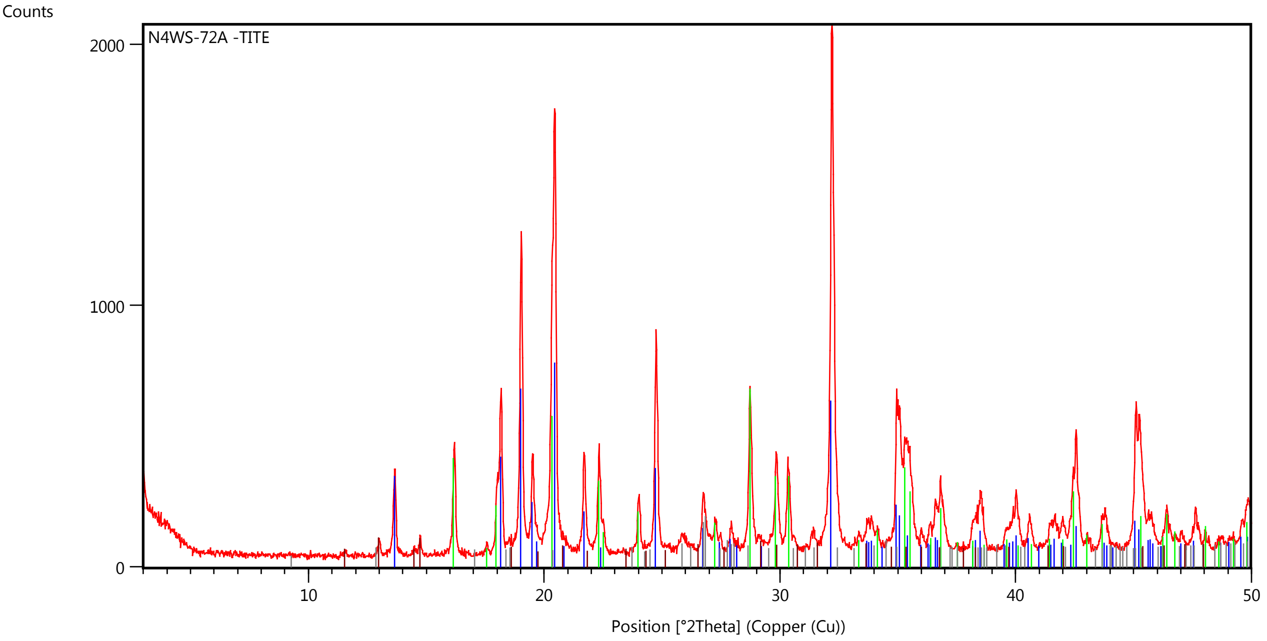

FASES IDENTIFICADAS

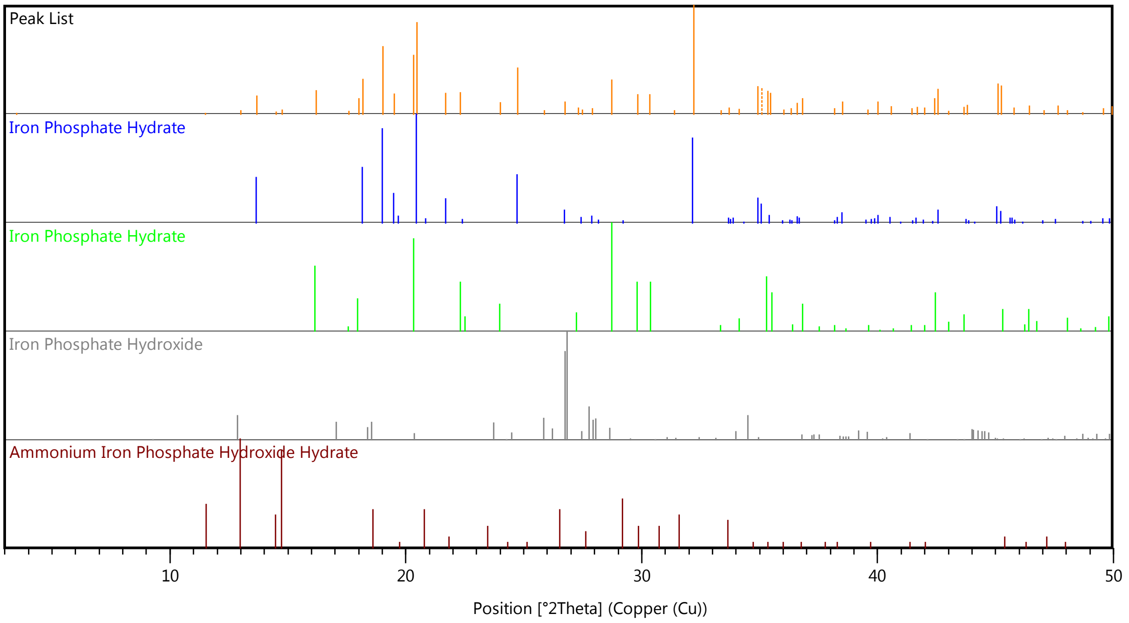

Escola Politécnica da Universidade de São Paulo
Departamento de Engenharia de Minas e de Petróleo
Laboratório de Caracterização Tecnológica
Av. Prof. Mello Moraes, 2373 CEP 05508-030 São Paulo - SP www.lct.poli.usp.br
Tel: 11 3091-5151 e-mail: lct@lct.poli.usp.br

RESULTADO DE IDENTIFICAÇÃO DE FASES POR DIFRATOMETRIA DE RAIOS X

RELATÓRIO: DRX 1022/19 **DATA:** 23/09/2019

CLIENTE: Luís Piló

AMOSTRA: N4WS-67 - TITE **IDENT. LCT:** 391-7699.HPF

1. MÉTODO

O estudo foi efetuado através do método do pó, mediante o emprego de difratômetro de raios X com detector sensível a posição.

A identificação das fases cristalinas, abaixo discriminadas, foi obtida por comparação do difratograma da amostra com os bancos de dados PDF2 do ICDD - International Centre for Diffraction Data e ICSD – Inorganic Crystal Structure Database.

2. RESULTADOS

Os resultados obtidos estão listados na tabela abaixo:

ICDD	Mineral/Composto	Fórmula Química	Obs
01-082-1164	Spheniscidita	$Fe_2(NH_4)(OH)(PO_4)_2(H_2O)_2$	e/ou
00-037-0466	Leucophosphita	KFe_2^{+3} $(PO_4)_2(OH)$ $2H_2O$	
01-076-0451	Fosfosiderita	$FePO_4(H_2O)_2$	
00-033-0667	Estrengita	$FePO_4$ $2H_2O$	pp

Nota: pp = possível presença

O difratograma obtido (cor vermelha), onde são assinaladas as linhas de difração correspondente(s) à(s) fase(s) identificada(s) (cada fase em uma cor distinta) é apresentado anexo.

Executado por: M.Sc. Gaspar Darin Filho (24/09/2019 09:55 BRT)
Revisado por: Dra. Maria Manuela Tassinari (24/09/2019 11:48 BRT)

Profa. Dra. Carina Ulsen
Coordenadora do LCT - Poli/USP

NOTA: Os resultados expostos acima referem-se apenas à(s) amostra(s) enviada(s) ao LCT; a representatividade da(s) mesma(s) é de inteira responsabilidade do cliente.

Verifique a autenticidade deste documento em www.lct.poli.usp.br utilizando o código **HOQY-OGTZ-LNRQ-OVWB**

DIFRATOGRAMA DE RAIOS X

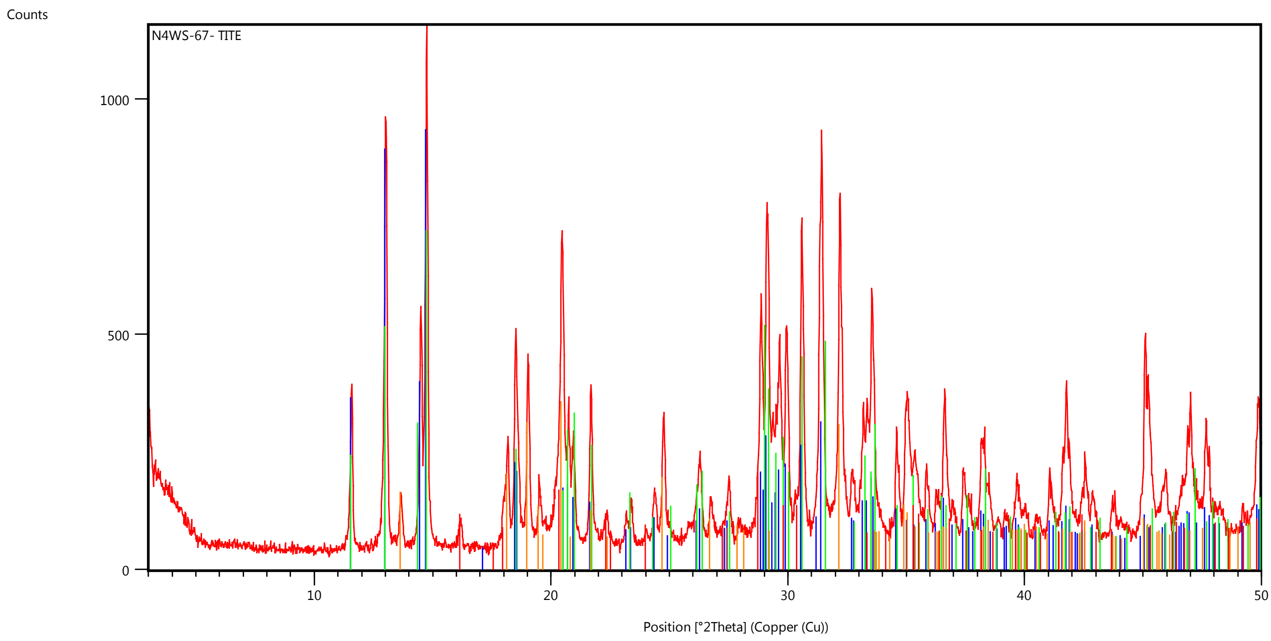

FASES IDENTIFICADAS

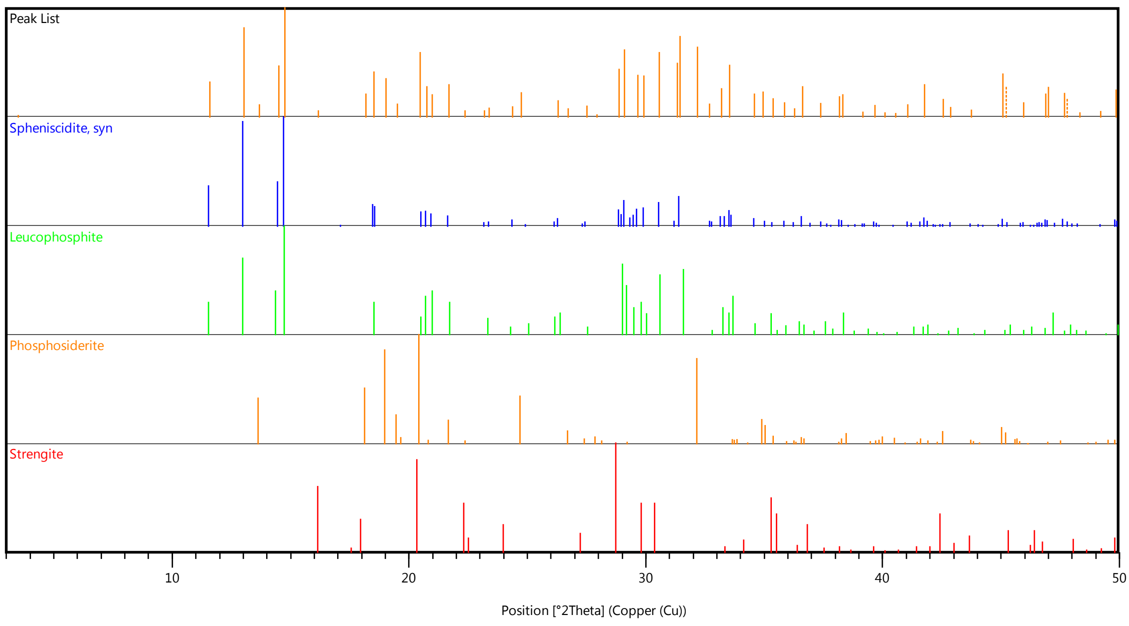

Escola Politécnica da Universidade de São Paulo
Departamento de Engenharia de Minas e de Petróleo
Laboratório de Caracterização Tecnológica
Av. Prof. Mello Moraes, 2373 CEP 05508-030 São Paulo - SP www.lct.poli.usp.br
Tel: 11 3091-5151 e-mail: lct@lct.poli.usp.br

RESULTADO DE IDENTIFICAÇÃO DE FASES POR DIFRATOMETRIA DE RAIOS X

RELATÓRIO: DRX 1023/19 **DATA:** 23/09/2019

CLIENTE: Luís Piló

AMOSTRA: N4WS-67A - TITE **IDENT. LCT:** 391-7700.HPF

1. MÉTODO

O estudo foi efetuado através do método do pó, mediante o emprego de difratômetro de raios X com detector sensível a posição.

A identificação das fases cristalinas, abaixo discriminadas, foi obtida por comparação do difratograma da amostra com os bancos de dados PDF2 do ICDD - International Centre for Diffraction Data e ICSD – Inorganic Crystal Structure Database.

2. RESULTADOS

Os resultados obtidos estão listados na tabela abaixo:

ICDD	Mineral/Composto	Fórmula Química	Obs
01-082-1164	Spheniscidita	$Fe_2(NH_4)(OH)(PO_4)_2(H_2O)_2$	e/ou
00-037-0466	Leucophosphita	$KFe_2{}^{+3}(PO_4)_2(OH)\ 2H_2O$	
01-076-0451	Fosfosiderita	$FePO_4(H_2O)_2$	
00-033-0667	Estrengita	$FePO_4\ 2H_2O$	

O difratograma obtido (cor vermelha), onde são assinaladas as linhas de difração correspondente(s) à(s) fase(s) identificada(s) (cada fase em uma cor distinta) é apresentado anexo.

Executado por: M.Sc. Gaspar Darin Filho (24/09/2019 09:55 BRT)
Revisado por: Dra. Maria Manuela Tassinari (24/09/2019 11:48 BRT)

Profa. Dra. Carina Ulsen
Coordenadora do LCT - Poli/USP

NOTA: Os resultados expostos acima referem-se apenas à(s) amostra(s) enviada(s) ao LCT; a representatividade da(s) mesma(s) é de inteira responsabilidade do cliente.

Verifique a autenticidade deste documento em www.lct.poli.usp.br utilizando o código **FOQZ-KHTZ-FJRQ-GTWB**

DIFRATOGRAMA DE RAIOS X

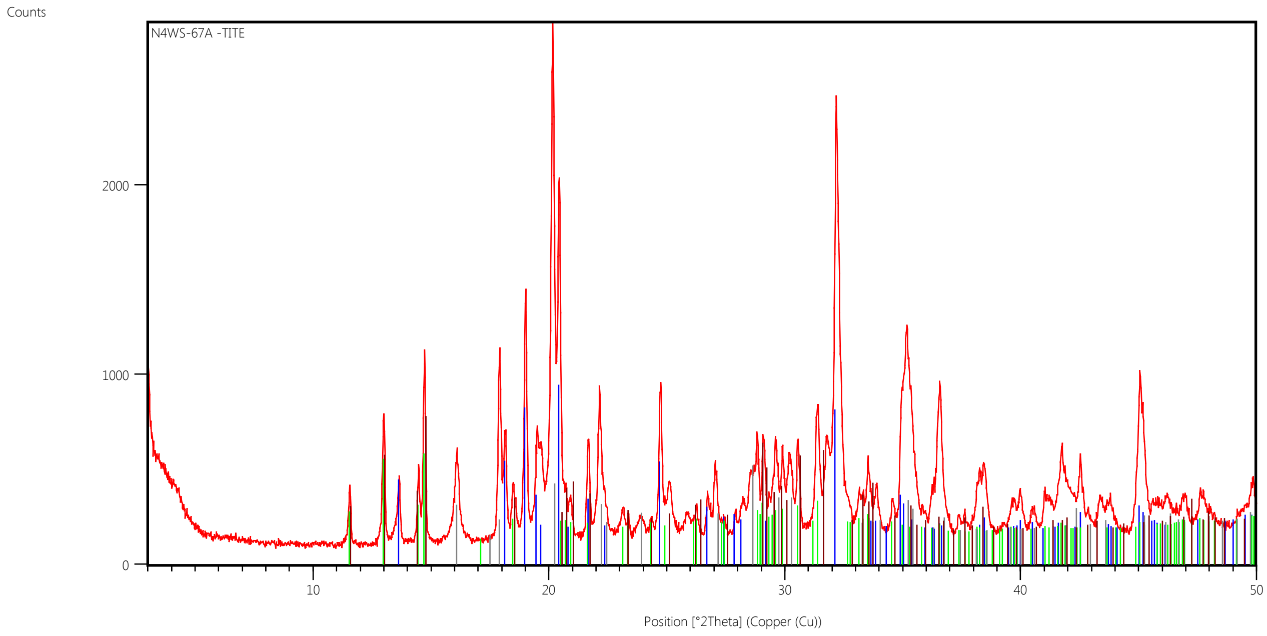

FASES IDENTIFICADAS

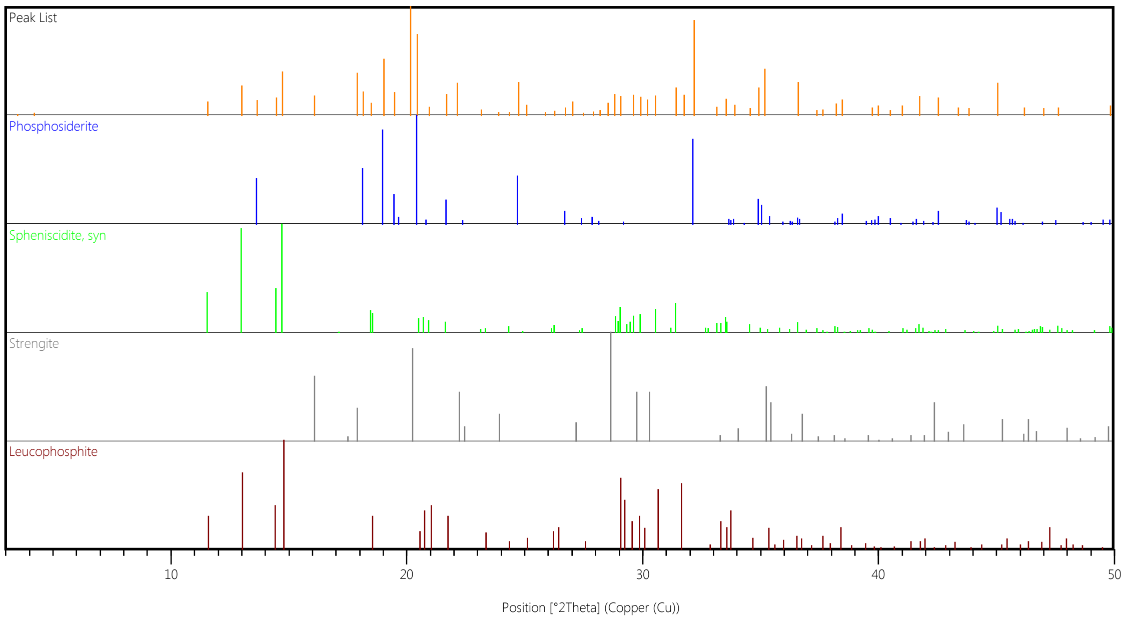

Escola Politécnica da Universidade de São Paulo
Departamento de Engenharia de Minas e de Petróleo
Laboratório de Caracterização Tecnológica
Av. Prof. Mello Moraes, 2373 CEP 05508-030 São Paulo - SP www.lct.poli.usp.br
Tel: 11 3091-5151 e-mail: lct@lct.poli.usp.br

RESULTADO DE IDENTIFICAÇÃO DE FASES POR DIFRATOMETRIA DE RAIOS X

RELATÓRIO: DRX 1024/19 **DATA:** 23/09/2019

CLIENTE: Luís Piló

AMOSTRA: M2-99 - TITE **IDENT. LCT:** 391-7701.HPF

1. MÉTODO

O estudo foi efetuado através do método do pó, mediante o emprego de difratômetro de raios X com detector sensível a posição.

A identificação das fases cristalinas, abaixo discriminadas, foi obtida por comparação do difratograma da amostra com os bancos de dados PDF2 do ICDD - International Centre for Diffraction Data e ICSD – Inorganic Crystal Structure Database.

2. RESULTADOS

Os resultados obtidos estão listados na tabela abaixo:

ICDD	Mineral/Composto	Fórmula Química	Obs
01-082-1164	Spheniscidita	$Fe_2(NH_4)(OH)(PO_4)_2(H_2O)_2$	e/ou
00-037-0466	Leucophosphita	$KFe_2^{+3}(PO_4)_2(OH)\ 2H_2O$	

O difratograma obtido (cor vermelha), onde são assinaladas as linhas de difração correspondente(s) à(s) fase(s) identificada(s) (cada fase em uma cor distinta) é apresentado anexo.

Executado por: M.Sc. Gaspar Darin Filho (24/09/2019 09:55 BRT)
Revisado por: Dra. Maria Manuela Tassinari (24/09/2019 11:48 BRT)

Profa. Dra. Carina Ulsen
Coordenadora do LCT - Poli/USP

NOTA: Os resultados expostos acima referem-se apenas à(s) amostra(s) enviada(s) ao LCT; a representatividade da(s) mesma(s) é de inteira responsabilidade do cliente.

Verifique a autenticidade deste documento em www.lct.poli.usp.br utilizando o código **BOQA-GHTA-OQRR-SZWB**

DIFRATOGRAMA DE RAIOS X

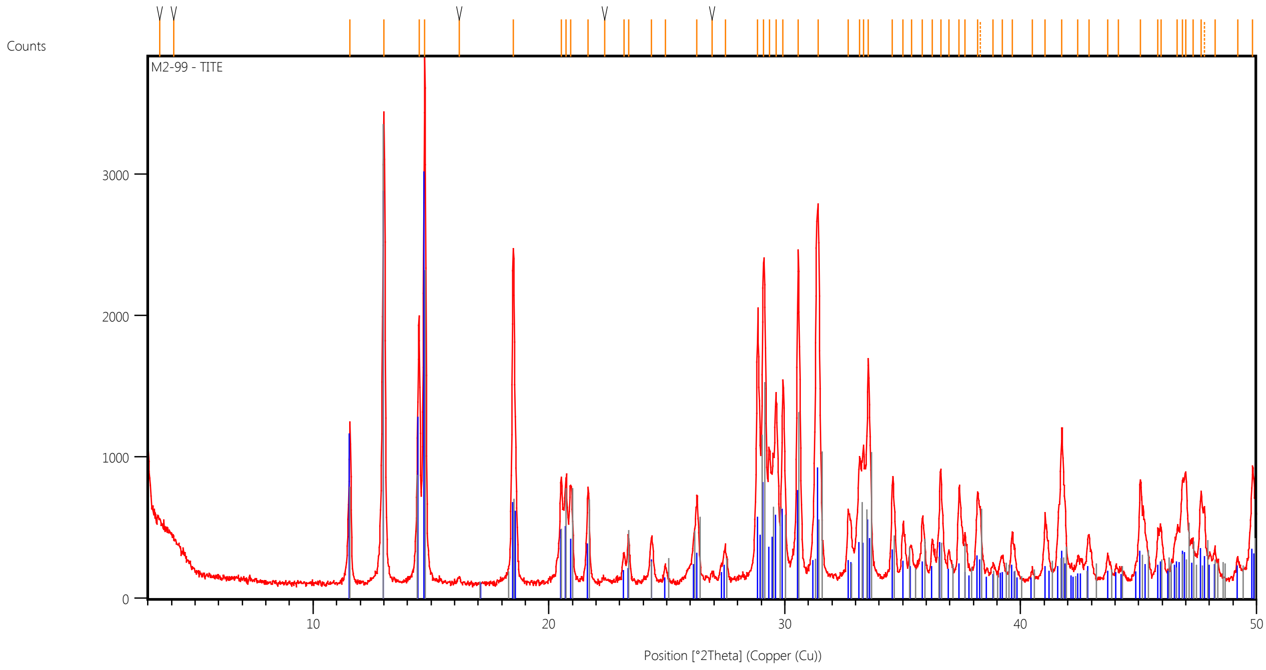

FASES IDENTIFICADAS

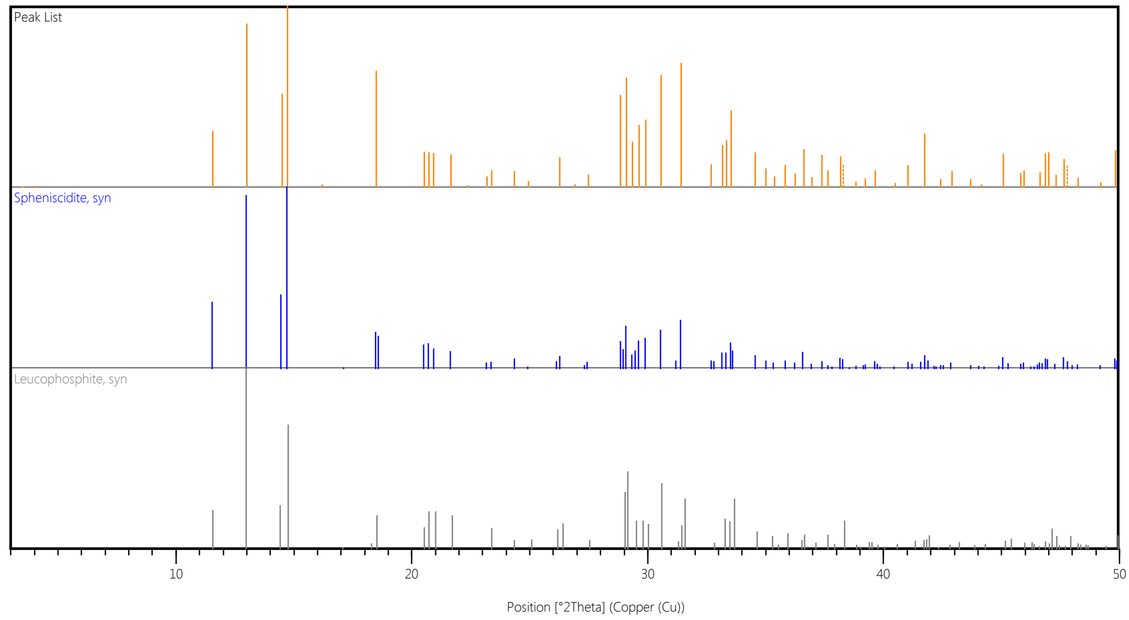

Escola Politécnica da Universidade de São Paulo
Departamento de Engenharia de Minas e de Petróleo
Laboratório de Caracterização Tecnológica
Av. Prof. Mello Moraes, 2373 CEP 05508-030 São Paulo - SP www.lct.poli.usp.br
Tel: 11 3091-5151 e-mail: lct@lct.poli.usp.br

RESULTADO DE IDENTIFICAÇÃO DE FASES POR DIFRATOMETRIA DE RAIOS X

RELATÓRIO: DRX 1025/19 **DATA:** 23/09/2019

CLIENTE: Luís Piló

AMOSTRA: M2-99A - TITE **IDENT. LCT:** 391-7702.HPF

1. MÉTODO

O estudo foi efetuado através do método do pó, mediante o emprego de difratômetro de raios X com detector sensível a posição.

A identificação das fases cristalinas, abaixo discriminadas, foi obtida por comparação do difratograma da amostra com os bancos de dados PDF2 do ICDD - International Centre for Diffraction Data e ICSD – Inorganic Crystal Structure Database.

2. RESULTADOS

Os resultados obtidos estão listados na tabela abaixo:

ICDD	Mineral/Composto	Fórmula Química	Obs
01-082-1164	Spheniscidita	$Fe_2(NH_4)(OH)(PO_4)_2(H_2O)_2$	e/ou
00-037-0466	Leucophosphita	$KFe_2^{+3}(PO_4)_2(OH)\ 2H_2O$	

O difratograma obtido (cor vermelha), onde são assinaladas as linhas de difração correspondente(s) à(s) fase(s) identificada(s) (cada fase em uma cor distinta) é apresentado anexo.

Executado por: M.Sc. Gaspar Darin Filho (24/09/2019 09:55 BRT)
Revisado por: Dra. Maria Manuela Tassinari (24/09/2019 11:48 BRT)

Profa. Dra. Carina Ulsen
Coordenadora do LCT - Poli/USP

NOTA: Os resultados expostos acima referem-se apenas à(s) amostra(s) enviada(s) ao LCT; a representatividade da(s) mesma(s) é de inteira responsabilidade do cliente.

Verifique a autenticidade deste documento em www.lct.poli.usp.br utilizando o código **GOQB-UHTA-RXRR-OYWB**

DIFRATOGRAMA DE RAIOS X

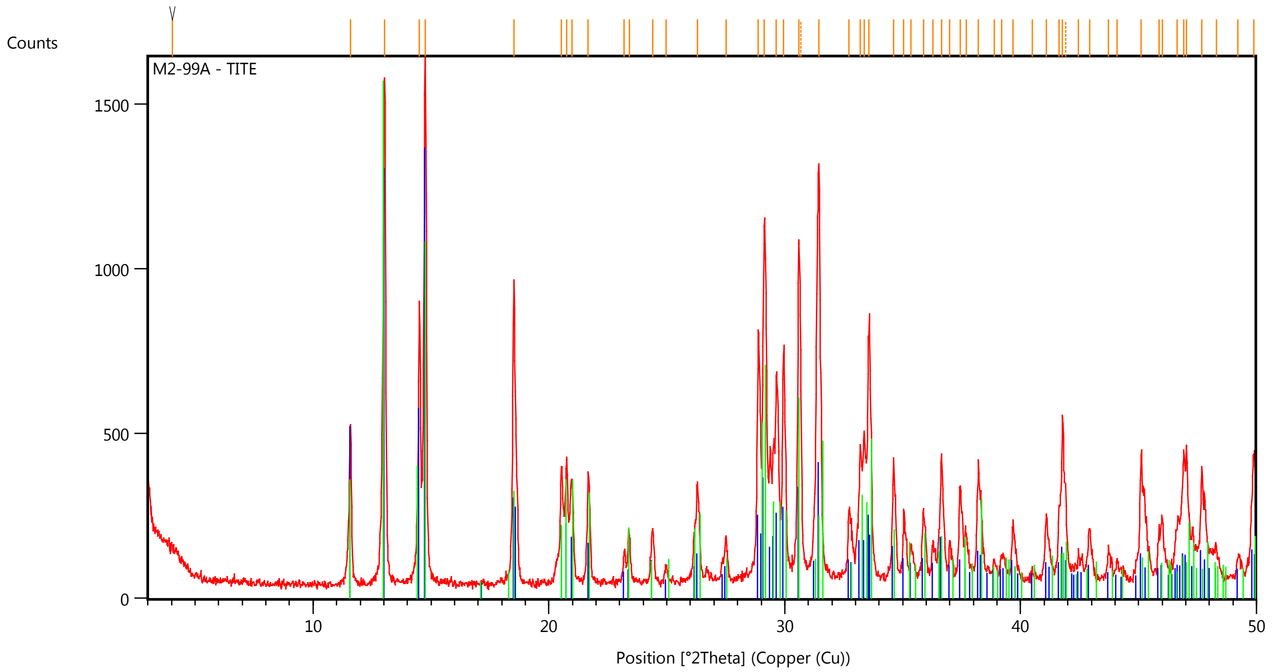

FASES IDENTIFICADAS

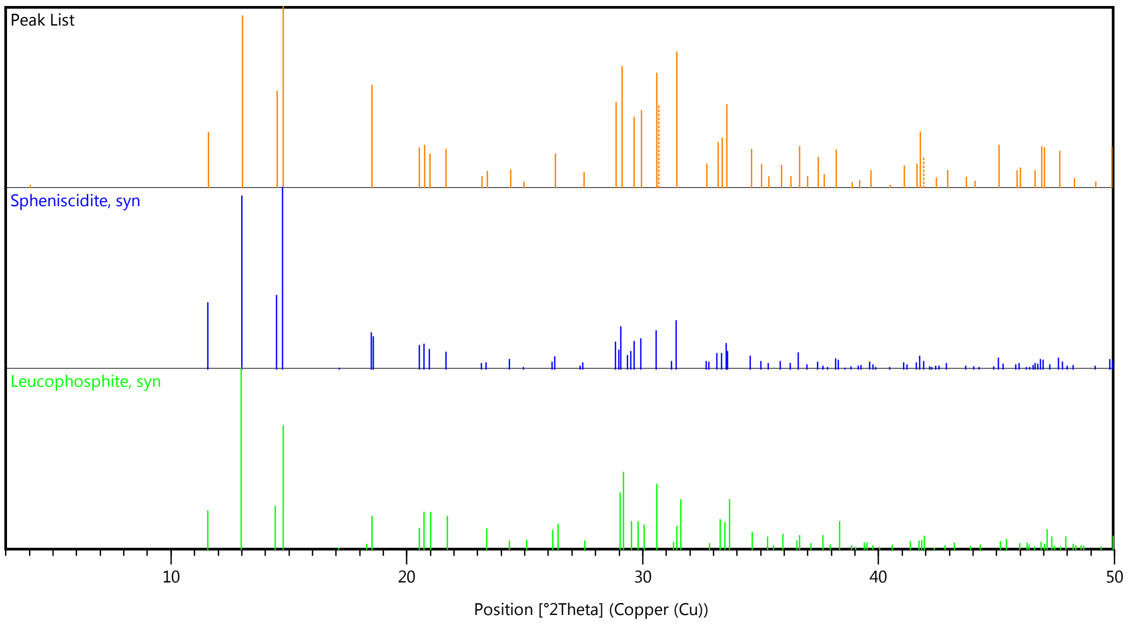

Escola Politécnica da Universidade de São Paulo
Departamento de Engenharia de Minas e de Petróleo
Laboratório de Caracterização Tecnológica
Av. Prof. Mello Moraes, 2373 CEP 05508-030 São Paulo - SP www.lct.poli.usp.br
Tel: 11 3091-5151 e-mail: lct@lct.poli.usp.br

RESULTADO DE IDENTIFICAÇÃO DE FASES POR DIFRATOMETRIA DE RAIOS X

RELATÓRIO: DRX 1026/19 **DATA:** 23/09/2019

CLIENTE: Luís Piló

AMOSTRA: S11B-94 - MITE **IDENT. LCT:** 391-7703.HPF

1. MÉTODO

O estudo foi efetuado através do método do pó, mediante o emprego de difratômetro de raios X com detector sensível a posição.

A identificação das fases cristalinas, abaixo discriminadas, foi obtida por comparação do difratograma da amostra com os bancos de dados PDF2 do ICDD - International Centre for Diffraction Data e ICSD – Inorganic Crystal Structure Database.

2. RESULTADOS

Os resultados obtidos estão listados na tabela abaixo:

ICDD	Mineral/Composto	Fórmula Química	Obs
01-082-1164	Spheniscidita	$Fe_2(NH_4)(OH)(PO_4)_2(H_2O)_2$	e/ou
00-037-0466	Leucophosphita	$KFe_2{}^{+3}(PO_4)_2(OH)\ 2H_2O$	
00-033-0667	Estrengita	$FePO_4\ 2H_2O$	
01-076-0451	Fosfosiderita	$FePO_4(H_2O)_2$	pp

Nota: pp = possível presença

O difratograma obtido (cor vermelha), onde são assinaladas as linhas de difração correspondente(s) à(s) fase(s) identificada(s) (cada fase em uma cor distinta) é apresentado anexo.

Executado por: M.Sc. Gaspar Darin Filho (24/09/2019 09:55 BRT)
Revisado por: Dra. Maria Manuela Tassinari (24/09/2019 11:48 BRT)

Profa. Dra. Carina Ulsen
Coordenadora do LCT - Poli/USP

NOTA: Os resultados expostos acima referem-se apenas à(s) amostra(s) enviada(s) ao LCT; a representatividade da(s) mesma(s) é de inteira responsabilidade do cliente.

Verifique a autenticidade deste documento em www.lct.poli.usp.br utilizando o código **MOQC-KITA-IFRR-QCWB**

Escola Politécnica da Universidade de São Paulo
Departamento de Engenharia de Minas e de Petróleo
Laboratório de Caracterização Tecnológica
Av. Prof. Mello Moraes, 2373 CEP 05508-030 São Paulo - SP www.lct.poli.usp.br
Tel: 11 3091-5151 e-mail: lct@lct.poli.usp.br

DIFRATOGRAMA DE RAIOS X

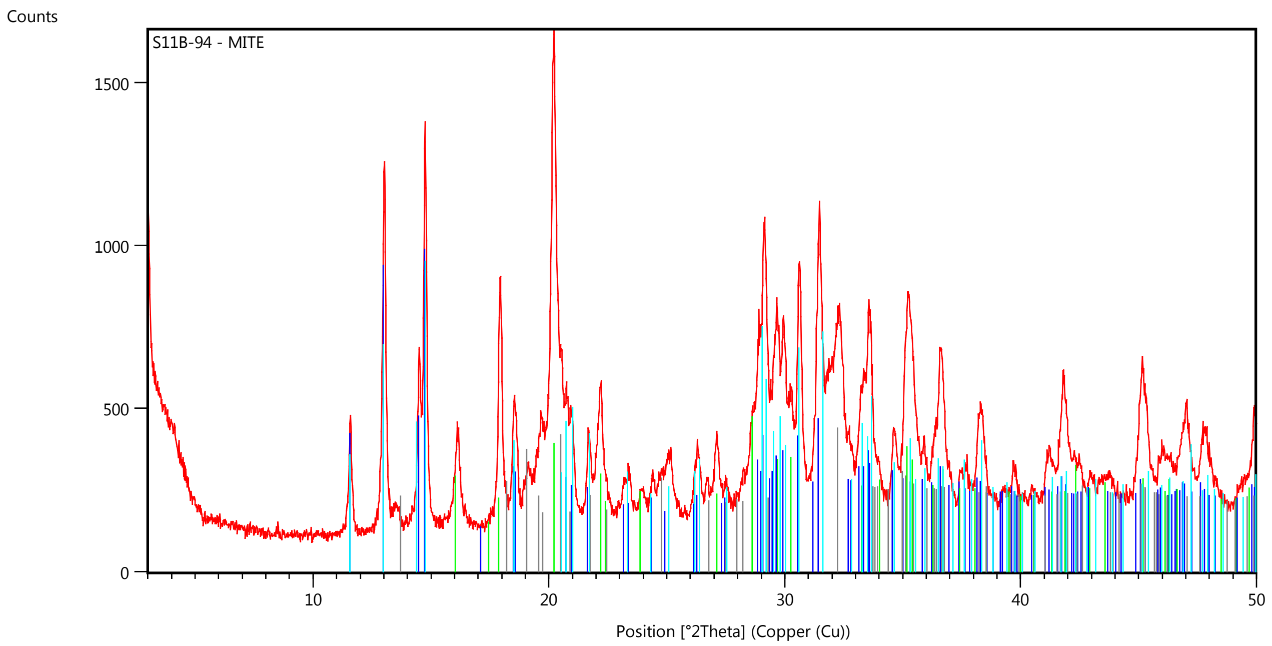

FASES IDENTIFICADAS

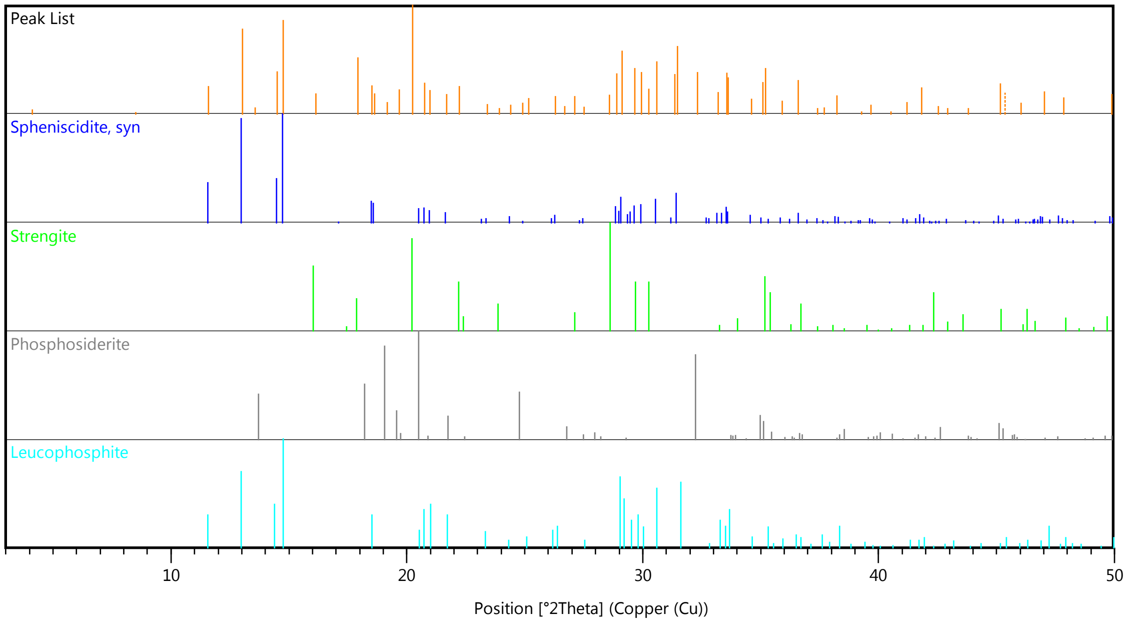

Escola Politécnica da Universidade de São Paulo
Departamento de Engenharia de Minas e de Petróleo
Laboratório de Caracterização Tecnológica
Av. Prof. Mello Moraes, 2373 CEP 05508-030 São Paulo - SP www.lct.poli.usp.br
Tel: 11 3091-5151 e-mail: lct@lct.poli.usp.br

RESULTADO DE IDENTIFICAÇÃO DE FASES POR DIFRATOMETRIA DE RAIOS X

RELATÓRIO: DRX 1226/19 **DATA:** 11/12/2019

CLIENTE: Luís Piló

AMOSTRA: N3-23-TM-A **IDENT. LCT:** 511-10065.HPF

1. MÉTODO

O estudo foi efetuado através do método do pó, mediante o emprego de difratômetro de raios X com detector sensível a posição.

A identificação das fases cristalinas, abaixo discriminadas, foi obtida por comparação do difratograma da amostra com os bancos de dados PDF2 do ICDD - International Centre for Diffraction Data e ICSD – Inorganic Crystal Structure Database.

2. RESULTADOS

Os resultados obtidos estão listados na tabela abaixo:

ICDD	Mineral	Fórmula Química	Obs
01-074-1904	Gipsita	$Ca(SO_4)(H_2O)_2$	

O difratograma obtido (cor vermelha), onde são assinaladas as linhas de difração correspondente(s) à(s) fase(s) identificada(s) (cada fase em uma cor distinta) é apresentado anexo.

Executado por: M.Sc. Gaspar Darin Filho (16/12/2019 18:04 BRT)
Revisado por: Dra. Maria Manuela Tassinari (16/12/2019 18:24 BRT)

Profa. Dra. Carina Ulsen
Coordenadora do LCT - Poli/USP

NOTA: Os resultados expostos acima referem-se apenas à(s) amostra(s) enviada(s) ao LCT; a representatividade da(s) mesma(s) é de inteira responsabilidade do cliente.

Verifique a autenticidade deste documento em www.lct.poli.usp.br utilizando o código **UFQV-QYYU-SAUT-IPIB**

Av. Prof. Mello Moraes, 2373 CEP 05508-030 São Paulo - SP www.lct.poli.usp.br
Tel: 11 3091-5151 e-mail: lct@lct.poli.usp.br

DIFRATOGRAMA DE RAIOS X

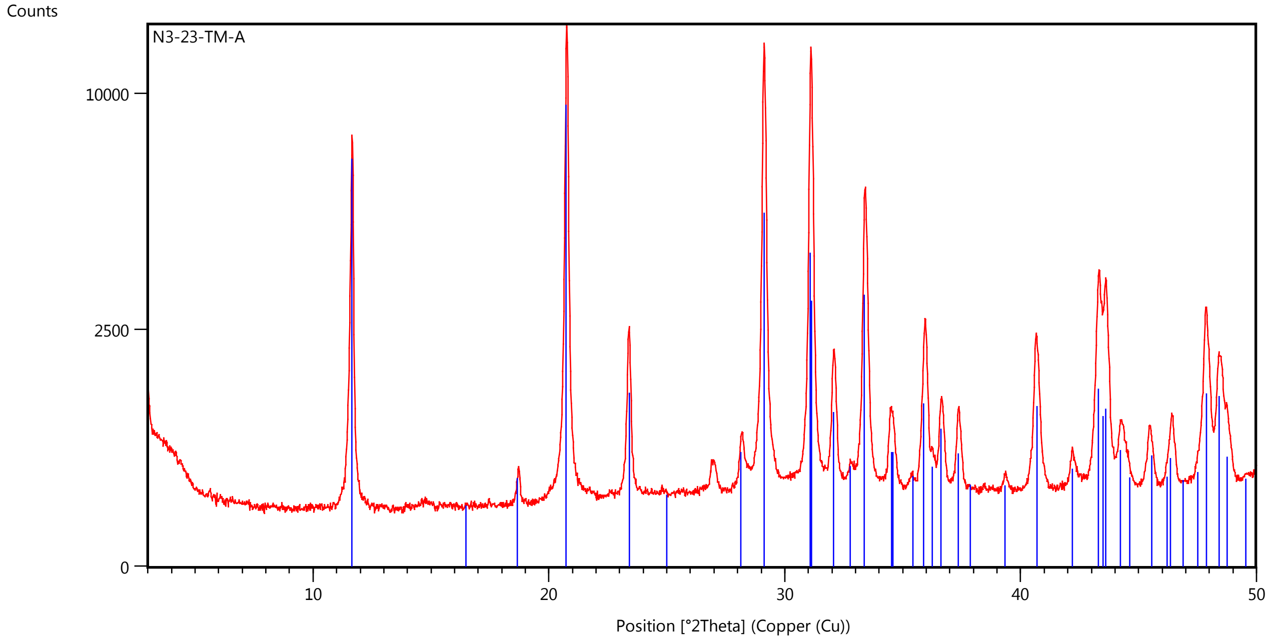

FASES IDENTIFICADAS

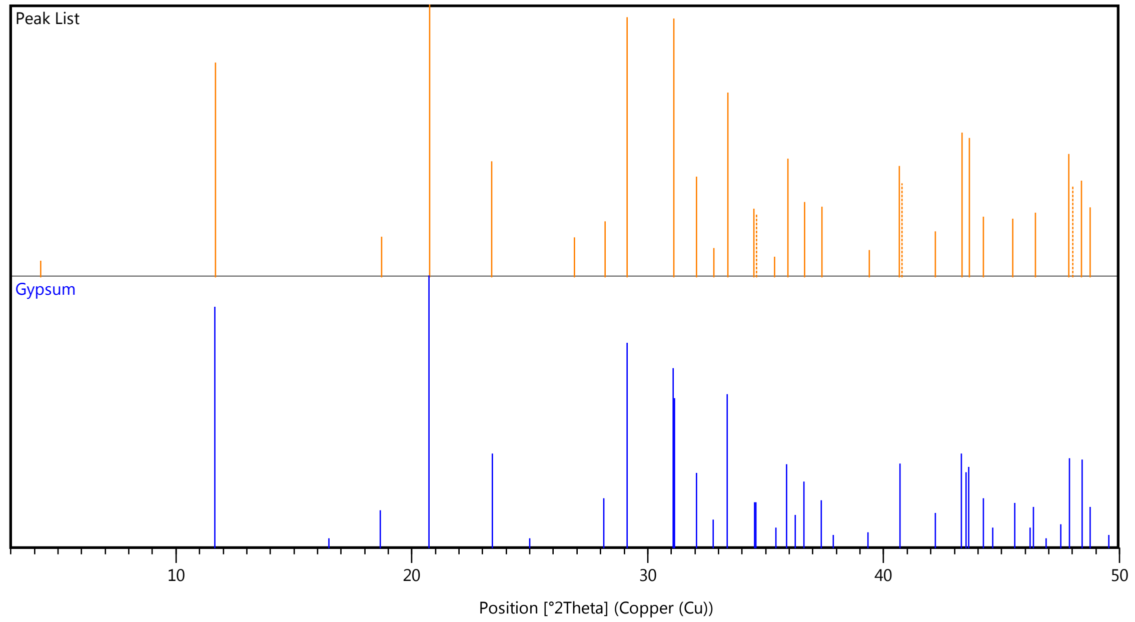

Escola Politécnica da Universidade de São Paulo
Departamento de Engenharia de Minas e de Petróleo
Laboratório de Caracterização Tecnológica
Av. Prof. Mello Moraes, 2373 CEP 05508-030 São Paulo - SP www.lct.poli.usp.br
Tel: 11 3091-5151 e-mail: lct@lct.poli.usp.br

RESULTADO DE IDENTIFICAÇÃO DE FASES POR DIFRATOMETRIA DE RAIOS X

RELATÓRIO: DRX 1227/19 **DATA:** 11/12/2019

CLIENTE: Luís Piló

AMOSTRA: N3-23-TM-B **IDENT. LCT:** 511-10066.HPF

1. MÉTODO

O estudo foi efetuado através do método do pó, mediante o emprego de difratômetro de raios X com detector sensível a posição.

A identificação das fases cristalinas, abaixo discriminadas, foi obtida por comparação do difratograma da amostra com os bancos de dados PDF2 do ICDD - International Centre for Diffraction Data e ICSD – Inorganic Crystal Structure Database.

2. RESULTADOS

Os resultados obtidos estão listados na tabela abaixo:

ICDD	Mineral	Fórmula Química	Obs
01-082-1164	Spheniscidita	$Fe_2(NH_4)(OH)(PO_4)_2(H_2O)_2$	
00-029-0981	Taranakita	$H_6K_3Al_5(PO_4)_8$ 18H_2O	

O difratograma obtido (cor vermelha), onde são assinaladas as linhas de difração correspondente(s) à(s) fase(s) identificada(s) (cada fase em uma cor distinta) é apresentado anexo.

Executado por: M.Sc. Gaspar Darin Filho (16/12/2019 18:04 BRT)
Revisado por: Dra. Maria Manuela Tassinari (16/12/2019 18:24 BRT)

Profa. Dra. Carina Ulsen
Coordenadora do LCT - Poli/USP

NOTA: Os resultados expostos acima referem-se apenas à(s) amostra(s) enviada(s) ao LCT; a representatividade da(s) mesma(s) é de inteira responsabilidade do cliente.

Verifique a autenticidade deste documento em www.lct.poli.usp.br utilizando o código **SFQW-OYYU-OHUT-MMIB**

Escola Politécnica da Universidade de São Paulo
Departamento de Engenharia de Minas e de Petróleo
Laboratório de Caracterização Tecnológica
Av. Prof. Mello Moraes, 2373 CEP 05508-030 São Paulo - SP www.lct.poli.usp.br
Tel: 11 3091-5151 e-mail: lct@lct.poli.usp.br

DIFRATOGRAMA DE RAIOS X

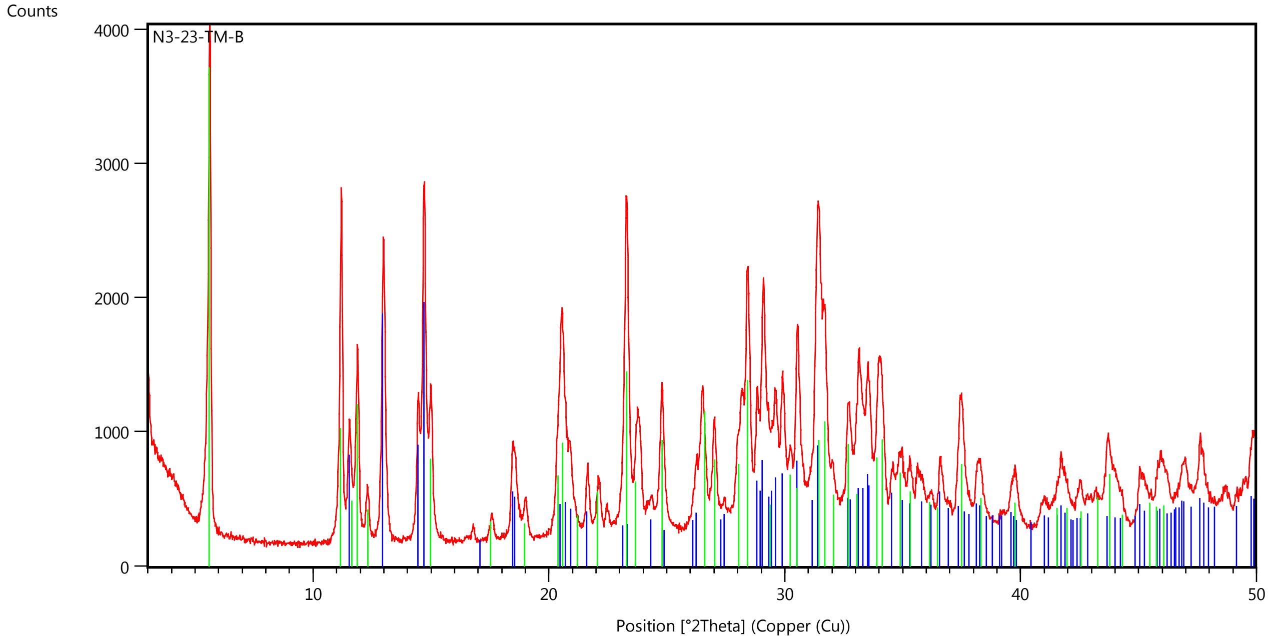

FASES IDENTIFICADAS

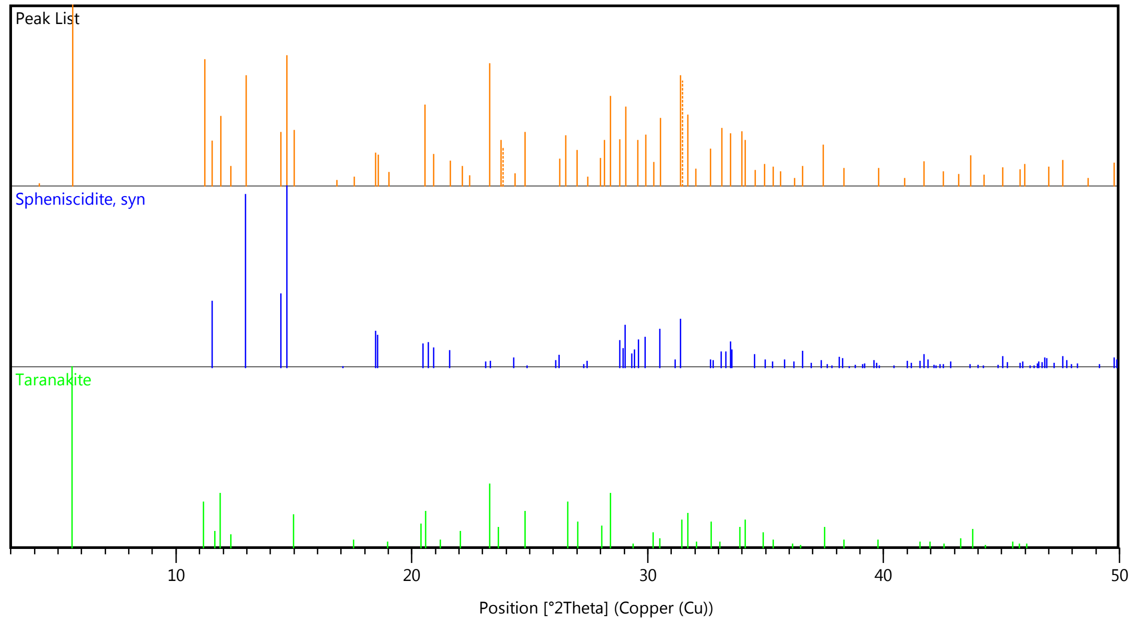

Supplement: S3 File — Reports issued by the Laboratório de Caracterização Tecnológica, Departamento de Engenharia de Minas e de Petróleo at the University of São Paulo´s Escola Politécnica, indicating mineral identification of spelothems samples using the powder method and a Panalytical X-ray diffractometer. (PDF) [file pone.0267870.s007.pdf]
