# Supplementary material for: Bats as ecosystem engineers in iron ore caves in the Carajás National Forest, Brazilian Amazonia
Source: PLoS One. 2023 May 11;18(5):e0267870. doi: 10.1371/journal.pone.0267870 (PMC10174506; doi:10.1371/journal.pone.0267870)
Supplement: S4 File — Reports issued by the Laboratório de Caracterização Tecnológica, Departamento de Engenharia de Minas e de Petróleo at the University of São Paulo´s Escola Politécnica, indicating mineral percentage in samples using the X-ray fluorescence spectrometer. (PDF) [file pone.0267870.s008.pdf]

Escola Politécnica da Universidade de São Paulo
Departamento de Engenharia de Minas e de Petróleo
Laboratório de Caracterização Tecnológica
Av. Prof. Mello Moraes, 2373 CEP 05508-030 São Paulo - SP www.lct.poli.usp.br
Tel: 11 3091-5151 e-mail: lct@lct.poli.usp.br

# RESULTADOS DE ANÁLISE QUÍMICA

**RELATÓRIO:** FRX 838-19 1 **REQ:** 1399-19 **DATA:** 19/12/2019

**CLIENTE:** Luís Piló

**1. MÉTODO:** Os teores apresentados foram dosados em amostra prensada, na calibração STD-1 (Standardless), relativa a análise sem padrões dos elementos químicos compreendidos entre o flúor e o urânio, em espectrômetro de fluorescência de raios X, marca Malvern Panalytical, modelo Zetium. Os valores foram normalizados a 100%. A Perda ao Fogo (PF) foi realizada a 1.020°C por 2h.

**2. RESULTADOS:**

| N° LCT | 10043 |
|---|---|
| Amostra | N3-23-P1-C1 |
| $Na_2O$ (%) | 0,18 |
| MgO (%) | 0,24 |
| $Al_2O_3$ (%) | 0,20 |
| $SiO_2$ (%) | 0,21 |
| $P_2O_5$ (%) | 11,8 |
| $SO_3$ (%) | 2,99 |
| Cl (%) | 0,25 |
| $K_2O$ (%) | 1,10 |
| CaO (%) | 3,91 |
| $TiO_2$ (%) | 0,03 |
| $Cr_2O_3$ (%) | nd |
| MnO (%) | 0,03 |
| $Fe_2O_3$ (%) | 6,40 |
| NiO (%) | <0,01 |
| CuO (%) | 0,03 |
| ZnO (%) | 0,16 |
| $SeO_2$ (%) | nd |
| Br (%) | 0,05 |
| $Rb_2O$ (%) | <0,01 |
| SrO (%) | <0,01 |
| $ZrO_2$ (%) | nd |
| $MoO_3$ (%) | nd |
| BaO (%) | nd |
| PF (%) | 72,4 |

0,01% - limite de quantificação FRX
nd - não detectado
obs 1: Teores de Fe, Ni, Cu, P, Zn e Rb dosados por ICP OES (relatório de ICP 179-19).

Executado por: Dra. Gislayne Kelmer - CRQ 04165656-4ªR (19/12/2019 11:52 BRT)
Revisado por: Saulo Colenci - CRQ 04262337-4ªR (19/12/2019 11:59 BRT)

Profa. Dra. Carina Ulsen
Coordenadora do LCT - Poli/USP

NOTA: Os resultados expostos acima referem-se apenas à(s) amostra(s) enviada(s) ao LCT; a representatividade da(s) mesma(s) é de inteira responsabilidade do cliente.

Verifique a autenticidade deste documento em www.lct.poli.usp.br utilizando o código **WHQA-BJYK-ISYU-EYRB**

Escola Politécnica da Universidade de São Paulo
Departamento de Engenharia de Minas e de Petróleo
Laboratório de Caracterização Tecnológica
Av. Prof. Mello Moraes, 2373 CEP 05508-030 São Paulo - SP www.lct.poli.usp.br
Tel: 11 3091-5151 e-mail: lct@lct.poli.usp.br

# RESULTADOS DE ANÁLISE QUÍMICA

**RELATÓRIO:** FRX 838-19 2 **REQ:** 1399-19 **DATA:** 19/12/2019

**CLIENTE:** Luís Piló

**1. MÉTODO:** Os teores apresentados foram dosados em amostra prensada, na calibração STD-1 (Standardless), relativa a análise sem padrões dos elementos químicos compreendidos entre o flúor e o urânio, em espectrômetro de fluorescência de raios X, marca Malvern Panalytical, modelo Zetium. Os valores foram normalizados a 100%. A Perda ao Fogo (PF) foi realizada a 1.020°C por 2h.

**2. RESULTADOS:**

| N° LCT | 10044 |
|---|---|
| Amostra | N3-23-P1-C2 |
| $Na_2O$ (%) | 0,19 |
| MgO (%) | 0,25 |
| $Al_2O_3$ (%) | 0,15 |
| $SiO_2$ (%) | 0,19 |
| $P_2O_5$ (%) | 10,7 |
| $SO_3$ (%) | 3,26 |
| Cl (%) | 0,31 |
| $K_2O$ (%) | 1,30 |
| CaO (%) | 5,30 |
| $TiO_2$ (%) | 0,03 |
| $Cr_2O_3$ (%) | nd |
| MnO (%) | 0,03 |
| $Fe_2O_3$ (%) | 4,30 |
| NiO (%) | <0,01 |
| CuO (%) | 0,03 |
| ZnO (%) | 0,15 |
| $SeO_2$ (%) | nd |
| Br (%) | 0,07 |
| $Rb_2O$ (%) | <0,01 |
| SrO (%) | <0,01 |
| $ZrO_2$ (%) | nd |
| $MoO_3$ (%) | nd |
| BaO (%) | nd |
| PF (%) | 73,7 |

0,01% - limite de quantificação FRX
nd - não detectado
obs 1: Teores de Fe, Ni, Cu, P, Zn e Rb dosados por ICP OES (relatório de ICP 179-19).

Executado por: Dra. Gislayne Kelmer - CRQ 04165656-4ªR (19/12/2019 11:52 BRT)
Revisado por: Saulo Colenci - CRQ 04262337-4ªR (19/12/2019 11:59 BRT)

Profa. Dra. Carina Ulsen
Coordenadora do LCT - Poli/USP

NOTA: Os resultados expostos acima referem-se apenas à(s) amostra(s) enviada(s) ao LCT; a representatividade da(s) mesma(s) é de inteira responsabilidade do cliente.

Verifique a autenticidade deste documento em www.lct.poli.usp.br utilizando o código **YHQC-CJYK-VZYU-QPRB**

Escola Politécnica da Universidade de São Paulo
Departamento de Engenharia de Minas e de Petróleo
Laboratório de Caracterização Tecnológica
Av. Prof. Mello Moraes, 2373 CEP 05508-030 São Paulo - SP www.lct.poli.usp.br
Tel: 11 3091-5151 e-mail: lct@lct.poli.usp.br

# RESULTADOS DE ANÁLISE QUÍMICA

**RELATÓRIO:** FRX 838-19 3 **REQ:** 1399-19 **DATA:** 19/12/2019

**CLIENTE:** Luís Piló

**1. MÉTODO:** Os teores apresentados foram dosados em amostra prensada, na calibração STD-1 (Standardless), relativa a análise sem padrões dos elementos químicos compreendidos entre o flúor e o urânio, em espectrômetro de fluorescência de raios X, marca Malvern Panalytical, modelo Zetium. Os valores foram normalizados a 100%. A Perda ao Fogo (PF) foi realizada a 1.020°C por 2h.

**2. RESULTADOS:**

| N° LCT | 10045 |
|---|---|
| Amostra | N3-23-P1-C3 |
| $Na_2O$ (%) | 0,20 |
| MgO (%) | 0,29 |
| $Al_2O_3$ (%) | 0,18 |
| $SiO_2$ (%) | 0,26 |
| $P_2O_5$ (%) | 5,60 |
| $SO_3$ (%) | 3,95 |
| Cl (%) | 0,32 |
| $K_2O$ (%) | 1,39 |
| CaO (%) | 4,83 |
| $TiO_2$ (%) | 0,02 |
| $Cr_2O_3$ (%) | nd |
| MnO (%) | 0,05 |
| $Fe_2O_3$ (%) | 2,80 |
| NiO (%) | <0,01 |
| CuO (%) | 0,05 |
| ZnO (%) | 0,21 |
| $SeO_2$ (%) | nd |
| Br (%) | 0,06 |
| $Rb_2O$ (%) | <0,01 |
| SrO (%) | <0,01 |
| $ZrO_2$ (%) | nd |
| $MoO_3$ (%) | nd |
| BaO (%) | nd |
| PF (%) | 79,4 |

0,01% - limite de quantificação FRX
nd - não detectado
obs 1: Teores de Fe, Ni, Cu, P, Zn e Rb dosados por ICP OES (relatório de ICP 179-19).

Executado por: Dra. Gislayne Kelmer - CRQ 04165656-4ªR (19/12/2019 11:52 BRT)
Revisado por: Saulo Colenci - CRQ 04262337-4ªR (19/12/2019 11:59 BRT)

Profa. Dra. Carina Ulsen
Coordenadora do LCT - Poli/USP

NOTA: Os resultados expostos acima referem-se apenas à(s) amostra(s) enviada(s) ao LCT; a representatividade da(s) mesma(s) é de inteira responsabilidade do cliente.

Verifique a autenticidade deste documento em www.lct.poli.usp.br utilizando o código **OHQE-XKYK-INYU-ASRB**

Escola Politécnica da Universidade de São Paulo
Departamento de Engenharia de Minas e de Petróleo
Laboratório de Caracterização Tecnológica
Av. Prof. Mello Moraes, 2373 CEP 05508-030 São Paulo - SP www.lct.poli.usp.br
Tel: 11 3091-5151 e-mail: lct@lct.poli.usp.br

# RESULTADOS DE ANÁLISE QUÍMICA

**RELATÓRIO:** FRX 838-19 4 **REQ:** 1399-19 **DATA:** 19/12/2019

**CLIENTE:** Luís Piló

**1. MÉTODO:** Os teores apresentados foram dosados em amostra prensada, na calibração STD-1 (Standardless), relativa a análise sem padrões dos elementos químicos compreendidos entre o flúor e o urânio, em espectrômetro de fluorescência de raios X, marca Malvern Panalytical, modelo Zetium. Os valores foram normalizados a 100%. A Perda ao Fogo (PF) foi realizada a 1.020°C por 2h.

**2. RESULTADOS:**

| N° LCT | 10046 |
|---|---|
| Amostra | N3-23-P1-C4 |
| $Na_2O$ (%) | 0,20 |
| MgO (%) | 0,27 |
| $Al_2O_3$ (%) | 0,26 |
| $SiO_2$ (%) | 0,31 |
| $P_2O_5$ (%) | 17,8 |
| $SO_3$ (%) | 3,43 |
| Cl (%) | 0,29 |
| $K_2O$ (%) | 1,31 |
| CaO (%) | 12,1 |
| $TiO_2$ (%) | 0,04 |
| $Cr_2O_3$ (%) | nd |
| MnO (%) | 0,09 |
| $Fe_2O_3$ (%) | 5,70 |
| NiO (%) | <0,01 |
| CuO (%) | 0,06 |
| ZnO (%) | 0,29 |
| $SeO_2$ (%) | nd |
| Br (%) | 0,05 |
| $Rb_2O$ (%) | <0,01 |
| SrO (%) | 0,01 |
| $ZrO_2$ (%) | nd |
| $MoO_3$ (%) | nd |
| BaO (%) | nd |
| PF (%) | 57,8 |

0,01% - limite de quantificação FRX
nd - não detectado
obs 1: Teores de Fe, Ni, Cu, P, Zn e Rb dosados por ICP OES (relatório de ICP 179-19).

Executado por: Dra. Gislayne Kelmer - CRQ 04165656-4ªR (19/12/2019 11:52 BRT)
Revisado por: Saulo Colenci - CRQ 04262337-4ªR (19/12/2019 11:59 BRT)

Profa. Dra. Carina Ulsen
Coordenadora do LCT - Poli/USP

NOTA: Os resultados expostos acima referem-se apenas à(s) amostra(s) enviada(s) ao LCT; a representatividade da(s) mesma(s) é de inteira responsabilidade do cliente.

Verifique a autenticidade deste documento em www.lct.poli.usp.br utilizando o código **QHQG-JKYK-QGYU-SMRB**

# RESULTADOS DE ANÁLISE QUÍMICA

**RELATÓRIO:** FRX 838-19 5 **REQ:** 1399-19 **DATA:** 19/12/2019

**CLIENTE:** Luís Piló

**1. MÉTODO:** Os teores apresentados foram dosados em amostra prensada, na calibração STD-1 (Standardless), relativa a análise sem padrões dos elementos químicos compreendidos entre o flúor e o urânio, em espectrômetro de fluorescência de raios X, marca Malvern Panalytical, modelo Zetium. Os valores foram normalizados a 100%. A Perda ao Fogo (PF) foi realizada a 1.020°C por 2h.

**2. RESULTADOS:**

| N° LCT | 10047 |
|---|---|
| Amostra | N3-23-P1-C5 |
| $Na_2O$ (%) | 0,19 |
| MgO (%) | 0,30 |
| $Al_2O_3$ (%) | 0,20 |
| $SiO_2$ (%) | 0,35 |
| $P_2O_5$ (%) | 4,40 |
| $SO_3$ (%) | 2,59 |
| Cl (%) | 0,35 |
| $K_2O$ (%) | 1,35 |
| CaO (%) | 3,25 |
| $TiO_2$ (%) | 0,03 |
| $Cr_2O_3$ (%) | nd |
| MnO (%) | 0,07 |
| $Fe_2O_3$ (%) | 3,40 |
| NiO (%) | <0,01 |
| CuO (%) | 0,08 |
| ZnO (%) | 0,43 |
| $SeO_2$ (%) | nd |
| Br (%) | 0,06 |
| $Rb_2O$ (%) | 0,01 |
| SrO (%) | <0,01 |
| $ZrO_2$ (%) | nd |
| $MoO_3$ (%) | nd |
| BaO (%) | nd |
| PF (%) | 82,9 |

0,01% - limite de quantificação FRX
nd - não detectado
obs 1: Teores de Fe, Ni, Cu, P, Zn e Rb dosados por ICP OES (relatório de ICP 179-19).

Executado por: Dra. Gislayne Kelmer - CRQ 04165656-4ªR (19/12/2019 11:52 BRT)
Revisado por: Saulo Colenci - CRQ 04262337-4ªR (19/12/2019 11:59 BRT)

Profa. Dra. Carina Ulsen
Coordenadora do LCT - Poli/USP

NOTA: Os resultados expostos acima referem-se apenas à(s) amostra(s) enviada(s) ao LCT; a representatividade da(s) mesma(s) é de inteira responsabilidade do cliente.

Verifique a autenticidade deste documento em www.lct.poli.usp.br utilizando o código **WHQI-LKYK-IUYU-GKRB**

Escola Politécnica da Universidade de São Paulo
Departamento de Engenharia de Minas e de Petróleo
Laboratório de Caracterização Tecnológica
Av. Prof. Mello Moraes, 2373 CEP 05508-030 São Paulo - SP www.lct.poli.usp.br
Tel: 11 3091-5151 e-mail: lct@lct.poli.usp.br

# RESULTADOS DE ANÁLISE QUÍMICA

**RELATÓRIO:** FRX 838-19 6 **REQ:** 1399-19 **DATA:** 19/12/2019

**CLIENTE:** Luís Piló

**1. MÉTODO:** Os teores apresentados foram dosados em amostra prensada, na calibração STD-1 (Standardless), relativa a análise sem padrões dos elementos químicos compreendidos entre o flúor e o urânio, em espectrômetro de fluorescência de raios X, marca Malvern Panalytical, modelo Zetium. Os valores foram normalizados a 100%. A Perda ao Fogo (PF) foi realizada a 1.020°C por 2h.

**2. RESULTADOS:**

| N° LCT | 10048 |
|---|---|
| Amostra | N3-23-P1-C6 |
| $Na_2O$ (%) | 0,13 |
| MgO (%) | 0,20 |
| $Al_2O_3$ (%) | 0,25 |
| $SiO_2$ (%) | 0,47 |
| $P_2O_5$ (%) | 13,9 |
| $SO_3$ (%) | 6,03 |
| Cl (%) | 0,18 |
| $K_2O$ (%) | 0,95 |
| CaO (%) | 10,4 |
| $TiO_2$ (%) | 0,04 |
| $Cr_2O_3$ (%) | nd |
| MnO (%) | 0,07 |
| $Fe_2O_3$ (%) | 4,50 |
| NiO (%) | <0,01 |
| CuO (%) | 0,07 |
| ZnO (%) | 0,31 |
| $SeO_2$ (%) | nd |
| Br (%) | 0,04 |
| $Rb_2O$ (%) | <0,01 |
| SrO (%) | <0,01 |
| $ZrO_2$ (%) | nd |
| $MoO_3$ (%) | nd |
| BaO (%) | nd |
| PF (%) | 62,4 |

0,01% - limite de quantificação FRX
nd - não detectado
obs 1: Teores de Fe, Ni, Cu, P, Zn e Rb dosados por ICP OES (relatório de ICP 179-19).

Executado por: Dra. Gislayne Kelmer - CRQ 04165656-4ªR (19/12/2019 11:52 BRT)
Revisado por: Saulo Colenci - CRQ 04262337-4ªR (19/12/2019 11:59 BRT)

Profa. Dra. Carina Ulsen
Coordenadora do LCT - Poli/USP

NOTA: Os resultados expostos acima referem-se apenas à(s) amostra(s) enviada(s) ao LCT; a representatividade da(s) mesma(s) é de inteira responsabilidade do cliente.

Verifique a autenticidade deste documento em www.lct.poli.usp.br utilizando o código **GHQK-WLYK-YBYU-WBRB**

Escola Politécnica da Universidade de São Paulo
Departamento de Engenharia de Minas e de Petróleo
Laboratório de Caracterização Tecnológica
Av. Prof. Mello Moraes, 2373 CEP 05508-030 São Paulo - SP www.lct.poli.usp.br
Tel: 11 3091-5151 e-mail: lct@lct.poli.usp.br

# RESULTADOS DE ANÁLISE QUÍMICA

**RELATÓRIO:** FRX 838-19 7 **REQ:** 1399-19 **DATA:** 19/12/2019

**CLIENTE:** Luís Piló

**1. MÉTODO:** Os teores apresentados foram dosados em amostra prensada, na calibração STD-1 (Standardless), relativa a análise sem padrões dos elementos químicos compreendidos entre o flúor e o urânio, em espectrômetro de fluorescência de raios X, marca Malvern Panalytical, modelo Zetium. Os valores foram normalizados a 100%. A Perda ao Fogo (PF) foi realizada a 1.020°C por 2h.

**2. RESULTADOS:**

| N° LCT | 10049 |
|---|---|
| Amostra | N3-23-P1-C7 |
| $Na_2O$ (%) | 0,08 |
| MgO (%) | 0,16 |
| $Al_2O_3$ (%) | 0,51 |
| $SiO_2$ (%) | 1,03 |
| $P_2O_5$ (%) | 14,2 |
| $SO_3$ (%) | 1,03 |
| Cl (%) | 0,09 |
| $K_2O$ (%) | 0,63 |
| CaO (%) | 1,23 |
| $TiO_2$ (%) | 0,10 |
| $Cr_2O_3$ (%) | nd |
| MnO (%) | 0,06 |
| $Fe_2O_3$ (%) | 22,3 |
| NiO (%) | <0,01 |
| CuO (%) | 0,15 |
| ZnO (%) | 0,43 |
| $SeO_2$ (%) | nd |
| Br (%) | 0,03 |
| $Rb_2O$ (%) | 0,01 |
| SrO (%) | <0,01 |
| $ZrO_2$ (%) | <0,01 |
| $MoO_3$ (%) | nd |
| BaO (%) | <0,01 |
| PF (%) | 58,0 |

0,01% - limite de quantificação FRX
nd - não detectado
obs 1: Teores de Fe, Ni, Cu, P, Zn e Rb dosados por ICP OES (relatório de ICP 179-19).

Executado por: Dra. Gislayne Kelmer - CRQ 04165656-4ªR (19/12/2019 11:52 BRT)
Revisado por: Saulo Colenci - CRQ 04262337-4ªR (19/12/2019 11:59 BRT)

Profa. Dra. Carina Ulsen
Coordenadora do LCT - Poli/USP

NOTA: Os resultados expostos acima referem-se apenas à(s) amostra(s) enviada(s) ao LCT; a representatividade da(s) mesma(s) é de inteira responsabilidade do cliente.

Verifique a autenticidade deste documento em www.lct.poli.usp.br utilizando o código **OHQM-ULYK-THYU-YRRB**

Escola Politécnica da Universidade de São Paulo
Departamento de Engenharia de Minas e de Petróleo
Laboratório de Caracterização Tecnológica
Av. Prof. Mello Moraes, 2373 CEP 05508-030 São Paulo - SP www.lct.poli.usp.br
Tel: 11 3091-5151 e-mail: lct@lct.poli.usp.br

# RESULTADOS DE ANÁLISE QUÍMICA

**RELATÓRIO:** FRX 838-19 8 **REQ:** 1399-19 **DATA:** 19/12/2019

**CLIENTE:** Luís Piló

**1. MÉTODO:** Os teores apresentados foram dosados em amostra prensada, na calibração STD-1 (Standardless), relativa a análise sem padrões dos elementos químicos compreendidos entre o flúor e o urânio, em espectrômetro de fluorescência de raios X, marca Malvern Panalytical, modelo Zetium. Os valores foram normalizados a 100%. A Perda ao Fogo (PF) foi realizada a 1.020°C por 2h.

**2. RESULTADOS:**

| N° LCT | 10050 |
|---|---|
| Amostra | N3-23-P2-C1 |
| $Na_2O$ (%) | 0,01 |
| MgO (%) | 0,07 |
| $Al_2O_3$ (%) | 0,64 |
| $SiO_2$ (%) | 3,86 |
| $P_2O_5$ (%) | 6,60 |
| $SO_3$ (%) | 0,54 |
| Cl (%) | 0,10 |
| $K_2O$ (%) | 0,39 |
| CaO (%) | 0,47 |
| $TiO_2$ (%) | 0,16 |
| $Cr_2O_3$ (%) | nd |
| MnO (%) | 0,03 |
| $Fe_2O_3$ (%) | 13,9 |
| NiO (%) | <0,01 |
| CuO (%) | 0,70 |
| ZnO (%) | 0,31 |
| $SeO_2$ (%) | nd |
| Br (%) | 0,04 |
| $Rb_2O$ (%) | 0,03 |
| SrO (%) | <0,01 |
| $ZrO_2$ (%) | <0,01 |
| $MoO_3$ (%) | nd |
| BaO (%) | 0,06 |
| PF (%) | 72,1 |

0,01% - limite de quantificação FRX
nd - não detectado
obs 1: Teores de Fe, Ni, Cu, P, Zn e Rb dosados por ICP OES (relatório de ICP 179-19).

Executado por: Dra. Gislayne Kelmer - CRQ 04165656-4ªR (19/12/2019 11:52 BRT)
Revisado por: Saulo Colenci - CRQ 04262337-4ªR (19/12/2019 11:59 BRT)

Profa. Dra. Carina Ulsen
Coordenadora do LCT - Poli/USP

NOTA: Os resultados expostos acima referem-se apenas à(s) amostra(s) enviada(s) ao LCT; a representatividade da(s) mesma(s) é de inteira responsabilidade do cliente.

Verifique a autenticidade deste documento em www.lct.poli.usp.br utilizando o código **LHQO-WLYK-ZOYU-WGRB**

Escola Politécnica da Universidade de São Paulo
Departamento de Engenharia de Minas e de Petróleo
Laboratório de Caracterização Tecnológica
Av. Prof. Mello Moraes, 2373 CEP 05508-030 São Paulo - SP www.lct.poli.usp.br
Tel: 11 3091-5151 e-mail: lct@lct.poli.usp.br

# RESULTADOS DE ANÁLISE QUÍMICA

**RELATÓRIO:** FRX 838-19 9 **REQ:** 1399-19 **DATA:** 19/12/2019

**CLIENTE:** Luís Piló

**1. MÉTODO:** Os teores apresentados foram dosados em amostra prensada, na calibração STD-1 (Standardless), relativa a análise sem padrões dos elementos químicos compreendidos entre o flúor e o urânio, em espectrômetro de fluorescência de raios X, marca Malvern Panalytical, modelo Zetium. Os valores foram normalizados a 100%. A Perda ao Fogo (PF) foi realizada a 1.020°C por 2h.

**2. RESULTADOS:**

| N° LCT | 10051 |
|---|---|
| Amostra | N3-23-P2-C2 |
| $Na_2O$ (%) | <0,01 |
| MgO (%) | 0,04 |
| $Al_2O_3$ (%) | 0,47 |
| $SiO_2$ (%) | 2,17 |
| $P_2O_5$ (%) | 9,60 |
| $SO_3$ (%) | 0,27 |
| Cl (%) | 0,12 |
| $K_2O$ (%) | 0,49 |
| CaO (%) | 0,29 |
| $TiO_2$ (%) | 0,24 |
| $Cr_2O_3$ (%) | nd |
| MnO (%) | 0,04 |
| $Fe_2O_3$ (%) | 19,7 |
| NiO (%) | <0,01 |
| CuO (%) | 0,74 |
| ZnO (%) | 0,35 |
| $SeO_2$ (%) | 0,01 |
| Br (%) | 0,03 |
| $Rb_2O$ (%) | <0,01 |
| SrO (%) | <0,01 |
| $ZrO_2$ (%) | 0,01 |
| $MoO_3$ (%) | <0,01 |
| BaO (%) | 0,02 |
| PF (%) | 65,4 |

0,01% - limite de quantificação FRX
nd - não detectado
obs 1: Teores de Fe, Ni, Cu, P, Zn e Rb dosados por ICP OES (relatório de ICP 179-19).

Executado por: Dra. Gislayne Kelmer - CRQ 04165656-4ªR (19/12/2019 11:52 BRT)
Revisado por: Saulo Colenci - CRQ 04262337-4ªR (19/12/2019 11:59 BRT)

Profa. Dra. Carina Ulsen
Coordenadora do LCT - Poli/USP

NOTA: Os resultados expostos acima referem-se apenas à(s) amostra(s) enviada(s) ao LCT; a representatividade da(s) mesma(s) é de inteira responsabilidade do cliente.

Verifique a autenticidade deste documento em www.lct.poli.usp.br utilizando o código **FHQQ-TLYK-XVYU-IARB**

Escola Politécnica da Universidade de São Paulo
Departamento de Engenharia de Minas e de Petróleo
Laboratório de Caracterização Tecnológica
Av. Prof. Mello Moraes, 2373 CEP 05508-030 São Paulo - SP www.lct.poli.usp.br
Tel: 11 3091-5151 e-mail: lct@lct.poli.usp.br

# RESULTADOS DE ANÁLISE QUÍMICA

**RELATÓRIO:** FRX 838-19 10 **REQ:** 1399-19 **DATA:** 19/12/2019

**CLIENTE:** Luís Piló

**1. MÉTODO:** Os teores apresentados foram dosados em amostra prensada, na calibração STD-1 (Standardless), relativa a análise sem padrões dos elementos químicos compreendidos entre o flúor e o urânio, em espectrômetro de fluorescência de raios X, marca Malvern Panalytical, modelo Zetium. Os valores foram normalizados a 100%. A Perda ao Fogo (PF) foi realizada a 1.020°C por 2h.

**2. RESULTADOS:**

| N° LCT | 10052 |
|---|---|
| Amostra | N3-23-P2-C3 |
| $Na_2O$ (%) | 0,01 |
| MgO (%) | 0,02 |
| $Al_2O_3$ (%) | 0,34 |
| $SiO_2$ (%) | 1,02 |
| $P_2O_5$ (%) | 6,40 |
| $SO_3$ (%) | 0,19 |
| Cl (%) | 0,09 |
| $K_2O$ (%) | 0,58 |
| CaO (%) | 0,11 |
| $TiO_2$ (%) | 0,10 |
| $Cr_2O_3$ (%) | <0,01 |
| MnO (%) | 0,02 |
| $Fe_2O_3$ (%) | 16,3 |
| NiO (%) | <0,01 |
| CuO (%) | 0,56 |
| ZnO (%) | 0,20 |
| $SeO_2$ (%) | <0,01 |
| Br (%) | 0,03 |
| $Rb_2O$ (%) | 0,01 |
| SrO (%) | nd |
| $ZrO_2$ (%) | <0,01 |
| $MoO_3$ (%) | <0,01 |
| BaO (%) | 0,01 |
| PF (%) | 74,0 |

0,01% - limite de quantificação FRX
nd - não detectado
obs 1: Teores de Fe, Ni, Cu, P, Zn e Rb dosados por ICP OES (relatório de ICP 179-19).

Executado por: Dra. Gislayne Kelmer - CRQ 04165656-4ªR (19/12/2019 11:52 BRT)
Revisado por: Saulo Colenci - CRQ 04262337-4ªR (19/12/2019 11:59 BRT)

Profa. Dra. Carina Ulsen
Coordenadora do LCT - Poli/USP

NOTA: Os resultados expostos acima referem-se apenas à(s) amostra(s) enviada(s) ao LCT; a representatividade da(s) mesma(s) é de inteira responsabilidade do cliente.

Verifique a autenticidade deste documento em www.lct.poli.usp.br utilizando o código **SHQS-HMYK-UCYU-ACRB**

Escola Politécnica da Universidade de São Paulo
Departamento de Engenharia de Minas e de Petróleo
Laboratório de Caracterização Tecnológica
Av. Prof. Mello Moraes, 2373 CEP 05508-030 São Paulo - SP www.lct.poli.usp.br
Tel: 11 3091-5151 e-mail: lct@lct.poli.usp.br

# RESULTADOS DE ANÁLISE QUÍMICA

**RELATÓRIO:** FRX 838-19 11 **REQ:** 1399-19 **DATA:** 19/12/2019

**CLIENTE:** Luís Piló

**1. MÉTODO:** Os teores apresentados foram dosados em amostra prensada, na calibração STD-1 (Standardless), relativa a análise sem padrões dos elementos químicos compreendidos entre o flúor e o urânio, em espectrômetro de fluorescência de raios X, marca Malvern Panalytical, modelo Zetium. Os valores foram normalizados a 100%. A Perda ao Fogo (PF) foi realizada a 1.020°C por 2h.

**2. RESULTADOS:**

| N° LCT | 10053 |
|---|---|
| Amostra | M2-99-C1 |
| $Na_2O$ (%) | 0,44 |
| MgO (%) | 0,35 |
| $Al_2O_3$ (%) | 0,06 |
| $SiO_2$ (%) | 0,14 |
| $P_2O_5$ (%) | 5,40 |
| $SO_3$ (%) | 2,46 |
| Cl (%) | 0,81 |
| $K_2O$ (%) | 1,74 |
| CaO (%) | 0,55 |
| $TiO_2$ (%) | <0,01 |
| $Cr_2O_3$ (%) | nd |
| MnO (%) | 0,02 |
| $Fe_2O_3$ (%) | 1,20 |
| NiO (%) | <0,01 |
| CuO (%) | 0,13 |
| ZnO (%) | 0,31 |
| $SeO_2$ (%) | nd |
| Br (%) | 0,08 |
| $Rb_2O$ (%) | <0,01 |
| SrO (%) | <0,01 |
| $ZrO_2$ (%) | nd |
| $MoO_3$ (%) | nd |
| BaO (%) | nd |
| PF (%) | 86,3 |

0,01% - limite de quantificação FRX
nd - não detectado
obs 1: Teores de Fe, Ni, Cu, P, Zn e Rb dosados por ICP OES (relatório de ICP 179-19).

Executado por: Dra. Gislayne Kelmer - CRQ 04165656-4ªR (19/12/2019 11:52 BRT)
Revisado por: Saulo Colenci - CRQ 04262337-4ªR (19/12/2019 11:59 BRT)

Profa. Dra. Carina Ulsen
Coordenadora do LCT - Poli/USP

NOTA: Os resultados expostos acima referem-se apenas à(s) amostra(s) enviada(s) ao LCT; a representatividade da(s) mesma(s) é de inteira responsabilidade do cliente.

Verifique a autenticidade deste documento em www.lct.poli.usp.br utilizando o código **HHQU-QMYK-SJYU-WDRB**

Escola Politécnica da Universidade de São Paulo
Departamento de Engenharia de Minas e de Petróleo
Laboratório de Caracterização Tecnológica
Av. Prof. Mello Moraes, 2373 CEP 05508-030 São Paulo - SP www.lct.poli.usp.br
Tel: 11 3091-5151 e-mail: lct@lct.poli.usp.br

# RESULTADOS DE ANÁLISE QUÍMICA

**RELATÓRIO:** FRX 838-19 12 **REQ:** 1399-19 **DATA:** 19/12/2019

**CLIENTE:** Luís Piló

**1. MÉTODO:** Os teores apresentados foram dosados em amostra prensada, na calibração STD-1 (Standardless), relativa a análise sem padrões dos elementos químicos compreendidos entre o flúor e o urânio, em espectrômetro de fluorescência de raios X, marca Malvern Panalytical, modelo Zetium. Os valores foram normalizados a 100%. A Perda ao Fogo (PF) foi realizada a 1.020°C por 2h.

**2. RESULTADOS:**

| N° LCT | 10054 |
|---|---|
| Amostra | M2-99-C2 |
| $Na_2O$ (%) | 0,26 |
| MgO (%) | 1,12 |
| $Al_2O_3$ (%) | 0,19 |
| $SiO_2$ (%) | 0,43 |
| $P_2O_5$ (%) | 11,9 |
| $SO_3$ (%) | 3,33 |
| Cl (%) | 0,58 |
| $K_2O$ (%) | 1,72 |
| CaO (%) | 1,92 |
| $TiO_2$ (%) | 0,02 |
| $Cr_2O_3$ (%) | <0,01 |
| MnO (%) | 0,08 |
| $Fe_2O_3$ (%) | 4,80 |
| NiO (%) | <0,01 |
| CuO (%) | 0,38 |
| ZnO (%) | 1,06 |
| $SeO_2$ (%) | <0,01 |
| Br (%) | 0,09 |
| $Rb_2O$ (%) | <0,01 |
| SrO (%) | <0,01 |
| $ZrO_2$ (%) | nd |
| $MoO_3$ (%) | nd |
| BaO (%) | nd |
| PF (%) | 72,1 |

0,01% - limite de quantificação FRX
nd - não detectado
obs 1: Teores de Fe, Ni, Cu, P, Zn e Rb dosados por ICP OES (relatório de ICP 179-19).

Executado por: Dra. Gislayne Kelmer - CRQ 04165656-4ªR (19/12/2019 11:52 BRT)
Revisado por: Saulo Colenci - CRQ 04262337-4ªR (19/12/2019 11:59 BRT)

Profa. Dra. Carina Ulsen
Coordenadora do LCT - Poli/USP

NOTA: Os resultados expostos acima referem-se apenas à(s) amostra(s) enviada(s) ao LCT; a representatividade da(s) mesma(s) é de inteira responsabilidade do cliente.

Verifique a autenticidade deste documento em www.lct.poli.usp.br utilizando o código **JHQW-TMYK-NQYU-ODRB**

Escola Politécnica da Universidade de São Paulo
Departamento de Engenharia de Minas e de Petróleo
Laboratório de Caracterização Tecnológica
Av. Prof. Mello Moraes, 2373 CEP 05508-030 São Paulo - SP www.lct.poli.usp.br
Tel: 11 3091-5151 e-mail: lct@lct.poli.usp.br

# RESULTADOS DE ANÁLISE QUÍMICA

**RELATÓRIO:** FRX 838-19 13 **REQ:** 1399-19 **DATA:** 19/12/2019

**CLIENTE:** Luís Piló

**1. MÉTODO:** Os teores apresentados foram dosados em amostra prensada, na calibração STD-1 (Standardless), relativa a análise sem padrões dos elementos químicos compreendidos entre o flúor e o urânio, em espectrômetro de fluorescência de raios X, marca Malvern Panalytical, modelo Zetium. Os valores foram normalizados a 100%. A Perda ao Fogo (PF) foi realizada a 1.020°C por 2h.

**2. RESULTADOS:**

| N° LCT | 10055 |
|---|---|
| Amostra | M2-99-C3 |
| $Na_2O$ (%) | 0,23 |
| MgO (%) | 0,11 |
| $Al_2O_3$ (%) | 0,11 |
| $SiO_2$ (%) | 0,32 |
| $P_2O_5$ (%) | 6,20 |
| $SO_3$ (%) | 2,94 |
| Cl (%) | 0,45 |
| $K_2O$ (%) | 1,27 |
| CaO (%) | 1,43 |
| $TiO_2$ (%) | 0,01 |
| $Cr_2O_3$ (%) | nd |
| MnO (%) | 0,03 |
| $Fe_2O_3$ (%) | 5,20 |
| NiO (%) | <0,01 |
| CuO (%) | 0,49 |
| ZnO (%) | 1,13 |
| $SeO_2$ (%) | <0,01 |
| Br (%) | 0,09 |
| $Rb_2O$ (%) | <0,01 |
| SrO (%) | <0,01 |
| $ZrO_2$ (%) | nd |
| $MoO_3$ (%) | nd |
| BaO (%) | nd |
| PF (%) | 80,0 |

0,01% - limite de quantificação FRX
nd - não detectado
obs 1: Teores de Fe, Ni, Cu, P, Zn e Rb dosados por ICP OES (relatório de ICP 179-19).

Executado por: Dra. Gislayne Kelmer - CRQ 04165656-4ªR (19/12/2019 11:52 BRT)
Revisado por: Saulo Colenci - CRQ 04262337-4ªR (19/12/2019 11:59 BRT)

Profa. Dra. Carina Ulsen
Coordenadora do LCT - Poli/USP

NOTA: Os resultados expostos acima referem-se apenas à(s) amostra(s) enviada(s) ao LCT; a representatividade da(s) mesma(s) é de inteira responsabilidade do cliente.

Verifique a autenticidade deste documento em www.lct.poli.usp.br utilizando o código **YHQY-KMYK-CWYU-EMRB**

Escola Politécnica da Universidade de São Paulo
Departamento de Engenharia de Minas e de Petróleo
Laboratório de Caracterização Tecnológica
Av. Prof. Mello Moraes, 2373 CEP 05508-030 São Paulo - SP www.lct.poli.usp.br
Tel: 11 3091-5151 e-mail: lct@lct.poli.usp.br

# RESULTADOS DE ANÁLISE QUÍMICA

**RELATÓRIO:** FRX 838-19 14 **REQ:** 1399-19 **DATA:** 19/12/2019

**CLIENTE:** Luís Piló

**1. MÉTODO:** Os teores apresentados foram dosados em amostra prensada, na calibração STD-1 (Standardless), relativa a análise sem padrões dos elementos químicos compreendidos entre o flúor e o urânio, em espectrômetro de fluorescência de raios X, marca Malvern Panalytical, modelo Zetium. Os valores foram normalizados a 100%. A Perda ao Fogo (PF) foi realizada a 1.020°C por 2h.

**2. RESULTADOS:**

| N° LCT | 10056 |
|---|---|
| Amostra | M2-99-C4 |
| $Na_2O$ (%) | 0,02 |
| MgO (%) | <0,01 |
| $Al_2O_3$ (%) | 0,02 |
| $SiO_2$ (%) | 0,07 |
| $P_2O_5$ (%) | 14,1 |
| $SO_3$ (%) | 0,20 |
| Cl (%) | 0,04 |
| $K_2O$ (%) | 0,19 |
| CaO (%) | 0,05 |
| $TiO_2$ (%) | <0,01 |
| $Cr_2O_3$ (%) | nd |
| MnO (%) | <0,01 |
| $Fe_2O_3$ (%) | 19,9 |
| NiO (%) | <0,01 |
| CuO (%) | 0,54 |
| ZnO (%) | 0,89 |
| $SeO_2$ (%) | nd |
| Br (%) | <0,01 |
| $Rb_2O$ (%) | <0,01 |
| SrO (%) | <0,01 |
| $ZrO_2$ (%) | nd |
| $MoO_3$ (%) | nd |
| BaO (%) | <0,01 |
| PF (%) | 64,0 |

0,01% - limite de quantificação FRX
nd - não detectado
obs 1: Teores de Fe, Ni, Cu, P, Zn e Rb dosados por ICP OES (relatório de ICP 179-19).

Executado por: Dra. Gislayne Kelmer - CRQ 04165656-4ªR (19/12/2019 11:52 BRT)
Revisado por: Saulo Colenci - CRQ 04262337-4ªR (19/12/2019 11:59 BRT)

Profa. Dra. Carina Ulsen
Coordenadora do LCT - Poli/USP

NOTA: Os resultados expostos acima referem-se apenas à(s) amostra(s) enviada(s) ao LCT; a representatividade da(s) mesma(s) é de inteira responsabilidade do cliente.

Verifique a autenticidade deste documento em www.lct.poli.usp.br utilizando o código **BHQA-INYL-ODYU-OJRB**

Escola Politécnica da Universidade de São Paulo
Departamento de Engenharia de Minas e de Petróleo
Laboratório de Caracterização Tecnológica
Av. Prof. Mello Moraes, 2373 CEP 05508-030 São Paulo - SP www.lct.poli.usp.br
Tel: 11 3091-5151 e-mail: lct@lct.poli.usp.br

# RESULTADOS DE ANÁLISE QUÍMICA

**RELATÓRIO:** FRX 839-19 **REQ:** 1400-19 **DATA:** 19/12/2019

**CLIENTE:** Luís Piló

**1. MÉTODO:** Os teores apresentados foram dosados em amostra prensada, na calibração STD-1 (Standardless), relativa a análise sem padrões dos elementos químicos compreendidos entre o flúor e o urânio, em espectrômetro de fluorescência de raios X, marca Malvern Panalytical, modelo Zetium. Os valores foram normalizados a 100%. A Perda ao Fogo (PF) foi realizada a 1.020°C por 2h.

**2. RESULTADOS:**

| N° LCT | 10057 | 10058 | 10059 | 10060 |
|---|---|---|---|---|
| Amostra | S11B-94-C1 | S11B-94-C2 | S11B-94-C3 | S11B-94-C5 |
| $Na_2O$ (%) | 0,02 | nd | nd | 0,04 |
| MgO (%) | 0,04 | nd | nd | 0,16 |
| $Al_2O_3$ (%) | 3,07 | 25,7 | 33,0 | 2,38 |
| $SiO_2$ (%) | 1,07 | 0,25 | 0,08 | 0,67 |
| $P_2O_5$ (%) | 22,9 | 54,3 | 60,0 | 18,7 |
| $SO_3$ (%) | 0,50 | 0,17 | 0,10 | 0,70 |
| Cl (%) | 0,03 | nd | nd | 0,08 |
| $K_2O$ (%) | 1,83 | 5,18 | 0,08 | 4,57 |
| CaO (%) | 0,04 | 0,03 | 0,03 | 0,15 |
| $TiO_2$ (%) | 0,22 | nd | nd | 0,41 |
| $Cr_2O_3$ (%) | 0,01 | nd | nd | 0,01 |
| MnO (%) | 0,03 | 0,01 | nd | 0,14 |
| $Fe_2O_3$ (%) | 35,4 | 2,27 | 2,59 | 23,5 |
| NiO (%) | nd | nd | nd | nd |
| CuO (%) | 0,44 | 0,01 | 0,04 | 1,62 |
| ZnO (%) | 0,14 | <0,01 | 0,01 | 0,15 |
| $SeO_2$ (%) | nd | nd | nd | nd |
| Br (%) | 0,10 | nd | nd | 0,10 |
| $Rb_2O$ (%) | 0,02 | <0,01 | nd | 0,03 |
| SrO (%) | nd | nd | nd | nd |
| $ZrO_2$ (%) | <0,01 | nd | nd | 0,01 |
| $MoO_3$ (%) | nd | nd | nd | <0,01 |
| BaO (%) | 0,45 | nd | 0,03 | 0,13 |
| PF (%) | 33,7 | 12,0 | 4,04 | 46,4 |

| N° LCT | 10061 | 10062 | 10063 | 10064 |
|---|---|---|---|---|
| Amostra | S11A-36-TVG | S11A-36-MVG | S11A-36-BVG | S11A-36-MVF |
| $Na_2O$ (%) | 0,02 | nd | 0,05 | nd |
| MgO (%) | 0,04 | 0,02 | 0,07 | 0,02 |
| $Al_2O_3$ (%) | 0,20 | 0,38 | 0,66 | 0,83 |
| $SiO_2$ (%) | 0,27 | 1,03 | 3,02 | 1,65 |
| $P_2O_5$ (%) | 0,56 | 1,72 | 8,00 | 1,25 |

Escola Politécnica da Universidade de São Paulo
Departamento de Engenharia de Minas e de Petróleo
Laboratório de Caracterização Tecnológica
Av. Prof. Mello Moraes, 2373 CEP 05508-030 São Paulo - SP www.lct.poli.usp.br
Tel: 11 3091-5151 e-mail: lct@lct.poli.usp.br

| N° LCT | 10061 | 10062 | 10063 | 10064 |
|---|---|---|---|---|
| Amostra | S11A-36-TVG | S11A-36-MVG | S11A-36-BVG | S11A-36-MVF |
| $SO_3$ (%) | 1,08 | 1,14 | 0,69 | 0,21 |
| Cl (%) | 0,05 | 0,03 | 0,04 | 0,01 |
| $K_2O$ (%) | 0,11 | 0,11 | 0,18 | 0,04 |
| CaO (%) | 0,13 | 0,31 | 0,36 | 0,03 |
| $TiO_2$ (%) | 0,04 | 0,19 | 0,52 | 0,47 |
| $Cr_2O_3$ (%) | nd | nd | 0,01 | 0,02 |
| MnO (%) | <0,01 | 0,01 | 0,02 | 0,01 |
| $Fe_2O_3$ (%) | 2,25 | 23,0 | 61,8 | 69,2 |
| NiO (%) | nd | nd | nd | nd |
| CuO (%) | 0,03 | 0,04 | 0,04 | 0,01 |
| ZnO (%) | 0,02 | <0,01 | 0,02 | nd |
| $SeO_2$ (%) | nd | nd | nd | nd |
| Br (%) | <0,01 | <0,01 | <0,01 | nd |
| $Rb_2O$ (%) | nd | nd | nd | nd |
| SrO (%) | <0,01 | nd | nd | nd |
| $ZrO_2$ (%) | nd | <0,01 | 0,02 | 0,02 |
| $MoO_3$ (%) | nd | nd | nd | nd |
| BaO (%) | nd | nd | nd | nd |
| PF (%) | 95,2 | 72,0 | 24,5 | 26,3 |

0,01% - limite de quantificação FRX
nd - não detectado

Executado por: Dra. Gislayne Kelmer - CRQ 04165656-4ªR (19/12/2019 11:52 BRT)
Revisado por: Saulo Colenci - CRQ 04262337-4ªR (19/12/2019 12:01 BRT)

Profa. Dra. Carina Ulsen
Coordenadora do LCT - Poli/USP

NOTA: Os resultados expostos acima referem-se apenas à(s) amostra(s) enviada(s) ao LCT; a representatividade da(s) mesma(s) é de inteira responsabilidade do cliente.

Verifique a autenticidade deste documento em www.lct.poli.usp.br utilizando o código **JJQC-JGYL-XAYU-YORB**

Escola Politécnica da Universidade de São Paulo
Departamento de Engenharia de Minas e de Petróleo
Laboratório de Caracterização Tecnológica
Av. Prof. Mello Moraes, 2373 CEP 05508-030 São Paulo - SP www.lct.poli.usp.br
Tel: 11 3091-5151 e-mail: lct@lct.poli.usp.br

# RESULTADOS DE ANÁLISE QUÍMICA

**RELATÓRIO:** FRX 839-19 1 **REQ:** 1400-19 **DATA:** 19/12/2019

**CLIENTE:** Luís Piló

**1. MÉTODO:** Os teores apresentados foram dosados em amostra prensada, na calibração STD-1 (Standardless), relativa a análise sem padrões dos elementos químicos compreendidos entre o flúor e o urânio, em espectrômetro de fluorescência de raios X, marca Malvern Panalytical, modelo Zetium. Os valores foram normalizados a 100%. A Perda ao Fogo (PF) foi realizada a 1.020°C por 2h.

**2. RESULTADOS:**

| N° LCT | 10057 |
|---|---|
| Amostra | S11B-94-C1 |
| $Na_2O$ (%) | 0,02 |
| MgO (%) | 0,04 |
| $Al_2O_3$ (%) | 3,07 |
| $SiO_2$ (%) | 1,07 |
| $P_2O_5$ (%) | 22,9 |
| $SO_3$ (%) | 0,50 |
| Cl (%) | 0,03 |
| $K_2O$ (%) | 1,83 |
| CaO (%) | 0,04 |
| $TiO_2$ (%) | 0,22 |
| $Cr_2O_3$ (%) | 0,01 |
| MnO (%) | 0,03 |
| $Fe_2O_3$ (%) | 35,4 |
| NiO (%) | nd |
| CuO (%) | 0,44 |
| ZnO (%) | 0,14 |
| $SeO_2$ (%) | nd |
| Br (%) | 0,10 |
| $Rb_2O$ (%) | 0,02 |
| SrO (%) | nd |
| $ZrO_2$ (%) | <0,01 |
| $MoO_3$ (%) | nd |
| BaO (%) | 0,45 |
| PF (%) | 33,7 |

0,01% - limite de quantificação FRX
nd - não detectado

Executado por: Dra. Gislayne Kelmer - CRQ 04165656-4ªR (19/12/2019 11:52 BRT)
Revisado por: Saulo Colenci - CRQ 04262337-4ªR (19/12/2019 12:01 BRT)

Profa. Dra. Carina Ulsen
Coordenadora do LCT - Poli/USP

NOTA: Os resultados expostos acima referem-se apenas à(s) amostra(s) enviada(s) ao LCT; a representatividade da(s) mesma(s) é de inteira responsabilidade do cliente.

Verifique a autenticidade deste documento em www.lct.poli.usp.br utilizando o código **YJQE-XDYL-WVYU-AQRB**

Escola Politécnica da Universidade de São Paulo
Departamento de Engenharia de Minas e de Petróleo
Laboratório de Caracterização Tecnológica
Av. Prof. Mello Moraes, 2373 CEP 05508-030 São Paulo - SP www.lct.poli.usp.br
Tel: 11 3091-5151 e-mail: lct@lct.poli.usp.br

# RESULTADOS DE ANÁLISE QUÍMICA

**RELATÓRIO:** FRX 839-19 2 **REQ:** 1400-19 **DATA:** 19/12/2019

**CLIENTE:** Luís Piló

**1. MÉTODO:** Os teores apresentados foram dosados em amostra prensada, na calibração STD-1 (Standardless), relativa a análise sem padrões dos elementos químicos compreendidos entre o flúor e o urânio, em espectrômetro de fluorescência de raios X, marca Malvern Panalytical, modelo Zetium. Os valores foram normalizados a 100%. A Perda ao Fogo (PF) foi realizada a 1.020°C por 2h.

**2. RESULTADOS:**

| N° LCT | 10058 |
|---|---|
| Amostra | S11B-94-C2 |
| $Na_2O$ (%) | nd |
| MgO (%) | nd |
| $Al_2O_3$ (%) | 25,7 |
| $SiO_2$ (%) | 0,25 |
| $P_2O_5$ (%) | 54,3 |
| $SO_3$ (%) | 0,17 |
| Cl (%) | nd |
| $K_2O$ (%) | 5,18 |
| CaO (%) | 0,03 |
| $TiO_2$ (%) | nd |
| $Cr_2O_3$ (%) | nd |
| MnO (%) | 0,01 |
| $Fe_2O_3$ (%) | 2,27 |
| NiO (%) | nd |
| CuO (%) | 0,01 |
| ZnO (%) | <0,01 |
| $SeO_2$ (%) | nd |
| Br (%) | nd |
| $Rb_2O$ (%) | <0,01 |
| SrO (%) | nd |
| $ZrO_2$ (%) | nd |
| $MoO_3$ (%) | nd |
| BaO (%) | nd |
| PF (%) | 12,0 |

0,01% - limite de quantificação FRX
nd - não detectado

Executado por: Dra. Gislayne Kelmer - CRQ 04165656-4ªR (19/12/2019 11:52 BRT)
Revisado por: Saulo Colenci - CRQ 04262337-4ªR (19/12/2019 12:01 BRT)

Profa. Dra. Carina Ulsen
Coordenadora do LCT - Poli/USP

NOTA: Os resultados expostos acima referem-se apenas à(s) amostra(s) enviada(s) ao LCT; a representatividade da(s) mesma(s) é de inteira responsabilidade do cliente.

Verifique a autenticidade deste documento em www.lct.poli.usp.br utilizando o código **MJQG-KEYL-DDYU-CARB**

Escola Politécnica da Universidade de São Paulo
Departamento de Engenharia de Minas e de Petróleo
Laboratório de Caracterização Tecnológica
Av. Prof. Mello Moraes, 2373 CEP 05508-030 São Paulo - SP www.lct.poli.usp.br
Tel: 11 3091-5151 e-mail: lct@lct.poli.usp.br

# RESULTADOS DE ANÁLISE QUÍMICA

**RELATÓRIO:** FRX 839-19 3 **REQ:** 1400-19 **DATA:** 19/12/2019

**CLIENTE:** Luís Piló

**1. MÉTODO:** Os teores apresentados foram dosados em amostra prensada, na calibração STD-1 (Standardless), relativa a análise sem padrões dos elementos químicos compreendidos entre o flúor e o urânio, em espectrômetro de fluorescência de raios X, marca Malvern Panalytical, modelo Zetium. Os valores foram normalizados a 100%. A Perda ao Fogo (PF) foi realizada a 1.020°C por 2h.

**2. RESULTADOS:**

| N° LCT | 10059 |
|---|---|
| Amostra | S11B-94-C3 |
| $Na_2O$ (%) | nd |
| MgO (%) | nd |
| $Al_2O_3$ (%) | 33,0 |
| $SiO_2$ (%) | 0,08 |
| $P_2O_5$ (%) | 60,0 |
| $SO_3$ (%) | 0,10 |
| Cl (%) | nd |
| $K_2O$ (%) | 0,08 |
| CaO (%) | 0,03 |
| $TiO_2$ (%) | nd |
| $Cr_2O_3$ (%) | nd |
| MnO (%) | nd |
| $Fe_2O_3$ (%) | 2,59 |
| NiO (%) | nd |
| CuO (%) | 0,04 |
| ZnO (%) | 0,01 |
| $SeO_2$ (%) | nd |
| Br (%) | nd |
| $Rb_2O$ (%) | nd |
| SrO (%) | nd |
| $ZrO_2$ (%) | nd |
| $MoO_3$ (%) | nd |
| BaO (%) | 0,03 |
| PF (%) | 4,04 |

0,01% - limite de quantificação FRX
nd - não detectado

Executado por: Dra. Gislayne Kelmer - CRQ 04165656-4ªR (19/12/2019 11:52 BRT)
Revisado por: Saulo Colenci - CRQ 04262337-4ªR (19/12/2019 12:01 BRT)

Profa. Dra. Carina Ulsen
Coordenadora do LCT - Poli/USP

NOTA: Os resultados expostos acima referem-se apenas à(s) amostra(s) enviada(s) ao LCT; a representatividade da(s) mesma(s) é de inteira responsabilidade do cliente.

Verifique a autenticidade deste documento em www.lct.poli.usp.br utilizando o código **TJQI-YEYL-FKYU-SERB**

Escola Politécnica da Universidade de São Paulo
Departamento de Engenharia de Minas e de Petróleo
Laboratório de Caracterização Tecnológica
Av. Prof. Mello Moraes, 2373 CEP 05508-030 São Paulo - SP www.lct.poli.usp.br
Tel: 11 3091-5151 e-mail: lct@lct.poli.usp.br

# RESULTADOS DE ANÁLISE QUÍMICA

**RELATÓRIO:** FRX 839-19 4 **REQ:** 1400-19 **DATA:** 19/12/2019

**CLIENTE:** Luís Piló

**1. MÉTODO:** Os teores apresentados foram dosados em amostra prensada, na calibração STD-1 (Standardless), relativa a análise sem padrões dos elementos químicos compreendidos entre o flúor e o urânio, em espectrômetro de fluorescência de raios X, marca Malvern Panalytical, modelo Zetium. Os valores foram normalizados a 100%. A Perda ao Fogo (PF) foi realizada a 1.020°C por 2h.

**2. RESULTADOS:**

| N° LCT | 10060 |
|---|---|
| Amostra | S11B-94-C5 |
| $Na_2O$ (%) | 0,04 |
| MgO (%) | 0,16 |
| $Al_2O_3$ (%) | 2,38 |
| $SiO_2$ (%) | 0,67 |
| $P_2O_5$ (%) | 18,7 |
| $SO_3$ (%) | 0,70 |
| Cl (%) | 0,08 |
| $K_2O$ (%) | 4,57 |
| CaO (%) | 0,15 |
| $TiO_2$ (%) | 0,41 |
| $Cr_2O_3$ (%) | 0,01 |
| MnO (%) | 0,14 |
| $Fe_2O_3$ (%) | 23,5 |
| NiO (%) | nd |
| CuO (%) | 1,62 |
| ZnO (%) | 0,15 |
| $SeO_2$ (%) | nd |
| Br (%) | 0,10 |
| $Rb_2O$ (%) | 0,03 |
| SrO (%) | nd |
| $ZrO_2$ (%) | 0,01 |
| $MoO_3$ (%) | <0,01 |
| BaO (%) | 0,13 |
| PF (%) | 46,4 |

0,01% - limite de quantificação FRX
nd - não detectado

Executado por: Dra. Gislayne Kelmer - CRQ 04165656-4ªR (19/12/2019 11:52 BRT)
Revisado por: Saulo Colenci - CRQ 04262337-4ªR (19/12/2019 12:01 BRT)

Profa. Dra. Carina Ulsen
Coordenadora do LCT - Poli/USP

NOTA: Os resultados expostos acima referem-se apenas à(s) amostra(s) enviada(s) ao LCT; a representatividade da(s) mesma(s) é de inteira responsabilidade do cliente.

Verifique a autenticidade deste documento em www.lct.poli.usp.br utilizando o código **XJQK-SEYL-NRYU-MXRB**

Escola Politécnica da Universidade de São Paulo
Departamento de Engenharia de Minas e de Petróleo
Laboratório de Caracterização Tecnológica
Av. Prof. Mello Moraes, 2373 CEP 05508-030 São Paulo - SP www.lct.poli.usp.br
Tel: 11 3091-5151 e-mail: lct@lct.poli.usp.br

# RESULTADOS DE ANÁLISE QUÍMICA

**RELATÓRIO:** FRX 839-19 5 **REQ:** 1400-19 **DATA:** 19/12/2019

**CLIENTE:** Luís Piló

**1. MÉTODO:** Os teores apresentados foram dosados em amostra prensada, na calibração STD-1 (Standardless), relativa a análise sem padrões dos elementos químicos compreendidos entre o flúor e o urânio, em espectrômetro de fluorescência de raios X, marca Malvern Panalytical, modelo Zetium. Os valores foram normalizados a 100%. A Perda ao Fogo (PF) foi realizada a 1.020°C por 2h.

**2. RESULTADOS:**

| N° LCT | 10061 |
|---|---|
| Amostra | S11A-36-TVG |
| $Na_2O$ (%) | 0,02 |
| MgO (%) | 0,04 |
| $Al_2O_3$ (%) | 0,20 |
| $SiO_2$ (%) | 0,27 |
| $P_2O_5$ (%) | 0,56 |
| $SO_3$ (%) | 1,08 |
| Cl (%) | 0,05 |
| $K_2O$ (%) | 0,11 |
| CaO (%) | 0,13 |
| $TiO_2$ (%) | 0,04 |
| $Cr_2O_3$ (%) | nd |
| MnO (%) | <0,01 |
| $Fe_2O_3$ (%) | 2,25 |
| NiO (%) | nd |
| CuO (%) | 0,03 |
| ZnO (%) | 0,02 |
| $SeO_2$ (%) | nd |
| Br (%) | <0,01 |
| $Rb_2O$ (%) | nd |
| SrO (%) | <0,01 |
| $ZrO_2$ (%) | nd |
| $MoO_3$ (%) | nd |
| BaO (%) | nd |
| PF (%) | 95,2 |

0,01% - limite de quantificação FRX
nd - não detectado

Executado por: Dra. Gislayne Kelmer - CRQ 04165656-4ªR (19/12/2019 11:52 BRT)
Revisado por: Saulo Colenci - CRQ 04262337-4ªR (19/12/2019 12:01 BRT)

Profa. Dra. Carina Ulsen
Coordenadora do LCT - Poli/USP

NOTA: Os resultados expostos acima referem-se apenas à(s) amostra(s) enviada(s) ao LCT; a representatividade da(s) mesma(s) é de inteira responsabilidade do cliente.

Verifique a autenticidade deste documento em www.lct.poli.usp.br utilizando o código **YJQM-FEYL-OZYU-IERB**

Escola Politécnica da Universidade de São Paulo
Departamento de Engenharia de Minas e de Petróleo
Laboratório de Caracterização Tecnológica
Av. Prof. Mello Moraes, 2373 CEP 05508-030 São Paulo - SP www.lct.poli.usp.br
Tel: 11 3091-5151 e-mail: lct@lct.poli.usp.br

# RESULTADOS DE ANÁLISE QUÍMICA

**RELATÓRIO:** FRX 839-19 6 **REQ:** 1400-19 **DATA:** 19/12/2019

**CLIENTE:** Luís Piló

**1. MÉTODO:** Os teores apresentados foram dosados em amostra prensada, na calibração STD-1 (Standardless), relativa a análise sem padrões dos elementos químicos compreendidos entre o flúor e o urânio, em espectrômetro de fluorescência de raios X, marca Malvern Panalytical, modelo Zetium. Os valores foram normalizados a 100%. A Perda ao Fogo (PF) foi realizada a 1.020°C por 2h.

**2. RESULTADOS:**

| N° LCT | 10062 |
|---|---|
| Amostra | S11A-36-MVG |
| $Na_2O$ (%) | nd |
| MgO (%) | 0,02 |
| $Al_2O_3$ (%) | 0,38 |
| $SiO_2$ (%) | 1,03 |
| $P_2O_5$ (%) | 1,72 |
| $SO_3$ (%) | 1,14 |
| Cl (%) | 0,03 |
| $K_2O$ (%) | 0,11 |
| CaO (%) | 0,31 |
| $TiO_2$ (%) | 0,19 |
| $Cr_2O_3$ (%) | nd |
| MnO (%) | 0,01 |
| $Fe_2O_3$ (%) | 23,0 |
| NiO (%) | nd |
| CuO (%) | 0,04 |
| ZnO (%) | <0,01 |
| $SeO_2$ (%) | nd |
| Br (%) | <0,01 |
| $Rb_2O$ (%) | nd |
| SrO (%) | nd |
| $ZrO_2$ (%) | <0,01 |
| $MoO_3$ (%) | nd |
| BaO (%) | nd |
| PF (%) | 72,0 |

0,01% - limite de quantificação FRX
nd - não detectado

Executado por: Dra. Gislayne Kelmer - CRQ 04165656-4ªR (19/12/2019 11:52 BRT)
Revisado por: Saulo Colenci - CRQ 04262337-4ªR (19/12/2019 12:01 BRT)

Profa. Dra. Carina Ulsen
Coordenadora do LCT - Poli/USP

NOTA: Os resultados expostos acima referem-se apenas à(s) amostra(s) enviada(s) ao LCT; a representatividade da(s) mesma(s) é de inteira responsabilidade do cliente.

Verifique a autenticidade deste documento em www.lct.poli.usp.br utilizando o código **FJQO-HFYL-FGYU-WJRB**

Escola Politécnica da Universidade de São Paulo
Departamento de Engenharia de Minas e de Petróleo
Laboratório de Caracterização Tecnológica
Av. Prof. Mello Moraes, 2373 CEP 05508-030 São Paulo - SP www.lct.poli.usp.br
Tel: 11 3091-5151 e-mail: lct@lct.poli.usp.br

# RESULTADOS DE ANÁLISE QUÍMICA

**RELATÓRIO:** FRX 839-19 7 **REQ:** 1400-19 **DATA:** 19/12/2019

**CLIENTE:** Luís Piló

**1. MÉTODO:** Os teores apresentados foram dosados em amostra prensada, na calibração STD-1 (Standardless), relativa a análise sem padrões dos elementos químicos compreendidos entre o flúor e o urânio, em espectrômetro de fluorescência de raios X, marca Malvern Panalytical, modelo Zetium. Os valores foram normalizados a 100%. A Perda ao Fogo (PF) foi realizada a 1.020°C por 2h.

**2. RESULTADOS:**

| N° LCT | 10063 |
|---|---|
| Amostra | S11A-36-BVG |
| $Na_2O$ (%) | 0,05 |
| MgO (%) | 0,07 |
| $Al_2O_3$ (%) | 0,66 |
| $SiO_2$ (%) | 3,02 |
| $P_2O_5$ (%) | 8,00 |
| $SO_3$ (%) | 0,69 |
| Cl (%) | 0,04 |
| $K_2O$ (%) | 0,18 |
| CaO (%) | 0,36 |
| $TiO_2$ (%) | 0,52 |
| $Cr_2O_3$ (%) | 0,01 |
| MnO (%) | 0,02 |
| $Fe_2O_3$ (%) | 61,8 |
| NiO (%) | nd |
| CuO (%) | 0,04 |
| ZnO (%) | 0,02 |
| $SeO_2$ (%) | nd |
| Br (%) | <0,01 |
| $Rb_2O$ (%) | nd |
| SrO (%) | nd |
| $ZrO_2$ (%) | 0,02 |
| $MoO_3$ (%) | nd |
| BaO (%) | nd |
| PF (%) | 24,5 |

0,01% - limite de quantificação FRX
nd - não detectado

Executado por: Dra. Gislayne Kelmer - CRQ 04165656-4ªR (19/12/2019 11:52 BRT)
Revisado por: Saulo Colenci - CRQ 04262337-4ªR (19/12/2019 12:01 BRT)

Profa. Dra. Carina Ulsen
Coordenadora do LCT - Poli/USP

NOTA: Os resultados expostos acima referem-se apenas à(s) amostra(s) enviada(s) ao LCT; a representatividade da(s) mesma(s) é de inteira responsabilidade do cliente.

Verifique a autenticidade deste documento em www.lct.poli.usp.br utilizando o código **UJQQ-AFYL-BMYU-WYRB**

Escola Politécnica da Universidade de São Paulo
Departamento de Engenharia de Minas e de Petróleo
Laboratório de Caracterização Tecnológica
Av. Prof. Mello Moraes, 2373 CEP 05508-030 São Paulo - SP www.lct.poli.usp.br
Tel: 11 3091-5151 e-mail: lct@lct.poli.usp.br

# RESULTADOS DE ANÁLISE QUÍMICA

**RELATÓRIO:** FRX 839-19 8 **REQ:** 1400-19 **DATA:** 19/12/2019

**CLIENTE:** Luís Piló

**1. MÉTODO:** Os teores apresentados foram dosados em amostra prensada, na calibração STD-1 (Standardless), relativa a análise sem padrões dos elementos químicos compreendidos entre o flúor e o urânio, em espectrômetro de fluorescência de raios X, marca Malvern Panalytical, modelo Zetium. Os valores foram normalizados a 100%. A Perda ao Fogo (PF) foi realizada a 1.020°C por 2h.

**2. RESULTADOS:**

| N° LCT | 10064 |
|---|---|
| Amostra | S11A-36-MVF |
| $Na_2O$ (%) | nd |
| MgO (%) | 0,02 |
| $Al_2O_3$ (%) | 0,83 |
| $SiO_2$ (%) | 1,65 |
| $P_2O_5$ (%) | 1,25 |
| $SO_3$ (%) | 0,21 |
| Cl (%) | 0,01 |
| $K_2O$ (%) | 0,04 |
| CaO (%) | 0,03 |
| $TiO_2$ (%) | 0,47 |
| $Cr_2O_3$ (%) | 0,02 |
| MnO (%) | 0,01 |
| $Fe_2O_3$ (%) | 69,2 |
| NiO (%) | nd |
| CuO (%) | 0,01 |
| ZnO (%) | nd |
| $SeO_2$ (%) | nd |
| Br (%) | nd |
| $Rb_2O$ (%) | nd |
| SrO (%) | nd |
| $ZrO_2$ (%) | 0,02 |
| $MoO_3$ (%) | nd |
| BaO (%) | nd |
| PF (%) | 26,3 |

0,01% - limite de quantificação FRX
nd - não detectado

Executado por: Dra. Gislayne Kelmer - CRQ 04165656-4ªR (19/12/2019 11:52 BRT)
Revisado por: Saulo Colenci - CRQ 04262337-4ªR (19/12/2019 12:01 BRT)

Profa. Dra. Carina Ulsen
Coordenadora do LCT - Poli/USP

NOTA: Os resultados expostos acima referem-se apenas à(s) amostra(s) enviada(s) ao LCT; a representatividade da(s) mesma(s) é de inteira responsabilidade do cliente.

Verifique a autenticidade deste documento em www.lct.poli.usp.br utilizando o código **FJQS-DFYL-FTYU-IPRB**

Escola Politécnica da Universidade de São Paulo
Departamento de Engenharia de Minas e de Petróleo
Laboratório de Caracterização Tecnológica
Av. Prof. Mello Moraes, 2373 CEP 05508-030 São Paulo - SP www.lct.poli.usp.br
Tel: 11 3091-5151 e-mail: lct@lct.poli.usp.br

# RESULTADOS DE ANÁLISE QUÍMICA

**RELATÓRIO:** FRX 245-19 **REQ:** 0301-19 **DATA:** 16/04/2019

**CLIENTE:** Luís Piló

**1. MÉTODO:** Os teores apresentados foram dosados em amostra prensada, na calibração STD-1 (Standardless), relativa a análise sem padrões dos elementos químicos compreendidos entre o flúor e o urânio, em espectrômetro de fluorescência de raios X, marca Malvern Panalytical, modelo Zetium. A Perda ao Fogo (PF) foi realizada a 1.020°C por 2h.

**2. RESULTADOS:**

| N° LCT | 2097 | 2100 | 2101 | 2103 |
|---|---|---|---|---|
| Amostra | S11D-83-SUP | N5S-63-SUP | N5S-63-60 | S11A-36-VERT |
| $Na_2O$ (%) | 0,12 | 0,04 | 0,12 | 0,19 |
| MgO (%) | 0,19 | 0,09 | 1,36 | 0,68 |
| $Al_2O_3$ (%) | 0,03 | 0,02 | 0,16 | 1,12 |
| $SiO_2$ (%) | 0,07 | 0,03 | 0,56 | 2,33 |
| $P_2O_5$ (%) | 9,68 | 10,6 | 22,9 | 4,79 |
| $SO_3$ (%) | 1,58 | 1,19 | 2,73 | 7,39 |
| Cl (%) | 0,29 | 0,10 | 0,28 | 0,37 |
| $K_2O$ (%) | 0,57 | 0,54 | 1,43 | 1,21 |
| CaO (%) | 0,20 | 0,42 | 1,35 | 1,31 |
| $TiO_2$ (%) | <0,01 | <0,01 | 0,02 | 0,63 |
| $Cr_2O_3$ (%) | nd | nd | nd | 0,04 |
| MnO (%) | <0,01 | <0,01 | 0,13 | 0,20 |
| $Fe_2O_3$ (%) | 1,15 | 1,63 | 4,70 | 20,6 |
| NiO (%) | <0,01 | <0,01 | <0,01 | <0,01 |
| CuO (%) | <0,01 | <0,01 | 0,06 | 0,12 |
| ZnO (%) | 0,13 | 0,12 | 0,51 | 0,06 |
| Br (%) | <0,01 | <0,01 | 0,04 | 0,02 |
| $Rb_2O$ (%) | <0,01 | <0,01 | <0,01 | <0,01 |
| SrO (%) | <0,01 | <0,01 | <0,01 | nd |
| $ZrO_2$ (%) | nd | nd | <0,01 | 0,01 |
| PF (%) | 86,0 | 85,2 | 63,7 | 59,0 |

0,01% - limite de quantificação FRX
nd - não detectado
obs 1: Teores de Fe, P e Zn dosados por ICP OES (relatório ICP 053-19) e convertidos estequiometricamente para óxidos. Demais teores apresentados (à exceção de PF) recalculados para fechamento global em 100%

Profa. Dra. Carina Ulsen
Coordenadora do LCT

Dra. Maria Manuela Tassinari
Pesquisadora Sênior

Saulo Colenci
Pesquisador
CRQ 04262337-4ªR

NOTA: Os resultados expostos acima referem-se apenas à(s) alíquota(s) enviada(s) ao LCT; a representatividade da(s) mesma(s) é de inteira responsabilidade do cliente.

Verifique a autenticidade deste documento em www.lct.poli.usp.br utilizando o código **NKQA-HSJA-UFWA-GYAB**
