# Supplementary material for: Bats as ecosystem engineers in iron ore caves in the Carajás National Forest, Brazilian Amazonia
Source: PLoS One. 2023 May 11;18(5):e0267870. doi: 10.1371/journal.pone.0267870 (PMC10174506; doi:10.1371/journal.pone.0267870)
Supplement: S5 File — Reports issued by the Laboratório de Caracterização Tecnológica, Departamento de Engenharia de Minas e de Petróleo at the University of São Paulo´s Escola Politécnica, indicating Fe, Ni, P, Rb and Zi concentrations (mg/kg) in guano, soil and speleothems samples using the optical emission spectrometer. (PDF) [file pone.0267870.s009.pdf]

Escola Politécnica da Universidade de São Paulo
Departamento de Engenharia de Minas e de Petróleo
Laboratório de Caracterização Tecnológica
Av. Prof. Mello Moraes, 2373 CEP 05508-030 São Paulo - SP www.lct.poli.usp.br
Tel: 11 3091-5151 e-mail: lct@lct.poli.usp.br

RESULTADOS DE ANÁLISE QUÍMICA

RELATÓRIO: ICP 053-19 rev1 **REQ:** 060-19 **DATA:** 22/04/2019

CLIENTE: Luís Piló

1. MÉTODO: Os teores apresentados foram determinados por análise quantitativa em amostras preparadas por digestão multiácida e dosados em espectrômetro de emissão óptica (ICP OES), marca Horiba, modelo Ultima Expert.

2. RESULTADOS:

	Elemento	Fe	Ni	P	Rb	Zn
	Unidade	(mg/kg)	(mg/kg)	(mg/kg)	(mg/kg)	(mg/kg)
N° LCT	Amostra LQ	1	3	3	5	1
2097	S11D-83-SUP	8042	<LQ	42204	<LQ	1041
2098	S11A-36-BG	16713	3	48716	<LQ	2941
2099	N3-23-BG	46608	13	50615	<LQ	5186
2100	N5S-63-SUP	11353	<LQ	46206	<LQ	936
2101	N5S-63-60	32941	6	99666	<LQ	4132
2102	S11C-41-SUP	10072	4	32346	<LQ	5314
2103	S11A-36-VERT	144186	4	20947	<LQ	456

LQ - Limite de quantificação
Solicitação do cliente para que os resultados fossem expressos em mg/kg

Profa. Dra. Carina Ulsen
Coordenadora do LCT

Dra. Maria Manuela Tassinari
Pesquisadora Sênior

Dra. Gislayne Kelmer
Pesquisadora

NOTA: Os resultados expostos acima referem-se apenas à(s) alíquota(s) enviada(s) ao LCT; a representatividade da(s) mesma(s) é de inteira responsabilidade do cliente.

Verifique a autenticidade deste documento em www.lct.poli.usp.br utilizando o código **KDQA-THKA-DKFA-KFBB**

Escola Politécnica da Universidade de São Paulo
Departamento de Engenharia de Minas e de Petróleo
Laboratório de Caracterização Tecnológica
Av. Prof. Mello Moraes, 2373 CEP 05508-030 São Paulo - SP www.lct.poli.usp.br
Tel: 11 3091-5151 e-mail: lct@lct.poli.usp.br

RESULTADOS DE ANÁLISE QUÍMICA

RELATÓRIO: ICP 146-19 **REQ:** 0238-19 **DATA:** 18/09/2019

CLIENTE: Luís Piló

1. MÉTODO: Os teores apresentados foram determinados por análise quantitativa em amostras preparadas por digestão multiácida e dosados em espectrômetro de emissão óptica (ICP OES), marca Horiba, modelo Ultima Expert.

2. RESULTADOS:

	Elemento	Cu	Fe	Ni	P	Rb	Zn
	Unidade	(mg/kg)	(mg/kg)	(mg/kg)	(mg/kg)	(mg/kg)	(mg/kg)
N° LCT	Amostra LQ	1	1	3	3	5	1
7696	N4WS-15 -TITE	7	655633	4	84463	<LQ	74
7697	N4WS-72 -TITE	3	390657	<LQ	177410	<LQ	58
7698	N4WS-72A -TITE	13	386628	3	179102	<LQ	96
7699	N4WS-67- TITE	7	380527	<LQ	181781	<LQ	66
7700	N4WS-67A -TITE	5	388115	<LQ	180366	50	59
7701	M2-99 - TITE	53	387981	<LQ	178425	<LQ	177
7702	M2-99A - TITE	78	369184	<LQ	180025	33	426
7703	S11B-94 - MITE	144	354923	<LQ	173807	<LQ	336

LQ - Limite de quantificação

Executado por: Dra. Gislayne Kelmer - CRQ 04165656-4ªR (18/09/2019 18:18 BRT)
Revisado por: Dra. Maria Manuela Tassinari (19/09/2019 17:08 BRT)

Profa. Dra. Carina Ulsen
Coordenadora do LCT - Poli/USP

NOTA: Os resultados expostos acima referem-se apenas à(s) amostra(s) enviada(s) ao LCT; a representatividade da(s) mesma(s) é de inteira responsabilidade do cliente.

Verifique a autenticidade deste documento em www.lct.poli.usp.br utilizando o código **SWQH-TFTQ-ORKQ-ADGB**

Escola Politécnica da Universidade de São Paulo
Departamento de Engenharia de Minas e de Petróleo
Laboratório de Caracterização Tecnológica
Av. Prof. Mello Moraes, 2373 CEP 05508-030 São Paulo - SP www.lct.poli.usp.br
Tel: 11 3091-5151 e-mail: lct@lct.poli.usp.br

RESULTADOS DE ANÁLISE QUÍMICA

RELATÓRIO: ICP 179-19 1 **REQ:** 0321-19 **DATA:** 19/12/2019

CLIENTE: Luís Piló

1. MÉTODO: Os teores apresentados foram determinados por análise quantitativa em amostra preparada por digestão multiácida e dosados em espectrômetro de emissão óptica (ICP OES), marca Horiba, modelo Ultima Expert.

2. RESULTADOS:

	Elemento	Cu	Fe	Ni	P	Rb	Zn
	Unidade	(%)	(%)	(mg/kg)	(%)	(mg/kg)	(%)
N° LCT	Amostra LQ	0,0001	0,0001	3	0,0003	5	0,0001
10043	N3-23-P1-C1	0,027	4,51	5	5,16	63	0,132

LQ - Limite de quantificação

Executado por: Dra. Gislayne Kelmer - CRQ 04165656-4ªR (19/12/2019 10:50 BRT)
Revisado por: Saulo Colenci - CRQ 04262337-4ªR (19/12/2019 11:29 BRT)

Profa. Dra. Carina Ulsen
Coordenadora do LCT - Poli/USP

NOTA: Os resultados expostos acima referem-se apenas à(s) amostra(s) enviada(s) ao LCT; a representatividade da(s) mesma(s) é de inteira responsabilidade do cliente.

Verifique a autenticidade deste documento em www.lct.poli.usp.br utilizando o código **CKQR-GZYH-XVYU-KWQB**

Escola Politécnica da Universidade de São Paulo
Departamento de Engenharia de Minas e de Petróleo
Laboratório de Caracterização Tecnológica
Av. Prof. Mello Moraes, 2373 CEP 05508-030 São Paulo - SP www.lct.poli.usp.br
Tel: 11 3091-5151 e-mail: lct@lct.poli.usp.br

RESULTADOS DE ANÁLISE QUÍMICA

RELATÓRIO: ICP 179-19 2 **REQ:** 0321-19 **DATA:** 19/12/2019

CLIENTE: Luís Piló

1. MÉTODO: Os teores apresentados foram determinados por análise quantitativa em amostra preparada por digestão multiácida e dosados em espectrômetro de emissão óptica (ICP OES), marca Horiba, modelo Ultima Expert.

2. RESULTADOS:

	Elemento	Cu	Fe	Ni	P	Rb	Zn
	Unidade	(%)	(%)	(mg/kg)	(%)	(mg/kg)	(%)
N° LCT	Amostra LQ	0,0001	0,0001	3	0,0003	5	0,0001
10044	N3-23-P1-C2	0,026	3,00	9	4,67	57	0,120

LQ - Limite de quantificação

Executado por: Dra. Gislayne Kelmer - CRQ 04165656-4ªR (19/12/2019 10:50 BRT)
Revisado por: Saulo Colenci - CRQ 04262337-4ªR (19/12/2019 11:29 BRT)

Profa. Dra. Carina Ulsen
Coordenadora do LCT - Poli/USP

NOTA: Os resultados expostos acima referem-se apenas à(s) amostra(s) enviada(s) ao LCT; a representatividade da(s) mesma(s) é de inteira responsabilidade do cliente.

Verifique a autenticidade deste documento em www.lct.poli.usp.br utilizando o código **ILQH-SAYJ-SJYU-SNQB**

Escola Politécnica da Universidade de São Paulo
Departamento de Engenharia de Minas e de Petróleo
Laboratório de Caracterização Tecnológica
Av. Prof. Mello Moraes, 2373 CEP 05508-030 São Paulo - SP www.lct.poli.usp.br
Tel: 11 3091-5151 e-mail: lct@lct.poli.usp.br

RESULTADOS DE ANÁLISE QUÍMICA

RELATÓRIO: ICP 179-19 3 **REQ:** 0321-19 **DATA:** 19/12/2019

CLIENTE: Luís Piló

1. MÉTODO: Os teores apresentados foram determinados por análise quantitativa em amostra preparada por digestão multiácida e dosados em espectrômetro de emissão óptica (ICP OES), marca Horiba, modelo Ultima Expert.

2. RESULTADOS:

		Elemento<br>Unidade<br>LQ	Cu<br>(%)<br>0,0001	Fe<br>(%)<br>0,0001	Ni<br>(mg/kg)<br>3	P<br>(%)<br>0,0003	Rb<br>(mg/kg)<br>5	Zn<br>(%)<br>0,0001
N° LCT	Amostra							
10045	N3-23-P1-C3		0,036	1,97	12	2,42	58	0,167

LQ - Limite de quantificação

Executado por: Dra. Gislayne Kelmer - CRQ 04165656-4ªR (19/12/2019 10:50 BRT)
Revisado por: Saulo Colenci - CRQ 04262337-4ªR (19/12/2019 11:29 BRT)

Profa. Dra. Carina Ulsen
Coordenadora do LCT - Poli/USP

NOTA: Os resultados expostos acima referem-se apenas à(s) amostra(s) enviada(s) ao LCT; a representatividade da(s) mesma(s) é de inteira responsabilidade do cliente.

Verifique a autenticidade deste documento em www.lct.poli.usp.br utilizando o código **DLQI-YAYJ-CCYU-SKQB**

Escola Politécnica da Universidade de São Paulo
Departamento de Engenharia de Minas e de Petróleo
Laboratório de Caracterização Tecnológica
Av. Prof. Mello Moraes, 2373 CEP 05508-030 São Paulo - SP www.lct.poli.usp.br
Tel: 11 3091-5151 e-mail: lct@lct.poli.usp.br

RESULTADOS DE ANÁLISE QUÍMICA

RELATÓRIO: ICP 179-19 4 **REQ:** 0321-19 **DATA:** 19/12/2019

CLIENTE: Luís Piló

1. MÉTODO: Os teores apresentados foram determinados por análise quantitativa em amostra preparada por digestão multiácida e dosados em espectrômetro de emissão óptica (ICP OES), marca Horiba, modelo Ultima Expert.

2. RESULTADOS:

	Elemento	Cu	Fe	Ni	P	Rb	Zn
	Unidade	(%)	(%)	(mg/kg)	(%)	(mg/kg)	(%)
N° LCT	Amostra LQ	0,0001	0,0001	3	0,0003	5	0,0001
10046	N3-23-P1-C4	0,044	3,97	11	7,74	61	0,231

LQ - Limite de quantificação

Executado por: Dra. Gislayne Kelmer - CRQ 04165656-4ªR (19/12/2019 10:50 BRT)
Revisado por: Saulo Colenci - CRQ 04262337-4ªR (19/12/2019 11:29 BRT)

Profa. Dra. Carina Ulsen
Coordenadora do LCT - Poli/USP

NOTA: Os resultados expostos acima referem-se apenas à(s) amostra(s) enviada(s) ao LCT; a representatividade da(s) mesma(s) é de inteira responsabilidade do cliente.

Verifique a autenticidade deste documento em www.lct.poli.usp.br utilizando o código **ILQJ-EAYJ-QPYU-WTQB**

Escola Politécnica da Universidade de São Paulo
Departamento de Engenharia de Minas e de Petróleo
Laboratório de Caracterização Tecnológica
Av. Prof. Mello Moraes, 2373 CEP 05508-030 São Paulo - SP www.lct.poli.usp.br
Tel: 11 3091-5151 e-mail: lct@lct.poli.usp.br

RESULTADOS DE ANÁLISE QUÍMICA

RELATÓRIO: ICP 179-19 5 **REQ:** 0321-19 **DATA:** 19/12/2019

CLIENTE: Luís Piló

1. MÉTODO: Os teores apresentados foram determinados por análise quantitativa em amostra preparada por digestão multiácida e dosados em espectrômetro de emissão óptica (ICP OES), marca Horiba, modelo Ultima Expert.

2. RESULTADOS:

		Elemento	Cu	Fe	Ni	P	Rb	Zn
		Unidade	(%)	(%)	(mg/kg)	(%)	(mg/kg)	(%)
N° LCT	Amostra	LQ	0,0001	0,0001	3	0,0003	5	0,0001
10047	N3-23-P1-C5		0,063	2,40	20	1,93	113	0,341

LQ - Limite de quantificação

Executado por: Dra. Gislayne Kelmer - CRQ 04165656-4ªR (19/12/2019 10:50 BRT)
Revisado por: Saulo Colenci - CRQ 04262337-4ªR (19/12/2019 11:29 BRT)

Profa. Dra. Carina Ulsen
Coordenadora do LCT - Poli/USP

NOTA: Os resultados expostos acima referem-se apenas à(s) amostra(s) enviada(s) ao LCT; a representatividade da(s) mesma(s) é de inteira responsabilidade do cliente.

Verifique a autenticidade deste documento em www.lct.poli.usp.br utilizando o código **ILQK-VAYJ-AWYU-SPQB**

Escola Politécnica da Universidade de São Paulo
Departamento de Engenharia de Minas e de Petróleo
Laboratório de Caracterização Tecnológica
Av. Prof. Mello Moraes, 2373 CEP 05508-030 São Paulo - SP www.lct.poli.usp.br
Tel: 11 3091-5151 e-mail: lct@lct.poli.usp.br

RESULTADOS DE ANÁLISE QUÍMICA

RELATÓRIO: ICP 179-19 6 **REQ:** 0321-19 **DATA:** 19/12/2019

CLIENTE: Luís Piló

1. MÉTODO: Os teores apresentados foram determinados por análise quantitativa em amostra preparada por digestão multiácida e dosados em espectrômetro de emissão óptica (ICP OES), marca Horiba, modelo Ultima Expert.

2. RESULTADOS:

	Elemento	Cu	Fe	Ni	P	Rb	Zn
	Unidade	(%)	(%)	(mg/kg)	(%)	(mg/kg)	(%)
N° LCT	Amostra LQ	0,0001	0,0001	3	0,0003	5	0,0001
10048	N3-23-P1-C6	0,056	3,12	17	6,06	10	0,251

LQ - Limite de quantificação

Executado por: Dra. Gislayne Kelmer - CRQ 04165656-4ªR (19/12/2019 10:50 BRT)
Revisado por: Saulo Colenci - CRQ 04262337-4ªR (19/12/2019 11:29 BRT)

Profa. Dra. Carina Ulsen
Coordenadora do LCT - Poli/USP

NOTA: Os resultados expostos acima referem-se apenas à(s) amostra(s) enviada(s) ao LCT; a representatividade da(s) mesma(s) é de inteira responsabilidade do cliente.

Verifique a autenticidade deste documento em www.lct.poli.usp.br utilizando o código **KLQL-ZBYJ-EDYU-WTQB**

Escola Politécnica da Universidade de São Paulo
Departamento de Engenharia de Minas e de Petróleo
Laboratório de Caracterização Tecnológica
Av. Prof. Mello Moraes, 2373 CEP 05508-030 São Paulo - SP www.lct.poli.usp.br
Tel: 11 3091-5151 e-mail: lct@lct.poli.usp.br

RESULTADOS DE ANÁLISE QUÍMICA

RELATÓRIO: ICP 179-19 7 **REQ:** 0321-19 **DATA:** 19/12/2019

CLIENTE: Luís Piló

1. MÉTODO: Os teores apresentados foram determinados por análise quantitativa em amostra preparada por digestão multiácida e dosados em espectrômetro de emissão óptica (ICP OES), marca Horiba, modelo Ultima Expert.

2. RESULTADOS:

	Elemento	Cu	Fe	Ni	P	Rb	Zn
	Unidade	(%)	(%)	(mg/kg)	(%)	(mg/kg)	(%)
N° LCT	Amostra LQ	0,0001	0,0001	3	0,0003	5	0,0001
10049	N3-23-P1-C7	0,118	15,6	14	6,20	122	0,345

LQ - Limite de quantificação

Executado por: Dra. Gislayne Kelmer - CRQ 04165656-4ªR (19/12/2019 10:50 BRT)
Revisado por: Saulo Colenci - CRQ 04262337-4ªR (19/12/2019 11:29 BRT)

Profa. Dra. Carina Ulsen
Coordenadora do LCT - Poli/USP

NOTA: Os resultados expostos acima referem-se apenas à(s) amostra(s) enviada(s) ao LCT; a representatividade da(s) mesma(s) é de inteira responsabilidade do cliente.

Verifique a autenticidade deste documento em www.lct.poli.usp.br utilizando o código **NLQM-OBYJ-NKYU-QRQB**

Escola Politécnica da Universidade de São Paulo
Departamento de Engenharia de Minas e de Petróleo
Laboratório de Caracterização Tecnológica
Av. Prof. Mello Moraes, 2373 CEP 05508-030 São Paulo - SP www.lct.poli.usp.br
Tel: 11 3091-5151 e-mail: lct@lct.poli.usp.br

RESULTADOS DE ANÁLISE QUÍMICA

RELATÓRIO: ICP 179-19 8 **REQ:** 0321-19 **DATA:** 19/12/2019

CLIENTE: Luís Piló

1. MÉTODO: Os teores apresentados foram determinados por análise quantitativa em amostra preparada por digestão multiácida e dosados em espectrômetro de emissão óptica (ICP OES), marca Horiba, modelo Ultima Expert.

2. RESULTADOS:

		Elemento	Cu	Fe	Ni	P	Rb	Zn
		Unidade	(%)	(%)	(mg/kg)	(%)	(mg/kg)	(%)
N° LCT	Amostra	LQ	0,0001	0,0001	3	0,0003	5	0,0001
10050	N3-23-P2-C1		0,557	9,76	56	2,89	260	0,245

LQ - Limite de quantificação

Executado por: Dra. Gislayne Kelmer - CRQ 04165656-4ªR (19/12/2019 10:50 BRT)
Revisado por: Saulo Colenci - CRQ 04262337-4ªR (19/12/2019 11:29 BRT)

Profa. Dra. Carina Ulsen
Coordenadora do LCT - Poli/USP

NOTA: Os resultados expostos acima referem-se apenas à(s) amostra(s) enviada(s) ao LCT; a representatividade da(s) mesma(s) é de inteira responsabilidade do cliente.

Verifique a autenticidade deste documento em www.lct.poli.usp.br utilizando o código **KLQN-YBYJ-WRYU-YMQB**

Escola Politécnica da Universidade de São Paulo
Departamento de Engenharia de Minas e de Petróleo
Laboratório de Caracterização Tecnológica
Av. Prof. Mello Moraes, 2373 CEP 05508-030 São Paulo - SP www.lct.poli.usp.br
Tel: 11 3091-5151 e-mail: lct@lct.poli.usp.br

RESULTADOS DE ANÁLISE QUÍMICA

RELATÓRIO: ICP 179-19 9 **REQ:** 0321-19 **DATA:** 19/12/2019

CLIENTE: Luís Piló

1. MÉTODO: Os teores apresentados foram determinados por análise quantitativa em amostra preparada por digestão multiácida e dosados em espectrômetro de emissão óptica (ICP OES), marca Horiba, modelo Ultima Expert.

2. RESULTADOS:

	Elemento	Cu	Fe	Ni	P	Rb	Zn
	Unidade	(%)	(%)	(mg/kg)	(%)	(mg/kg)	(%)
N° LCT	Amostra LQ	0,0001	0,0001	3	0,0003	5	0,0001
10051	N3-23-P2-C2	0,592	13,8	41	4,18	41	0,284

LQ - Limite de quantificação

Executado por: Dra. Gislayne Kelmer - CRQ 04165656-4ªR (19/12/2019 10:50 BRT)
Revisado por: Saulo Colenci - CRQ 04262337-4ªR (19/12/2019 11:29 BRT)

Profa. Dra. Carina Ulsen
Coordenadora do LCT - Poli/USP

NOTA: Os resultados expostos acima referem-se apenas à(s) amostra(s) enviada(s) ao LCT; a representatividade da(s) mesma(s) é de inteira responsabilidade do cliente.

Verifique a autenticidade deste documento em www.lct.poli.usp.br utilizando o código **BLQO-SBYJ-CYYU-AMQB**

Escola Politécnica da Universidade de São Paulo
Departamento de Engenharia de Minas e de Petróleo
Laboratório de Caracterização Tecnológica
Av. Prof. Mello Moraes, 2373 CEP 05508-030 São Paulo - SP www.lct.poli.usp.br
Tel: 11 3091-5151 e-mail: lct@lct.poli.usp.br

RESULTADOS DE ANÁLISE QUÍMICA

RELATÓRIO: ICP 179-19 10 **REQ:** 0321-19 **DATA:** 19/12/2019

CLIENTE: Luís Piló

1. MÉTODO: Os teores apresentados foram determinados por análise quantitativa em amostra preparada por digestão multiácida e dosados em espectrômetro de emissão óptica (ICP OES), marca Horiba, modelo Ultima Expert.

2. RESULTADOS:

	Elemento	Cu	Fe	Ni	P	Rb	Zn
	Unidade	(%)	(%)	(mg/kg)	(%)	(mg/kg)	(%)
N° LCT	Amostra LQ	0,0001	0,0001	3	0,0003	5	0,0001
10052	N3-23-P2-C3	0,445	11,4	20	2,78	120	0,161

LQ - Limite de quantificação

Executado por: Dra. Gislayne Kelmer - CRQ 04165656-4ªR (19/12/2019 10:50 BRT)
Revisado por: Saulo Colenci - CRQ 04262337-4ªR (19/12/2019 11:29 BRT)

Profa. Dra. Carina Ulsen
Coordenadora do LCT - Poli/USP

NOTA: Os resultados expostos acima referem-se apenas à(s) amostra(s) enviada(s) ao LCT; a representatividade da(s) mesma(s) é de inteira responsabilidade do cliente.

Verifique a autenticidade deste documento em www.lct.poli.usp.br utilizando o código **SLQP-OCYJ-OFYU-AFQB**

Escola Politécnica da Universidade de São Paulo
Departamento de Engenharia de Minas e de Petróleo
Laboratório de Caracterização Tecnológica
Av. Prof. Mello Moraes, 2373 CEP 05508-030 São Paulo - SP www.lct.poli.usp.br
Tel: 11 3091-5151 e-mail: lct@lct.poli.usp.br

RESULTADOS DE ANÁLISE QUÍMICA

RELATÓRIO: ICP 179-19 11 **REQ:** 0321-19 **DATA:** 19/12/2019

CLIENTE: Luís Piló

1. MÉTODO: Os teores apresentados foram determinados por análise quantitativa em amostra preparada por digestão multiácida e dosados em espectrômetro de emissão óptica (ICP OES), marca Horiba, modelo Ultima Expert.

2. RESULTADOS:

		Elemento	Cu	Fe	Ni	P	Rb	Zn
		Unidade	(%)	(%)	(mg/kg)	(%)	(mg/kg)	(%)
N° LCT	Amostra	LQ	0,0001	0,0001	3	0,0003	5	0,0001
10053	M2-99-C1		0,100	0,861	5	2,37	<LQ	0,249

LQ - Limite de quantificação

Executado por: Dra. Gislayne Kelmer - CRQ 04165656-4ªR (19/12/2019 10:50 BRT)
Revisado por: Saulo Colenci - CRQ 04262337-4ªR (19/12/2019 11:29 BRT)

Profa. Dra. Carina Ulsen
Coordenadora do LCT - Poli/USP

NOTA: Os resultados expostos acima referem-se apenas à(s) amostra(s) enviada(s) ao LCT; a representatividade da(s) mesma(s) é de inteira responsabilidade do cliente.

Verifique a autenticidade deste documento em www.lct.poli.usp.br utilizando o código **GLQQ-YCYJ-ZLYU-YRQB**

Escola Politécnica da Universidade de São Paulo
Departamento de Engenharia de Minas e de Petróleo
Laboratório de Caracterização Tecnológica
Av. Prof. Mello Moraes, 2373 CEP 05508-030 São Paulo - SP www.lct.poli.usp.br
Tel: 11 3091-5151 e-mail: lct@lct.poli.usp.br

RESULTADOS DE ANÁLISE QUÍMICA

RELATÓRIO: ICP 179-19 12 **REQ:** 0321-19 **DATA:** 19/12/2019

CLIENTE: Luís Piló

1. MÉTODO: Os teores apresentados foram determinados por análise quantitativa em amostra preparada por digestão multiácida e dosados em espectrômetro de emissão óptica (ICP OES), marca Horiba, modelo Ultima Expert.

2. RESULTADOS:

		Elemento	Cu	Fe	Ni	P	Rb	Zn
		Unidade	(%)	(%)	(mg/kg)	(%)	(mg/kg)	(%)
N° LCT	Amostra	LQ	0,0001	0,0001	3	0,0003	5	0,0001
10054	M2-99-C2		0,301	3,38	13	5,19	12	0,851

LQ - Limite de quantificação

Executado por: Dra. Gislayne Kelmer - CRQ 04165656-4ªR (19/12/2019 10:50 BRT)
Revisado por: Saulo Colenci - CRQ 04262337-4ªR (19/12/2019 11:29 BRT)

Profa. Dra. Carina Ulsen
Coordenadora do LCT - Poli/USP

NOTA: Os resultados expostos acima referem-se apenas à(s) amostra(s) enviada(s) ao LCT; a representatividade da(s) mesma(s) é de inteira responsabilidade do cliente.

Verifique a autenticidade deste documento em www.lct.poli.usp.br utilizando o código **LLQR-GCYJ-VSYU-KPQB**

Escola Politécnica da Universidade de São Paulo
Departamento de Engenharia de Minas e de Petróleo
Laboratório de Caracterização Tecnológica
Av. Prof. Mello Moraes, 2373 CEP 05508-030 São Paulo - SP www.lct.poli.usp.br
Tel: 11 3091-5151 e-mail: lct@lct.poli.usp.br

RESULTADOS DE ANÁLISE QUÍMICA

RELATÓRIO: ICP 179-19 13 **REQ:** 0321-19 **DATA:** 19/12/2019

CLIENTE: Luís Piló

1. MÉTODO: Os teores apresentados foram determinados por análise quantitativa em amostra preparada por digestão multiácida e dosados em espectrômetro de emissão óptica (ICP OES), marca Horiba, modelo Ultima Expert.

2. RESULTADOS:

	Elemento	Cu	Fe	Ni	P	Rb	Zn
	Unidade	(%)	(%)	(mg/kg)	(%)	(mg/kg)	(%)
N° LCT	Amostra LQ	0,0001	0,0001	3	0,0003	5	0,0001
10055	M2-99-C3	0,395	3,61	21	2,69	55	0,909

LQ - Limite de quantificação

Executado por: Dra. Gislayne Kelmer - CRQ 04165656-4ªR (19/12/2019 10:50 BRT)
Revisado por: Saulo Colenci - CRQ 04262337-4ªR (19/12/2019 11:29 BRT)

Profa. Dra. Carina Ulsen
Coordenadora do LCT - Poli/USP

NOTA: Os resultados expostos acima referem-se apenas à(s) amostra(s) enviada(s) ao LCT; a representatividade da(s) mesma(s) é de inteira responsabilidade do cliente.

Verifique a autenticidade deste documento em www.lct.poli.usp.br utilizando o código **BLQS-TDYJ-EEYU-SKQB**

Escola Politécnica da Universidade de São Paulo
Departamento de Engenharia de Minas e de Petróleo
Laboratório de Caracterização Tecnológica
Av. Prof. Mello Moraes, 2373 CEP 05508-030 São Paulo - SP www.lct.poli.usp.br
Tel: 11 3091-5151 e-mail: lct@lct.poli.usp.br

RESULTADOS DE ANÁLISE QUÍMICA

RELATÓRIO: ICP 179-19 14 **REQ:** 0321-19 **DATA:** 19/12/2019

CLIENTE: Luís Piló

1. MÉTODO: Os teores apresentados foram determinados por análise quantitativa em amostra preparada por digestão multiácida e dosados em espectrômetro de emissão óptica (ICP OES), marca Horiba, modelo Ultima Expert.

2. RESULTADOS:

		Elemento	Cu	Fe	Ni	P	Rb	Zn
		Unidade	(%)	(%)	(mg/kg)	(%)	(mg/kg)	(%)
N° LCT	Amostra	LQ	0,0001	0,0001	3	0,0003	5	0,0001
10056	M2-99-C4		0,430	13,9	14	6,16	53	0,718

LQ - Limite de quantificação

Executado por: Dra. Gislayne Kelmer - CRQ 04165656-4ªR (19/12/2019 10:50 BRT)
Revisado por: Saulo Colenci - CRQ 04262337-4ªR (19/12/2019 11:29 BRT)

Profa. Dra. Carina Ulsen
Coordenadora do LCT - Poli/USP

NOTA: Os resultados expostos acima referem-se apenas à(s) amostra(s) enviada(s) ao LCT; a representatividade da(s) mesma(s) é de inteira responsabilidade do cliente.

Verifique a autenticidade deste documento em www.lct.poli.usp.br utilizando o código **MLQT-EDYJ-YKYU-IWQB**
