# Supplementary material for: Bats as ecosystem engineers in iron ore caves in the Carajás National Forest, Brazilian Amazonia
Source: PLoS One. 2023 May 11;18(5):e0267870. doi: 10.1371/journal.pone.0267870 (PMC10174506; doi:10.1371/journal.pone.0267870)
Supplement: S6 File — Reports issued by the Laboratório de Caracterização Tecnológica, Departamento de Engenharia de Minas e de Petróleo at the University of São Paulo´s Escola Politécnica, indicating Fe, Ni, P, Rb and Zi concentrations (mg/kg) in stalactites samples using the optical emission spectrometer. (PDF) [file pone.0267870.s010.pdf]

Escola Politécnica da Universidade de São Paulo
Departamento de Engenharia de Minas e de Petróleo
Laboratório de Caracterização Tecnológica
Av. Prof. Mello Moraes, 2373 CEP 05508-030 São Paulo - SP www.lct.poli.usp.br
Tel: 11 3091-5151 e-mail: lct@lct.poli.usp.br

RESULTADOS DE ANÁLISE QUÍMICA

RELATÓRIO: ICP 146-19 **REQ:** 0238-19 **DATA:** 18/09/2019

CLIENTE: Luís Piló

1. MÉTODO: Os teores apresentados foram determinados por análise quantitativa em amostras preparadas por digestão multiácida e dosados em espectrômetro de emissão óptica (ICP OES), marca Horiba, modelo Ultima Expert.

2. RESULTADOS:

	Elemento	Cu	Fe	Ni	P	Rb	Zn
	Unidade	(mg/kg)	(mg/kg)	(mg/kg)	(mg/kg)	(mg/kg)	(mg/kg)
N° LCT	Amostra LQ	1	1	3	3	5	1
7696	N4WS-15 -TITE	7	655633	4	84463	<LQ	74
7697	N4WS-72 -TITE	3	390657	<LQ	177410	<LQ	58
7698	N4WS-72A -TITE	13	386628	3	179102	<LQ	96
7699	N4WS-67- TITE	7	380527	<LQ	181781	<LQ	66
7700	N4WS-67A -TITE	5	388115	<LQ	180366	50	59
7701	M2-99 - TITE	53	387981	<LQ	178425	<LQ	177
7702	M2-99A - TITE	78	369184	<LQ	180025	33	426
7703	S11B-94 - MITE	144	354923	<LQ	173807	<LQ	336

LQ - Limite de quantificação

Executado por: Dra. Gislayne Kelmer - CRQ 04165656-4ªR (18/09/2019 18:18 BRT)
Revisado por: Dra. Maria Manuela Tassinari (19/09/2019 17:08 BRT)

Profa. Dra. Carina Ulsen
Coordenadora do LCT - Poli/USP

NOTA: Os resultados expostos acima referem-se apenas à(s) amostra(s) enviada(s) ao LCT; a representatividade da(s) mesma(s) é de inteira responsabilidade do cliente.

Verifique a autenticidade deste documento em www.lct.poli.usp.br utilizando o código **SWQH-TFTQ-ORKQ-ADGB**

Escola Politécnica da Universidade de São Paulo
Departamento de Engenharia de Minas e de Petróleo
Laboratório de Caracterização Tecnológica
Av. Prof. Mello Moraes, 2373 CEP 05508-030 São Paulo - SP www.lct.poli.usp.br
Tel: 11 3091-5151 e-mail: lct@lct.poli.usp.br

RESULTADOS DE ANÁLISE QUÍMICA

RELATÓRIO: ICP 045-19 rev1 **REQ:** 057-19 **DATA:** 22/04/2019

CLIENTE: Luís Piló

1. MÉTODO: Os teores apresentados foram determinados por análise quantitativa em amostras preparadas por digestão multiácida e dosados em espectrômetro de emissão óptica (ICP OES), marca Horiba, modelo Ultima Expert.

2. RESULTADOS:

	Elemento	Fe	Ni	P	Rb	Zn
	Unidade	(mg/kg)	(mg/kg)	(mg/kg)	(mg/kg)	(mg/kg)
N° LCT	Amostra LQ	1	3	3	5	1
2094	N3-23-TITE	381084	<LQ	178709	105	426
2095	N3-74-TITE	393139	<LQ	169786	<LQ	74
2096	S11A-36-TITE	393557	<LQ	169196	<LQ	54

LQ - Limite de quantificação
Solicitação do cliente para que os resultados fossem expressos em mg/kg

Profa. Dra. Carina Ulsen
Coordenadora do LCT

Dra. Maria Manuela Tassinari
Pesquisadora Sênior

Dra. Gislayne Kelmer
Pesquisadora

NOTA: Os resultados expostos acima referem-se apenas à(s) alíquota(s) enviada(s) ao LCT; a representatividade da(s) mesma(s) é de inteira responsabilidade do cliente.

Verifique a autenticidade deste documento em www.lct.poli.usp.br utilizando o código **OAQA-QQKA-KSFA-SBBB**

Escola Politécnica da Universidade de São Paulo
Departamento de Engenharia de Minas e de Petróleo
Laboratório de Caracterização Tecnológica
Av. Prof. Mello Moraes, 2373 CEP 05508-030 São Paulo - SP www.lct.poli.usp.br
Tel: 11 3091-5151 e-mail: lct@lct.poli.usp.br

RESULTADOS DE ANÁLISE QUÍMICA

RELATÓRIO: ICP 074-19 **REQ:** 091-19 **DATA:** 14/05/2019

CLIENTE: Luís Piló

1. MÉTODO: Os teores apresentados foram determinados por análise quantitativa em amostras preparadas por digestão multiácida e dosados em espectrômetro de emissão óptica (ICP OES), marca Horiba, modelo Ultima Expert.

2. RESULTADOS:

Nº LCT	Amostra	Elemento<br>Unidade<br>LQ	Cu<br>(mg/kg)<br>1	Na<br>(mg/kg)<br>5
2094	N3-23-TITE		489	62
2095	N3-74-TITE		51	28
2096	S11A-36-TITE		45	32

LQ - Limite de quantificação

Profa. Dra. Carina Ulsen
Coordenadora do LCT

Dra. Maria Manuela Tassinari
Pesquisadora Sênior

Dra. Gislayne Kelmer
Pesquisadora

NOTA: Os resultados expostos acima referem-se apenas à(s) alíquota(s) enviada(s) ao LCT; a representatividade da(s) mesma(s) é de inteira responsabilidade do cliente.

Verifique a autenticidade deste documento em www.lct.poli.usp.br utilizando o código **LQQA-YCLA-PMOA-OMCB**
