# Supplementary material for: Bats as ecosystem engineers in iron ore caves in the Carajás National Forest, Brazilian Amazonia
Source: PLoS One. 2023 May 11;18(5):e0267870. doi: 10.1371/journal.pone.0267870 (PMC10174506; doi:10.1371/journal.pone.0267870)
Supplement: Attachment — Submitted filename: PONE-D-22-11101 review - Copy.pdf [file pone.0267870.s012.pdf]

PONE-D-22-11101

Pilo et al: Bats as ecosystem engineers in iron ore caves in the Carajás National Forest, Brazilian Amazonia

Review

This might be considered to be ancillary, but I was very disturbed by the apparently wanton destructive sampling of so many speleothems. For example, line 227-230 notes the rare occurrence of stalactites and stalagmites … yet these researchers *took 18 of them*. The total number of speleothem samples they report (line 136) is 108. This is an unbelievable number of samples of a non-renewable resource.

The very basis of the research – the designation of "bat cave" versus "non-bat cave" is not rigorous at all … in fact does not seem to have been clearly defined at all. How many bats is required to fit the requirement? One bat? one thousand? We are simply told that nine bat caves were chosen (and it is only by looking at the Supplementary text that we discover that two of them had no guano – at least I think that is what Table S1 says – depends on the meaning of "expressive")

The research aim is not clearly laid out. Their aim is "to provide evidence of the role of bats as ecosystem engineers" … but they offer no concrete research questions. I soon understood that it is not the bats themselves that effect the engineering, but rather it is the guano. However, I had to surmise this from their immediate jump to "We analyzed guano …". They do not state, anywhere in the introduction, the very obvious step that they would compare the features of non-bat caves to bat caves (i.e., test the hypothesis that bat caves would show significant differences in process and geomorphology (erosional and depositional features) from non-bat caves. We eventually get to this at line 131-135. I would like the basic research strategy to be explained *before* the methods section.

Basis of research sampling is … strange ….

As I understand it, in order to prove that bats engineer their ecosystem, the authors need to prove that there are significant differences between caves with bats versus caves without bats. Normally, when comparing two populations to see if one shows a significant difference from the other, I would expect samples to be relatively well matched so that , as far as is possible, only one variable is different between the two. In this case, I would expect caves to be selectively matched for as many geographic/geologic variables as possible, with bat presence the only major difference. So, I would expect to see roughly similar sample sizes, and some explanation of the sampling strategy. However, the sampling is astonishingly lopsided: for the detailed studies these authors compared nine bat caves (of which two appear to have no guano, and of which at least two are designated as active and at least one as inactive – I have not found details for all of them) with 26 non-bat caves (very few details/results given for these caves) and for the cave size comparisons they compare the nine bat caves against *more than a thousand* non-bat caves. I see no effort to control for other (biological or non-biological) variables that might explain differences. Even something very simple as location in relation to scarp face will govern the size of these caves regardless of bat population (smaller further from scarp).

Were the nine the *only* caves in the region that housed bats? … and, as mentioned above, what constitutes a bat cave? also, do we know that all 1303 non-bat caves were actually devoid of bats? In this geological situation, the "caves" are simply the porosity voids that are big enough for humans to enter … so, I would be surprised if these colonial bats would choose to live in the tiniest ones.

The authors do lean rather a lot on the difference in size of the caves. However, I am not sure that comparing size is valid. With reference to comparing size of bat and non-bat caves, I am bothered by the possibility/probability that bat caves are bigger *simply because bats choose to colonize only the bigger caves* (a question with no clear consensus in bat biology research). These types of colonial bats require a high temperature and humidity to thrive, and small caves inherently have larger surface area (i.e., more energy-conducting rock surface) to volume ratios, so it requires more energy to maintain the high temperatures in smaller colonies and in smaller voids. I suggest that, in finding 1303 non-bat caves to compare with only 9 bat caves they are of course going to be sampling many tiny caves, and thus biasing the results towards smaller non-bat caves.

Manuscript structure is not always quite logical: e.g., In the section “Cave and speleothem characterization” the small-scale features are described and the component minerals listed, starting with all the depositional forms. There is no indication that bat and non-bat caves were being compared until line 236. Then they suddenly offer a comparison of the two types – but from the cave survey data rather than the features in the cave. My obvious question is: why offer all those lists of minerals in the speleothems but fail to report on the comparison between cave types.

Guano Sampling: Table S2 gives us information on only some of the caves about whether they are active or inactive. However, nowhere can I find a complete list of the nine caves … therefore I cannot tell whether the guanos sampled were from active or inactive caves. Table 2 appears to lump them all together. Surely, a guano that is actively accumulating will have a somewhat different chemistry from a guano that is relict?

I also have many issues with the discussion section …

Line 328: “Acid solutions generated by the decomposition of guano and possible associated microbial activity produced various forms of corrosion in the floor and walls of those bat caves”. This raises several problems in my mind.

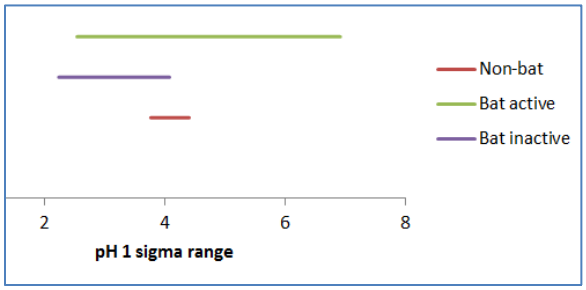

- Nothing whatsoever has been said in all of the results about *forms of corrosion*. These must be documented! … and we need to see that the corrosion forms in bat caves *are different* from those in non-bat caves.
- Since very little chemistry was done on waters from non-bat caves, how do we know that these do not also have acidic solutions? (an average pH of 4.1 for the non-bat waters might be considered to be rather acidic when compared to typical rain water pH of 5.7, and the pH values of the bat cave waters completely overlap the non-bat cave waters, and are not significantly different in a t-test.

Line 330: “bat caves were deeper, larger and bulkier”. Yes, this is shown by the numbers presented … but, as explained above, I feel that the choice of samples was not appropriate, and thus this point has not been adequately demonstrated.

Line 331:... the bat caves “had more abundant, diversified and bigger speleothems” – is absolutely not supported by any evidence presented. That is largely because the section describing speleothems gave no indication of relative distribution, or relative diversity, or relative size for the two difference cave types.

Line 331: “In an unprecedented example of bioengineering, we provided the first evidence that the long-term presence of bats (up to 23,000 years before present) and the guano deposits they produce mediated biological and chemical interactions which, by in turn, contributes to alter the geomorphology of those iron ore caves”. I have two comments:

- This is certainly not unprecedented! … There are now many publications reporting the erosional effects of bats/guano. This present study perhaps suggests a more significant role of bat guano in these particular caves, but similar studies have already been published. For example (one example of many), Figueira et al 2019 provide, for these same caves, analyses of speleothem, of guano, and of cave drip water, and attribute corrosion and deposition to bat guano … so this aspect of the present research is not unique. If there had been a clear demonstration of bat-v non-bat cave differences, then perhaps it might have been unique.
- Long-term presence? Yes, they may have shown that some of the guano is quite old (but that is nothing new), but no evidence is presented that the caves with younger guano have different features from the caves with older guano. So, their dating is interesting enough, but does not prove that the *duration* of bat presence has any significance.

Here I have stopped reviewing the manuscript. The rest of the discussion is reasonable .. but it is based on inadequate data. While I think the hypothesis is good and most probably correct, this manuscript does not offer adequate proof.

I am sorry that this manuscript is not more satisfactory. I think that the observations are interesting, but the research is poorly designed and cannot be published as it is. I lament that it has already been “published” in pre-print form.

I recommend that this manuscript be rejected.

I have attached two other files:

Review text with notes.docx (I copied the text into Word, since it is so much easier to make comments that way)

Supplementary material with notes.docx
