# Supplementary material for: Bats as ecosystem engineers in iron ore caves in the Carajás National Forest, Brazilian Amazonia
Source: PLoS One. 2023 May 11;18(5):e0267870. doi: 10.1371/journal.pone.0267870 (PMC10174506; doi:10.1371/journal.pone.0267870)
Supplement: Attachment — Submitted filename: PONE-D-22-11101 Supplementary Material with notes - Copy.pdf [file pone.0267870.s014.pdf]

Bats as ecosystem engineers in iron ore caves in the Carajás National Forest, Brazilian Amazonia

Luis B. Piló[1], Allan Calux[2], Rafael Scherer[3] & Enrico Bernard[1,*]

[1]Laboratório de Ciência Aplicada à Conservação da Biodiversidade, Departamento de Zoologia, Universidade Federal de Pernambuco, Recife PE, Brazil

[2]Carstografica – Karst Applied Research Centre, Campinas SP, Brazil

[3] Grupo Espeleológico de Marabá, Marabá PA, Brazil

* Corresponding author: enrico.bernard@ufpe.br https://orcid.org/0000-0002-2304-1978

Table S1: Caves studied in the Carajás National Forest, Pará State, Brazilian Amazonia, indicating those harboring large bat colonies (bat caves) and the presence of experessive guano deposits. Guano and water samples were collected from the caves for analyses on the pH and mineral composition.

Cave	Coordinates (SAD69/22M)		Samples (identification)	Bat cave?	Expressive guano deposits?
	UTM E	UTM N			
CAV-0041	570340	9290825	2 (T01, T02)		
CRIS-0020	645071	9289468	1 (T02)		
N5SM2-0099	596307	9321757	2 (Tite, TiteA)	+	+
N3-0023	586379	9331983	5 (A5/S, A6/S, TITE, TM-A, TM-B)	+	+
N3-0074	585168	9331968	1 (Tite)	+	
N4E-0014	592917	9332512	1 (T01)		
N4E-0023	592031	9333013	1 (T01)		
N4E-0033	592972	9332288	1 (T01)		
N4E-0072	593664	9333140	4 (T01, T02, T03, T04)		
N4E-0092	593106	9332366	5 (T01, T02, T03, T04, T05)		
N4WS-0015	589697	9329388	1 (Tite)		
N4WS-0067	589452	9328690	11 (Tite, Titea, A, S, T-02, T-03, T-05, T1, T02, T03, T04, T05)	+	
N4WS-0072	589672	9328580	3 (Tite, A, A2/S)	+	+

Comment [t1]: It would be so much easier for us, if you simply listed all the bat caves first I have shaded them so that I can see them more clearly (bat + guano = fawn; bat, no guano = pink)

Comment [t2]: same comment as in text

Comment [t3]: It would be very useful for us to be able to see which sample is guano and which is water. The sample codes mean little to us.

Formatted Table

Formatted Table

N5E-0002	597594	9328450	4 (AM-09, AM-11, AM-12, AM-13)		
N5E-0003	597245	9327356	4 (AM-15, AM-15-1, AM-16, AM-16a)		
N5E-0005	596329	9327242	2 (AM-22, AM-23)		
N5S-0004	595814	9325049	1 (AM-22)		
N5S-0014	595871	9325070	1 (AM-07)		
N5S-0015	595886	9325086	1 (AM-08)		
N5S-0017	597412	9327012	1 (AM-32)		
N5S-0021	596716	9327033	2 (AM-35)		
N5S-0063	595682	9325264	2 (AM-35-1)		
NV-0011	621068	9285015	1 (T02)		
S11-0007	583854	9286363	1 (T01)		
S11A-0036	560311	9301740	1 (Tite)	+	+
S11B-0094	567130	9298208	4 (Mite, A2/S, C2, C2a)	+	+
S11C-0041	568524	9294526	2 (A3/S, A5/S)	+	+
S11D-0001	571128	9292715	2 (T01, T02)		
S11D-0012	570832	9292964	3 (T01, T02, T03)		
S11D-0047	575721	9291345	2 (T01, T02)		
S11D-0055	575188	9291828	1 (T01)		
S11D-0078	575634	9293373	3 (T01, T02, T03)		
S11D-0083	574802	9292886	7 (T01, T02, T03, T04, T05, A2/S, A4/S)	+	+
S11D-0094	575039	9293131	1 (T01)		
S11D-0096	574738	9293228	1 (T01)		
S11D-0101	570402	9293694	3 (T01, T02, TO3)		

Table S2. pH of superficial and circulating water samples collected in caves in the Carajás National Forest, Pará State, Brazilian Amazonia. Caves included active and inactive bat caves holding large colonies of bats. UTM coordinates of caves provided.

Sample	Cave status	UTM SAD69		pH
M2-0019-W03	active bat cave	596258	9321716	6.3

Formatted Table

Formatted Table

Comment [t4]: The term "active" is often given to a cave that shows active geomorphological processes – such as a river. and "inactive" would refer to a high level, dry cave. However, in this case, I presume that "active" means it has a viable population of bats.

Comment [t5]: If it is inactive, how can it be holding large colonies of bats?

Comment [t6]: I shaded each cave so that I could see each more clearly, but the whole table is too long, with repetition of information on each line. See below

M2-0019-W04	active bat cave	596258	9321716	7.58
M2-0099-W01	active bat cave	596258	9321716	5.07
M2-0099-W02	active bat cave	596258	9321716	6.79
M2-0099-W03	active bat cave	596258	9321716	1.46
M2-0099-W04	active bat cave	596258	9321716	1.48
M2-0099-W05	active bat cave	596258	9321716	3.21
M2-0099-W06	active bat cave	596258	9321716	6.68
M2-0099-W07	active bat cave	596258	9321716	7.12
M2-0099-W08	active bat cave	596258	9321716	7.38
S11A-0036-W01	active bat cave	560261	9301699	6.91
S11A-0036-W02	active bat cave	560261	9301699	4.93
S11A-0036-W03	active bat cave	560261	9301699	2.88
S11A-0036-W04	active bat cave	560261	9301699	2.89
S11A-0036-W05	active bat cave	560261	9301699	2.5
S11A-0036-W06	active bat cave	560261	9301699	4.87
S11A-0036-W07	active bat cave	560261	9301699	7.2
S11A-0036-W08	active bat cave	560261	9301699	2.77
S11A-0036-W09	active bat cave	560261	9301699	2.7
S11A-0036-W10	active bat cave	560261	9301699	2.86
S11A-0036-W11	active bat cave	560261	9301699	2.9
S11B-0168-W01	active bat cave	560261	9301699	7.59
**N3-0023-W01**	inactive bat cave	586379	9331983	3.79
**N3-0023-W02**	inactive bat cave	586379	9331983	3.36
**N3-0023-W03**	inactive bat cave	586379	9331983	2.56
**N3-0023-W04**	inactive bat cave	586379	9331983	2.7
**N3-0023-W05**	inactive bat cave	586379	9331983	4.02
**N3-0023-W06**	inactive bat cave	586379	9331983	3.06

**N3-0023-W07**	inactive bat cave	586379	9331983	2.76
**N3-0023-W08**	inactive bat cave	586379	9331983	2.98
**N3-0023-W09**	inactive bat cave	586379	9331983	3.53
**N3-0023-W10**	inactive bat cave	586379	9331983	2.67
**N3-0023-W11**	inactive bat cave	586379	9331983	2.24
**N3-0023-W12**	inactive bat cave	586379	9331983	2.45
**N3-0023-W13**	inactive bat cave	586379	9331983	2.8
**N3-0023-W14**	inactive bat cave	586379	9331983	2.53
**N3-0023-W15**	inactive bat cave	586379	9331983	2.41
**N3-0023-W16**	inactive bat cave	586379	9331983	2.8
**N3-0023-W17**	inactive bat cave	586379	9331983	3.23
S11B-0094-W01		567130	9298209	3.55
S11B-0094-W02		567130	9298209	3.26
S11B-0094-W03		567130	9298209	3.96
S11B-0094-W04		567130	9298209	2.4
S11B-0094-W05		567130	9298209	2.73
S11B-0094-W06		567130	9298209	3.16
S11B-0094-W07		567130	9298209	5.78
S11B-0094-W08		567130	9298209	3.13
N4WS-0067-W01		567130	9298209	5.99
N4WS-0067-W02		567130	9298209	2.2
N4WS-0067-W03		567130	9298209	2.37
**N5S11-W01**	non-bat caves	596301	9325116	4.09
**N5S11-W02**	non-bat caves	596301	9325116	3.81

Comment [t7]: ? why no designation of type of cave? The manuscript text seems to suggest this is a bat cave

Comment [t8]: same UTM as previous cave, but different code??

**N5S11-W03**	non-bat caves	596301	9325116	3.93
**N5S12-W01**	non-bat caves	596768	9325320	3.91
**N5S12-W02**	non-bat caves	596768	9325320	4.08
**N5S12-W03**	non-bat caves	596768	9325320	4.7
LA-01	superficial waters	569401	9292849	5.05
LA-02	superficial waters	569401	9292849	5.02
LC-01	superficial waters	560900	9298124	5.11
S11C-1	superficial waters	568000	9940742	4.49
S11C-1	superficial waters	568000	9940742	4.61
S11C-2	superficial waters	568214	9950682	5.47
S11C-3		568119	9295436	4.69
S11B-1		567955	9295993	5.01
LRT-1		566851	9297887	5.52
BC-1		563651	9298893	4.82

A few more comments:

Table S2 shows only six water samples from only two non-bat caves (what about the other 24 that were supposed to have been studied?), but 22 from active bat caves along with 28 from inactive bat caves.

Table S2 gives no designation for sampling sites in S11B-0094, but the text implies that it is a bat cave, as does Table S1.

Table S2 … lots of space taken up for repetition of information. I shaded each cave so that I could more easily see them. The cave status and UTMs are repeated for every sample in that cave …

Table S2 could be more compact with only three lines for each location – something like:

Cave: M2-0019, active bat cave. UTM SAD69: 596258 9321716									
Sample	W03	W04	W01	etc					
pH	6.3	7.58	5.07	etc					
Cave: S11A-0036, etc.									
Sample	W01	W02	etc						
pH	6.91	4.93	etc.						
