# Supplementary material for: Bats as ecosystem engineers in iron ore caves in the Carajás National Forest, Brazilian Amazonia
Source: PLoS One. 2023 May 11;18(5):e0267870. doi: 10.1371/journal.pone.0267870 (PMC10174506; doi:10.1371/journal.pone.0267870)
Supplement: Attachment — Submitted filename: Answers to the reviewers.pdf [file pone.0267870.s017.pdf]

Recife, Brazil, January 23rd 2023.

Dear Editor,

Please find attached the corrected version of the manuscript PONE-D-22-11101R2 “Bats as ecosystem engineers in iron ore caves in the Carajás National Forest, Brazilian Amazonia”. We have addressed all the suggestions made by the reviewers. Bellow you will find a detailed lists of corrections. This was the fourth revision session of our manuscript and we hope that no other point is raised now so the manuscript can be finally published. Thank you very much.

Enrico Bernard

Request from the Editorial Staff:

We noted that a large quantity of rare speleothems, a non-renewable resource, were sampled for this study. Please provide ethical and scientific justification for the speleothem's sample size and a copy of your ethics documentation approving the collection of the speleothems (and an English translation, if the original is not in English language).

>> As stated in our previous answers to the reviewers, we once again clarify that speleothems, including stalactites, stalagmites and crusts, **were not collected in an unplanned, excessive or aleatory approach**. Speleothems were collected from **specific caves and, whenever possible, those speleothems collected provided multiple samples used for different purposes**, including chemical analysis, radiocarbon dating and the preparation of cutting profiles for stratigraphic analyses. Several of the samples we analyzed provided first data for Brazil, justifying their collections. As specified before, Table S2 details the caves sampled and the methodology approach in each of them. A total of **18 stalactites, stalagmites and crusts** were collected, producing **108 samples** for analysis. Reviewer #1, which originally raised this concern, was likely induced to a misinterpretation because our original text was not clear enough. He very likely was induced to confound samples with collections. Anyways, we had clarified that in the next corrected version of the manuscript and, once again, here. All collections were strictly authorized by CECAV/ICMBio, the federal institution responsible for cave research and protection in Brazil. Besides the original legal permit, **we have now attached an official letter from CECAV/ICMBio confirming that sample collections were in accordance with current legislation and authorized, with no objections, by the federal agency.** A translated version of that letter is also presented.

Reviewers' comments:

Reviewer #4

1 - The discussion between authors and previous reviewers about the way of writing chemical formula can be simply addressed by referring to the IMA conventional writings, which is the only acceptable way for minerals.

>> Please note that, contrary to the previous reviewer which raised such point, our formulae were originally presented in strictly accordance with IMA conventional writings (http://cnmnc.units.it/). We have double checked and corrected three typos (incorrect space between elements, missing digit). All other formulae were originally correctly presented.

2 - 202-203: it is not clear which material has been dated on phosphate speleothems using 14C method. Phosphate or organic matter trapped in?

>> The organic matter trapped in. Clarified now along the line.

3 - Uncorrected spellings: 333 jarosite; 344 taranakite; 373 phosphosiderite.

>> All corrected now. Thank you.

4 - 506 : why water percolating across iron is heated? Exothermic reaction??

>> No. The iron formation in Carajás is covered by iron-rich breccia generically known as canga, which act as caprock on plateau tops. Dripping is significant inside the caves due to their proximity to the surface and porosity of the canga and this layer tends to become quite hot due to the absorption of solar radiation. Therefore, the percolating water is heated not by exothermic reaction, but due to the higher temperature of the canga.

5 - Fig 6 and 7 from the initial version have been removed in the revised version. However, from the caption, it seem these illustration could provide additional morphological consideration (fig 6), and a conceptual model (fig7), however I haven't seen these figures…

>> Please note that Figs. 6 and 7 were not removed in the revised version. They are still present and this was probably a mistake caused by the uploading system.

Reviewer #5

1. Keywords: "Biogenenic corrosion" should be revised as "Biogenic corrosion"; "bat nesting" should be revised as "bat roosting" on Line 526; "nesting bats" should be revised as "roosting bats" on Line 531.

>> All corrected. Thank you.
