# Supplementary material for: Bats as ecosystem engineers in iron ore caves in the Carajás National Forest, Brazilian Amazonia
Source: PLoS One. 2023 May 11;18(5):e0267870. doi: 10.1371/journal.pone.0267870 (PMC10174506; doi:10.1371/journal.pone.0267870)
Supplement: Attachment — Submitted filename: Answers2.docx [file pone.0267870.s016.docx]

Recife, Brazil, September 12^th^, 2022

Dear Miquel Vall-llosera Camps, Senior Editor

After my appeal on the editorial decision for the manuscript PONE-D-22-11101R1, and following your instructions, I would like to submit a corrected version of the manuscript. Regarding Rviewer #3´s comments, these are our answers:

1. In 'Materials and Methods', X-ray diffraction and X-ray spectrometry were mentioned. However, no real data were presented except the list of the minerals in 'Results: Speleothems characterization'. Without proper presentation of the data, it was impossible to review the characterization part of the manuscript. It must be challenging to systematically present the experimental evidence due to a large number of samples.

>> In fact, as clearly stated in our Methodology, that part of the analysis was conducted at the University of São Paulo´s Escola Politécnica, in Brazil, a private lab hired for us to do that task. **We have now attached as Supplementary Material all the official reports issued by all the laboratories we have hired (DRX, FRX, ICP, organic chemistry, and radio carbon analyses).**

2. Reviewer #3 indicated a "lack of experimental data" as the reason to recommend the unfavorable decision.

>> Reviewer #3 did not present what lack of data is that. How can we, authors, contest this position if we don't even know what kind of data the reviewer is referring to? In case he/she is referring to the points raised by Reviewer #2, regarding expected matched samples (batcaves × non-bat caves), we once again emphasize the answers we provided before:

- We really would like to have had similar sample sizes (active bat caves x inactive bat caves x non-bat caves) to test our hypotheses in a statistically flawless approach, controlling all possible variables. However, in the real world this was not possible, since caves are unique and there are no two similar natural caves.
- We demonstrated that bat caves are 0.2% of 1,309 caves analyzed in Carajás National Forest. Therefore, it would be impossible for us to have a larger number of bat caves to match the number of non-bat caves in the region.
- We demonstrated that cave size alone does not explain bat choice for these specific caves: Other caves as large as the largest bat cave studied [see Figure S1] have no signs of previous occupation by bats.
- The deep corrosion signs, the richness and variety of speleothems described **were observed only in bat caves** (active or inactive).
- Speleothems in caves with no expressive guano deposits were of local occurrence and small, **while the greater diversity, abundance and size of speleothems was found only in the bat caves**.
- Once again, this reinforces how unique and singular bat caves and their associated processes are, precluding us to have similar standardized and heterogeneous sample sizes. Insisting that we would have similar number of caves for the three categories we analyzed (active bat caves, inactive bat caves, non-bat caves) to address statistical issues is, therefore, impossible.

3. Reviwer #3 indicated we had no experimental evidence to directly support the microbial activity.

>> In fact, we have no experimental evidence **because this was not our goal in this manuscript**. In our discussion, we clearly stated that biological activity very likely has an important role in the processes we observed. **Please check lines 426-457**. In that part of our discussion, we present evidence produced by other authors, including evidence for other iron ore caves in Brazil. **Testing biological activity is an open question in our field site and we had intention to approach that in the near future, but not in this paper**. Therefore, rejecting our paper because of the lack of evidence based on an analysis that was not our objective – but was clearly and correctly presented, discussed and supported by references – seems unfair to us.

4. Reviewer #3 mentioned “several typos”, without clearly indicating them, except for:

p11, final paragraph: M5SM2-0099 should read N5SM2-0099.

>> Corrected.

p17–: chemical formula for minerals. (e.g.) Fe3+(PO4) 2H2O looks unconventional. Usually, FePO4 2H2O.

>> We preserved the same right formulae provided in the reports issued by University of São Paulo´s Escola Politécnica. **Please check the now included reports**.

We have double-checked the entire manuscript for typos and did our best to detect and correct them.

Please note that our speleothem collections were all approved by the Centro Nacional de Pesquisa e Conservação de Cavernas CECAV, the federal agency responsible for that kind of permit. All our analysis were strictly under the law and we have now provided copies of the issued permits.

Based on the answers provided above, please consider our revised version.

Thank you,

Enrico Bernard
